# Umbrella Reviews Conducted in an Oncology Healthcare Context Focusing on Supportive Care, Systems, and Models of Care: A Review of Umbrella Reviews

**DOI:** 10.1002/cam4.71708

**Published:** 2026-03-25

**Authors:** İrem Koç, John Goodwin, Josephine Hegarty

**Affiliations:** ^1^ College of Medicine and Health, Cancer Research University College Cork Cork Ireland; ^2^ Department of Nursing, Faculty of Health Science Yeditepe University Istanbul Turkey; ^3^ School of Nursing and Midwifery University College Cork Cork Ireland

**Keywords:** cancer, models of care, supportive care, survivorship, umbrella review

## Abstract

**Aims:**

Healthcare providers need access to evidence‐based knowledge to deliver interventions effectively within clinical settings. This paper aims to provide an overview of umbrella reviews (URs) conducted in an oncology healthcare context. In this review, the oncology healthcare context relates to focusing on care delivery systems, supportive care, supportive care needs, and supportive care interventions, systems, and models of care for cancer survivorship care and support.

**Methods:**

This overview followed the Preferred Reporting Items for Overviews of Reviews (PRIOR) statement. The literature search was performed in seven databases including PubMed, CINAHL, APA PsycArticles, APA PsycInfo, SocINDEX with Full Text, Embase, and Cochrane Database from 2012 to 2023. The search terms and eligibility criteria were organized around the PCC framework. The JBI Critical Appraisal Checklist for Systematic Reviews and Research Synthesis was used for quality assessment.

**Results:**

Seventy‐six umbrella reviews were included in this review. Only four reviews demonstrated low quality. Tables and bubble maps helped to outline the key findings of included URs. Recommendations to aid future studies and reviews were summarized into five overarching themes.

**Conclusions:**

In conclusion, we outlined the range and gaps in topics covered, as well as the key features of umbrella reviews examining oncological supportive care systems and models of care over the past decade. Future research should address the multidimensional nature and complexity of clinical implementation and methodological concerns; consider underrepresented populations and be more targeted to inform policy development.

## 1 Introduction

It is reported that the global output of publications surpassed three million articles in 2022 [1]. The increasing rate of publishing new scientific evidence and the unprecedented pace of making such evidence available open access means that health care professionals have access to huge volumes of information at their fingertips. However, having access to large volumes of information can be confusing and difficult to interpret for healthcare professionals, policy makers, and patients. Umbrella reviews (URs) were developed as part of the solution as the umbrella review synthesizes information from numerous systematic reviews providing a summative overview of a large body of evidence [2]. Various approaches exist for conducting different types of literature reviews, each described by their unique methodologies, outcomes and methods of synthesis [3]. Systematically conducted reviews represent the most commonly employed approach, within these approaches [4]. Systematic reviews and meta‐analyses (SRMAs) hold an elevated status in the evidence hierarchy [5]. Currently, clinicians utilize SRMAs for the purpose of accessing robust evidence to underpin practice [6]. A well‐conducted systematic review can be practicable in keeping healthcare providers current and up to date in their field [7]. Nonetheless, the increasing number of SRMAs in recent years has led to uncertainty among decision‐makers and decision makers spending a lot of time sourcing and collating evidence.

This has occurred for several reasons principally because of the varying conclusions of authors across SRMAs in terms of the synthesis methods used and the associated low levels of confidence in the quality of the evidence making clinical decisions more difficult. Supplementary phases for synthesis are required given the increasing volume of systematic reviews giving rise to an increase in the volume of published URs [8].

Globally people are living longer, the prevalence of numerous cancer risk factors is also increasing in parallel which means more people are being diagnosed with cancer each year. The global burden to health systems of cancer and cancer treatments and individual level cancer morbidity is rising. An estimated 18.5 million new cancer cases and 10.4 million cancer‐related deaths were reported worldwide in 2023 [9]. The development of medical technologies and improvements in early screening increase the rates of recovery and survival [10, 11]. Supportive care interventions for individuals with cancer are expected to develop and extend over time [12, 13]. Clinical health care teams require access to evidence to effectively implement the right interventions within clinical environments. Dependable information is crucial for addressing symptom management, providing supportive care, addressing survivorship needs, and enhancing quality of life [12]. Thus, there is a more pressing need to synthesize such evidence for clinicians, patients, researchers, and decision‐makers [13]. The primary aim of this paper is to describe the areas of focus of URs conducted which focus on care delivery systems, supportive care, supportive care needs and supportive care interventions, systems and models of care for cancer survivorship supportive care. Perspectives on the added benefits, strengths and weaknesses of such approaches, types of conclusions drawn by authors and areas for further development were highlighted.

## 2 Methods

### 2.1 Protocol and Registration

This review followed the Preferred Reporting Items for Overviews of Reviews (PRIOR) statement (Data S1) [14]. Details of the review methodology have been registered with the Open Science Framework (OSF) to improve transparency (https://doi.org/10.17605/OSF.IO/ZABW5).

### 2.2 Problem Identification

URs are increasingly being used to assist in combining the evidence from multiple systematic reviews particularly in healthcare contexts to inform decision‐making. Such overarching reviews provide useful synthesis of the literature to guide others interested in viewing the totality of the evidence in an area, but who may not have the time or skills to combine the data themselves. This UR broadly builds on the work of Aromataris et al. [15] when they looked at methodological developments in the conduct and reporting of umbrella reviews.

### 2.3 Search Strategy and Eligibility Criteria

A literature search was performed in seven key databases, including PubMed, CINAHL, Embase, and the Cochrane Database of Systematic Reviews, as well as APA PsycArticles, APA PsycInfo, and SocINDEX with Full Text, which were accessed via EBSCO, with date limits from January 2012 to August 2023. Gray literature was not included. We updated the electronic search in July 2024, expanding the available search from September 2023 to December 2023, using a similar search strategy. The search terms and eligibility criteria were organized around the PCC (Population [or participants], Concept, Context) framework (Table 1) [17, 18]. Population included individuals with “Cancer” and the core concept of interest was “Umbrella review.” In terms of context no search terms or limiters were applied. Sources from any country, region, cultural context were of interest to the authors. The search used the key search strings, and a title only search function as it is anticipated that the population and methods would be part of the title of a publication. The search was exported to Covidence for a two‐stage screening. All screening were conducted independently and in pairs by two authors (I.K & J.H.). Consensus were reached with a third reviewer (J.G.) if disagreements occurred.

**TABLE 1:** Using population (or participants), concept, context (PCC) to outline key search terms and eligibility criteria.

PCC	Title search for terms	Inclusion criteria	Exclusion criteria
Population	Cancer OR neoplasm OR adenocarcinoma OR tumor OR tumour OR malign* OR carcinoma OR choriocarcinoma OR metastat* OR sarcoma OR oncology OR leukaemi* OR leukaemia OR lymphoma	Individuals with a diagnosis of cancer of any age, and any cancer diagnosis, at any stage of cancer survivorship. Family and careers of individuals with cancer	Benign tumors. Pre‐cancerous conditions. Focused on cross‐disease approaches.
Concept	No specific search terms	Oncology healthcare context focusing on supportive care; supportive care interventions; managing symptoms and concerns; improving quality of life; understanding and addressing supportive care needs; systems and models of cancer care and models of survivorship care	Umbrella review (UR) with a focus on understanding the physiological/biological/pathological/cellular basis of cancer, cancer symptoms and their treatment. Association of cancer with other conditions, for example, Alzheimer's disease and diabetes. Laboratory based. Intercellular communication pertaining to the tumor microenvironment. Biomarkers. Vaccine response and safety in patients with cancer. Prediction of cancer risk; exposures and factors linked to cancer risk, cancer outcomes and cancer treatment outcomes. Prediction models. Models focusing on prognostic factors. Screening and tests for detection of cancer. Focus on help seeking or delays in diagnosis. Focused on prevention of cancer in the general population. Efficacy, safety and cost‐effectiveness of diagnostic tests, artificial intelligence and imaging and radiological approaches. Efficacy, safety and cost‐effectiveness of surgical approaches, radiotherapy, chemotherapy, immunotherapy, targeted therapies, systemic treatments, pharmacological treatments for cancer. Stem cell transplantation and pharmacogenetics. Health technology assessments. Education and training of health care professionals. Chinese medicine or alternative therapy‐based interventions for preventing and treating cancer. Overview, evaluation, psychometric properties and methodological limitations of survey tools/measures/patient reported outcomes. Interventions targeting knowledge of or behaviors of health care professionals. Focused on diagnosis and impact of COVID19 on diagnosis and delayed diagnosis. Reviews focused on cost effectiveness of treatments
Context	No specific search terms	Any context 10 years publication time frame from January 1st, 2012, to 31st December 2023#	
Design	“Umbrella review” OR “review* of review*” OR “review* of systematic review*” OR “overview* of review*” OR “overview* of systematic review*” OR “synthes* of review*” OR “synthes* of systematic review*” OR “summar* of systematic review*” OR “meta‐review”	Umbrella review or “systematic review of systematic reviews” or meta‐review	Other types of synthesis of systematic reviews which are not termed as umbrella reviews (URs) or overview or review of systematic reviews. Protocols

*Note:* *Truncation is used to broaden the search and pick up words with other endings in database searches. #January 2012 was selected as the lower boundary because umbrella reviews on oncology care have expanded substantially over the past decade (Nayak et al. [16]). Reviews published prior to this date were less likely to reflect contemporary oncology practice.

### 2.4 Data Extraction and Assessment

Data extraction was completed by one person (I.K.) and checked by a second (J.H. or J.G.). Any disagreements were resolved by a third person.

Key data extracted included authors, year; number and type of included papers within the UR and details of meta‐analysis completed; focus of the UR; the methodological framework used to support the UR; overview of quality assessment processes; details of pre‐registration of the umbrella review; details of the assessment of overlap between systematic reviews; definition of UR; perspectives on the added benefits, strengths and weaknesses of doing an UR; format of the presentation of the results (tabular and/or visual); high level detail of the conclusions drawn by authors and areas of further development highlighted by authors. A detailed data extraction table and references of included URs is provided in Table S1.

The Joanna Briggs Institute Critical Appraisal Checklist for Systematic Reviews and Research Synthesis was used for quality assessment [15, 19]. The checklist covered 11 questions, including compatibility of the review question, inclusion criteria and search strategy. The checklist also assessed how the quality assessment was done, what strategies were used to reduce data extraction errors, and the likelihood of publication bias. If a paper searched more than six databases, we accepted that it utilized adequate resources; we selected “yes” for the fourth question (Were the sources and resources used to search for studies adequate?). Each question was answered as “yes,” “no,” or “unclear.” Not applicable “NA” also was used in rare instances. The quality assessment table and references of included URs are provided in Table S2.

### 2.5 Data Analysis and Presentation of Findings

We summarized the details of the included publications using an umbrella review of umbrella review approach [14]. Data were presented in tabular format, with summative data presented on key study characteristics. Bubble maps were created using Excel for Microsoft 365 (Version 2406) to visually display details of the key characteristics of included URs. Methodological deficits or weaknesses/limitations of included URs were presented as frequencies (*n*, %). A narrative synthesis was performed to summarize recommendations for future reviews and research.

## 3 Findings

**FIGURE 1:**
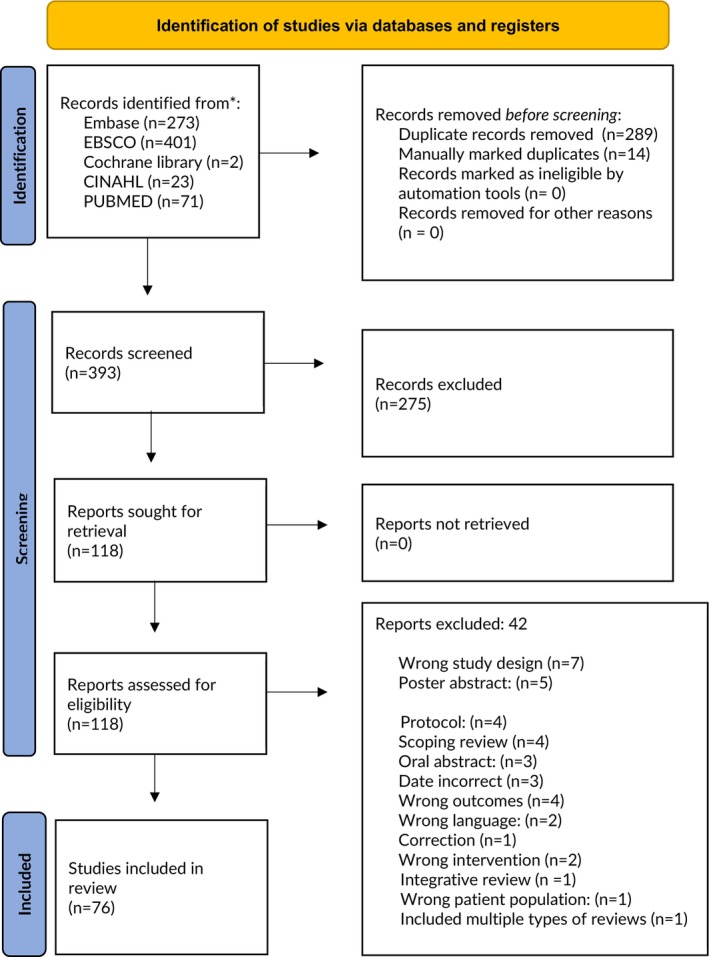
PRISMA flow diagram of study selection, adapted from [20].

Citations (*n* = 770) were exported to Covidence. After duplicates were removed, the remaining 393 citations were screened, and 275 papers were deemed irrelevant. Over 118 full‐text papers were reviewed for eligibility, and finally 76 URs were included (Figure 1). We provided details of the full text excluded papers as a list in the Table S3.

### 3.1 Study Characteristics

Table 2 summarizes details of included URs. Our detailed data extraction table is provided in Table S1.

**TABLE 2:** Included umbrella reviews details.

Characteristics of included URs	Findings
Year of publication	2023 40.7% (*n* = 31). 2022 18.4% (*n* = 14). 2020 10.5% (*n* = 8). 2021 9.2% (*n* = 7). 2015 6.5% (*n* = 5). 2019 5.2% (*n* = 4). 2018 3.9% (*n* = 3). 2016 2.6% (*n* = 2). 2017 1.3% (*n* = 1). 2014 1.3% (*n* = 1)
Topics covered by aim	Evaluating current evidence on interventions 84.2% (*n* = 64). Symptom management 28.9% (*n* = 22). Provision of supportive care/management of needs 17.1% (*n* = 13). Quality of life improvement initiatives 11.8% (*n* = 9). Models of care, pathways, organizational/system focus 10.5% (*n* = 8). Optimize experiences and meet the needs of caregivers, and family members 6.5% (*n* = 5). Unmet needs/unmet supportive care needs 2.6% (*n* = 2)
Type of included papers	Systematic reviews (SRs) 22.3% (*n* = 17). SRs + Meta‐analysis 75% (*n* = 57). SRs + Meta‐synthesis 6.5% (*n* = 5). SRs focused only on including trials 50% (*n* = 38)
Details of UR synthesis completed	Narrative synthesis 38.1% (*n* = 29). Quantitative synthesis 28.9% (*n* = 22). Thematic qualitative synthesis 10.2% (*n* = 7). Evidence mapping 1.3% (*n* = 1). Not reported 23.6% (*n* = 18)
The methodological framework used to support umbrella review	PRISMA guidelines 48.6% (*n* = 37). Cochrane handbook guidance 28.9% (*n* = 22). JBI guidelines for UR6.5% (*n* = 5). PRIOR guidelines 9.2% (*n* = 7). Not reported 22.3% (*n* = 17)
Quality assessment tools	AMSTAR‐2 50% (*N* = 38). AMSTAR 21% (*n* = 16). GRADE approach (Cochrane) 26.3% (*n* = 20). JBI critical appraisal checklist 11.8% (*n* = 9). ROBIS tool 10.5% (*n* = 8). PRISMA guidance and checklist as a quality metric 11.8% (*n* = 9). Cochrane tool risk of bias (ROB) 2.6% (*n* = 2). Not reported 2.6% (*n* = 2)
Pre‐registration of the umbrella review	Yes 57.8% (*n* = 44). No 1.3% (*n* = 1). Protocol drafted, included as a supplementary file, but not pre‐published 5.2% (*n* = 4). Not reported 35.5% (*n* = 27)
Method used for the assessment of overlap between SRs	CCA (calculated corrected area) 15.7% (*n* = 12). Manually calculated 3.9% (*n* = 3). Not reported 77.6% (*n* = 59)
Population covered by aim	Adult patients 60.5% (*n* = 46). Mixed age sample as patients 46% (*n* = 35). Children and adolescents 6.5% (*n* = 5). Children only 1.3% (*n* = 1). AYA (adolescents and young adults) 3.9% (*n* = 3). Carers 5.2% (*n* = 4). Health care professionals 5.2% (*n* = 4). Family members 2.6% (*n* = 2)
Cancer diagnosis	Mixed cancer groups (cancers specified in list below) 42.1% (*n* = 32). Single cancer type 32.8% (*n* = 25). Breast 52.6% (*n* = 40). Lung 31.2% (*n* = 24). Digestive organs including colorectal 27.6% (*n* = 21). Prostate 26.3% (*n* = 20). Lymphoid, hematopoietic, and related tissue 22.3% (*n* = 17). Oral cavity and pharynx/head and neck 19.7% (*n* = 15). Mixed cancer sample (cancers not specified) 25% (*n* = 19)
Stages of cancer survivorship	Mixed phases on cancer survivorship journey 50% (*n* = 38). Acute survivorship (during treatment) 34.2% (*n* = 26). Adjuvant treatments (≤ 5 years post treatment) 31.5% (*n* = 24). Extended cancer survivorship beyond 5 years after treatment 23.6% (*n* = 18). Focus on palliative care 7.8% (*n* = 6). Metastatic cancer or advanced cancer 7.8% (*n* = 6). Focus on end of life 2.6% (*n* = 2). Not reported 27.6% (*n* = 21)
Format of the presentation of the results	Tabular 100% (*n* = 76). Visual figure/graph/picture 93.4% (*n* = 71). Mapping with heat maps as an example 2.6% (*n* = 2)
Perspectives on the added strengths of the umbrella review	Over‐view of evidence provided and summarized into UR 94.7% (*n* = 72). First to synthesize level 1 evidence on the topic area 26.3% (*n* = 20). Useful for clinicians 13.1% (*n* = 10). Overview reduces risk of bias and adds robustness to the area 14.4% (*n* = 11). A broad range of outcomes studied in the reviews included 6.5% (*n* = 5)
High level detail of areas of further development highlighted/recommendations for future	More research or trials needed 82.3% (*n* = 56). Survivorship interventions should be developed, tested and validated as rapidly as anticancer therapies are advancing 33.8% (*n* = 23). Recommended to report according to the acknowledged reporting standards to improve the quality of evidence 20.5% (*n* = 14). More focus on integrated care, shared care, care coordination across boundaries 11.7% (*n* = 8)
High level conclusion of the UR	Evidence from multiple systematic reviews demonstrated that targeted interventions benefit cancer survivors with specific symptoms 59.2% (*n* = 45). More research needed, with rigorous methodological designs 44.7% (*n* = 34). Multidimensional nature of interventions by combining different elements reinforces the effect 19.7% (*n* = 15). Findings show that there is lack of evidence 19.7% (*n* = 15)
Reasons given for not conducting meta‐analysis	Heterogeneity in methodologies of included reviews 14.4% (*n* = 11). Heterogeneity in outcomes or outcome measures 10.5% (*n* = 8). Heterogeneity in included interventions 6.5% (*n* = 5). Not reported 40.7% (*n* = 31)

Abbreviations: AMSTAR = A MeaSurement Tool to Assess Systematic Reviews; JBI = Joanna Briggs Institute; PRIOR = Preferred Reporting Items for Overviews of Reviews; PRISMA = Preferred Reporting Items for Systematic Reviews and Meta Analyses; ROBIS = Risk Of Bias In Systematic review.

The aims of the included URs addressed: summarizing and evaluating current evidence on interventions (84.2%), symptom management (28.9%), provision of supportive care/care to meet/manage particular needs (17.1%), models of care, pathways, coordination/organizational/referral/systems focus (10.5%), quality of life improvement initiatives (11.8%), experiences of or meeting needs of carers/caregivers or family members (6.5%), provision of information to meet particular needs (3.9%), unmet needs/unmet supportive care needs (2.6%), overall experience of illness/treatment (3.9%), prevention of adverse effects (1.3%).

**FIGURE 2:**
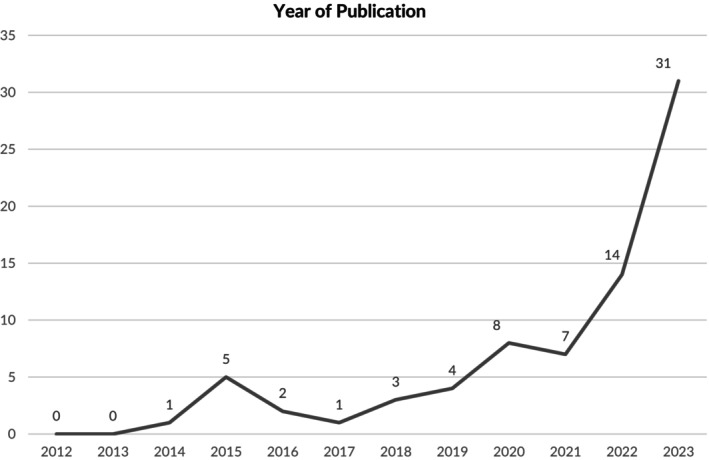
Year of publication. *y*‐axis shows number of included umbrella reviews, and *x*‐axis indicates year of publication.

The number of included URs increased each year from 2012 (*n* = 0), 2014 (*n* = 1) to 2023 (*n* = 31) (Figure 2). The Preferred Reporting Items for Systematic Reviews and Meta‐Analyses (PRISMA) guidelines or checklist was employed in 48.7% (*n* = 37) of the URs, indicating considerable commitment to standardized and transparent reporting practices.

Quality assessment using the AMSTAR 2 tool was conducted in 50% (*n* = 38) of included URs. In the large number of included URs, at least one quality assessment tool was used. However, there were few URs in which no quality assessment was performed (*n* = 2). Over half of the URs (*n* = 44) had a pre‐registered protocol. However, many of the included URs (*n* = 32) were not pre‐registered.

The main focus of the URs was on adult patients (*n* = 46) and mixed‐age patient samples (*n* = 35). A smaller group of URs included carers (*n* = 4), children and/or adolescents as patients (*n* = 5), and healthcare professionals (*n* = 4) as their sample. Mixed types of cancer (*n* = 32), and a single cancer group (*n* = 35) were the most frequent diagnosis represented in the URs.

A substantial number of the URs (*n* = 21), did not clearly specify the stage of cancer survivorship. A focus on mixed phases in the cancer survivorship journey (*n* = 38) was most common. A synthesis of the perspectives on the added benefits and strengths of conducting URs showed that the overview of existing evidence and the synthesis provided within the UR were viewed as particularly valuable. Furthermore, authors of 20 of the URs stated they were the first to synthesize level 1 evidence on the topic. The included URs emphasized numerous limitations including substantial overlap of included studies within reviews (*n* = 15), heterogeneity between different study designs (*n* = 15), and the inclusion of only English language studies (*n* = 24). The absence of reassessment of the quality of individual studies (*n* = 3) and employing limited specific search terms in the included reviews (*n* = 1) were also stated limitations, albeit to a limited extent. UR authors have emphasized that more research and more trials are needed (*n* = 61), outlining the need for the development of specific survivorship interventions in a substantial number of URs (*n* = 26). More focus on integrated care, shared care, care coordination across boundaries was recommended in eight URs.

The main conclusions of 59.2% (*n* = 45) of included URs was that targeted interventions benefit cancer survivors with specific symptoms. While 44.7% also highlighted the requirement for future research with robust methodological designs and sufficiently powered sample sizes (*n* = 34), and 19.7% reinforced the positive benefits of multidimensional interventions (*n* = 15). The high level of supportive care needs of family members or carers, and differences in information and supportive care needs between and within migrant and ethnic minority cancer patients and survivors were the main conclusions of a limited number of URs (*n* = 1 UR each). This suggests a limited focus on addressing the needs of family members and individuals from migrant and ethnic minority backgrounds within the reviewed URs.

### 3.2 Intervention Characteristics

**FIGURE 3:**
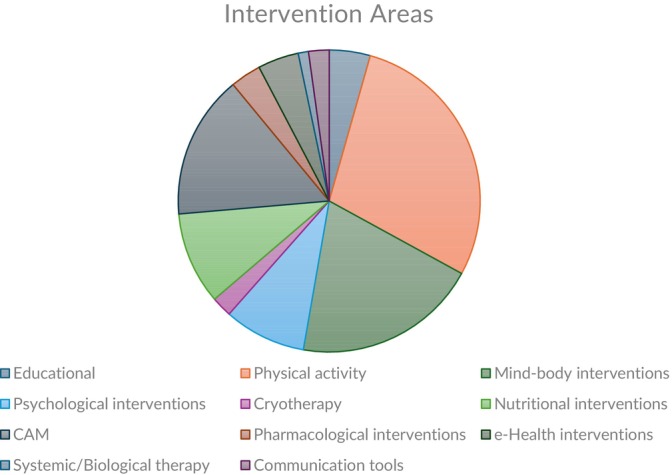
Intervention areas addressed within included URs.

Our findings showed that there was a huge diversity of non‐pharmacological interventions within the reviews, as we categorized them. The categorized interventions are outlined in Figure 3. Physical activity/exercises interventions (28%) combining aerobic and anaerobic phases were the most often summarized intervention.

The second most frequently targeted area was mind–body interventions (20%), which include yoga, mindfulness, massage, qigong, cognitive therapy, tai chi, music therapy, and hypnosis. Complementary and Alternative Medicine (CAM) interventions (15%) like acupuncture, meditation, Chinese herbal medicine, and reiki were the third major focus area. Nutritional interventions such as vitamins, probiotic supplements, were emphasized within some of the included reviews (10%). Psychological interventions (9%) combined with psychoeducational interventions were also investigated within reviews. Equal focus was assigned to eHealth/mHealth (5%) and educational interventions (5%). Pharmacological (3%), communication tools (2%), cryotherapy (2%), and systemic/biological therapy (1%) were approached less frequently.

### 3.3 Limitations of Included Umbrella Reviews

Our findings showed that included URs had several weaknesses/limitations. We classified these limitations into eight themes: extent and focus; heterogeneity/diversity; study and review quality; data and reporting; language and geographic limitations; methodology; sample size and population; and overlap (Table 3).

**TABLE 3:** Weaknesses/limitations of included umbrella reviews as reported by review authors and summarized using a frequency approach.

Extent and focus	Heterogeneity/diversity	Study and review quality	Data and reporting	Language and geographic limitations	Methodology	Sample size and population	Overlap
Limited focus of UR 3.9% (*n* = 3). Focus on limited cancer diagnosis9.2% (*n* = 7). Limited number of articles for inclusion in UR 9.2% (*n* = 7)	Heterogeneity across study populations/diagnosis within SRs 25% (*n* = 19). Heterogeneity across different study designs within included reviews 19.7% (*n* = 15). Heterogeneity of measurement tools within SRs 21% (*n* = 16)	Quality of included research within UR was low 23.6% (*n* = 18). Quality of included research within reviews was low 11.8% (*n* = 9). Poor transparency in reporting on ROB and/or quality appraisal 11.8% (*n* = 9)	Limited detail re cancer stage, stage of survivorship, treatments within SRs 14.4% (*n* = 11). Lack of detail in reporting of interventions making replication of studies difficult 10.5% (*n* = 8). Inconsistency in results within included studies 7.8% (*n* = 6)	Included reviews were only in the English language 31.5% (*n* = 24). Limited research conducted in Europe 1.3% (*n* = 1)	UR not focusing on primary research 11.8% (*n* = 9). Not suitable for subgroup analysis 9.2% (*n* = 7)	Limited ability to generalize from study findings 13.1% (*n* = 10). Small sample size within included studies within the reviews 10.5% (*n* = 8)	Significant overlap of including studies within reviews within UR 19.7% (*n* = 15)

Abbreviations: RoB = Risk of bias; UR = Umbrella review.

### 3.4 Quality Assessment

The quality assessment of the included URs is provided in Table S2. About 61 URs scored 8 or more out of 11 criteria (high‐quality); 11 URs met five to seven criteria (moderate quality) and 4 scored fewer than five criteria (low quality). Removing the low‐quality studies would not meaningfully change the results. Including all available umbrella reviews provided a more complete picture of the literature; this is especially important in summarizing data narratively and where understanding trends in emerging or niche topics is important.

### 3.5 Evidence Mapping

**FIGURE 4:**
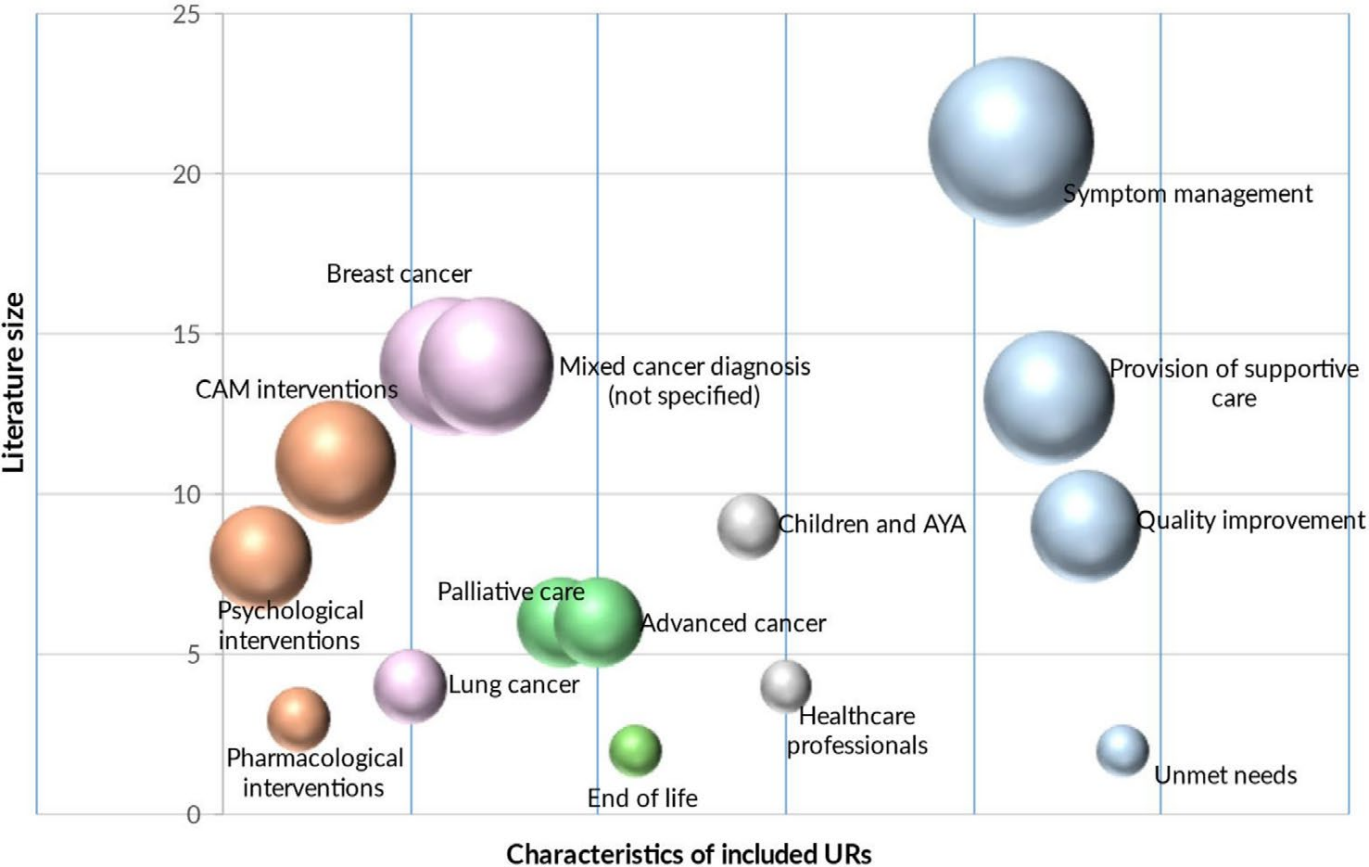
Bubble map 1. The key characteristics of included URs (i.e., intervention type, cancer diagnosis, stages of cancer survivorship, target population, and topics covered by aim) outlined on the x‐axis and literature size that is, the number of URs based are outlined on the *y*‐axis. Bubble colors: Intervention type (orange); diagnosis (light pink); stages of cancer survivorship (green); target population (gray); topics covered by aim (light blue). Quality of life improvement initiatives is abbreviated to quality improvement. AYA, adolescent and young adults; CAM, complementary and alternative medicine.

**FIGURE 5:**
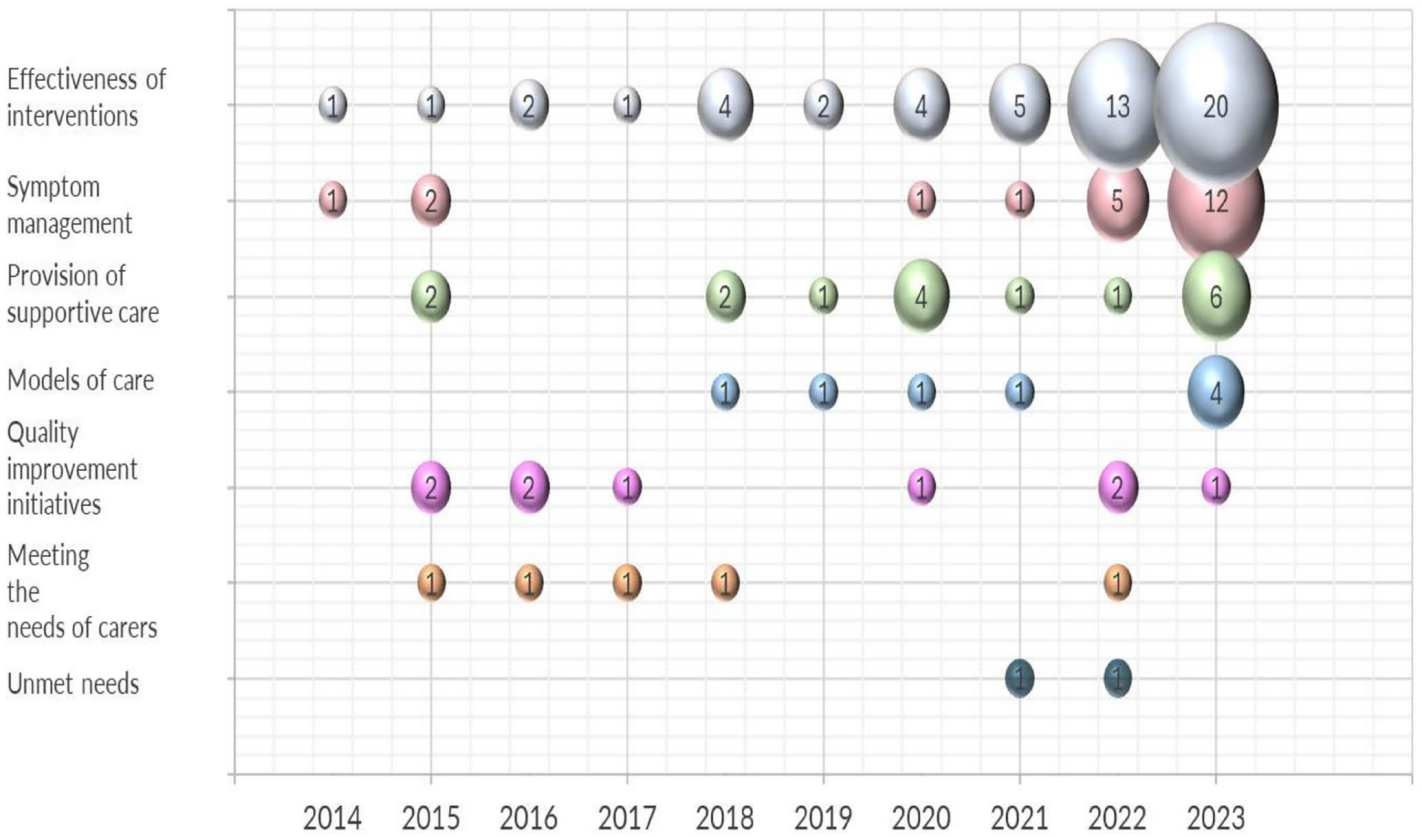
Matrix bubble map. This bubble map demonstrates the topics covered by aim (*y*‐axis) and their publication numbers per year (*x*‐axis). Bubble colors: Effectiveness of interventions (gray); symptom managements (light pink); provision of supportive care (light green); models of care (blue); quality of life improvement initiatives (lilac); meeting the needs of carers (orange); unmet needs (dark green). Quality of life improvement initiatives is abbreviated to quality improvement initiatives in the figure.

We described the key characteristics of included URs (Figure 4), and interventions on cancer‐related symptoms (Figure 5) utilizing bubble plots. The first bubble plots outline the number of included URs grouped by intervention type, cancer diagnosis, stages of cancer survivorship, target populations, and topics covered by aim. The most targeted populations were mixed cancer diagnosis details of which were not specified by authors (*n* = 19) and breast cancer as a single included diagnostic group (*n* = 15). However, lung cancer (*n* = 4) was the least addressed population within the included URs. Twenty‐two URs focused on symptom management. Provision of supportive care (*n* = 13) and quality of life improvement initiatives (*n* = 9) were more highlighted topics than unmet needs (*n* = 2).

The second matrix bubble map (Figure 5) indicates the topics covered by aim (*y*‐axis), including effectiveness of interventions, symptom management, provision of supportive care, models of care/system focus, quality of life improvement initiatives, optimizing to meet the needs of caregivers, and unmet needs and their frequencies year by year (*x*‐axis). The number of URs reflecting effectiveness of interventions (*n* = 13) and symptom management (*n* = 5) dramatically increased in 2022. Effectiveness of interventions (*n* = 20), symptom management (*n* = 12), provision of supportive care (*n* = 6), and models of care/system focus (*n* = 4) were the most addressed topics within included URs in 2023.

### 3.6 Recommendations for Future Research

We summarized recommendations for future reviews and research in Table 4. Our recommendations will aid in developing high‐quality evidence for future studies and reviews. We grouped the recommendations into 5 themes: future review efforts, future trials, future intervention and experimental studies, population and target samples, and future implementation by policymakers, healthcare managers, leaders, teams, and researchers. Details of the stages of iteratively grouping codes and creating themes and a listing of recommendations are provided as Supporting Information (Table S4 and Data S2).

**TABLE 4:** Recommendations for future reviews and research extracted and summarized from included URs.

Overarching theme	Subthemes
Recommendations for future review efforts	To aid the development of evidence informed policy and best practice guidelines, future reviews of literature should: Adhere to methodological and reporting standards and guidelines; comply with the PRISMA statement. Use standardized reporting of the parameters of the different interventions. Adhere more strictly to the AMSTAR‐2, PRISMA, and GRADE criteria for quality appraisal. Where appropriate consider selecting randomized controlled trials of higher quality and lower risk of bias to provide more rigorous evidence. Incorporate a wider range of research types and reviews (not only Cochrane reviews), to avoid overlooking high‐quality research that potentially contain unbiased and important recommendations regarding real world implementation contexts. Provide exclusion lists, and definitions of outcomes in their protocols. Report evidence based on separate objectives, interventional studies, and type of studies and explore subgroup analyses and potential subgroup effects through head‐to‐head comparisons. Explicitly report evidence gaps in primary research for outcomes in the domains of health promotion, managing chronic conditions, clinical structure, and decision‐making. Include a search of the gray literature, to reduce publication bias. It is recommended that future research and high‐quality trials should: Include large‐scale longitudinal research with long‐term follow‐up evaluations, and clearly defined targeted measurement indicators, strictly controlling bias involving patients who have survived cancer or live with chronic illness. Use ecological transient assessment to dynamically measure symptoms. Focus on various aspects of survivorship such as experiences of ongoing symptoms, financial toxicity, multi‐morbidity, and psychological issues, including fear of cancer recurrence, mental health disorders, and stigma. Focus on various aspects of care delivery and organization for example ensuring the effectiveness of survivorship care plans, the distress thermometer, and decision aids in improving patient participation in consultations; promoting shared decision making; and improving understanding, satisfaction, and treatment outcomes. Test accessibility, reach, uptake, fidelity, and scalability of the tools/interventions in large‐scale and real‐world settings. Assess whether positive impacts from interventions can be maintained in the long term in the real‐world setting. Develop symptomics using symptom network analysis. Clarify the quality of the evidence regarding the validity and reliability of tools and e measures in the assessment of outcomes such as fatigue within the target population, to support a more coherent and robust evidence base. Include larger, fully powered randomized controlled trials and meta‐analyses to provide cancer patients, survivors and their families with the optimum evidence to inform the best possible quality of life. Assess alternative outcome measures, and the difference between objective and subjective outcome measures to understand patients' interpretations of their conditions, and to enable early detection of side‐effects. Provide consensus around a core outcome set to measure psychological morbidity impact
Recommendations for future trials	To provide more useful data for evidence syntheses and clinical practice guidelines, future well reported randomized controlled trials should: Register with a recognized platform. Declare funding sources in future publications to reduce funding bias. Estimate the optimal sample size based on the existing research results, be fully powered to ensure sufficient sample size, aiming to ensure that the conclusions drawn from the research are valid. Include homogeneous samples of patients with cancer to allow for more definitive conclusions. Conduct blinding, allocation concealment and use placebo in the control group as appropriate. Explicitly report patient‐important outcomes. Adopt more specific quality of life measurement tools such as the Short Form 36 questionnaire, and the European Organization for Research and Treatment of Cancer Quality of Life Questionnaire‐series. Employ the incidence of adverse events as one of the main evaluation indices. Report implementation and results according to the CONSORT statement, to provide a high methodological quality
Recommendations for future interventions and experimental studies	Future intervention‐based studies should: Attain the perspectives of patients and all stakeholders in the co‐design of interventions and interventional studies. Seek to address the impairing effects of cancer treatments, support home and working life, and address patient's physical and psychological needs and optimize patients' experiences. Evaluate the short and long‐term effects of pharmacological interventions, dietary supplements, herbal medicine, complementary and integrative medicine interventions, nutritional therapies, aerobic/non‐aerobic exercise interventions, cryotherapy interventions, psychosocial interventions and web‐based interventions to enable more robust conclusions about the efficacy of interventions. This includes their impact on supporting the prevention and management of cancer and treatment related symptoms (e.g., pain, fatigue, and anorexia), symptom clusters, cancer recurrence and promoting health and wellbeing. Explore changes in symptom clusters or symptom networks over time during adjuvant treatment. Consider how individual can overcome barriers to engagement in physical activity and seek to evaluate suitable exercises for patients with poor exercise acceptance. Focus on spirituality, social connections, body image and coping strategies as outcomes in patients with cancer. Investigate the impact of interventions in a broader range of patient populations. Use telemedicine, tailored eHealth interventions, wearable health technology‐based interventions and educational interventions for symptoms such as cancer‐related fatigue, supporting chronic disease management, medication adherence and management, cancer screening, surveillance for recurrence, and disease prevention, whilst considering selected populations' digital health literacy. Focus on the development of novel interventions to improve patient‐physician communication and investigate communication competencies between patients and healthcare providers. Future well‐designed randomized controlled trials of interventions, in order to provide high certainty of the evidence, should: Use a comprehensive quality framework for standardized evaluation of interventions in cancer survivors. Focus on the effects of interventions on symptoms and assess symptoms regularly in clinical settings to aid in identifying effective therapies, treatments, and management strategies. Use The Medical Research Council framework for complex interventions. Be based on underlying theoretical model(s), behavior change techniques and consider factors which might influence and modify the effect of interventions. Use standardized reporting of the parameters of the different interventions and establish a systematic way of reporting and providing individualized interventions. Optimize patients' adherence to intervention protocols. Clarify the presence of short‐ and long‐term toxicities of interventions. Assess how to sustain intervention effects over a longer follow‐up period. Be more transparent in the description of complex interventions and routine care, trial design, adherence to trial protocol and intervention, including timing of assessment, the duration of the intervention. Describe the treatment protocol/intervention according to the TIDieR checklist (Template for the intervention description and replication), to help organize the reporting of interventions and facilitate replication in other trials. To enable translation of evidence into practice, future interventional research should: Explore the barriers to and facilitators of intervention implementation across various types of cancer patients at different stages. Focus on interventions that save resources and are relatively easy to implement in daily practice. Ensure long‐term follow‐up to increase intervention utilization and application in the clinical settings. Bridge the gap between various types of interventional research and application in clinical practice. Evaluate economic factors, including cost analysis and adverse events, to examine the potential cost‐effectiveness or cost‐minimization of interventions. Cost‐effective interventions should be translated and popularized in clinical practice. Develop standard operating procedures, standards and guidelines to guide implementors on the optimal approach to deploy interventions, and delivery methods. Compare the benefits of specific intervention implementation strategies compared with usual care in a population in real‐world settings
Recommendations for diverse diagnosis/patients	Future research should: Expand the populations included in research to include different cancer types, age ranges reflecting the lifespan, diverse languages, demographic groups, educational levels, and include population in remote, rural, or low‐resource settings. Prioritize inclusion of patients traditionally underrepresented in studies. Address the priorities of the patients and their families in low‐ and middle‐income countries. Consider the acceptability and effectiveness of interventions targeting people living beyond all types of cancer especially those with baseline poor overall quality of life. Expand the understanding of effective models of care in diverse cancer survivor populations including pediatric cancer survivors, adolescent and young adult survivor group, older adults, and a broader range of cancer types as well as advanced stages of the disease. Personalize the data according to each patient's needs and use “The International Classification of Functioning, Disability and Health model” as a common framework to help prioritize personalized goals for patients. Focus on the information and supportive needs, sensitive topics such as body image, and sexuality of migrant and ethnic minority cancer patients and survivors. Develop decision trees for selecting the most appropriate model of care for individual cancer survivors, along with implementation guides and standardized outcomes for evaluation. Address understanding the unique cultural factors of indigenous populations worldwide who are facing cancer. Consider breast cancer survivors with BRCA1/2 gene mutations, women receiving tailored treatments, and women from low socioeconomic backgrounds, their complex health care needs, and those experiencing late effects. Focus on specific tumor types and include direct comparisons between therapeutic options among specific cancer population clusters and trajectories
Recommendations for policymakers, health care managers, leaders, team and researchers to implement in the future	It is recommended that clinicians/policymakers/researchers should: Provide leaflets and brochures in the language of the patients or survivors affected by cancer. Become familiar with the beneficial effects of supportive interventions. Name specific personnel responsible for disseminating particular information addressing patients' needs. Implement industry‐standard data encryption for web‐based interventions, to ensure the security of private information. Evaluate the use of different technological platforms to provide telemedicine services. Integrate palliative care into primary care services, to improve access to palliative care for patients living in remote areas. Prioritize access to palliative care for patients with limited treatment options or persistent frailty despite rehabilitative attempts. Assess the country's readiness for the provision and integration of evidence‐based care and develop a sufficient body of evidence to support policy development. Collaborate with national and international organizations to secure funding for improving healthcare provision. Consider consensus best‐practice standards, including standardized definitions and criteria for cancer supportive care management. Invest time and resources in training a competent care workforce and supporting volunteers for the health of cancer patients, as this is a facilitator in addressing workforce shortages

## 4 Discussion

This overview of reviews synthesized findings from 76 URs and focused on care delivery systems, supportive care needs, and supportive care interventions, systems, and models of care for cancer survivorship care and support. Overall, findings indicated that the number of eligible included reviews increased year‐by‐year from 2012 (*n* = 0), 2014 (*n* = 1) to 2023 (*n* = 31). The aims of the URs covered diverse topic areas. Evaluating current evidence on interventions was the most prevalent topic articulated within umbrella review aims. The most researched interventions included physical activity/exercise interventions (28%), and mind–body interventions (20%). Relatively fewer reviews addressed interventions such as psychological (9%), eHealth/mHealth (5%), and educational interventions (5%) which indicates that these areas may need more investigation. Unmet supportive care needs (*n* = 2) were the least focused topic covered by umbrella review aims.

The review published by O'Connor et al. [21] stated that despite some patients having curative cancer evidence demonstrates that some patients experience substantial morbidity after treatment. This morbidity is linked with consistent unmet needs related to supportive care [21]. More research is needed to focus on unmet supportive care needs in cancer. Additionally research involving patients and their families in low‐and middle‐income countries, underrepresented populations and rare cancers should be ramped up [22, 23].

This review highlights the importance of methodological rigor in conducting research with a view to high quality research, better informing future guidelines and healthcare and thus being better value for money [24]. Processes which provide a systematic approach to rating the quality and subsequently the certainty of evidence within evidence syntheses (such as Grading of Recommendations Assessment, Development and Evaluation [GRADE]) provides higher quality evidence to support clinical practice and policy development.

Based on our findings, a sizable number of reviews (*n* = 17) have not reported the methodological framework used to support their umbrella review. Recommendations (Table 4) suggest that to aid the development of evidence‐informed policy and best practice guidelines, future reviews should adhere to methodological and reporting standards. Another recommendation indicates that reviews and trials should be registered with a recognized platform to prevent publication bias.

This umbrella review highlights that URs are the highest level of evidence summary in healthcare. Gathering data from multiple studies enhances statistical power, research efficiency, and the reliability of the conclusions. URs offer clinicians and decision‑makers a clear, comprehensive overview of evidence which helps support decision making [2, 25]. However, it should be noted that URs have several limitations. The most significant concern is the duplication of included primary studies, which is prevalent in numerous reviews. This duplication can potentially introduce bias when synthesizing the findings. Furthermore, the heterogeneity of the included study designs, interventions, outcomes, and populations has a consequential effect on the comparability and interpretability of the findings. Publication bias and selective reporting represent additional quality threats to the reliability of synthesized findings in URs. The incorporation of methodologically weak or moderately robust reviews can compromise the quality of conclusions drawn from URs. Inclusion of low to moderate quality reviews within URs with pooled estimates may result in exaggerated treatment or supportive care benefits,however, inclusion of all reviews, regardless of their quality is recommended to minimize this risk [26].

Limited included URs addressed adolescent and young adults (AYA) (*n* = 3), children (*n* = 1), and carers (*n* = 3). Children and AYAs create a distinct and vulnerable group of patients since the cellular or molecular dynamics of their cancer may not be as well understood. The minimal research efforts and trial enrolment directed toward AYA malignancies and the lack of AYA specific supportive care needs to be addressed. Both child and adolescent cancer survival rates are improving day by day, and the importance of developing survivorship care plans for the long‐term and lifelong adverse effects of cancer treatment is becoming more obvious [27, 28]. Although many URs cover populations of any age, there is a need to specifically focus on child, adolescent, and young adult patients. Furthermore, reviews encompassing lip, oral cavity and pharynx cancer, or head and neck cancer, are less extensive than those prioritizing more common cancers such as breast cancer. We found that a key evidence gap relates to fewer studies having addressed rarer cancers; for example, types of acute myeloid leukemia, soft tissue carcinoma, oral and nasal cavity cancers, and kidney cancer [28, 29]. Patients who experience rare cancers suffer from unique barriers more than those diagnosed with common cancers. Limited access to clinical trials, poorer availability of evidence‐based care and decision‐making guidance for treatments are some of the challenges confronted by patients with rarer cancer [30]. The review published by Hegarty et al. [31] stated that survivorship care plans should be customized to different cancer types according to their unique needs. Hence, all cancer types should be investigated to determine their specific survivorship care and unmet needs, rather than implementing a “one‐size‐fits‐all” approach [32]. A number of URs reflected recommendations that samples should be expanded to include not only different cancer types but also age ranges reflecting the lifespan, and varied socio‐demographic and underrepresented groups. The National Institutes of Health describes underrepresented population groups as those who face health inequalities due to factors such as sexual preferences, gender identity, economic and social challenges, physical conditions like disabilities, and race and ethnicity [30].

This overview of URs highlighted the lack of emphasis on reporting the stage of cancer survivorship care with many reviews not specifying the stage of cancer survivorship (*n* = 15). Eighteen URs addressed the post‐treatment phase (extended cancer survivorship), which is to be welcomed. Including a comprehensive survivorship care plan referencing a variety of lifestyle changes can positively impact survivors' welfare [32, 33]. Therefore, research relating to the survivorship care pathway should focus on all stages of the cancer journey reflecting key transitions and treatment stages comprising physical, psychological, and social dimensions addressing potential and actual long‐term chronic health issues and comorbidity for patients [34]. Evaluating the deficiencies in survivorship care is an important research field to ensure equity and timeliness of responses to patient needs.

Systematic reviews and meta‐analyses (SR/MAs) are considered practical solutions for supplying up‐to‐date evidence to healthcare professionals as they provide robust evidence. However, substantial diversities in diagnosis, interventions, outcomes, and measurement methodologies prohibit the calculation of a combined effect size, which is essential for meta‐analyses [35, 36].

This review of reviews addressed broad range of URs that including multiple survivorship care plans, populations, diagnosis, interventions, supportive care models, unmet needs, and symptom management strategies. Our review provides an understanding of existing literature gaps, future recommendations, common weaknesses of included reviews in the oncology healthcare context. However, this review demonstrated several limitations. Included reviews were limited to the English language. URs provide an overview of global evidence synthesized within systematic reviews; however, the presentation of findings would be enhanced by presenting the evidence in terms of the geographic regions covered. Interestingly this review has not included reference to the challenge of balancing human self‐reporting processes, human validation processes and automated tools and platforms that employ artificial intelligence (AI) approaches in the conduction of reviews and in terms of the timeliness of the review; fiscal and human resource related costs; the relevance, accuracy, reliability and quality of the ultimate evidence synthesis.

## 5 Conclusions

This comprehensive overview synthesized a broad range of URs in the cancer context, covering topics such as symptom management, unmet needs, provision of supportive care, intervention implementation, models of care, and systems of care. Additionally, extensive recommendations for future reviews and trials are provided to improve the quality of future research and clinical cancer care. Recommendations are made for future research to address the multidimensional nature of clinical implementation and associated methodological challenges, to consider specific target populations, and to specifically inform policy development. This review of URs facilitates understanding by providing a comprehensive summary of published URs in the last 10 years on oncological supportive care, systems, and models of care. Future research may benefit from considering the highlighted recommendations.

## Author Contributions

**İrem Koç:** conceptualization; investigation; funding acquisition; writing – original draft; methodology; visualization; writing – review and editing; formal analysis. **John Goodwin:** conceptualization; writing – review and editing; supervision; methodology; validation; formal analysis. **Josephine Hegarty:** conceptualization; writing – review and editing; supervision; validation; methodology; project administration; formal analysis; writing – original draft; resources.

## Funding

Erasmus+ Internship Grant Programme which is a European Union funding programme. This work has been completed as part of a European Cooperation in Science and Technology (COST Association) project titled: CA21152 Implementation Network Europe for Cancer Survivorship Care (INE‐CSC). The views expressed in this paper are those of the authors of the paper and not the EU Commission.

## Ethics Statement

Obtaining ethical approval was not required since this review analyzed existing data and did not involve human or non‐human (animal) participants, material, or data.

## Conflicts of Interest

The authors declare no conflicts of interest.

## Supporting information


**Table S1:** Data extraction outlining characteristics of included umbrella reviews.

**Table S2:** Quality assessment of included umbrella reviews using JBI quality assessment tool.

**Table S3:** Details of papers excluded at full text review stage.

**Table S4:** Recommendations extracted directly from included umbrella reviews.

**Data S1:** Supporting Information.

**Data S2:** Supporting Information.

## Data Availability

This published overview of umbrella reviews comprehensively presents all relevant data within the Supporting Information.
