# Supplementary material for: Umbrella Reviews Conducted in an Oncology Healthcare Context Focusing on Supportive Care, Systems, and Models of Care: A Review of Umbrella Reviews
Source: Cancer Med. 2026 Mar 25;15(4):e71708. doi: 10.1002/cam4.71708 (PMC13140849; doi:10.1002/cam4.71708)
Supplement: Supplementary file 1 — Table S1: Data extraction outlining characteristics of included umbrella reviews. [file CAM4-15-e71708-s002.docx]

SUPPLEMENTARY INFORMATION 1

Includes details of the characteristics of included umbrella reviews and the corresponding reference list

TABLE S1 Data extraction outlining characteristics of included umbrella reviews

| **Author, year** | **Aim of umbrella review** | **Topics covered by aim of umbrella review** | **Number of included systematic reviews** | **Types of included systematic reviews** | **Details of synthesis completed** | **Quality assessment tool(s)** | **Population** | **Cancer diagnosis** | **Survivorship stage** |
| --- | --- | --- | --- | --- | --- | --- | --- | --- | --- |
| Abu-Odah, 2020^[1](#r1)^ | To synthesize evidence from previous reviews and deliver a more comprehensive mapping of the existing literature about personal, system, policy, and organizational challenges and possible facilitators on the provision of PC services for cancer patients in LMICs. | Provision of information to particular needs | 14 | Systematic reviews | NR | AMSTAR-2 | Adult patients | Mixed cancer sample (cancers not specified) | Focus on palliative care |
| Adam, 2015^[2](#r2)^ | To determine whether educational interventions can improve cancer pain management and to characterize components of cancer pain educational interventions. | Symptom management  Focus on optimizing experiences, of or meeting needs of caregivers/family members  Summarizing current evidence on educational interventions | 8 | Systematic reviews and SRs focused only including trials | Narrative synthesis | PRISMA checklist | Adult patients,  Caregivers,  Family members | Mixed cancer sample (cancers not specified) | NR |
| Amatya,2021^[3](#r3)^ | To evaluate existing evidence from published systematic reviews for the effectiveness of rehabilitation interventions in patients with lymphoma. | Summarizing current evidence on rehabilitation interventions | 12 | Systematic reviews and meta-analysis | NR | AMSTAR-2    GRADE approach (Cochrane) | Adult patients | Single cancer group  (lymphoma) | NR |
| Bao, 2014^[4](#r4)^ | To summarize available evidence, appraise the evidence level, and give suggestions to future research and practice on complementary and alternative medicine on adult cancer pain. | Symptom management  Summarizing current evidence on complementary and alternative medicine interventions | 27 | Systematic reviews and meta-analysis | NR | GRADE approach (Cochrane)  R-AMSTAR | Adult patients | Mixed cancer sample (cancers not specified) | NR |
| Belloni a, 2021^[5](#r5)^ | To identify which cancer population can mostly benefit from physical exercise interventions for reducing cancer related fatigue. | Provision of supportive care/manage particular needs/symptoms  Summarizing current evidence on physical exercise interventions | 10 | Systematic reviews and meta-analysis + SRs focused only including trials | Quantitative synthesis | ROBIS tool | Adult patients | Mixed cancer sample (cancers specified)  (Breast, male genital organs, digestive organs) | Acute survivorship  Adjuvant treatments |
| Belloni b, 2023^[6](#r6)^ | To synthesize the existing SRs and meta-analysis on nonpharmacologic interventions for reducing CRF in adult cancer patients. | Symptom management  Summarizing current evidence on non-pharmacological interventions | 28 | Systematic reviews and meta-analysis + SRs focused only including trials | Quantitative synthesis | ROBIS tool | Adult patients | Mixed cancer sample (cancers specified)  (Breast, male genital organs, digestive organs) | Acute survivorship  Adjuvant treatments  Extended cancer survivorship beyond 5 years after treatment |
| Belloni c, 2023^[7](#r7)^ | To systematically review the available evidence about physical exercise’s effects on improving adult cancer patients’ fatigue. | Symptom management  Summarizing current evidence on CAM interventions | 22 | Systematic reviews and meta-analysis + SRs focused only including trials | Quantitative synthesis | ROBIS tool | Adult patients | Mixed cancer sample (cancers not specified) | Adjuvant treatments  Extended cancer survivorship beyond 5 years after treatment |
| Belloni d, 2023^[8](#r8)^ | To describe relationships between psychosocial interventions and fatigue in adult patients with cancer, providing an overall estimate of their effect on CRF. | Symptom management  Summarizing current evidence on psychosocial interventions | 16 | Systematic reviews and meta-analysis + SRs focused only including trials | Quantitative synthesis | ROBIS tool | Adult patients | Mixed cancer sample (cancers not specified) | NR |
| Belloni e, 2021^[9](#r9)^ | To critically appraise the available systematic reviews on pharmacological intervention for improving CRF in adult cancer patients. | Symptom management  Summarizing current evidence on pharmacological interventions | 6 | Systematic reviews and meta-analysis | Quantitative synthesis | ROBIS tool | Adult patients | Mixed cancer sample (cancers not specified) | Acute survivorship  Adjuvant treatments  Extended cancer survivorship beyond 5 years after treatment |
| Bracchiglione, 2023^1^[^0^](#r10) | To evaluate and summarize the evidence of all relevant systematic reviews examining the benefits and harms of SOTs versus UPSC in advanced HBCs. | Overall experience of illness +/- treatment  Provision of supportive care/manage particular needs/symptoms | 18 | Systematic reviews and meta-analysis | Quantitative synthesis | AMSTAR-2    GRADE approach (Cochrane) | Adult patients | Mixed cancer sample (cancers specified)  Digestive organs  Hepatobiliary cancers (hepatocellular carcinoma, intrahepatic or extrahepatic cholangiocarcinoma and gallbladder cancer) | Acute survivorship  Metastatic cancer or advanced cancer |
| Butow, 2020^[11](#r11)^ | To summarize available evidence, appraise the evidence level, and give suggestions to future research and practice on CAM on adult cancer pain. | Provision of supportive care/manage particular needs/symptoms  Summarizing current evidence on complementary therapy interventions | 19 | Systematic reviews and meta-synthesis | Thematic qualitative synthesis | JBI critical appraisal checklist | Adult patients and AYAs | Mixed cancer sample (cancers specified)  (Breast, female genital organs, head and neck cancer, skin cancer, lymphoid cancer, digestive organs) | Adjuvant treatments  Extended cancer survivorship beyond 5 years after treatment |
| Casuso-Holgado, 2022^[12](#r12)^ | To develop an overview of systematic reviews to summarize the available evidence on the effectiveness of mind–body practices for cancer-related symptoms management. | Symptom management  Summarizing current evidence on mind-body interventions  Focus on optimizing experiences, of or meeting needs of caregivers/family members | 38 | Systematic reviews and meta-analysis | Narrative synthesis | AMSTAR-2    GRADE approach (Cochrane) | Adult patients | Mixed cancer sample (cancers specified)  (Breast, male genital organs, head and neck cancer, lung cancer, lymphoid cancer, digestive organs) | Mixed phases on survivorship journey (not specified) |
| Cedenilla Ramón, 2023^[13](#r13)^ | To provide evidence on the efficacy of psychosocial interventions for reducing cancer-related fatigue. | Symptom management  Summarizing current evidence on psychosocial and non-pharmacological interventions | 11 | Systematic reviews + SRs focused only including trials | NR | PRISMA checklist | Mixed age sample | Mixed cancer sample (cancers specified)  (Breast, male genital organs) | Acute survivorship  Adjuvant treatments  Extended cancer survivorship beyond 5 years after treatment  Focus on end of life |
| Chan a, 2023^1^[^4^](#r14) | To provide an overview of the existing literature in patient navigation in cancer care and the status of cancer patient navigation models and to promote consistency in expectations across the international community by conceptualizing cancer patient navigation using existing evidence. | Models of care, pathways, coordination/organizational/referral/ system focus | 61 | Systematic reviews and meta-analysis  Systematic reviews and meta-synthesis | Narrative synthesis | JBI critical appraisal checklist | Carers  Healthcare professionals | Mixed cancer sample (cancers specified)  (Breast, female and male genital organs, head and neck cancer, lung cancer, digestive organs, thyroid and other endocrine glands) | Focus on diagnosis  Acute survivorship  Transitions in survivorship  Focus on palliative care  Focus on end of life |
| Chan b, 2021^[15](#r15)^ | To synthesize the state of evidence on the efficacy and implementation of ‘tele-survivorship’ telemedicine in the post-treatment cancer survivorship phase. | Models of care, pathways, coordination/organizational/referral/ system focus  Summarizing current evidence on telemedicine interventions | 29 | Systematic reviews | Narrative synthesis | AMSTAR-2 | Mixed age sample | Mixed cancer sample (cancers specified)  (Breast, female and male genital organs, head and neck cancer, skin cancer, nervous system, lung cancer, digestive organs, thyroid and other endocrine glands) | Transitions in survivorship  Adjuvant treatments  Extended cancer survivorship beyond 5 years after treatment  Metastatic cancer or advanced cancer  Focus on palliative care |
| Chan c, 2023^[16](#r16)^ | To critically assess the effectiveness and implementation of different models of post-treatment cancer survivorship care compared to specialist-led models of survivorship care assessed in published systematic reviews. | Models of care, pathways, coordination/organizational/referral/ system focus | 12 | Systematic reviews and meta-analysis | Narrative synthesis | AMSTAR-2 | Adult patients  Healthcare professionals | Mixed cancer sample (cancers specified)  (Breast and lung cancer) | Transitions in survivorship  Adjuvant treatments  Extended cancer survivorship beyond 5 years after treatment  Metastatic cancer or advanced cancer  Focus on palliative care |
| Choi, 2022^[17](#r17)^ | To comprehensively summarize and critically evaluate the current evidence from SRs to determine the efficacy of AT (although acupuncture) the management of CRF. | Symptom management  Summarizing current evidence on acupuncture and moxibustion interventions | 15 | Systematic reviews and meta-analysis + SRs focused only including trials | Narrative synthesis  Qualitative synthesis | AMSTAR-2    GRADE approach (Cochrane) | Mixed age sample | Mixed cancer sample (cancers specified) | Mixed phases on survivorship journey (not specified) |
| Chung, 2015^[18](#r18)^ | To summarize evidence on Chinese herbal medicine for cancer palliative care. | Summarizing current evidence on Chinese herbal medicine interventions | 51 | Systematic reviews and meta-analysis | Quantitative synthesis | AMSTAR  CHIMERAS | Mixed age sample | Mixed cancer sample (cancers specified)  (Female genital organs, digestive organs, lung, respiratory organs) | Adjuvant treatments  Focus on palliative care |
| Conway, 2015^[19](#r19)^ | To appraise and synthesize evidence from only high-quality systematic reviews focused on the prevention, detection or management of cancer treatment-induced cardiotoxicity. | Provision of supportive care/manage particular needs/symptoms | 18 | Systematic reviews and meta-analysis | Narrative synthesis | AMSTAR | Mixed age sample | Mixed cancer sample (cancers specified)  (Breast, female genital organs, male genital organs, lymphoid cancer) | Acute survivorship |
| Crawford‐Williams, 2018^[20](#r20)^ | To systematically review the evidence for interventions addressing key domains of the American Cancer Society and American Society of Clinical Oncology Prostate Cancer Survivorship Care Guidelines: health promotion, surveillance, physical side effects, psychosocial management, and care coordination. | Provision of supportive care/manage particular needs/symptoms  Summarizing current evidence on interventions for prostate cancer | 44 | Systematic reviews and meta-analysis + SRs focused only including trials | Narrative synthesis | DARE | Adult patients | Single cancer group  (Prostate cancer) | Mixed phases on survivorship journey (not specified) |
| Duncan, 2017^[21](#r21)^ | To gather the evidence for practitioners, patients and their carers about effective non-pharmacological interventions to improve QoL in cancer survivors. | Summarizing current evidence on non-pharmacological interventions  Quality of life improvement initiatives  Focus on optimizing experiences, of or meeting needs of caregivers/family members | 21 | Systematic reviews + SRs focused only including trials | Narrative synthesis | AMSTAR | Adult patients | Mixed cancer sample (cancers specified)  (Breast, female and male genital organs, lung, digestive organs, head and neck, urinary tract, lymphoid cancer) | Acute survivorship  Adjuvant treatments  Extended cancer survivorship beyond 5 years after treatment |
| Edbrooke, 2023^[22](#r22)^ | To summarize exercise intervention efficacy and safety across the care continuum. | Symptom management  Models of care, pathways, coordination/organizational/referral/ system focus  Summarizing current evidence on exercise interventions  Quality of life improvement initiatives | 30 | Systematic reviews and meta-analysis + SRs focused only including trials | Narrative synthesis | AMSTAR-2    GRADE approach (Cochrane) | Adult patients | Single cancer group  (Lung cancer) | Mixed phases on survivorship journey (not specified) |
| Fournier, 2023^[23](#r23)^ | To identify existing digital interventions developed to provide supportive care to patients with cancer and their relatives in the cancer care continuum. | Summarizing current evidence on web-based or app-based digital health interventions  Focus on optimizing experiences, of or meeting needs of caregivers/family members | 20 | Systematic reviews and meta-analysis | Narrative synthesis | AMSTAR-2 | Mixed age sample  Family members | Mixed cancer sample (cancers not specified) | Mixed phases on survivorship journey (not specified) |
| Gkantaifi, 2020^[24](#r24)^ | To better clarify the preventive/therapeutic role of honey in the management of OM in patients with H-N cancer undergoing RT with or without chemotherapy. | Provision of supportive care/manage particular needs/symptoms  Prevention of adverse effects  Summarizing current evidence on honey interventions | 12 | Systematic reviews and meta-analysis | NR | NR | Mixed age sample | Single cancer group  (Head and neck cancer) | Acute survivorship |
| Grimmett, 2022^[25](#r25)^ | To provide a narrative synthesis of the impact of prehabilitation interventions on psychological outcomes in individuals awaiting cancer surgery. | Unmet needs/ unmet supportive care needs  Summarizing current evidence on psychological prehabilitation interventions | 20 | Systematic reviews and meta-analysis | Narrative synthesis | NR | Mixed age sample | Mixed cancer sample (cancers specified)  (Lung, and digestive organs) | Mixed phases on survivorship journey (not specified) |
| Hall, 2022^[26](#r26)^ | To synthesize the evidence for strategies to self-manage the side-effects of adjuvant endocrine therapy. | Provision of supportive care/manage particular needs/symptoms  Summarizing current evidence on interventions for improving self-manage side-effects of adjuvant endocrine therapy | 51 | Systematic reviews and meta-analysis  Clinical guidelines | Narrative synthesis | AMSTAR-2  AGREE-2 checklist | Mixed age sample | Single cancer group  (Breast cancer) | Mixed phases on survivorship journey (not specified) |
| Hou, 2023^[27](#r27)^ | To discuss the effects of physical activity on cancer-related fatigue in lung cancer patients. | Symptom management  Summarizing current evidence on exercise interventions | 13 | Systematic reviews and meta-analysis | Narrative synthesis | AMSTAR-2    GRADE approach (Cochrane)  CERQual checklist | Mixed age sample | Single cancer group  (Lung cancer) | NR |
| Huang, 2021^[28](#r28)^ | To identify and summarize the existing evidence of the effectiveness of tai chi in patients with breast cancer using a systematic overview. | Summarizing current evidence on tai chi interventions | 6 | Systematic reviews and meta-analysis + SRs focused only including trials | Quantitative synthesis | AMSTAR-2    GRADE approach (Cochrane)  PRISMA checklist | Adult patients | Single cancer group  (Breast cancer) | Acute survivorship |
| Jiang, 2020^[29](#r29)^ | To evaluate evidence from published systematic reviews about the effectiveness of exercise interventions on fatigue management in breast cancer patients. | Symptom management  Summarizing current evidence on exercise interventions | 24 | Systematic reviews and meta-analysis | NR | AMSTAR-2    GRADE approach (Cochrane) | Adult patients | Single cancer group  (Breast cancer) | NR |
| Khosroshahi a, 2023^[30](#r30)^ | To evaluate the evidence from published SRMAs of RCT that examine the effects of probiotics on diarrhea caused by chemotherapy and radiotherapy. | Summarizing current evidence on probiotic supplementation interventions  Provision of supportive care/manage particular needs/symptoms | 13 | Systematic reviews and meta-analysis + SRs focused only including trials | Quantitative synthesis | AMSTAR-2    GRADE approach (Cochrane)  ROB (Cochrane tool)  ICEMAN | Adult patients | Mixed cancer sample (cancers specified)  Male and female genital organs, (Lung, digestive organs) | Acute survivorship |
| Khosroshahi b, 2023^[31](#r31)^ | To provide a comprehensive evaluation of nutritional interventions for patients with cancer with OM, as well as to assess the quality of this evidence. | Summarizing current evidence on nutritional interventions  Provision of supportive care/manage particular needs/symptoms | 26 | Systematic reviews and meta-analysis + SRs focused only including trials | Quantitative synthesis | AMSTAR-2    GRADE approach (Cochrane) | Mixed age sample | Mixed cancer sample (cancers not specified) | NR |
| Khosroshahi c, 2022^[32](#r32)^ | To provide insight into the effects of oral cryotherapy against oral mucositis in patients with cancer, as well as to assess the certainty of this evidence. | Symptom management  Summarizing current evidence on oral cryotherapy interventions | 10 | Systematic reviews and meta-analysis | Quantitative synthesis | AMSTAR-2    GRADE approach (Cochrane)  ROB (Cochrane tool) | Mixed age sample | Mixed cancer sample (cancers specified)  (Lymphoma and solid tumours) | NR |
| Kim, 2018^[33](#r33)^ | To assess the current evidence for the various therapeutic options available for Aromatase inhibitor-associated arthralgia. | Provision of supportive care/manage particular needs/symptoms  Summarizing current evidence on aromatase inhibitor-associated arthralgia | 6 | Systematic reviews + SRs focused only including trials | Quantitative synthesis | AMSTAR-2 | Mixed age sample | Single cancer group  (Breast cancer) | Acute survivorship |
| Knowles, 2022^[34](#r34)^ | To summarize and synthesize the evidence for the impact of physical activity on patient outcomes (physical and psychological), health service use and cancer outcomes. | Summarizing current evidence on physical activity intervention  Quality of life improvement initiatives | 15 | Systematic reviews and meta-analysis + SRs focused only including trials | NR | JBI critical appraisal checklist | Older than 65 years patients | Mixed cancer sample (cancers specified)  (Breast, male genital organs, lung, digestive organs, urinary tract cancers) | Focus on diagnosis  Acute survivorship  Adjuvant treatments  Extended cancer survivorship beyond 5 years after treatment |
| Laidsaar-Powell, 2019^[35](#r35)^ | To systematically identify and describe all previously conducted systematic reviews of qualitative cancer survivorship, to provide insight into areas of research saturation and paucity. | Models of care, pathways, coordination/organizational/referral/ system focus | 60 | Systematic reviews and meta-synthesis | Content analysis | JBI critical appraisal checklist | Adult patients | Mixed cancer sample (cancers specified)  (Breast,female and male genital organs, lung, digestive organs, head and neck, skin, urinary tract, lymphoid cancers) | Mixed phases on survivorship journey (not specified) |
| Lake, 2022^[36](#r36)^ | To assess effectiveness of lifestyle interventions for female breast cancer survivors on weight loss, BMI, body composition, (HRQoL), physical functioning, psychosocial measures, biomarkers. | Summarizing current evidence on weight loss interventions  Quality improvement initiatives | 17 | Systematic reviews and meta-analysis | Narrative synthesis | JBI critical appraisal checklist | Adult (female) patients | Single cancer group  (Breast cancer) | Acute survivorship  Adjuvant treatments  Extended cancer survivorship beyond 5 years after treatment |
| Lavasidis, 2022^[37](#r37)^ | To summarize the evidence and evaluate the validity and the robustness of the effects of any supportive intervention on outcomes related to childhood cancer. | Summarizing current evidence on supportive interventions | 34 | Systematic reviews and meta-analysis + SRs focused only including trials | Quantitative synthesis | AMSTAR-2 | Children and AYAs | Single cancer group  (Childhood cancers) | Mixed phases on survivorship journey (not specified) |
| Lee a, 2023^[38](#r38)^ | To systematically appraise evidence on whether cryotherapy can reduce the incidence and severity of OM. | Provision of supportive care/manage particular needs/symptoms  Summarizing current evidence on cryotherapy intervention | 5 | Systematic reviews and meta-analysis | Quantitative synthesis | The Revised Assessment of Multiple Systematic Reviews | Adult patients | Mixed cancer sample (cancers specified)  (Solid cancers and Hematologic malignancies) | Acute survivorship |
| Lee b, 2018^[39](#r39)^ | This article critically examines the systematic reviews and meta-analysis of complementary therapies for cancer patients to appraise the evidence level, and offers suggestions for future research and practice. | Summarizing current evidence on complementary therapies | 104 | Systematic reviews and meta-analysis | NR | AMSTAR-2 | Mixed age sample | Mixed cancer sample (cancers specified)  (Breast, male genital organs, lung, digestive organs, head and neck, lymphoid cancers) | Acute survivorship  Adjuvant treatments  Extended cancer survivorship beyond 5 years after treatment |
| Leslie, 2022^[40](#r40)^ | To synthesize evidence on recruitment challenges and enablers, factors that promote engagement and adherence to web-based intervention content, and factors that promote the efficacy of web-based psychosocial interventions for patients with cancer and cancer survivors. | Summarizing current evidence on web-based psychosocial interventions | 20 | Systematic reviews and meta-analysis  Systematic reviews and meta-synthesis | Narrative and thematic synthesis | PRISMA checklist | Adult patients and AYAs  (>16 years) | Mixed cancer sample (cancers not specified) | Mixed phases on survivorship journey (not specified) |
| Licqurish, 2019^[41](#r41)^ | To examine the evidence from systematic reviews of tools intended to facilitate communication during consultations between physicians and people diagnosed with cancer. | Provision of supportive care/manage particular needs/symptoms  Summarizing current evidence on communication tools-facilitate communication during consultations  Uni-disciplinary focus (physicians) | 11 | Systematic reviews | Narrative synthesis | AMSTAR-2 | Adult patients  Healthcare professionals | Mixed cancer sample (cancers not specified) | Mixed phases on survivorship journey (not specified) |
| Li, 2023^[42](#r42)^ | To assess the quality of previous SRs and assimilate the existing evidence base to promote determining effective evidence-based interventions for CRF in adults and help clinicians to interpret the evidence. | Symptom management  Summarizing current evidence on complementary and alternative medicine interventions | 30 | Systematic reviews and meta-analysis + SRs focused only including trials | Quantitative synthesis | AMSTAR-2  GRADE approach (Cochrane)  ROBIS tool  PRISMA checklist | Adult patients | Mixed cancer sample (cancers specified)  (Breast, lung, lymphoid) | Mixed phases on survivorship journey (not specified) |
| Loh, 2015^[43](#r43)^ | To examine systematic reviews on the rehabilitation methods for post-operative women with breast cancer, with a view on the comprehensiveness of these methods used, and if they consider breast cancer as a chronic illness. | Provision of supportive care/manage particular needs/symptoms  Summarizing current evidence on physical and occupational therapy, exercise, psychosocial, alternative/complementary, nutritional, alternative interventions  Quality of life improvement initiatives | 7 | Systematic reviews + SRs focused only including trials | NR | AMSTAR | Adult patients | Single cancer group  (Breast cancer) | Adjuvant treatments  Extended cancer survivorship beyond 5 years after treatment |
| Mainou, 2023^[44](#r44)^ | To present all systematic reviews focusing on adverse events due to antimyeloma treatments. | Overall experience of illness +/- treatment | 23 | Systematic reviews + SRs focused only including trials | Quantitative synthesis | AMSTAR-2 | NR | Mixed cancer sample (cancers not specified | Mixed phases on survivorship journey (not specified) |
| Mazzocco, 2023^[45](#r45)^ | To provide a concise summary to facilitate an evidence-based decision on integrating Qigong into cancer patients’ care. | Summarizing current evidence on mind-body interventions | 19 | Systematic reviews and meta-analysis + SRs focused only including trials | NR | AMSTAR-2 | Mixed age sample | Mixed cancer sample (cancers specified)  (Breast, lung) | Mixed phases on survivorship journey (not specified) |
| Mentink, 2023^[46](#r46)^ | To provide a comprehensive overview of up-to-date evidence on the effects of complementary therapies on physical, psychological, and general patient-reported health outcomes in patients with cancer. | Summarizing current evidence on complementary therapy interventions | 100 | Systematic reviews and meta-analysis + SRs focused only including trials | Quantitative synthesis | AMSTAR-2 | Adult patients | Mixed cancer sample (cancers not specified | NR |
| Mokhtari-Hessari, 2020^[47](#r47)^ | To update the current knowledge on health-related quality of life in breast cancer patients. | Evaluating current evidence on interventions  Quality of life improvement initiatives | 82 | Systematic reviews and meta-analysis | Findings presented chronologically | AMSTAR | Mixed age sample | Single cancer group  (Breast cancer) | Focus on diagnosis  Acute survivorship  Adjuvant treatments  Extended cancer survivorship beyond 5 years after treatment |
| Olsson Möller, 2019^[48](#r48)^ | To evaluate the current evidence on rehabilitation interventions in female patients following breast cancer treatment. | Summarizing current evidence on rehabilitation intervention | 37 | Systematic reviews + SRs focused only including trials | NR | AMSTAR-2 | Adult patients | Single cancer group  (Breast cancer) | Acute survivorship |
| Pedro, 2021^[49](#r49)^ | To investigate the effect of structured mindfulness‐based interventions on psychological outcomes (such as anxiety, depression, and stress), QoL, as well as biological outcomes (e.g., inflammatory response), focusing on patients with cancer and cancer survivors. | Summarizing current evidence on mindfulness‐based interventions | 10 | Systematic reviews and meta-analysis + SRs focused only including trials | Narrative synthesis | AMSTAR | Adult patients | Mixed cancer sample (cancers specified)  (Breast, male genital organs, lung, digestive organs cancers) | Focus on diagnosis  Acute survivorship  Adjuvant treatments  Extended cancer survivorship beyond 5 years after treatment |
| Petrigna, 2023^[50](#r50)^ | To analyze the protocols of different physical activity interventions and to eventually propose a standard operating procedure for possible exercise training in breast cancer patients | Provision of supportive care/manage particular needs/ symptoms  Summarizing current evidence on physical activity interventions | 30 | Systematic reviews and meta-analysis + SRs focused only including trials | Narrative synthesis | AMSTAR  Seventeen studies adopted Cochrane Handbook to detect the quality of the included studies  PEDro scale | Adult patients | Single cancer group  (Breast cancer) | Mixed phases on survivorship journey (not specified) |
| Qiu, 2023^[51](#r51)^ | To systematically summarize the interventions to promote physical activity behavior change and increase physical activity among cancer patients. | Summarizing current evidence on any interventions to promote physical activity behavior | 26 | Systematic reviews and meta-analysis + SRs focused only including trials  Systematic reviews and meta-synthesis | Narrative synthesis | AMSTAR-2  JBI critical appraisal checklist | Mixed age sample | Mixed cancer sample (cancers specified)  (Breast, digestive organs, childhood cancers) | Acute survivorship  Transitions in survivorship  Adjuvant treatments  Extended cancer survivorship beyond 5 years after treatment |
| Rafn, 2023^[52](#r52)^ | To summarize the evidence for effects of different treatment modalities for lymphedema in breast cancer survivors. | Summarizing current evidence on surgical, pharmacological, exercise, CDP, laser therapy, kinesio taping, and acupuncture interventions | 18 | Systematic reviews and meta-analysis + SRs focused only including trials | Quantitative synthesis | AMSTAR-2 | Adult patients | Single cancer group  (Breast cancer) | NR |
| Rapti, 2023^[53](#r53)^ | To examine the effects of exercise/physical activity on fatigue, HRQOL, pain, cardiovascular/cardiorespiratory fitness, and physical function on pediatric cancer patients. | Summarizing current evidence on exercise and physical activity interventions | 13 | Systematic reviews and meta-analysis | Narrative synthesis | AMSTAR-2 | Children and adolescents  <19 years (or older participants with cancer if the initiation of disease was before they reach 19 years) | Mixed cancer sample (cancers specified)  (Lymphoid, hematopoietic, childhood cancers) | Acute survivorship  Transitions in survivorship  Adjuvant treatments  Extended cancer survivorship beyond 5 years after treatment |
| Riccetti, 2021^[54](#r54)^ | To provide a comprehensive overview on the needs of migrants and ethnic minority cancer patients. | Unmet needs/unmet supportive care needs | 5 | Systematic reviews | NR | JBI critical appraisal checklist | Migrants, and ethnic minorities | Mixed cancer sample (cancers not specified) | Mixed phases on survivorship journey (not specified) |
| King, 2023^[55](#r55)^ | To provide the first truly comprehensive and exhaustive summary of qualitative evidence focusing on survivorship experiences of women with BC, across both early and advanced stage disease. | Provision of supportive care/manage particular needs/symptoms  Models of care, pathways, coordination/organizational/referral/ system focus | 25 | Systematic reviews | Qualitative synthesis | JBI critical appraisal checklist  Qualitative Critical Appraisal Skills Programme (CASP) | Adult patients | Single cancer group  (Breast cancer) | Focus on diagnosis  Acute survivorship  Transitions in survivorship  Adjuvant treatments  Extended cancer survivorship beyond 5 years after treatment  Metastatic or advanced cancer |
| Schroter, 2023^[56](#r56)^ | To summarize and critically assess the available evidence of treatment methods for ORN in adults with head and neck cancer from SRs yielding more high-quality evidence for clinical practice. | Summarizing current evidence on osteoradionecrosis treatment interventions | 6 | Systematic reviews and meta-analysis + SRs focused only including trials | NR | AMSTAR-2  GRADE approach (Cochrane) | Adult patients | Single cancer group  (Head and neck cancer) | NR |
| Shi, 2023^[57](#r57)^ | To summarize and evaluate the evidence from current systematic reviews/meta-analysis on the effectiveness of acupuncture treatment for CIPN. | Summarizing current evidence on acupuncture interventions | 9 | Systematic reviews and meta-analysis + SRs focused only including trials | Quantitative synthesis | AMSTAR-2  GRADE approach (Cochrane)  PRISMA checklist | Adult patients | Mixed cancer sample (cancers not specified) | NR |
| Slev, 2016^[58](#r58)^ | To synthesize evidence from systematic reviews on the effects of eHealth for cancer patients or their informal caregivers. | Summarizing current evidence on eHealth interventions  Quality improvement initiatives  Focus on optimizing experiences of or meeting needs of carers/caregivers or family members | 10 | Systematic reviews | Qualitative synthesis | OQAQ | Adult patients  Carers | Mixed cancer sample (cancers not specified) | Mixed phases on survivorship journey (not specified) |
| Specchia, 2020^[59](#r59)^ | To perform an umbrella review summarizing the available evidence on the impact of tumor boards on healthcare outcomes and processes. | Models of care, pathways, coordination/organizational/referral/ system focus | 5 | Systematic reviews | NR | AMSTAR-2 | Mixed age sample | Mixed cancer sample (cancers specified)  (Breast, female and male genital organs, lung, head and neck, urinary tract, skin, central nervous system, lymphoid cancers) | NR |
| Sun, 2023^[60](#r60)^ | To summarize evidence from SRMAs regarding the impact of dyadic interventions delivered to both members of a cancer dyad, including a cancer patient and caregiver. | Summarizing current evidence on dyadic interventions | 18 | Systematic reviews and meta-analysis + SRs focused only including trials | Narrative synthesis | AMSTAR-2  GRADE approach (Cochrane) | Mixed age sample  Carers  Family members | Mixed cancer sample (cancers not specified) | Mixed phases on survivorship journey (not specified) |
| Tuominen, 2018^[61](#r61)^ | To summarize the results of reviews assessing the effectiveness of nursing interventions among patients with cancer. | Models of care, pathways, coordination/organizational/referral/ system focus  Focus on optimizing experiences of or meeting needs of carers/caregivers or family members  Summarizing current evidence on nursing interventions | 9 | Systematic reviews and meta-analysis | NR | AMSTAR | Adult patients | Mixed cancer sample (cancers specified)  (female genital organs, lung cancer) | NR |
| Trigueros-Murillo, 2023^[62](#r62)^ | To summarize the available evidence from systematic reviews with meta-analysis on the effects of music-based interventions in adults diagnosed with cancer. | Symptom management  Summarizing current evidence on music-based interventions | 13 | Systematic reviews and meta-analysis | Narrative synthesis | AMSTAR-2 | Adult patients | Mixed cancer sample (cancers specified)  (Breast, male genital organs, lung, head and neck, urinary tract, skin, lymphoid cancers) | NR |
| Tune, 2022^[63](#r63)^ | To identify how quality of mHealth interventions for cancer survivors was described. | Summarizing current evidence on mHealth interventions | 7 | Systematic reviews | Qualitative synthesis | AMSTAR | Adult patients | Mixed cancer sample (cancers specified)  (Breast, male genital organs, digestive, lung, head and neck, lymphoid cancers) | NR |
| Vieira Nascimento, 2023^[64](#r64)^ | To systematically summarize the evidence for photobiomodulation therapy in the prevention and treatment of OM in patients undergoing cancer treatment. | Symptom management  Summarizing current evidence on photobiomodulation therapy interventions | 16 | Systematic reviews + SRs focused only including trials | Quantitative synthesis | AMSTAR-2  PRISMA checklist | Mixed age sample | Mixed cancer sample (cancers not specified) | NR |
| Wang a, 2022^[65](#r65)^ | To summarize the existing systematic reviews on the effectiveness of CM in health-related outcomes and health care utilization outcomes for cancer patient care, and highlight the consistent and contradictory findings. | Overall experience of illness +/- treatment  Summarizing current evidence on case management interventions | 8 | Systematic reviews and meta-analysis | Narrative synthesis | JBI critical appraisal checklist | Mixed age sample | Mixed cancer sample (cancers specified)  (Breast, male and female genital organs, digestive, lung, lymphoid cancers) | Focus on diagnosis  Acute survivorship  Transitions in survivorship  Adjuvant treatments  Extended cancer survivorship beyond 5 years after treatment  Metastatic or advanced cancer |
| Wang b, 2023^[66](#r66)^ | To provide an overview of the methodological quality, risk of bias, quality of reporting, and quality of evidence for SRs/MAs of acupuncture for BCRL. | Summarizing current evidence on acupuncture interventions | 8 | Systematic reviews and meta-analysis | NR | AMSTAR-2  GRADE approach (Cochrane)  ROBIS  PRISMA checklist | Adult patients | Single cancer group  (Breast cancer) | NR |
| Wu a, 2015^[67](#r67)^ | To summarise the evidence on acupuncture for palliative care of cancer. | Symptom management  Summarizing current evidence on acupuncture interventions  Quality of life improvement initiatives | 23 | Systematic reviews and meta-analysis + SRs focused only including trials | Narrative synthesis | AMSTAR | Mixed age sample | Mixed cancer sample (cancers specified)  (Breast, male genital organs, lung, head and neck cancers) | Acute survivorship  Adjuvant treatments  Metastatic or advanced cancer  Focus on palliative care |
| Wu b, 2016^[68](#r68)^ | To evaluate the comparative effectiveness of different CHM for improving QoL among NSCLC patients who are receiving chemotherapy. | Quality of life improvement initiatives  Summarizing current evidence on chinese herbal medicine interventions | 61 | Systematic reviews + SRs focused only including trials | Quantitative synthesis | AMSTAR | Mixed age sample | Single cancer group  (Lung cancer) | Acute survivorship  Adjuvant treatments |
| Xing, 2023^[69](#r69)^ | To synthesize findings on the clinical effectiveness of MLD in treating BCRL by critically assessing the quality of existing literature and attempting to resolve discordant outcomes. | Symptom management  Summarizing current evidence on manual lymphatic drainage and/or physiotherapy | 7 | Systematic reviews and meta-analysis + SRs focused only including trials | NR | AMSTAR-2  GRADE approach (Cochrane)  GRADE-CERQual | Mixed age sample | Single cancer group  (Breast cancer) | NR |
| Zanghi, 2022^[70](#r70)^ | To analyze the efficacy of different physical-activity interventions in the physical, mental, and social spheres of breast-cancer survivors. | Summarizing current evidence on physical-activity interventions | 12 | Systematic reviews and meta-analysis + SRs focused only including trials | Narrative synthesis | AMSTAR | Mixed age sample | Single cancer group  (Breast cancer) | Acute survivorship  Adjuvant treatments |
| Zhang a, 2022^[71](#r71)^ | To evaluate and summarize the SRs that assess the effects and safety of acupuncture for cancer-related conditions, and to inform clinical practice and future studies. | Symptom management  Summarizing current evidence on cancer related conditions | 51 | Systematic reviews and meta-analysis + SRs focused only including trials | Narrative synthesis | AMSTAR-2  ROBIS tool | Mixed age sample | Mixed cancer sample (cancers specified)  (Breast, female genital organs, digestive cancers) | Mixed phases on survivorship journey (not specified) |
| Zhang b, 2023^[72](#r72)^ | To summarize evidence regarding the efficacy of exercise interventions to reduce cancer fatigue, as determined in SRs and/or MAs. | Symptom management  Summarizing current evidence on exercise interventions | 46 | Systematic reviews and meta-analysis + SRs focused only including trials | Qualitative synthesis | AMSTAR-2 | Adult patients | Mixed cancer sample (cancers specified)  (Breast, male genital organs, digestive, lung, lymphoid cancers) | NR |
| Zhang c, 2020^[73](#r73)^ | To evaluate the quality of SR/MAs on alternative exercise traditions in cancer care. | Summarizing current evidence on alternative exercise traditions interventions | 26 | Systematic reviews and meta-analysis | NR | AMSTAR  PRISMA checklist | Mixed age sample | Mixed cancer sample (cancers not specified) | Mixed phases on survivorship journey (not specified) |
| Zhao, 2023^[74](#r74)^ | To examine current research evidence concerning the effectiveness of exercise on symptom management in breast cancer patients undergoing adjuvant treatment. | Symptom management  Summarizing current evidence on exercise interventions | 15 | Systematic reviews and meta-analysis | Narrative synthesis | AMSTAR-2 | Adult patients | Single cancer group  (Breast cancer) | Adjuvant treatments |
| Zhou a, 2022^[75](#r75)^ | To critically appraise and consolidate evidence from current SRs/MAs on the effects of exercise interventions on CRF in breast cancer patients. | Symptom management  Summarizing current evidence on exercise interventions | 29 | Systematic reviews and meta-analysis + SRs focused only including trials | Quantitative synthesis | AMSTAR-2  GRADE approach (Cochrane) | Adult patients | Single cancer group  (Breast cancer) | Mixed phases on survivorship journey (not specified) |
| Zhou b, 2020^[76](#r76)^ | To identify, appraise and summarise systematic reviews of exercise interventions for surgical lung cancer patients. | Summarizing current evidence on exercise interventions | 7 | Systematic reviews and meta-analysis + SRs focused only including trials | NR | AMSTAR-2  GRADE approach (Cochrane) | Mixed age sample | Single cancer group  (Lung cancer) | NR |

Abbreviations: AYAs, Adolescent and young adult patients; BC, Breast cancer; BCRL, Breast cancer-related lymphoedema; CAM, Complementary and alternative medicine; CERQual, Confidence in the Evidence from Reviews of Qualitative research; CHIMERAS, Chinese Integrative Medicine Evidence Rating System; CHM, Chinese herbal medicine; CM, Case management; CRF, Cancer-related fatigue; DARE, Database of Abstracts of Reviews of Effects; HBCs, Hepatobiliary cancers; H-N, Head and neck cancer; HRQoL, Health-related quality of life; ICEMAN, Development of the Instrument to assess the Credibility of Effect Modification Analyses; LMICs, Low- and middle-income countries; MLD, Manual Lymphatic Drainage; NR, Not reported; NSCLC, Nonsmall Cell Lung Cancer; OM, Oral mucositis; OQAQ, Overview Quality Assessment Questionnaire; ORN, Osteoradionecrosis treatment; PC, palliative care; QoL, Quality of life; RCT, Randomized clinical trials; SOTs, Systemic oncological treatments including chemotherapy, immunotherapy and targeted/biological therapies; SRs, Systematic reviews; SR/Mas, Systematic reviews and meta-analysis; UPSC, Usual practice supportive care.

**REFERENCES (Listing of the 76 included Umbrella review papers)**

1. Abu-Odah H, Molassiotis A, Liu J. Challenges on the provision of palliative care for patients with cancer in low- and middle-income countries: a systematic review of reviews. *BMC Palliat Care*. 2020;19(1):55. doi:10.1186/s12904-020-00558-5

2. Adam R, Bond C, Murchie P. Educational interventions for cancer pain. A systematic review of systematic reviews with nested narrative review of randomized controlled trials. *Patient Educ Couns*. 2015;98(3):269-282. doi:10.1016/j.pec.2014.11.003

3. Amatya B, Khan F, Lew TE, Dickinson M. Rehabilitation in patients with lymphoma: An overview of Systematic Reviews. *J Rehabil Med*. 2021;53(3):jrm00163. doi:10.2340/16501977-2810

4. Bao Y, Kong X, Yang L, et al. Complementary and Alternative Medicine for Cancer Pain: An Overview of Systematic Reviews. *Evid Based Complement Alternat Med*. 2014;2014:170396. doi:10.1155/2014/170396

5. Belloni S, Arrigoni C, Caruso R. Effects from physical exercise on reduced cancer-related fatigue: a systematic review of systematic reviews and meta-analysis. *Acta Oncol*. 2021;60(12):1678-1687. doi:10.1080/0284186X.2021.1962543

6. Belloni S, Arrigoni C, Baroni I, et al. Non-pharmacologic interventions for improving cancer-related fatigue (CRF): A systematic review of systematic reviews and pooled meta-analysis. *Semin Oncol*. 2023;50(1-2):49-59. doi:10.1053/j.seminoncol.2023.03.004

7. Belloni S, Bonucci M, Arrigoni C, Dellafiore F, Caruso R. A Systematic Review of Systematic Reviews and a Pooled Meta-Analysis on Complementary and Integrative Medicine for Improving Cancer-Related Fatigue. *Clin Ther*. 2023;45(1):e54-e73. doi:10.1016/j.clinthera.2022.12.001

8. Belloni S, Arrigoni C, Arcidiacono MA, et al. A Systematic Review of Systematic Reviews and Pooled Meta-Analysis on Psychosocial Interventions for Improving Cancer-Related Fatigue. *Semin Oncol Nurs*. 2023;39(3):151354. doi:10.1016/j.soncn.2022.151354

9. Belloni S, Arrigoni C, de Sanctis R, Arcidiacono MA, Dellafiore F, Caruso R. A systematic review of systematic reviews and pooled meta-analysis on pharmacological interventions to improve cancer-related fatigue. *Critical Reviews in Oncology/Hematology*. 2021;166:103373. doi:10.1016/j.critrevonc.2021.103373

10. Bracchiglione J, Rodríguez-Grijalva G, Requeijo C, et al. Systemic Oncological Treatments versus Supportive Care for Patients with Advanced Hepatobiliary Cancers: An Overview of Systematic Reviews. *Cancers (Basel)*. 2023;15(3):766. doi:10.3390/cancers15030766

11. Butow P, Laidsaar-Powell R, Konings S, Lim CYS, Koczwara B. Return to work after a cancer diagnosis: a meta-review of reviews and a meta-synthesis of recent qualitative studies. *J Cancer Surviv*. 2020;14(2):114-134. doi:10.1007/s11764-019-00828-z

12. Casuso-Holgado MJ, Heredia-Rizo AM, Gonzalez-Garcia P, Muñoz-Fernández MJ, Martinez-Calderon J. Mind-body practices for cancer-related symptoms management: an overview of systematic reviews including one hundred twenty-nine meta-analyses. *Support Care Cancer*. 2022;30(12):10335-10357. doi:10.1007/s00520-022-07426-3

13. Cedenilla Ramón N, Calvo Arenillas JI, Aranda Valero S, Sánchez Guzmán A, Moruno Miralles P. Psychosocial Interventions for the Treatment of Cancer-Related Fatigue: An Umbrella Review. *Curr Oncol*. 2023;30(3):2954-2977. doi:10.3390/curroncol30030226

14. Chan RJ, Milch VE, Crawford-Williams F, et al. Patient navigation across the cancer care continuum: An overview of systematic reviews and emerging literature. *CA Cancer J Clin*. 2023;73(6):565-589. doi:10.3322/caac.21788

15. Chan RJ, Crichton M, Crawford-Williams F, et al. The efficacy, challenges, and facilitators of telemedicine in post-treatment cancer survivorship care: an overview of systematic reviews. *Ann Oncol*. 2021;32(12):1552-1570. doi:10.1016/j.annonc.2021.09.001

16. Chan RJ, Crawford-Williams F, Crichton M, et al. Effectiveness and implementation of models of cancer survivorship care: an overview of systematic reviews. *J Cancer Surviv*. 2023;17(1):197-221. doi:10.1007/s11764-021-01128-1

17. Choi TY, Ang L, Jun JH, Alraek T, Lee MS. Acupuncture and Moxibustion for Cancer-Related Fatigue: An Overview of Systematic Reviews and Meta-Analysis. *Cancers (Basel)*. 2022;14(10):2347. doi:10.3390/cancers14102347

18. Chung VCH, Wu X, Hui EP, et al. Effectiveness of Chinese herbal medicine for cancer palliative care: overview of systematic reviews with meta-analyses. *Sci Rep*. 2015;5:18111. doi:10.1038/srep18111

19. Conway A, McCarthy AL, Lawrence P, Clark RA. The prevention, detection and management of cancer treatment-induced cardiotoxicity: a meta-review. *BMC Cancer*. 2015;15:366. doi:10.1186/s12885-015-1407-6

20. Crawford-Williams F, March S, Goodwin BC, et al. Interventions for prostate cancer survivorship: A systematic review of reviews. *Psychooncology*. 2018;27(10):2339-2348. doi:10.1002/pon.4888

21. Duncan M, Moschopoulou E, Herrington E, et al. Review of systematic reviews of non-pharmacological interventions to improve quality of life in cancer survivors. *BMJ Open*. 2017;7(11):e015860. doi:10.1136/bmjopen-2017-015860

22. Edbrooke L, Bowman A, Granger CL, et al. Exercise across the Lung Cancer Care Continuum: An Overview of Systematic Reviews. *Journal of Clinical Medicine*. 2023;12(5):1871. doi:10.3390/jcm12051871

23. Fournier V, Duprez C, Grynberg D, Antoine P, Lamore K. Are digital health interventions valuable to support patients with cancer and caregivers? An umbrella review of web-based and app-based supportive care interventions. *Cancer Med*. 2023;12(23):21436-21451. doi:10.1002/cam4.6695

24. Gkantaifi A, Alongi F, Vardas E, et al. Honey Against Radiation-induced Oral Mucositis in Head and Neck Cancer Patients. An Umbrella Review of Systematic Reviews and Meta- Analyses of the Literature. *Rev Recent Clin Trials*. 2020;15(4):360-369. doi:10.2174/1574887115666200709140405

25. Grimmett C, Heneka N, Chambers S. Psychological Interventions Prior to Cancer Surgery: a Review of Reviews. *Curr Anesthesiol Rep*. 2022;12(1):78-87. doi:10.1007/s40140-021-00505-x

26. Hall LH, King NV, Graham CD, et al. Strategies to self-manage side-effects of adjuvant endocrine therapy among breast cancer survivors: an umbrella review of empirical evidence and clinical guidelines. *J Cancer Surviv*. 2022;16(6):1296-1338. doi:10.1007/s11764-021-01114-7

27. Hou W, Zhai L, Yang Y, et al. Is physical activity effective against cancer-related fatigue in lung cancer patients? An umbrella review of systematic reviews and meta-analyses. *Support Care Cancer*. 2023;31(3):161. doi:10.1007/s00520-023-07627-4

28. Huang J, Liu H, Chen J, Cai X, Huang Y. The Effectiveness of Tai Chi in Patients With Breast Cancer: An Overview of Systematic Reviews and Meta-Analyses. *Journal of Pain and Symptom Management*. 2021;61(5):1052-1059. doi:10.1016/j.jpainsymman.2020.10.007

29. Jiang M, Ma Y, Yun B, Wang Q, Huang C, Han L. Exercise for fatigue in breast cancer patients: An umbrella review of systematic reviews. *Int J Nurs Sci*. 2020;7(2):248-254. doi:10.1016/j.ijnss.2020.03.001

30. Amiri Khosroshahi R, Zeraattalab-Motlagh S, Sarsangi P, Nielsen SM, Mohammadi H. Effect of probiotic supplementation on chemotherapy- and radiotherapy-related diarrhoea in patients with cancer: an umbrella review of systematic reviews and meta-analyses. *Br J Nutr*. 2023;130(10):1754-1765. doi:10.1017/S0007114523000910

31. Amiri Khosroshahi R, Talebi S, Zeraattalab-Motlagh S, et al. Nutritional interventions for the prevention and treatment of cancer therapy-induced oral mucositis: an umbrella review of systematic reviews and meta-analysis. *Nutrition Reviews*. 2023;81(9):1200-1212. doi:10.1093/nutrit/nuac105

32. Khosroshahi RA, Talebi S, Travica N, Mohammadi H. Cryotherapy for oral mucositis in cancer: review of systematic reviews and meta-analysis. *BMJ Supportive & Palliative Care*. 2023;13(e3):e570-e577. doi:10.1136/spcare-2022-003636

33. Kim TH, Kang JW, Lee TH. Therapeutic options for aromatase inhibitor-associated arthralgia in breast cancer survivors: A systematic review of systematic reviews, evidence mapping, and network meta-analysis. *Maturitas*. 2018;118:29-37. doi:10.1016/j.maturitas.2018.09.005

34. Knowles R, Kemp E, Miller M, Davison K, Koczwara B. Physical activity interventions in older people with cancer: A review of systematic reviews. *Eur J Cancer Care (Engl)*. 2022;31(5):e13637. doi:10.1111/ecc.13637

35. Laidsaar-Powell R, Konings S, Rankin N, et al. A meta-review of qualitative research on adult cancer survivors: current strengths and evidence gaps. J Cancer Surviv. 2019;13(6):852-889. doi:10.1007/s11764-019-00803-8

36. Lake B, Damery S, Jolly K. Effectiveness of weight loss interventions in breast cancer survivors: a systematic review of reviews. *BMJ Open*. 2022;12(10):e062288. doi:10.1136/bmjopen-2022-062288

37. Lavasidis G, Markozannes G, Voorhies K, et al. Supportive interventions for childhood cancer: An umbrella review of randomized evidence. *Crit Rev Oncol Hematol*. 2022;180:103845. doi:10.1016/j.critrevonc.2022.103845

38. Lee CC, Kuo SF, Chang WP, Guo SL, Huang TW. Effectiveness of Cryotherapy on Cancer Therapy-Induced Oral Mucositis: An Umbrella Review. *Cancer Nurs*. 2023;46(5):E288-E296. doi:10.1097/NCC.0000000000001128

39. Lee SM, Choi HC, Hyun MK. An Overview of Systematic Reviews: Complementary Therapies for Cancer Patients. *Integr Cancer Ther*. 2019;18:1534735419890029. doi:10.1177/1534735419890029

40. Leslie M, Beatty L, Hulbert-Williams L, et al. Web-Based Psychological Interventions for People Living With and Beyond Cancer: Meta-Review of What Works and What Does Not for Maximizing Recruitment, Engagement, and Efficacy. *JMIR Cancer*. 2022;8(3):e36255. doi:10.2196/36255

41. Licqurish SM, Cook OY, Pattuwage LP, et al. Tools to facilitate communication during physician-patient consultations in cancer care: An overview of systematic reviews. *CA Cancer J Clin*. 2019;69(6):497-520. doi:10.3322/caac.21573

42. Li P, Wang Q, Liu L, et al. The Role of Complementary and Alternative Medicine on Cancer-Related Fatigue in Adults: An Overview of Systematic Reviews. *Integr Cancer Ther*. 2023;22:15347354231188947. doi:10.1177/15347354231188947

43. Loh SY, Musa AN. Methods to improve rehabilitation of patients following breast cancer surgery: a review of systematic reviews. *Breast Cancer (Dove Med Press)*. 2015;7:81-98. doi:10.2147/BCTT.S47012

44. Mainou M, Bougioukas KI, Malandris K, et al. Reporting of adverse events of treatment interventions in multiple myeloma: an overview of systematic reviews. *Ann Hematol*. 2024;103(8):2681-2697. doi:10.1007/s00277-023-05517-7

45. Mazzocco K, Milani A, Ciccarelli C, Marzorati C, Pravettoni G. Evidence for Choosing Qigong as an Integrated Intervention in Cancer Care: An Umbrella Review. *Cancers (Basel)*. 2023;15(4):1176. doi:10.3390/cancers15041176

46. Mentink M, Verbeek D, Noordman J, Timmer-Bonte A, von Rosenstiel I, van Dulmen S. The Effects of Complementary Therapies on Patient-Reported Outcomes: An Overview of Recent Systematic Reviews in Oncology. *Cancers*. 2023;15(18):4513. doi:10.3390/cancers15184513

47. Mokhtari-Hessari P, Montazeri A. Health-related quality of life in breast cancer patients: review of reviews from 2008 to 2018. *Health Qual Life Outcomes*. 2020;18(1):338. doi:10.1186/s12955-020-01591-x

48. Olsson Möller U, Beck I, Rydén L, Malmström M. A comprehensive approach to rehabilitation interventions following breast cancer treatment - a systematic review of systematic reviews. *BMC Cancer*. 2019;19(1):472. doi:10.1186/s12885-019-5648-7

49. Pedro J, Monteiro-Reis S, Carvalho-Maia C, Henrique R, Jerónimo C, Silva ER. Evidence of psychological and biological effects of structured Mindfulness-Based Interventions for cancer patients and survivors: A meta-review. *Psycho-Oncology*. 2021;30(11):1836-1848. doi:10.1002/pon.5771

50. Petrigna L, Zanghì M, Maugeri G, D’Agata V, Musumeci G. Methodological consideration for a physical activity intervention in breast cancer population: An umbrella review. *Heliyon*. 2023;9(7):e17470. doi:10.1016/j.heliyon.2023.e17470

51. Qiu L, Ye M, Tong Y, Jin Y. Promoting physical activity among cancer survivors: an umbrella review of systematic reviews. *Support Care Cancer*. 2023;31(5):301. doi:10.1007/s00520-023-07760-0

52. Rafn BS, Bodilsen A, von Heymann A, et al. Examining the efficacy of treatments for arm lymphedema in breast cancer survivors: an overview of systematic reviews with meta-analyses. *eClinicalMedicine*. 2024;67:102397. doi:10.1016/j.eclinm.2023.102397

53. Rapti C, Dinas PC, Chryssanthopoulos C, Mila A, Philippou A. Effects of Exercise and Physical Activity Levels on Childhood Cancer: An Umbrella Review. *Healthcare (Basel)*. 2023;11(6):820. doi:10.3390/healthcare11060820

54. Riccetti N, Werner AM, Ernst M, Hempler I, Singer S. Migrants and ethnic minorities with cancer: an umbrella review on their information and supportive care needs. *Onkologe*. 2021;27(2):133-144. doi:10.1007/s00761-020-00872-w

55. R King, et al. Psychosocial experiences of breast cancer survivors: a meta-review. *J Cancer Surviv*. 2024;18(1):84-123. doi:10.1007/s11764-023-01336-x

56. Schroter GT, Stopiglia RMM, Carvalho GL, et al. Osteoradionecrosis treatment in head and neck cancer patients: An overview of systematic reviews. *Spec Care Dentist*. 2024;44(3):621-635. doi:10.1111/scd.12910

57. Shi H, Yuan X, Fan W, Yang X, Liu G. An umbrella review of the evidence to guide decision-making in acupuncture therapies for chemotherapy-induced peripheral neuropathy. *J Cancer Res Clin Oncol*. 2023;149(17):15939-15955. doi:10.1007/s00432-023-05369-8

58. Slev VN, Mistiaen P, Pasman HRW, Verdonck-de Leeuw IM, van Uden-Kraan CF, Francke AL. Effects of eHealth for patients and informal caregivers confronted with cancer: A meta-review. *Int J Med Inform*. 2016;87:54-67. doi:10.1016/j.ijmedinf.2015.12.013

59. Specchia ML, Frisicale EM, Carini E, et al. The impact of tumor board on cancer care: evidence from an umbrella review. *BMC Health Serv Res*. 2020;20(1):73. doi:10.1186/s12913-020-4930-3

60. Sun Q, Wang K, Chen Y, Peng X, Jiang X, Peng J. Effectiveness of dyadic interventions among cancer dyads: An overview of systematic reviews and meta-analyses. *J Clin Nurs*. 2024;33(2):497-530. doi:10.1111/jocn.16890

61. Tuominen L, Stolt M, Meretoja R, Leino-Kilpi H. Effectiveness of nursing interventions among patients with cancer: An overview of systematic reviews. *J Clin Nurs*. 2019;28(13-14):2401-2419. doi:10.1111/jocn.14762

62. Trigueros-Murillo A, Martinez-Calderon J, Casuso-Holgado MJ, González-García P, Heredia-Rizo AM. Effects of music-based interventions on cancer-related pain, fatigue, and distress: an overview of systematic reviews. *Support Care Cancer*. 2023;31(8):488. doi:10.1007/s00520-023-07938-6

63. Tune T, Goh S, Williams PAH, Koczwara B. How Is Quality of mHealth Interventions for Cancer Survivors Defined and Described? An Umbrella Review. *JCO Clin Cancer Inform*. 2022;6:e2100203. doi:10.1200/CCI.21.00203

64. Vieira Nascimento M, Costa FWG, de Oliveira Filho OV, Silva PG de B, de Freitas Pontes KM. Management of Cancer Therapy-Induced Oral Mucositis Using Photobiomodulation Therapy: An Overview of Systematic Reviews. *Photobiomodul Photomed Laser Surg*. 2023;41(10):513-538. doi:10.1089/photob.2023.0091

65. Wang N, Chen J, Chen W, et al. The effectiveness of case management for cancer patients: an umbrella review. *BMC Health Serv Res*. 2022;22(1):1247. doi:10.1186/s12913-022-08610-1

66. Wang L, Du X, Hu P, Zhang Y, Yao M, Che X. Quality of evidence supporting the role of acupuncture for breast cancer-related lymphoedema: an overview of systematic reviews and meta-analyses. *J Cancer Res Clin Oncol*. 2023;149(18):16669-16678. doi:10.1007/s00432-023-05419-1

67. Wu X, Chung VCH, Hui EP, et al. Effectiveness of acupuncture and related therapies for palliative care of cancer: overview of systematic reviews. *Sci Rep*. 2015;5:16776. doi:10.1038/srep16776

68. Wu X, Chung VCH, Lu P, et al. Chinese Herbal Medicine for Improving Quality of Life Among Nonsmall Cell Lung Cancer Patients: Overview of Systematic Reviews and Network Meta-Analysis. *Medicine*. 2016;95(1):e2410. doi:10.1097/MD.0000000000002410

69. Xing W, Duan D, Ye C, et al. Effectiveness of manual lymphatic drainage for breast cancer-related lymphoedema: an overview of systematic reviews and meta-analyses. doi:10.22514/ejgo.2023.001

70. Zanghì M, Petrigna L, Maugeri G, D’Agata V, Musumeci G. The Practice of Physical Activity on Psychological, Mental, Physical, and Social Wellbeing for Breast-Cancer Survivors: An Umbrella Review. *Int J Environ Res Public Health*. 2022;19(16):10391. doi:10.3390/ijerph191610391

71. Zhang XW, Hou WB, Pu FL, et al. Acupuncture for cancer-related conditions: An overview of systematic reviews. *Phytomedicine*. 2022;106:154430. doi:10.1016/j.phymed.2022.154430

72. Zhang YB, Zhong XM, Han N, Tang H, Wang SY, Lin WX. Effectiveness of exercise interventions in the management of cancer-related fatigue: a systematic review of systematic reviews. *Support Care Cancer*. 2023;31(3):153. doi:10.1007/s00520-023-07619-4

73. Zhang Y, Yao F, Kuang X, et al. How Can Alternative Exercise Traditions Help Against the Background of the COVID-19 in Cancer Care? An Overview of Systematic Reviews. *Cancer Manag Res*. 2020;12:12927-12944. doi:10.2147/CMAR.S282491

74. Zhao Y, Tang L, Shao J, et al. The effectiveness of exercise on the symptoms in breast cancer patients undergoing adjuvant treatment: an umbrella review of systematic reviews and meta-analyses. *Front Oncol*. 2023;13:1222947. doi:10.3389/fonc.2023.1222947

75. Zhou HJ, Wang T, Xu YZ, et al. Effects of exercise interventions on cancer-related fatigue in breast cancer patients: an overview of systematic reviews. *Support Care Cancer*. 2022;30(12):10421-10440. doi:10.1007/s00520-022-07389-5

76. Zhou W, Woo S, Larson JL. Effects of perioperative exercise interventions on lung cancer patients: An overview of systematic reviews. *Journal of Clinical Nursing*. 2020;29(23-24):4482-4504. doi:10.1111/jocn.15511
