# Supplementary material for: Umbrella Reviews Conducted in an Oncology Healthcare Context Focusing on Supportive Care, Systems, and Models of Care: A Review of Umbrella Reviews
Source: Cancer Med. 2026 Mar 25;15(4):e71708. doi: 10.1002/cam4.71708 (PMC13140849; doi:10.1002/cam4.71708)
Supplement: Supplementary file 2 — Table S2: Quality assessment of included umbrella reviews using JBI quality assessment tool. [file CAM4-15-e71708-s003.docx]

TABLE S2 Quality assessment of included umbrella reviews using JBI quality assessment tool

| **Author, year** | **1. Is the review question clearly and explicitly stated?** | **2. Were the inclusion criteria appropriate for the review question?** | **3. Was the search strategy appropriate?** | **4. Were the sources and resources used to search for studies adequate?** | **5.****Were the criteria for appraising studies appropriate?** | **6.****Was critical appraisal conducted by two or more reviewers independently?** | **7.Were there methods to minimize errors in data extraction?** | **8.Were the methods used to combine studies appropriate?** | **9.Was the likelihood of publication bias assessed?** | **10.Were recommendations for polices and/or practice supported by the reported data?** | **11.Were the specific directives for new research appropriate?** | **Overall** |
| --- | --- | --- | --- | --- | --- | --- | --- | --- | --- | --- | --- | --- |
| Abu-Odah, 2020^[1](#r1)^ | Yes | Yes | Yes | Yes | Yes | Yes | Yes | Unclear | No | Yes | Yes | 9/11 |
| Adam, 2015^[2](#r2)^ | Yes | Yes | Yes | Yes | Yes | No | No | Yes | Not Applicable | Yes | Yes | 9/11 |
| Amatya,2021^[3](#r3)^ | Yes | Yes | Yes | Yes | Yes | Yes | No | Unclear | No | Yes | Yes | 9/11 |
| Bao, 2014^[4](#r4)^ | Yes | Yes | Yes | No | Yes | No | Yes | Unclear | No | Yes | Yes | 6/11 |
| Belloni a, 2021^[5](#r5)^ | Yes | Yes | Yes | No | Yes | Yes | Yes | Yes | Yes | Yes | Yes | 10/11 |
| Belloni b, 2023^[6](#r6)^ | Yes | Yes | Yes | No | Yes | Yes | Yes | Yes | No | Yes | Yes | 9/11 |
| Belloni c, 2023^[7](#r7)^ | Yes | Yes | Yes | No | Yes | Yes | Yes | Yes | Yes | Yes | Yes | 9/11 |
| Belloni d, 2023^[8](#r8)^ | Yes | Yes | Yes | No | Yes | Yes | Yes | Yes | Unclear | Yes | Yes | 9/11 |
| Belloni e, 2021^[9](#r9)^ | Yes | Yes | Yes | No | Yes | Yes | Yes | Yes | No | Yes | Yes | 9/11 |
| Bracchiglione, 2023^1^[^0^](#r10) | Yes | Yes | Yes | Yes | Yes | Yes | Yes | Yes | Yes | Yes | Yes | 11/11 |
| Butow, 2020^[11](#r11)^ | Yes | Yes | Yes | No | Yes | Yes | Yes | Yes | Not Applicable | Yes | Yes | 9/11 |
| Casuso-Holgado, 2022^[12](#r12)^ | Yes | Yes | Yes | No | Yes | Yes | No | Yes | No | Yes | No | 6/11 |
| Cedenilla Ramón, 2023^[13](#r13)^ | Yes | Yes | Yes | No | Yes | Yes | Yes | Unclear | No | Yes | Yes | 8/11 |
| Chan a, 2023^1^[^4^](#r14) | Yes | Yes | Yes | No | Yes | Yes | Yes | Yes | No | Yes | Yes | 9/11 |
| Chan b, 2021^[15](#r15)^ | Yes | Yes | Yes | No | Yes | Yes | Yes | Yes | No | Yes | Yes | 9/11 |
| Chan c, 2023^[16](#r16)^ | Yes | Yes | Yes | Yes | Yes | Yes | Yes | Yes | No | Yes | Yes | 10/11 |
| Choi, 2022^[17](#r17)^ | Yes | Yes | Yes | Yes | Yes | Yes | Yes | Yes | Not Applicable | Yes | Yes | 10/11 |
| Chung, 2015^[18](#r18)^ | Yes | Yes | Yes | Yes | Yes | Yes | Yes | Yes | No | Yes | Yes | 10/11 |
| Conway, 2015^[19](#r19)^ | Yes | Yes | Yes | Yes | Yes | Yes | Yes | Yes | No | Yes | Yes | 10/11 |
| Crawford‐Williams, 2018^[20](#r20)^ | Yes | Yes | Yes | Yes | Yes | No | No | Yes | No | Yes | Yes | 9/11 |
| Duncan, 2017^[21](#r21)^ | Yes | Yes | Yes | Yes | Yes | Unclear | Yes | Yes | No | Yes | Yes | 9/11 |
| Edbrooke, 2023^[22](#r22)^ | Yes | Yes | Yes | No | Yes | Yes | Yes | Yes | Yes | No | Yes | 8/11 |
| Fournier, 2023^[23](#r23)^ | Yes | Yes | Yes | No | Yes | Yes | Yes | Yes | No | Yes | Yes | 9/11 |
| Gkantaifi, 2020^[24](#r24)^ | Yes | Unclear | No | No | No | Not Applicable | Unclear | Unclear | No | Yes | Yes | 3/11 |
| Grimmett, 2022^[25](#r25)^ | Yes | No | Unclear | Yes | No | No | No | Unclear | No | Yes | Yes | 6/11 |
| Hall, 2022^[26](#r26)^ | Yes | Yes | Unclear | Yes | Yes | Yes | Yes | Yes | Yes | Yes | Yes | 10/11 |
| Hou, 2023^[27](#r27)^ | Yes | Yes | Yes | No | Yes | Yes | Yes | Yes | Yes | Yes | Yes | 9/11 |
| Huang, 2021^[28](#r28)^ | Yes | Yes | Yes | Yes | Yes | Yes | Yes | Yes | Yes | Yes | Yes | 11/11 |
| Jiang, 2020^[29](#r29)^ | Yes | Yes | Yes | Yes | Yes | Yes | Yes | Unclear | Yes | Yes | Yes | 10/11 |
| Khosroshahi a, 2023^[30](#r30)^ | Yes | Yes | Yes | No | Yes | Yes | Yes | Yes | Yes | Yes | Yes | 10/11 |
| Khosroshahi b, 2023^[31](#r31)^ | Yes | Yes | Yes | No | Yes | Yes | Yes | Yes | Yes | No | No | 3/11 |
| Khosroshahi c, 2022^[32](#r32)^ | Yes | Yes | Yes | No | Yes | Yes | Yes | Yes | Yes | Yes | Yes | 10/11 |
| Kim, 2018^[33](#r33)^ | Yes | Yes | Yes | No | Yes | Yes | Yes | Yes | Yes | Yes | Yes | 10/11 |
| Knowles, 2022^[34](#r34)^ | Yes | Yes | Yes | No | Yes | No | Yes | Unclear | No | No | Yes | 6/11 |
| Laidsaar-Powell, 2019^[35](#r35)^ | Yes | Yes | Yes | No | Yes | Unclear | Yes | Yes | Not Applicable | Yes | Yes | 8/11 |
| Lake, 2022^[36](#r36)^ | Yes | Yes | Yes | Yes | Yes | Yes | Yes | Yes | No | Yes | Yes | 10/11 |
| Lavasidis, 2022^[37](#r37)^ | Yes | Yes | Yes | No | Yes | Unclear | Unclear | Yes | Yes | Yes | Yes | 8/11 |
| Lee a, 2023^[38](#r38)^ | Yes | Yes | No | No | Yes | Yes | Yes | Yes | No | Yes | No | 7/11 |
| Lee b, 2018^[39](#r39)^ | Yes | Yes | Yes | No | Yes | Yes | Yes | Unclear | No | No | Yes | 7/11 |
| Leslie, 2022^[40](#r40)^ | Yes | Yes | Yes | No | Yes | Yes | Yes | Unclear | No | Yes | Yes | 8/11 |
| Licqurish, 2019^[41](#r41)^ | No | Yes | Yes | Yes | Yes | Yes | Yes | Yes | No | Yes | Yes | 9/11 |
| Li, 2023^[42](#r42)^ | No | Yes | Yes | Yes | Yes | Yes | Yes | No | Yes | Yes | Yes | 9/11 |
| Loh, 2015^[43](#r43)^ | Yes | Yes | Yes | No | Yes | Yes | Yes | Unclear | No | Yes | Yes | 8/11 |
| Mainou, 2023^[44](#r44)^ | No | No | Yes | No | Yes | No | Yes | No | No | No | No | 3/11 |
| Mazzocco, 2023^[45](#r45)^ | Yes | Yes | Yes | No | Yes | Yes | Unclear | Unclear | No | Yes | Yes | 8/11 |
| Mentink, 2023^[46](#r46)^ | No | Yes | Yes | No | Yes | Yes | No | Yes | Yes | Yes | Yes | 8/11 |
| Mokhtari-Hessari, 2020^[47](#r47)^ | Yes | Yes | No | No | Yes | Unclear | Unclear | Yes | Yes | No | No | 4/11 |
| Olsson Möller, 2019^[48](#r48)^ | Yes | Yes | Yes | Yes | Yes | Yes | Yes | Unclear | Yes | Yes | Yes | 10/11 |
| Pedro, 2021^[49](#r49)^ | Yes | Yes | Yes | Yes | Yes | Yes | Yes | Unclear | No | Yes | Yes | 9/11 |
| Petrigna, 2023^[50](#r50)^ | Yes | Yes | Yes | No | Yes | Yes | Yes | Yes | Yes | Yes | Yes | 10/11 |
| Qiu, 2023^[51](#r51)^ | No | Yes | Yes | Yes | Yes | Yes | Yes | Yes | No | Yes | Yes | 9/11 |
| Rafn, 2023^[52](#r52)^ | Yes | Yes | Yes | No | Yes | Yes | Yes | Yes | Yes | No | No | 8/11 |
| Rapti, 2023^[53](#r53)^ | Yes | Yes | Yes | No | Yes | Yes | Yes | Yes | No | Yes | Yes | 9/11 |
| Riccetti, 2021^[54](#r54)^ | Yes | Yes | Yes | No | Yes | Yes | Unclear | Unclear | No | Yes | Yes | 9/11 |
| King, 2023^[55](#r55)^ | Yes | No | Yes | No | Yes | Yes | Yes | Yes | Not Applicable | Yes | Yes | 6/11 |
| Schroter, 2023^[56](#r56)^ | Yes | Yes | Yes | No | Yes | Yes | Yes | Unclear | No | No | Yes | 8/11 |
| Shi, 2023^[57](#r57)^ | Yes | Yes | Yes | Yes | Yes | Yes | Yes | Yes | Yes | Yes | Yes | 11/11 |
| Slev, 2016^[58](#r58)^ | Yes | Yes | Yes | No | Yes | Yes | Yes | Yes | Not Applicable | Yes | Yes | 9/11 |
| Specchia, 2020^[59](#r59)^ | Yes | Yes | Yes | No | Yes | Yes | Yes | Unclear | No | Yes | Yes | 8/11 |
| Sun, 2023^[60](#r60)^ | Yes | Yes | Yes | Yes | Yes | Yes | Yes | Yes | Yes | Yes | Yes | 11/11 |
| Tuominen, 2018^[61](#r61)^ | Yes | Yes | Yes | No | Yes | Yes | Yes | Yes | No | Yes | Yes | 9/11 |
| Trigueros-Murillo, 2023^[62](#r62)^ | Yes | Yes | Yes | Yes | Yes | Yes | Yes | Yes | No | No | No | 8/11 |
| Tune, 2022^[63](#r63)^ | No | No | Yes | Yes | No | No | No | Yes | Not Applicable | Yes | Yes | 5/11 |
| Vieira Nascimento, 2023^[64](#r64)^ | Yes | Yes | Yes | Yes | Yes | Yes | Yes | No | No | No | No | 7/11 |
| Wang a, 2022^[65](#r65)^ | Yes | Yes | Yes | No | Yes | Yes | Yes | Yes | No | Yes | Yes | 9/11 |
| Wang b, 2023^[66](#r66)^ | Yes | Yes | Yes | Yes | Yes | Yes | Yes | Unclear | No | Yes | Yes | 9/11 |
| Wu a, 2015^[67](#r67)^ | Yes | Yes | Yes | Yes | Yes | Yes | Yes | Yes | No | Yes | Yes | 10/11 |
| Wu b, 2016^[68](#r68)^ | Yes | Yes | Yes | Yes | Yes | Yes | Yes | Yes | Yes | Yes | Yes | 11/11 |
| Xing, 2023^[69](#r69)^ | Yes | Yes | Yes | No | Yes | Yes | Yes | Unclear | No | Yes | Yes | 8/11 |
| Zanghi, 2022^[70](#r70)^ | Yes | Yes | Yes | No | Yes | Yes | Yes | Yes | No | No | No | 7/11 |
| Zhang a, 2022^[71](#r71)^ | Yes | Yes | Yes | No | Yes | Yes | Yes | Yes | Yes | Yes | Yes | 10/11 |
| Zhang b, 2023^[72](#r72)^ | Yes | Yes | Yes | No | Yes | Yes | Yes | Yes | Unclear | No | Yes | 8/11 |
| Zhang c, 2020^[73](#r73)^ | Yes | Unclear | Yes | Yes | Yes | No | Yes | Unclear | No | Yes | Yes | 7/11 |
| Zhao, 2023^[74](#r74)^ | Yes | Yes | Yes | Yes | Yes | Yes | Yes | Yes | No | Yes | Yes | 10/11 |
| Zhou a, 2022^[75](#r75)^ | Yes | Yes | Yes | Yes | Yes | Yes | Yes | Yes | Yes | Yes | Yes | 11/11 |
| Zhou b, 2020^[76](#r76)^ | No | Yes | Yes | Yes | Yes | Yes | Yes | Unclear | No | Yes | Yes | 9/11 |

Tool taken from [Aromataris E, Fernandez R, Godfrey C, Holly C, Kahlil H, Tungpunkom P. Summarizing systematic reviews: methodological development, conduct and reporting of an Umbrella review approach. Int J Evid Based Healthc. 2015;13(3):132-40](https://jbi.global/critical-appraisal-tools). [^77^](#r77)

Yes: Question was answered in UR

No: Question was not answered in UR

Unclear: Question was not explained sufficiently in UR

Not applicable: If fifth question (Were the criteria for appraising studies appropriate?) was answered as “No”, sixth question (Was critical appraisal conducted by two or more reviewers independently?) was considered as “Not Applicable”. If the eighth question (Were the methods used to combine studies appropriate?) was qualitative synthesis, ninth question (Was the likelihood of publication bias assessed?) was answered as “Not Applicable”.

**Overview of quality assessment**

The majority of included URs clearly stated the review question (n=70; 92%). A substantial number of URs had the relevant inclusion criteria, and PICO components were clearly defined (n=67; 88.1%). Almost all URs had an appropriate search strategy (n=73; 96%) and stated specific directives for new research (n=67; 88.1%). If a paper searched more than six databases, we accepted that they used adequate resources, we selected "yes" for the fourth question (Were the sources and resources used to search for studies adequate?). Less than half URs used adequate sources (n=34; 44.7%). Most of the URs conducted appropriate methods for quality appraisal (n=73; 96%), and they conducted the quality assessment by two or more reviewers (n=62; 81.5%). URs used adequate sources (n=34; 44.7%). Fifty-two URs (68.4%) used appropriate methods to combine studies, twenty URs (26.3%) used unclear methods, and four URs (5%) did not use appropriate methods to combine studies. The number of included six databases, we accepted that they used adequate resources, we selected "yes" for the fourth question (Were the sources and resources used to search for studies adequate?). Less than half URs used adequate sources (n=34; 44.7%). Most of the URs conducted appropriate methods for quality appraisal (n=73; 96%), and they conducted the quality assessment by two or more reviewers (n=62; 81.5%). URs used adequate sources (n=34; 44.7%). Fifty-two URs (68.4%) used appropriate methods to combine studies, twenty URs (26.3%) used unclear methods, and four URs (5%) did not use appropriate methods to combine studies. 84.1% of included URs minimized errors in data extraction (n=64) and 81.5% recommended for policy and future research (n=62). Fewer URs assessed the likelihood of publication bias (n=23; 30.2%).

**REFERENCES**

1. Abu-Odah H, Molassiotis A, Liu J. Challenges on the provision of palliative care for patients with cancer in low- and middle-income countries: a systematic review of reviews. *BMC Palliat Care*. 2020;19(1):55. doi:10.1186/s12904-020-00558-5

2. Adam R, Bond C, Murchie P. Educational interventions for cancer pain. A systematic review of systematic reviews with nested narrative review of randomized controlled trials. *Patient Educ Couns*. 2015;98(3):269-282. doi:10.1016/j.pec.2014.11.003

3. Amatya B, Khan F, Lew TE, Dickinson M. Rehabilitation in patients with lymphoma: An overview of Systematic Reviews. *J Rehabil Med*. 2021;53(3):jrm00163. doi:10.2340/16501977-2810

4. Bao Y, Kong X, Yang L, et al. Complementary and Alternative Medicine for Cancer Pain: An Overview of Systematic Reviews. *Evid Based Complement Alternat Med*. 2014;2014:170396. doi:10.1155/2014/170396

5. Belloni S, Arrigoni C, Caruso R. Effects from physical exercise on reduced cancer-related fatigue: a systematic review of systematic reviews and meta-analysis. *Acta Oncol*. 2021;60(12):1678-1687. doi:10.1080/0284186X.2021.1962543

6. Belloni S, Arrigoni C, Baroni I, et al. Non-pharmacologic interventions for improving cancer-related fatigue (CRF): A systematic review of systematic reviews and pooled meta-analysis. *Semin Oncol*. 2023;50(1-2):49-59. doi:10.1053/j.seminoncol.2023.03.004

7. Belloni S, Bonucci M, Arrigoni C, Dellafiore F, Caruso R. A Systematic Review of Systematic Reviews and a Pooled Meta-Analysis on Complementary and Integrative Medicine for Improving Cancer-Related Fatigue. *Clin Ther*. 2023;45(1):e54-e73. doi:10.1016/j.clinthera.2022.12.001

8. Belloni S, Arrigoni C, Arcidiacono MA, et al. A Systematic Review of Systematic Reviews and Pooled Meta-Analysis on Psychosocial Interventions for Improving Cancer-Related Fatigue. *Semin Oncol Nurs*. 2023;39(3):151354. doi:10.1016/j.soncn.2022.151354

9. Belloni S, Arrigoni C, de Sanctis R, Arcidiacono MA, Dellafiore F, Caruso R. A systematic review of systematic reviews and pooled meta-analysis on pharmacological interventions to improve cancer-related fatigue. *Critical Reviews in Oncology/Hematology*. 2021;166:103373. doi:10.1016/j.critrevonc.2021.103373

10. Bracchiglione J, Rodríguez-Grijalva G, Requeijo C, et al. Systemic Oncological Treatments versus Supportive Care for Patients with Advanced Hepatobiliary Cancers: An Overview of Systematic Reviews. *Cancers (Basel)*. 2023;15(3):766. doi:10.3390/cancers15030766

11. Butow P, Laidsaar-Powell R, Konings S, Lim CYS, Koczwara B. Return to work after a cancer diagnosis: a meta-review of reviews and a meta-synthesis of recent qualitative studies. *J Cancer Surviv*. 2020;14(2):114-134. doi:10.1007/s11764-019-00828-z

12. Casuso-Holgado MJ, Heredia-Rizo AM, Gonzalez-Garcia P, Muñoz-Fernández MJ, Martinez-Calderon J. Mind-body practices for cancer-related symptoms management: an overview of systematic reviews including one hundred twenty-nine meta-analyses. *Support Care Cancer*. 2022;30(12):10335-10357. doi:10.1007/s00520-022-07426-3

13. Cedenilla Ramón N, Calvo Arenillas JI, Aranda Valero S, Sánchez Guzmán A, Moruno Miralles P. Psychosocial Interventions for the Treatment of Cancer-Related Fatigue: An Umbrella Review. *Curr Oncol*. 2023;30(3):2954-2977. doi:10.3390/curroncol30030226

14. Chan RJ, Milch VE, Crawford-Williams F, et al. Patient navigation across the cancer care continuum: An overview of systematic reviews and emerging literature. *CA Cancer J Clin*. 2023;73(6):565-589. doi:10.3322/caac.21788

15. Chan RJ, Crichton M, Crawford-Williams F, et al. The efficacy, challenges, and facilitators of telemedicine in post-treatment cancer survivorship care: an overview of systematic reviews. *Ann Oncol*. 2021;32(12):1552-1570. doi:10.1016/j.annonc.2021.09.001

16. Chan RJ, Crawford-Williams F, Crichton M, et al. Effectiveness and implementation of models of cancer survivorship care: an overview of systematic reviews. *J Cancer Surviv*. 2023;17(1):197-221. doi:10.1007/s11764-021-01128-1

17. Choi TY, Ang L, Jun JH, Alraek T, Lee MS. Acupuncture and Moxibustion for Cancer-Related Fatigue: An Overview of Systematic Reviews and Meta-Analysis. *Cancers (Basel)*. 2022;14(10):2347. doi:10.3390/cancers14102347

18. Chung VCH, Wu X, Hui EP, et al. Effectiveness of Chinese herbal medicine for cancer palliative care: overview of systematic reviews with meta-analyses. *Sci Rep*. 2015;5:18111. doi:10.1038/srep18111

19. Conway A, McCarthy AL, Lawrence P, Clark RA. The prevention, detection and management of cancer treatment-induced cardiotoxicity: a meta-review. *BMC Cancer*. 2015;15:366. doi:10.1186/s12885-015-1407-6

20. Crawford-Williams F, March S, Goodwin BC, et al. Interventions for prostate cancer survivorship: A systematic review of reviews. *Psychooncology*. 2018;27(10):2339-2348. doi:10.1002/pon.4888

21. Duncan M, Moschopoulou E, Herrington E, et al. Review of systematic reviews of non-pharmacological interventions to improve quality of life in cancer survivors. *BMJ Open*. 2017;7(11):e015860. doi:10.1136/bmjopen-2017-015860

22. Edbrooke L, Bowman A, Granger CL, et al. Exercise across the Lung Cancer Care Continuum: An Overview of Systematic Reviews. *Journal of Clinical Medicine*. 2023;12(5):1871. doi:10.3390/jcm12051871

23. Fournier V, Duprez C, Grynberg D, Antoine P, Lamore K. Are digital health interventions valuable to support patients with cancer and caregivers? An umbrella review of web-based and app-based supportive care interventions. *Cancer Med*. 2023;12(23):21436-21451. doi:10.1002/cam4.6695

24. Gkantaifi A, Alongi F, Vardas E, et al. Honey Against Radiation-induced Oral Mucositis in Head and Neck Cancer Patients. An Umbrella Review of Systematic Reviews and Meta- Analyses of the Literature. *Rev Recent Clin Trials*. 2020;15(4):360-369. doi:10.2174/1574887115666200709140405

25. Grimmett C, Heneka N, Chambers S. Psychological Interventions Prior to Cancer Surgery: a Review of Reviews. *Curr Anesthesiol Rep*. 2022;12(1):78-87. doi:10.1007/s40140-021-00505-x

26. Hall LH, King NV, Graham CD, et al. Strategies to self-manage side-effects of adjuvant endocrine therapy among breast cancer survivors: an umbrella review of empirical evidence and clinical guidelines. *J Cancer Surviv*. 2022;16(6):1296-1338. doi:10.1007/s11764-021-01114-7

27. Hou W, Zhai L, Yang Y, et al. Is physical activity effective against cancer-related fatigue in lung cancer patients? An umbrella review of systematic reviews and meta-analyses. *Support Care Cancer*. 2023;31(3):161. doi:10.1007/s00520-023-07627-4

28. Huang J, Liu H, Chen J, Cai X, Huang Y. The Effectiveness of Tai Chi in Patients With Breast Cancer: An Overview of Systematic Reviews and Meta-Analyses. *Journal of Pain and Symptom Management*. 2021;61(5):1052-1059. doi:10.1016/j.jpainsymman.2020.10.007

29. Jiang M, Ma Y, Yun B, Wang Q, Huang C, Han L. Exercise for fatigue in breast cancer patients: An umbrella review of systematic reviews. *Int J Nurs Sci*. 2020;7(2):248-254. doi:10.1016/j.ijnss.2020.03.001

30. Amiri Khosroshahi R, Zeraattalab-Motlagh S, Sarsangi P, Nielsen SM, Mohammadi H. Effect of probiotic supplementation on chemotherapy- and radiotherapy-related diarrhoea in patients with cancer: an umbrella review of systematic reviews and meta-analyses. *Br J Nutr*. 2023;130(10):1754-1765. doi:10.1017/S0007114523000910

31. Amiri Khosroshahi R, Talebi S, Zeraattalab-Motlagh S, et al. Nutritional interventions for the prevention and treatment of cancer therapy-induced oral mucositis: an umbrella review of systematic reviews and meta-analysis. *Nutrition Reviews*. 2023;81(9):1200-1212. doi:10.1093/nutrit/nuac105

32. Khosroshahi RA, Talebi S, Travica N, Mohammadi H. Cryotherapy for oral mucositis in cancer: review of systematic reviews and meta-analysis. *BMJ Supportive & Palliative Care*. 2023;13(e3):e570-e577. doi:10.1136/spcare-2022-003636

33. Kim TH, Kang JW, Lee TH. Therapeutic options for aromatase inhibitor-associated arthralgia in breast cancer survivors: A systematic review of systematic reviews, evidence mapping, and network meta-analysis. *Maturitas*. 2018;118:29-37. doi:10.1016/j.maturitas.2018.09.005

34. Knowles R, Kemp E, Miller M, Davison K, Koczwara B. Physical activity interventions in older people with cancer: A review of systematic reviews. *Eur J Cancer Care (Engl)*. 2022;31(5):e13637. doi:10.1111/ecc.13637

35. Laidsaar-Powell R, Konings S, Rankin N, et al. A meta-review of qualitative research on adult cancer survivors: current strengths and evidence gaps. J Cancer Surviv. 2019;13(6):852-889. doi:10.1007/s11764-019-00803-8

36. Lake B, Damery S, Jolly K. Effectiveness of weight loss interventions in breast cancer survivors: a systematic review of reviews. *BMJ Open*. 2022;12(10):e062288. doi:10.1136/bmjopen-2022-062288

37. Lavasidis G, Markozannes G, Voorhies K, et al. Supportive interventions for childhood cancer: An umbrella review of randomized evidence. *Crit Rev Oncol Hematol*. 2022;180:103845. doi:10.1016/j.critrevonc.2022.103845

38. Lee CC, Kuo SF, Chang WP, Guo SL, Huang TW. Effectiveness of Cryotherapy on Cancer Therapy-Induced Oral Mucositis: An Umbrella Review. *Cancer Nurs*. 2023;46(5):E288-E296. doi:10.1097/NCC.0000000000001128

39. Lee SM, Choi HC, Hyun MK. An Overview of Systematic Reviews: Complementary Therapies for Cancer Patients. *Integr Cancer Ther*. 2019;18:1534735419890029. doi:10.1177/1534735419890029

40. Leslie M, Beatty L, Hulbert-Williams L, et al. Web-Based Psychological Interventions for People Living With and Beyond Cancer: Meta-Review of What Works and What Does Not for Maximizing Recruitment, Engagement, and Efficacy. *JMIR Cancer*. 2022;8(3):e36255. doi:10.2196/36255

41. Licqurish SM, Cook OY, Pattuwage LP, et al. Tools to facilitate communication during physician-patient consultations in cancer care: An overview of systematic reviews. *CA Cancer J Clin*. 2019;69(6):497-520. doi:10.3322/caac.21573

42. Li P, Wang Q, Liu L, et al. The Role of Complementary and Alternative Medicine on Cancer-Related Fatigue in Adults: An Overview of Systematic Reviews. *Integr Cancer Ther*. 2023;22:15347354231188947. doi:10.1177/15347354231188947

43. Loh SY, Musa AN. Methods to improve rehabilitation of patients following breast cancer surgery: a review of systematic reviews. *Breast Cancer (Dove Med Press)*. 2015;7:81-98. doi:10.2147/BCTT.S47012

44. Mainou M, Bougioukas KI, Malandris K, et al. Reporting of adverse events of treatment interventions in multiple myeloma: an overview of systematic reviews. *Ann Hematol*. 2024;103(8):2681-2697. doi:10.1007/s00277-023-05517-7

45. Mazzocco K, Milani A, Ciccarelli C, Marzorati C, Pravettoni G. Evidence for Choosing Qigong as an Integrated Intervention in Cancer Care: An Umbrella Review. *Cancers (Basel)*. 2023;15(4):1176. doi:10.3390/cancers15041176

46. Mentink M, Verbeek D, Noordman J, Timmer-Bonte A, von Rosenstiel I, van Dulmen S. The Effects of Complementary Therapies on Patient-Reported Outcomes: An Overview of Recent Systematic Reviews in Oncology. *Cancers*. 2023;15(18):4513. doi:10.3390/cancers15184513

47. Mokhtari-Hessari P, Montazeri A. Health-related quality of life in breast cancer patients: review of reviews from 2008 to 2018. *Health Qual Life Outcomes*. 2020;18(1):338. doi:10.1186/s12955-020-01591-x

48. Olsson Möller U, Beck I, Rydén L, Malmström M. A comprehensive approach to rehabilitation interventions following breast cancer treatment - a systematic review of systematic reviews. *BMC Cancer*. 2019;19(1):472. doi:10.1186/s12885-019-5648-7

49. Pedro J, Monteiro-Reis S, Carvalho-Maia C, Henrique R, Jerónimo C, Silva ER. Evidence of psychological and biological effects of structured Mindfulness-Based Interventions for cancer patients and survivors: A meta-review. *Psycho-Oncology*. 2021;30(11):1836-1848. doi:10.1002/pon.5771

50. Petrigna L, Zanghì M, Maugeri G, D’Agata V, Musumeci G. Methodological consideration for a physical activity intervention in breast cancer population: An umbrella review. *Heliyon*. 2023;9(7):e17470. doi:10.1016/j.heliyon.2023.e17470

51. Qiu L, Ye M, Tong Y, Jin Y. Promoting physical activity among cancer survivors: an umbrella review of systematic reviews. *Support Care Cancer*. 2023;31(5):301. doi:10.1007/s00520-023-07760-0

52. Rafn BS, Bodilsen A, von Heymann A, et al. Examining the efficacy of treatments for arm lymphedema in breast cancer survivors: an overview of systematic reviews with meta-analyses. *eClinicalMedicine*. 2024;67:102397. doi:10.1016/j.eclinm.2023.102397

53. Rapti C, Dinas PC, Chryssanthopoulos C, Mila A, Philippou A. Effects of Exercise and Physical Activity Levels on Childhood Cancer: An Umbrella Review. *Healthcare (Basel)*. 2023;11(6):820. doi:10.3390/healthcare11060820

54. Riccetti N, Werner AM, Ernst M, Hempler I, Singer S. Migrants and ethnic minorities with cancer: an umbrella review on their information and supportive care needs. *Onkologe*. 2021;27(2):133-144. doi:10.1007/s00761-020-00872-w

55. R King, et al. Psychosocial experiences of breast cancer survivors: a meta-review. *J Cancer Surviv*. 2024;18(1):84-123. doi:10.1007/s11764-023-01336-x

56. Schroter GT, Stopiglia RMM, Carvalho GL, et al. Osteoradionecrosis treatment in head and neck cancer patients: An overview of systematic reviews. *Spec Care Dentist*. 2024;44(3):621-635. doi:10.1111/scd.12910

57. Shi H, Yuan X, Fan W, Yang X, Liu G. An umbrella review of the evidence to guide decision-making in acupuncture therapies for chemotherapy-induced peripheral neuropathy. *J Cancer Res Clin Oncol*. 2023;149(17):15939-15955. doi:10.1007/s00432-023-05369-8

58. Slev VN, Mistiaen P, Pasman HRW, Verdonck-de Leeuw IM, van Uden-Kraan CF, Francke AL. Effects of eHealth for patients and informal caregivers confronted with cancer: A meta-review. *Int J Med Inform*. 2016;87:54-67. doi:10.1016/j.ijmedinf.2015.12.013

59. Specchia ML, Frisicale EM, Carini E, et al. The impact of tumor board on cancer care: evidence from an umbrella review. *BMC Health Serv Res*. 2020;20(1):73. doi:10.1186/s12913-020-4930-3

60. Sun Q, Wang K, Chen Y, Peng X, Jiang X, Peng J. Effectiveness of dyadic interventions among cancer dyads: An overview of systematic reviews and meta-analyses. *J Clin Nurs*. 2024;33(2):497-530. doi:10.1111/jocn.16890

61. Tuominen L, Stolt M, Meretoja R, Leino-Kilpi H. Effectiveness of nursing interventions among patients with cancer: An overview of systematic reviews. *J Clin Nurs*. 2019;28(13-14):2401-2419. doi:10.1111/jocn.14762

62. Trigueros-Murillo A, Martinez-Calderon J, Casuso-Holgado MJ, González-García P, Heredia-Rizo AM. Effects of music-based interventions on cancer-related pain, fatigue, and distress: an overview of systematic reviews. *Support Care Cancer*. 2023;31(8):488. doi:10.1007/s00520-023-07938-6

63. Tune T, Goh S, Williams PAH, Koczwara B. How Is Quality of mHealth Interventions for Cancer Survivors Defined and Described? An Umbrella Review. *JCO Clin Cancer Inform*. 2022;6:e2100203. doi:10.1200/CCI.21.00203

64. Vieira Nascimento M, Costa FWG, de Oliveira Filho OV, Silva PG de B, de Freitas Pontes KM. Management of Cancer Therapy-Induced Oral Mucositis Using Photobiomodulation Therapy: An Overview of Systematic Reviews. *Photobiomodul Photomed Laser Surg*. 2023;41(10):513-538. doi:10.1089/photob.2023.0091

65. Wang N, Chen J, Chen W, et al. The effectiveness of case management for cancer patients: an umbrella review. *BMC Health Serv Res*. 2022;22(1):1247. doi:10.1186/s12913-022-08610-1

66. Wang L, Du X, Hu P, Zhang Y, Yao M, Che X. Quality of evidence supporting the role of acupuncture for breast cancer-related lymphoedema: an overview of systematic reviews and meta-analyses. *J Cancer Res Clin Oncol*. 2023;149(18):16669-16678. doi:10.1007/s00432-023-05419-1

67. Wu X, Chung VCH, Hui EP, et al. Effectiveness of acupuncture and related therapies for palliative care of cancer: overview of systematic reviews. *Sci Rep*. 2015;5:16776. doi:10.1038/srep16776

68. Wu X, Chung VCH, Lu P, et al. Chinese Herbal Medicine for Improving Quality of Life Among Nonsmall Cell Lung Cancer Patients: Overview of Systematic Reviews and Network Meta-Analysis. *Medicine*. 2016;95(1):e2410. doi:10.1097/MD.0000000000002410

69. Xing W, Duan D, Ye C, et al. Effectiveness of manual lymphatic drainage for breast cancer-related lymphoedema: an overview of systematic reviews and meta-analyses. doi:10.22514/ejgo.2023.001

70. Zanghì M, Petrigna L, Maugeri G, D’Agata V, Musumeci G. The Practice of Physical Activity on Psychological, Mental, Physical, and Social Wellbeing for Breast-Cancer Survivors: An Umbrella Review. *Int J Environ Res Public Health*. 2022;19(16):10391. doi:10.3390/ijerph191610391

71. Zhang XW, Hou WB, Pu FL, et al. Acupuncture for cancer-related conditions: An overview of systematic reviews. *Phytomedicine*. 2022;106:154430. doi:10.1016/j.phymed.2022.154430

72. Zhang YB, Zhong XM, Han N, Tang H, Wang SY, Lin WX. Effectiveness of exercise interventions in the management of cancer-related fatigue: a systematic review of systematic reviews. *Support Care Cancer*. 2023;31(3):153. doi:10.1007/s00520-023-07619-4

73. Zhang Y, Yao F, Kuang X, et al. How Can Alternative Exercise Traditions Help Against the Background of the COVID-19 in Cancer Care? An Overview of Systematic Reviews. *Cancer Manag Res*. 2020;12:12927-12944. doi:10.2147/CMAR.S282491

74. Zhao Y, Tang L, Shao J, et al. The effectiveness of exercise on the symptoms in breast cancer patients undergoing adjuvant treatment: an umbrella review of systematic reviews and meta-analyses. *Front Oncol*. 2023;13:1222947. doi:10.3389/fonc.2023.1222947

75. Zhou HJ, Wang T, Xu YZ, et al. Effects of exercise interventions on cancer-related fatigue in breast cancer patients: an overview of systematic reviews. *Support Care Cancer*. 2022;30(12):10421-10440. doi:10.1007/s00520-022-07389-5

76. Zhou W, Woo S, Larson JL. Effects of perioperative exercise interventions on lung cancer patients: An overview of systematic reviews. *Journal of Clinical Nursing*. 2020;29(23-24):4482-4504. doi:10.1111/jocn.15511

77. JBI Critical Appraisal Tools | JBI. Accessed July 30, 2024. https://jbi.global/critical-appraisal-tools
