# Supplementary material for: Umbrella Reviews Conducted in an Oncology Healthcare Context Focusing on Supportive Care, Systems, and Models of Care: A Review of Umbrella Reviews
Source: Cancer Med. 2026 Mar 25;15(4):e71708. doi: 10.1002/cam4.71708 (PMC13140849; doi:10.1002/cam4.71708)
Supplement: Supplementary file 3 — Table S3: Details of papers excluded at full text review stage. [file CAM4-15-e71708-s001.docx]

TABLE S3 Details of papers excluded at full text review stage

| **Reasons** | **References** |
| --- | --- |
| Protocol | Aguilera-Eguía RA, Gutiérrez-Arias R, Zaror C, Seron P. Effectiveness of physical exercise programmes in reducing complications associated with secondary lymphoedema to breast cancer: a protocol for an overview of systematic reviews. *BMJ open*. 2023;13(7):e071630. doi:10.1136/bmjopen-2023-071630 |
| Wrong patient population | Arayici ME, Basbinar Y, Ellidokuz H. The impact of cancer on the severity of disease in patients affected with COVID-19: an umbrella review and meta-meta-analysis of systematic reviews and meta-analyses involving 1,064,476 participants. *Clinical and experimental medicine*. Published online 2022. doi:10.1007/s10238-022-00911-3 |
| Date incorrect | Brouwers MC, Garcia K, Makarski J, Daraz L, Evidence Expert Panel, KT for Cancer Control in Canada Project Research Team. The landscape of knowledge translation interventions in cancer control: what do we know and where to next? A review of systematic reviews. Implementation science : IS. 2011;6((Brouwers M.C.) McMaster University, Department of Oncology, Hamilton, Ontario, Canada.(Garcia K.; Makarski J.; Daraz L.; Evidence Expert Panel; KT for Cancer Control in Canada Project Research Team)):130. doi:10.1186/1748-5908-6-130 |
| Oral abstract | Chung VCH, Wu XY, Hui EP, et al. Effectiveness and safety of Chinese herbal medicine for cancer palliative care: Overview of systematic reviews with meta-analysis. Journal of Alternative and Complementary Medicine. 2016;22(6):A56. doi:10.1089/acm.2016.29003.abstracts |
| Wrong study design | Cocchiara RA, Sciarra I, D’Egidio V, et al. Returning to work after breast cancer: A systematic review of reviews. *Work*. 2018;61(3):463-476. doi:10.3233/WOR-182810 |
| Wrong study design | Contri A, Paltrinieri S, Torreggiani M, et al. Patient-reported outcome measure to implement routine assessment of cancer survivors’ unmet needs: An overview of reviews and COSMIN analysis. Cancer Treatment Reviews. 2023;120:N.PAG-N.PAG. doi:10.1016/j.ctrv.2023.102622 |
| Wrong outcomes | Cooper K, Campbell F, Harnan S, Sutton A. Association between stress, depression or anxiety and cancer: Rapid review of. Compr Psychoneuroendocrinol. doi: 10.1016/j.cpnec.2023.100215 |
| Scoping review | Edney LC, Roseleur J, Gray J, Koczwara B, Karnon J. Mapping a decade of interventions to address the supportive care needs of individuals living with or beyond cancer: a scoping review of reviews. Supportive Care in Cancer. 2022;30(5):3793-3804. doi:10.1007/s00520-021-06713-9 |
| Date incorrect | Ernst E, Lee MS. Acupuncture for palliative and supportive cancer care: A systematic review of systematic reviews. *Journal of Pain and Symptom Management*. 2010;40(1):e3-e5. doi:10.1016/j.jpainsymman.2010.03.010 |
| Poster abstract | Gerges M, Mazariego C, Meyers J, Gellert B, Sheppard J. Australian cancer survivors’ unmet supportive care needs: A meta-review of what’s next? *Asia-Pacific Journal of Clinical Oncology*. 2022;18((Gerges M.; Meyers J.; Gellert B.; Sheppard J.) Cancer Council NSW, Woolloomooloo, NSW, Australia(Mazariego C.) Daffodil Centre, University of Sydney,a joint venture with Cancer Council NSW, Sydney, NSW, Australia):212. doi:10.1111/ajco.13869 |
| Wrong outcomes | Goldkuhle M, Narayan VM, Weigl A, Dahm P, Skoetz N. A systematic assessment of Cochrane reviews and systematic reviews published in high-impact medical journals related to cancer. *BMJ open*. 2018;8(3):e020869. doi:10.1136/bmjopen-2017-020869 |
| Wrong intervention | Gualtieri P, Cianci R, Frank G, et al. Pancreatic Ductal Adenocarcinoma and Nutrition: Exploring the Role of Diet and Gut Health. Nutrients. 2023;15(20):4465. doi:10.3390/nu15204465 |
| Included multiple types of reviews | Guccione L, Fullerton S, Gough K, et al. Why is advance care planning underused in oncology settings? A systematic overview of reviews to identify the benefits, barriers, enablers, and interventions to improve uptake. Frontiers in oncology. 2023;13:1040589. doi:10.3389/fonc.2023.1040589 |
| Poster abstract | He S, Shepherd H, Agar M, Shaw J. Implementation of geriatric assessments in cancer care: An umbrella review. *Asia-Pacific Journal of Clinical Oncology*. 2022;18((He S.; Shaw J.) School of Psychology, Faculty of Science, University of Sydney, Camperdown, NSW, Australia(Shepherd H.) Susan Wakil School of Nursing and Midwifery, Faculty of Medicine and Health, University of Sydney, Camperdown, NSW, Australia(Agar M.)):184. doi:10.1111/ajco.13869 |
| Protocol | He Y, Liu Y, May BH, et al. Effectiveness of acupuncture for cancer pain: protocol for an umbrella review and meta-analyses of controlled trials. *BMJ open*. 2017;7(12):e018494. doi:10.1136/bmjopen-2017-018494 |
| Abstract book | Jheng YS, Chen SY, Tang ST. Posttraumatic Growth in Cancer Patients: A Review of Systematic Reviews and Meta-analyses. *Palliative Medicine*. 2023;37(1):174. doi:10.1177/02692163231172891 |
| Wrong study design | Kemp E, Geerse O, Knowles R, Nekhlyudov L, Mohammadi L, Koczwara B. An umbrella review of interventions for breast cancer survivors: Mapping available evidence according to the Quality of Cancer Survivorship Care Framework. Asia-Pacific Journal of Clinical Oncology. 2020;16(SUPPL 8):193. doi:10.1111/ajco.13498 |
| Poster abstract | Kemp E, Lawn S, Clark RA, et al. Interventions to manage cardiovascular disease risk in cancer patients and survivors: An umbrella review. *Asia-Pacific Journal of Clinical Oncology*. 2020;16(SUPPL 8):136. doi:10.1111/ajco.13498 |
| Poster abstract | Knowles R, Kemp E, Miller M, Koczwara B. The impact of lifestyle interventions on health-related outcomes in older people with cancer: An umbrella review. *Asia-Pacific Journal of Clinical Oncology*. 2020;16(SUPPL 8):193. doi:10.1111/ajco.13498 |
| Date incorrect | Lepore SJ, Coyne JC. Psychological interventions for distress in cancer patients: A review of reviews. Annals of Behavioral Medicine. 2006;32(2):85-92. doi:10.1207/s15324796abm3202_2 |
| Oral abstract | Licqurish S, Pattuwage L, Chima S, Qama A, Emery J. Interventions for maximizing quality communication in cancer care: A systematic review of systematic reviews. *Journal of Global Oncology*. 2018;4((Licqurish S.) University of Melbourne Centre for Cancer Research, VIC, Australia(Pattuwage L.; Chima S.; Emery J.) University of Melbourne Centre for Cancer Research, University of Melbourne, Australia(Qama A.) Doherty Institute, WHO Collaborating Centre):99s. doi:10.1200/jgo.18.48000 |
| Correction | Mokhtari-Hessari P, Montazeri A. Correction to: Health-related quality of life in breast cancer patients: review of reviews from 2008 to 2018. *Health and quality of life outcomes*. 2022;20(1):35. doi:10.1186/s12955-022-01942-w |
| Wrong outcomes | Muka T, Li JJX, Farahani SJ, Ioannidis JPA. Changes in cancer prevention and management and patient needs during the COVID-19 pandemic: An umbrella review of systematic reviews. medRxiv. 2022;((Muka T.) Institute of Social and Preventive Medicine, University of Bern, Bern, Switzerland(Muka T.; Ioannidis J.P.A., jioannid@stanford.edu) Meta-Research Innovation Center at Stanford (METRICS), Stanford University, Stanford, CA, United States(Li J.J.X). doi:10.1101/2022.12.18.22283642 |
| Wrong outcomes | Palmer Kelly E, Paredes AZ, Tsilimigras DI, Hyer JM, Pawlik TM. The role of religion and spirituality in cancer care: An umbrella review of the literature. Surgical oncology. 2022;42:101389. doi:10.1016/j.suronc.2020.05.004 |
| Wrong study design | Pearson SE, Taylor J, Patel P, Baguley DM. Cancer survivors treated with platinum-based chemotherapy affected by ototoxicity and the impact on quality of life: a narrative synthesis systematic review. International Journal of Audiology. 2019;58(11):685-695. doi:10.1080/14992027.2019.1660918 |
| Wrong interventio | Rammant E, Van Wilder L, Van Hemelrijck M, et al. Health-related quality of life overview after different curative treatment options in muscle-invasive bladder cancer: an umbrella review. *Quality of life research : an international journal of quality of life aspects of treatment, care and rehabilitation*. 2020;29(11):2887-2910. doi:10.1007/s11136-020-02544-z |
| Wrong study design | Redondo-Sánchez D, Petrova D, Rodríguez-Barranco M, Fernández-Navarro P, Jiménez-Moleón JJ, Sánchez MJ. Socio-Economic Inequalities in Lung Cancer Outcomes: An Overview of Systematic Reviews. *Cancers*. 2022;14(2). doi:10.3390/cancers14020398 |
| Wrong language | Reif K, de Vries U, Petermann F. What does really help against cancer-related fatigue? An overview of systematic reviews. *Pflege*. 2012;25(6):439-457. doi: 10.1024/1012-5302/a000246 |
| Wrong study design | Sasaki Y, Cheon C, Motoo Y, et al. [Complementary and Alternative Medicine for Breast Cancer Patients: An Overview of Systematic Reviews]. *Yakugaku zasshi : Journal of the Pharmaceutical Society of Japan*. 2019;139(7):1027-1046. doi:10.1248/yakushi.18-00215 |
| Scoping review | Shaffer KM, Turner KL, Siwik C, et al. Digital health and telehealth in cancer care: a scoping review of reviews. *The Lancet Digital health*. 2023;5(5):e316-e327. doi:10.1016/S2589-7500(23)00049-3 |
| Scoping review | Smrke U, Mlakar I, Lin S, Musil B, Plohl N. Language, Speech, and Facial Expression Features for Artificial Intelligence-Based Detection of Cancer Survivors’ Depression: Scoping Meta-Review. JMIR mental health. 2021;8(12):e30439. doi:10.2196/30439 |
| Abstract book | Sulosaari V, Beurskens J, Erickson N, Laviano A, Torcato Parreira S. Nutrition in people with cancer: Overview of reviews to summarise the evidence for cancer nursing practice. *Annals of Oncology*. 2021;32((Sulosaari V.) Health and Well-being, Turku University of Applied Sciences, Turku, Finland(Beurskens J.) Intestinal Failure Unit, Radboud University Medical Centre, Hb Nijmegen, Netherlands(Erickson N.) Ludwig Maximilian University Clinic, Comprehensive C):S1278. doi:10.1016/j.annonc.2021.08.692 |
| Abstract book | Tedla M, Stevens S, Wilson J, Moore D, Mehanna H. Dysphagia, other functional disorders and interventions to improve the symptoms 6-12 months after head and neck cancer treatment. Systematic review of systematic reviews. *Dysphagia*. 2016;31(2):325. doi:10.1007/s00455-016-9698-6 |
| Wrong study design | Towler P, Molassiotis A, Brearley SG. What is the evidence for the use of acupuncture as an intervention for symptom management in cancer supportive and palliative care: an integrative overview of reviews. *Supportive Care in Cancer*. 2013;21(10):2913-2923. doi:10.1007/s00520-013-1882-8 |
| Integrative review | Treanor CJ, Li J, Donnelly M. Cognitive impairment among prostate cancer patients: An overview of reviews. European Journal of Cancer Care. 2017;26(6):n/a-N.PAG. doi:10.1111/ecc.12642 |
| Abstract book | Wallen M, Joseph R, Dick Y, et al. PREHABILITATION before CANCER TREATMENT AN OVERVIEW of SYSTEMATIC REVIEWS. *Supportive Care in Cancer*. 2023;31((Wallen M.; Joseph R.; Dick Y.; Han C.Y.; Chan R.J.) Flinders University, Caring Futures Institute, Adelaide, Australia(Cheng L.J.) National University Singapore, Saw Swee Hock School of Public Health, Singapore, Singapore(Hart N.H.) University of Technol):S257. doi:10.1007/s00520-023-07786-4 |
| Wrong language | Wang YX, Li HP, Jiang XX, Zhang MM, Wang HX, Ding XT. Overview of review on the prevention and treatment of breast cancer-related lymphedema. *Chinese Journal of Cancer Prevention and Treatment*. 2019;26(8):588-594. doi: 10.3389/fonc.2022.1062472 |
| Oral abstract | Wu XY, Chung VCH, Lu P, et al. Chinese herbal medicine for improving quality of life in non-small cell lung cancer patients: Overview of systematic reviews and network meta-analysis. *Journal of Alternative and Complementary Medicine*. 2016;22(6):A55. doi:10.1089/acm.2016.29003.abstracts |
| Protocol | Xu J, Li H, Sze DMY, Chan VWS, Yang AWH. Effectiveness of qigong and tai chi in the quality of life of patients with cancer: protocol for an umbrella review. *BMJ open*. 2022;12(4):e057980. doi:10.1136/bmjopen-2021-057980 |
| Protocol | Yan B, Xu X, Cheung DST, Lin CC. Spiritual and religious interventions for adults with cancer and their carers: an overview of systematic reviews. *Cochrane Database of Systematic Reviews*. 2020;2020(7). doi:10.1002/14651858.CD013675 |
| Poster abstract | Yee J, Renton C, Konings S, et al. Interventions for cancer-related fatigue: A review of systematic reviews. *Asia-Pacific Journal of Clinical Oncology*. 2020;16(SUPPL 8):202. doi:10.1111/ajco.13498 |
| Scoping review | Zhang Y, Zhang Y, Liu S, et al. Acupuncture for cancer pain: a scoping review of systematic reviews and meta-analyses. *Frontiers in oncology*. 2023;13:1169458. doi:10.3389/fonc.2023.1169458 |
