# Supplementary material for: Umbrella Reviews Conducted in an Oncology Healthcare Context Focusing on Supportive Care, Systems, and Models of Care: A Review of Umbrella Reviews
Source: Cancer Med. 2026 Mar 25;15(4):e71708. doi: 10.1002/cam4.71708 (PMC13140849; doi:10.1002/cam4.71708)
Supplement: Supplementary file 4 — Table S4: Recommendations extracted directly from included umbrella reviews. [file CAM4-15-e71708-s004.docx]

Table S4 Recommendations extracted directly from included umbrella reviews

| Author (year)  Review title | Explicit Recommendations (**copy and paste text** here) from discussion, future/recommendations, conclusion sections) |
| --- | --- |
| Abu-Odah et al (2020)  Challenges on the provision of **palliative care** for patients with cancer in **low- and middle-income countries**: a systematic review of reviews | “Investment in terms of time and resources in the training of competent a palliative care workforce is a recommended facilitator in addressing the **workforce shortages**. Also, volunteers can play crucial roles in supporting the health of cancer patients and overcoming workforce shortages”  Therefore, it is recommended that policy makers [in **low- and middle-income** countries] collaborate with national and international organisations to secure funding for **improving health care provision** [in terms of palliative care].  Integrating palliative care into primary care services is a recommended strategy to improve access to palliative care for patients living in remote area.  Future research efforts [in palliative care] are needed to develop a body of evidence that is adequate to support effective learning and policy development. Furthermore, other potential challenges that may hinder the provision of palliative care that have not been covered in this review may form the basis for future studies. For instance, two significant aspects may be considered. The first aspect is the **health care professionals’ voice** [in palliative care]. As most reviews have focused on knowledge, attitudes, and beliefs, none has investigated **communication competencies between patient and healthcare providers and their relationship with their patients**, an important aspect for the successful provision of palliative care. Communicating professionally with patients improves their attitudes [113–115]. The second aspect is the **patients’/family voice** [in palliative care]. Most reviews have examined the attitudes of patients and their families towards palliative care services, but none studied the **priorities, needs, and wishes of patients about palliative care services in low- and middle-income countries**. For policy development, **assessing the country readiness for the provision and integration of palliative care i**s an essential step to an effective adoption. |
| Adam (2015)  **Educational interventions** for **cancer pain**. A systematic review of systematic reviews with nested narrative review of randomized controlled trials | There is a requirement for future research [cancer pain] to identify the **active components of complex interventions**, and to be able to **target interventions to groups** most likely to benefit. **Trials of educational interventions** [relating to cancer pain] should be based upon an **underlying theoretical model** and take account of factors which might influence and modify the effect. In **cancer pain educational interventions**, these **factors would include intervention setting; dose of intervention; patient prognosis; and baseline pain score**s. The **Medical Research Council framework for complex interventions [25] could be used** to guide future projects. It might also be desirable **to** **assess alternative outcome measures**, for example use of out of hours and emergency services. Empowered, educated patients who are able to self-manage may require less emergency care. |
| Amatya et al (2021)  **Rehabilitation** in **patients with lymphoma**: an overview of systematic reviews | “This ICF **(International Classification of Functioning, Disability and Health) model** **can be used as a common framework** to help prioritize personalized goals for PwL (patients with lymphoma), to set rehabilitation criteria.”  “[Lymphoma] Patients with **limited treatment options or persistent frailty** despite rehabilitative attempts **should be offered palliative care** in combination with or, where appropriate, replacing restorative and curative approaches.”  “Future studies [lymphoma] should consider **patient characteristics, outcome measures, timing, mode and intensity of rehabilitation interventions.”** |
| Amiri- Khosroshahi c et al 2022  **Cryotherapy** for **oral mucositis** in cancer: review of systematic reviews and meta-analysis | The **mucosal tissue of children is different from that of adults**, and **more interventions are needed** to provide more conclusive evidence. The paediatric oncology group recommends oral cryotherapy as a preventive intervention for oral mucositis.  Further RCTs are required to generate results that are more clinically reliable. |
| Belloni d (2023)  A systematic review of systematic reviews and pooled meta-analysis on **psychosocial interventions** for improving **cancer-related fatigue** | Although psychosocial interventions have been extensively studied and represent a valuable option for treating specific fatigue dimensions, research is warranted to evaluate the **efficacy of particular interventions within population clusters and examine their long-term effectiveness.** |
| Belloni b (2023)  **Non-pharmacologic interventions** for improving **cancer-related fatigue** (CRF): A systematic review of systematic reviews and pooled meta-analysis | In line with our results, the European Society for Medical Oncology **guidelines recommend nonpharmacologic interventions**, **including physical exercise, psychoeducational, and mind-body approaches** as a valid option **for** **reducing cancer related fatigue**, excepting acupuncture because of the related adverse events [112].  While the literature encompasses numerous studies testing the **efficacy of complementary and integrative medicine** **interventions and physical exercise** on cancer patients, additional studies are needed to clarify the **effect of self-management/e-health and educational interventions on** **cancer related fatigue** considering selected populations’ digital health literacy. However, rather than supporting an overall intervention approach, **targeting subgroups of cancer populations**— in terms of **demographic, clinical, and behavioural characteristics**— who best benefit from a specific intervention type might lead to maximizing the effect of the applied intervention. Future research should **focus on testing these interventions on specific cancer population clusters and trajectories**. |
| Belloni a (2021)  Effects from **physical exercise** on reduced **cancer**-**related fatigue**: a systematic review of systematic reviews and meta-analysis | “**Physical activity** recommendations should **be integrated into patients’ experiences** within the context of a patients’ life, recognizing the **impairing effects of cancer treatments,** **home and working life**, and **patient’s physical and psychological needs** [7].”  “However, additional valuable research needs to be conducted to enable an overall synthesis of the **effect of physical exercise on other cancer diagnoses”**  “For this reason, future research should clarify the quality of the evidence regarding the **validity and reliability of the several tools to measure** **cancer-related fatigue** for providing a **theory-grounded base for clinicians and researchers who require determining which domain of fatigue is more susceptible to physical exercise.** The future **critical appraisal of the characteristics of the available tools for measuring cancer-related fatigue** could help to clarify with more precision in which domains cancer-related fatigue could be defined, as currently, there is no consensus about this aspect [17].”  Further research should focus on **frameworks’** **implementation** to deliver tailored interventions. |
| Belloni e (2021)  A systematic review of systematic reviews and pooled meta-analysis on **pharmacological interventions** to improve **cancer-related fatigue** | Further accurate and targeted studies need to be conducted to **specifically address pharmacological interventions for the treatment of CRF (cancer related fatigue)**, considering **individual cancer population clusters** and **direct comparisons between therapeutic options**.  Although no severe adverse events were reported in the current research, and no significant statistical difference was found between patients treated with methylphenidate/dexamphetamine and patients with placebo, the safety of **psychostimulants** still needs to be investigated in future trials for assessing effects of the long-term therapies (Wood et al., 2014). |
| Belloni c (2023)  A Systematic Review of Systematic Reviews and a Pooled Meta-Analysis on **Complementary and Integrative Medicine** for **Improving Cancer-Related Fatigue** | Our findings regarding **nutritional/herbal supplements** are consistent with clinical guidelines on this topic, discouraging the **consumption of acetyl-L-carnitine** during cancer treatments. However, these results require further **in-depth investigations** if we consider that **dietary supplements** are the most **commonly used CIM** (**complementary and integrative medicine)** among patients with cancer during and after cancer treatments.  This study also identified the main current knowledge gaps, given the **lack of focus on specific cancer diseases** and **direct comparisons between concurrent** **complementary and integrative medicine**, to determine the most effective CIM intervention in reducing CRF. For this reason, we recommend **more robust research** to fill these gaps. |
| Bracchiglione 2023  **Systemic oncological treatments** versus **supportive care** for patients with **advanced hepatobiliary cancers**: an overview of systematic reviews | Future primary studies should consider and **explicitly report** other **patient-important outcomes** (such as QoL (quality of life), symptom control and quality of end of life care), in order to **provide useful data for evidence syntheses** and clinical practice guidelines. Future SRs should also consider these outcomes in their **protocols**, **planning to meta-analyse** data from primary studies or **explicitly report evidence gaps in primary research**. |
| Butow (2020)  **Return to work** after a **cancer diagnosis**: a meta-review of reviews and a meta-synthesis of recent qualitative studies | We need data on the **perspectives of all stakeholders** to inform **intervention development**, to ensure we target all influential factors. Thus, future reviews and individual studies should focus specifically on ensuring these perspectives are heard and understood.  Our findings supported Feuerstein’s framework of RTW (return to work) [7], but complemented it by **suggesting a greater focus on cultural considerations**, underemphasised in this framework. Future **interventions should be multi-factorial** and informed by these findings, addressing the diverse range of issues impacting return to work reported by cancer survivors themselves, including **survivors’ personal goals and needs**, **workplace communication**, **culture, policy and resources, and the wider family, culture and societal context.** |
| Casuso‑Holgado (2022)  **Mind–body practices** for cancerrelated **symptoms management**: an overview of systematic reviews including one hundred twentynine metaanalyses | NR |
| Cedenilla Ramón (2023)  **Psychosocial interventions** for the treatment of **cancer-related fatigue**: an umbrella review | The findings demonstrate the need for the publication **of more detailed descriptions of complex interventions**, promoting **methodological strictness** and **transparency in the design** and throughout the trial process.  Given the current state of the evidence, we recommend that researchers **improve quality and reporting**.  Attention should also be given to the **timing of assessment**, the **duration of the intervention** to maximize benefits, **longer follow-up periods** and the **comparison of psychosocial interventions** versus **usual care or attentional controls**. Further evidence is needed from high-quality trials **with large samples** that fully report rigorous methodological characteristics in the design stage and estimate the optimal sample size based on the existing research results, so as to ensure that the conclusions of the research carried out are sufficiently credible.  Additional studies with **homogeneous samples of cancer patients** are needed.  Targeting patients most in need (i.e., those **reporting clinically significant levels of fatigue**) to **eliminate potential floor effects**, would be a helpful approach in future studies.  The **standardized reporting of the parameters of the different programs** would be useful for investigators and would allow the aggregation of findings between the different trials, thus **enabling the design of specific intervention protocols**. |
| Chan c (2023)  **Effectiveness** and implementation of models of **cancer survivorship care**: an overview of systematic reviews | Future research is needed to expand the understanding of **effective models of care in diverse cancer survivor populations** **including paediatric cancer survivors, adolescent and young adult (AYA) survivor groups** [55], **older adults** [56], and **a broader range of cancer types** as well as **advanced stages of the diseas**e. Future studies should also **prioritize robust primary studies to address gaps in the literature** for outcomes in the domains of health promotion, **chronic conditions**, **clinical structure, and decision-making**. Addressing these gaps will help determine effective models of care using accurate measures [57].  Future research must **expand healthcare outcomes beyond the quality of life** and execute additional **robust economic evaluations** for a wider range of models of care to provide evidence for health systems to fund and **promote the transition to alternative models**. Additionally, interventions tested in trials should be **further tested in real-world settings, especially at the population level**.  In addition, the **development of best practice guidelines** including decision trees for selecting the most appropriate model of care for the **local setting** and individual cancer survivor, **implementation guides, and standardized outcomes** for the evaluation would be helpful to advance this field of science and practice. Future research evaluating models of care should be conducted with **clear descriptions of the model of care elements** and characteristics using **validated tools to assess outcomes** and to identify **sustainable and viable alternatives to specialist-led care.** |
| Chan a (2023)  **Patient navigation** across the **cancer care continuum**: An overview of systematic reviews and emerging literature | Future research should dedicate focus toward evaluating the **effectiveness of patient navigation** **in other common cancers**, such as **prostate cancer, lung cancer, and melanoma**; rare cancer types; and **hematologic malignancies**. Furthermore, **cancer stage was rarely reported in the literature**, and the effectiveness of patient navigation interventions **for patients with advanced or metastatic cancers and those in palliative care and end‐of‐life care settings** needs to be explored.151 Third, there is also **a lack of evaluation including solid clinical end points** (such as survival). Although such clinical end points may be more distal outcomes of navigation, it is important for future evaluations to consider the **inclusion of such end points to further support** the sustainability of such programs.  Future policy research is needed to inform consensus **best‐practice standards** (including **standardized definitions and criteria**) for cancer patient navigation that are specific to the context.  Research into **indigenous populations worldwide** is needed to understand the **unique cultural factors facing indigenous people**, including their pathways to health and well‐being and their access barriers to cancer care. |
| Chan b (2021)  The efficacy, **challenges, and facilitators of telemedicine** in **post-treatment cancer survivorship** care: an overview of systematic reviews | For telemedicine delivery designed to replace **face-to-face consults**, research is needed to establish minimally noninferiority or superiority **compared with usual care.**  Undertake research to explore the **use of telemedicine** to **support chronic disease management**, **medication management**, **cancer screening, surveillance** for recurrence, **and disease prevention**, as well as addressing outcomes across multiple domains of care.  In the assessment of outcomes, **validated tools and objective measures should be prioritized**. Additional outcomes of interest can include knowledge, **self-efficacy, motivation, and adherence**.  Conduct **economic evaluations** to examine potential **cost-effectiveness** or **cost-minimization** following **implementation of telemedicine interventions/services**.  **Develop standards and guidelines** to guide implementors on **the optimal approach to deploy telemedicine**. This includes strategies to engage and identify survivors who will most likely benefit, as well as to **improve accessibility for survivors with different technology** literacy and potential cognitive challenges.  Determine the optimal delivery methods using standardized telemedicine screening/assessing tools, interventions, and outcomes.  Conduct studies to **evaluate the use of different technological platforms** (e.g. application-based versus videoconferencing, or a combination) to provide telemedicine services.  Conduct implementation research studies to **examine and maximize reach, effectiveness, adoption, implementation and maintenance outcomes** of the telemedicine strategies over time.  Expand studies to examine uptake of telemedicine from an equity and disparities perspective through **clearly defined populations covering different cancer** **types, ages, languages, demographic groups, educational levels**, and in **remote, rural, or low-resource settings** over extended periods. |
| Choi (2022)  **Acupuncture** and **moxibustion** for **cancer-related fatigue**: an overview of systematic reviews and meta-analysis | **Cancer related fatigue should be assessed on a regular basis in clinical settings** to aid in the identification of appropriate and **effective therapies, treatments, and management [31].**  However, because this **symptom is becoming more common**, and because it can have a significant impact on a patient’s daily life, **healthcare practitioners should be encouraged** to **inquire about cancer related fatigue**, and to pay attention to its management.  Further research is needed to establish firm evidence and further recommendation. |
| Chung (2015)  Effectiveness of **Chinese herbal medicine** for **cancer palliative care**: overview of systematic reviews with meta-analyses | Future SR should **comply with the PRISMA statement** such that it is **more useable for policy makers and clinicians**.  In the future, **well reported observational studies and RCTs** are needed to clarify the presence of **short and long term toxicities of Chinese herbal medicine**.  To **prevent publication bias**, it is recommended that all **clinical trials protocols** on the topic should **register with a recognized platform** (e.g. the Chinese Clinical Trial Registry).  Future trials are suggested to adopt **more specific QoL (quality of life) measurement tool** such as the Short Form 36 questionnaire, and the European Organisation for Research and Treatment of Cancer Quality of Life Questionnaire-core 30.  Future trials are suggested to investigate the **effectiveness of Chinese herbal medicine in managing common symptoms** like pain, fatigue, anorexia, insomnia, limbs edema and constipation.  To provide **more rigorous evidence** on the effectiveness of Chinese herbal medicine, future SRs and trials must **adhere to high methodological and reporting standards.** |
| Conway (2015)  The **prevention, detection and management** of cancer **treatment-induced cardiotoxicity**: a meta-review | It is recommended that future meta-reviews that **focus on the prevention, detection and management** of **cancer treatment-induced toxicities** should **not include only Cochrane reviews**, as high-quality systematic reviews that **potentially contain unbiased and important recommendations for practice** could be overlooked. |
| Crawford‐Williams (2018)  **Interventions** for **prostate cancer survivorship**: A systematic review of reviews | Future research is needed to **examine the acceptability and effectiveness of exercise** **and psychosocial interventions** in **more diverse populations**, and strategies to support optimal translation into routine clinical practice are needed. In a controlled trial, the **benefits of specific exercise regimes** **compared** with **usual care can be clearly tested**; however, there is limited evidence of these interventions being scaled effectively to reach large sections of the population in real‐world settings.  Future trials also need to **assess how to sustain intervention effects** over a longer **follow‐up period**. |
| Duncan (2017)  Review of systematic reviews of **non-pharmacological interventions** to **improve quality of life** in cancer survivors | There is not much evidence to **support comparative effectiveness of intervention modalities such as** **group versus individual**, **monodimensional versus multidimensional or multidisciplinary**; further work is needed to **examine these different approaches**. Given the **accessibility of social media** and its p**opularit**y, the findings that email contact was related to poorer **quality of life need further investigation**; although interactive websites were beneficial, the overall findings about digital interventions were equivocal.  Future research should consider the **effectiveness of interventions** **targeting people living beyond all types of cancer** and **with poor overall quality of life**. |
| Edbrooke (2023)  **Exercise** across the **lung cancer care** continuum: an overview of systematic reviews | Additional higher-quality research is needed, particularly in **non-surgical populations**, including **subgroup analyses to determine optimal exercise types and settings.** |
| Gkantaifi (2020)  **Honey** against **radiation-induced oral mucositis** in **head and neck cancer** patients. an umbrella review of systematic reviews and meta- analyses of the literature | A comparison between studies could be possible by using **toxicity criteria** applied by each investigator. An **absence of a high level of evidence trials** is observed regarding the **role of honey in oral mucotisis management**. Indeed, although some trials are characterized by adequate randomization and methodology, they lack in blindness. Moreover**, other randomized trials lack in blindness allocation concealment while others do not have adequate data or their samples are too small to give statistical power**. It is necessary that further trials should be conducted, fulfilling all the aforementioned characteristics in order to achieve a high level of evidence. In addition to this, one further interesting issue concerns the **variety of mucositis toxicity scales** that have occasionally been used. A question arises, thus, about the fact that if trials using the **same Oral mucositis toxicity scale were conducted**, **more powerful data** with strong cohesion could be derived. It is noteworthy that if more trials were conducted by setting and subsequently **studying the same criteria**, such as weight loss, pain degree or oral mucositis duration, concerning the participants' quality of life during their treatment, highly valuable data, which could **improve the quality of life,** might arise. |
| Grimmett (2022)  **Psychological interventions** prior to **cancer surgery**: a review of reviews | Researchers are encouraged to use the **template for the intervention** description and **replication (TIDieR) checklist** [65].  Consensus around a **core outcome set to measure** impact on **psychological morbidity** is also required for use in both unimodal **psychological prehabilitation interventions** as well as multimodal approaches. Agreement around **optimal timing of assessments** in relation **to surgery and neoadjuvant/ adjvant therapies** is also warranted.  The **Principles and Guidance for Prehabilitation Within the Management and Support of People with Cancer** [60••] recommends a **triage system** that includes universal, targeted, and **specialist levels of prehabilitation**. This strategy proposes that all healthcare professionals can be equipped to provide “**universal” psychological support**.  We encourage those designing and reporting new clinical trials to **provide sufficient information to replicate their intervention**. Attention should also be given to **ensure new programs** have the **potential to be implemented within clinical practice** with consideration for designing and testing care that is **accessible and has the potential for large-scale implementation**. |
| Hall (2022)  Strategies to **self-manage side-effects** of **adjuvant endocrine therapy among breast cancer survivors**: an umbrella review of empirical evidence and clinical guidelines | Guideline developers should consider describing the **resource implications of applying recommendations** and **suggest audit criteria** to monitor and drive implementation. Furthermore, editorial independence was assessed as being particularly poor for the guidelines identified. **To improve transparency within guideline development**, the views of the **funding body** and **competing interests** of the contributors should be disclosed and easy to identify.  Healthcare professionals should be aware that although the **risk of harm** is unlikely with most of these strategies, the **likelihood of benefit is often unclear**.  There is a need for **high-quality longitudinal primary research** of promising **self-management interventions** for **women with breast cancer using adjuvant endocrine therapy**. Findings from such trials should be **incorporated within clinical guidelines** that have considered how to **implement these strategies into routine practice**. |
| Hou (2023)  Is **physical activity** effective against **cancerrelated fatigue** **in lung cancer** patients? An umbrella review of systematic reviews and metaanalyses | In order to confirm the **effects of resistance exercise**, more **rigorously designed**, **high-quality, large-scale,** randomized controlled **trials will be required** in the future.  Further reviews should focus on **standardization in reporting** and also aim to select randomized controlled trials of **higher quality and lower risk of bias**.  Given the difficulties we encountered in extracting data on **the specific modalities of exercise** (frequency, intensity, duration, and type), future studies should **refine the relevant data to facilitate clinical application**.  In the future, future studies should perform a **long-term follow-up** to **increase exercise intervention utilization** and **application in the clinical setting**.  More **rigorous randomized controlled trials** are recommended for verification in the future.  **Large randomized controlled trials** in patients with **advanced lung cancer** are recommended for verification, so as **to find suitable exercise for** patients with **poor exercise acceptance**.  We offer the following suggestions: first, **strictly controlling bias** when conducting original research; second, retrieve as much **comprehensive information from relevant original research** as possible in the writing of the review; third, **update systematic review in time** when new original research is being published; last but not least, it is recommended to **carry out more diversified original research in different environments**. |
| Huang (2021)  The **effectiveness of tai chi** in patients with **breast cancer**: an overview of systematic reviews and meta-analyses | **Exercise therapy** has been suggested as an **effective and convenient support care** intervention for **patients with breast cancer.**  The **efficacy of Tai chi in the treatment** of breast cancer **requires high-quality studies** to provide more convincing evidence.  We recommend that authors, readers, reviewers, and editors to become more acquainted with and to **more strictly adhere to the AMSTAR-2, PRISMA**, and **GRADE criteria** in future research.  Further rigorous, **comprehensive systematic reviews of meta-analysis and randomized controlled trials** that adhere to the guidelines are **required to provide robust evidence** for definitive conclusions. |
| Jiang (2020)  **Exercise for fatigue in breast cancer** patients: An umbrella review of systematic reviews | Our result indicated that **aerobic exercise is a supportive intervention** on **reducing cancer related fatigue** and the quality of evidence was graded as moderate. These activities are **cost-effectiveness** and **should** **be popularized in clinical practice**.  Hence, **whether Yoga could alleviate fatigue among breast cancer patients** needs **further rigorous randomized controlled trials**.  It has been possible to suggest key components of future exercise interventions for fatigue in breast cancer patients. Firstly, future research is required to **clarify the effectiveness of different intervention modalities** (frequency, intensity, time and type) for breast cancer patients with fatigue to allow **determination of exercise dose-response**. Secondly, it is essential for future **randomized controlled trials and Meta analyses** should **not only focus on the effects of exercise on fatigue** but also how to **improve breast cancer patients’ adherence to exercise protocols**. Thirdly, additional studies are needed to **evaluate the cost-analysis and adverse events of exercise**. Fourthly, there is an urgent need for **innovative methods to generate high-quality evidence.** |
| Amiri Khosroshahi a (2023)  Effect of **probiotic supplementation** on **chemotherapy- and radiotherapy related diarrhoea** in patients with cancer: an umbrella review of systematic reviews and meta-analyses | There is an urgent need for **high-quality randomized controlled trials with a large sample size**, **better study design** and **more complete outcome assessment** to **provide high-quality evidence for the effects of probiotics on chemotherapy and radiotherapy-related diarrhoea** as well as **explore potential subgroup effects.** |
| Amiri Khosroshahi b (2023)  **Nutritional interventions** for the **prevention and treatment** of **cancer therapy-induced oral mucositis**: an umbrella review of systematic reviews and meta-analysis | It is important that **higher-quality** **randomized controlled trials** be conducted for **more conclusive and clinically applicable results** to be generated. |
| Kim (2018)  **Therapeutic options** for **aromatase inhibitor-associated arthralgia in breast cancer survivors**: A systematic review of systematic reviews, evidence mapping, and network meta-analysis | Future studies of **interventions** **aromatase inhibitor-associated arthralgia** for should **include an analysis of adverse events**.  A strategy for **controlling** **aromatase inhibitor-associated arthralgia** should not be focused on estrogen replacement therapy but **on symptom management**.  In conclusion, **acupuncture has been the most frequently tested** **intervention for** **aromatase inhibitor-associated arthralgia** but can only be **recommended cautiously** based on the current evidence.  Further evaluation of the **effects of pharmacologic interventions, aerobic exercise, Nordic walking, omega-3 fatty acids, and vitamin D is necessary**. |
| Knowles (2022)  **Physical activity interventions** in **older people with cancer**: A review of systematic reviews | The review also highlights some notable gaps in evidence including **lower representation of cancers** other than **prostate and patients with comorbidities and sarcopenia** that should be **prioritised in future research**.  Future research should focus on **mechanisms underlying physical activity** effectiveness and on **underrepresented populations**. |
| Laidsaar-Powell (2019)  A meta-review of qualitative research on **adult cancer survivors**: current **strengths and evidence gaps** | There are **evidence gaps in** other topics, such as **spirituality, social connections, body image and coping strategies**. This indicates that there may be an opportunity for more focused and thorough reviews on these issues.  There was also a paucity of reviews on a number of other aspects of survivorship, such as **experiences of ongoing symptoms** (including side/late effects), **financial toxicity**, **multi-morbidity, and psychological issues** such as **fear of cancer recurrence, mental health disorders, stigma** and characteristics associated with better adjustment such as resilience. This likely indicates that these topics are currently underresearched or **lack comprehensive synthesis**, and there may be **potential opportunities for novel research** contributions. |
| Lake (2022)  **Effectiveness** of **weight loss interventions** in **breast cancer survivors**: a systematic review of reviews | Further research is required to determine the most effective combination/balance of these components, along with the **optimum duration and intensity**. Furthermore, studies with **long-term follow-up** are essential to assess whether positive impacts from intervention can be maintained in the long term, and crucially, whether or not this translates to a **reduction in the likelihood** of breast cancer **recurrence**. There was a paucity of evidence concerning the potential role of bariatric surgery as an effective weight loss intervention for breast cancer survivors. It is likely that bariatric surgery is key to weight loss for certain groups of patients, and **future research into surgical** rather than simply lifestyle interventions is needed. **Further research is needed** to define the optimum **combination, intensity and duration** of **a multimodal** component **intervention** for breast cancer survivors. |
| Lavasidis (2022)  **Supportive interventions** for **childhood cancer**: An umbrella review of randomized evidence | The absence of **large RCTs** highlights the **need to expand the research** in the field with larger studies and individual patient data meta-analyses, in order to provide the **young cancer patients and survivors** with the **best care** available. In the clinical practice guideline that was included in our review, (Loeffen et al., 2020) the **use of hypnosis** was **strongly recommended** for all **needle procedures**, including **lumbar puncture** and **bone marrow aspiration**.  **A yearly psychosocial assessment** for **childhood cancer survivors** is therefore **recommended for prevention**.  The available scientific literature needs to be augmented and expanded**, including larger RCTs and meta-analyses** of individual patient data, in order to allow better decision-making and provide paediatric cancer patients, survivors and their families with the **best possible quality of life**. |
| Lee a (2023)  **Effectiveness of Cryotherapy** on Cancer Therapy–**Induced Oral Mucositis** | **Cryotherapy** should be initiated **5 to 30 minutes before cancer therapy** to ensure that **blood vessels** in the oral cavity have **sufficient time to constrict**.  Care should be taken to prevent an **oral temperature of less than 18°C** during **cryotherapy.**  **Cryotherapy** should be started **5 minutes before chemotherapy**, with the patient placing **crushed ice** or small pieces of ice in the mouth. Cryotherapy should **not exceed 30 minutes** per session.  Research on the **effectiveness of cryotherapy** in **reducing oral mucositis severity** remains limited; thus, more **clinical trials are warranted**. |
| Lee b (2018)  An overview of systematic reviews: **complementary therapies** for **cancer patients** | Future studies must **treat the risk of methodological bias** with caution. **High-quality systematic review** in which selection of high-quality studies is combined with adequate methodology, are needed to **clarify the true efficacy** of **complementary therapies** for cancer patients. |
| Leslie (2022)  **Web-based psychological interventions** for people living with and beyond cancer: meta-review of what works and what does not for **maximizing recruitment, engagement, and efficacy** | First, for a greater number **of fully powered randomized controlled trials**, to enable more **robust conclusions about the efficacy** of **web-based psychosocial oncology interventions**. Second, we recommend that authors of future studies **report study uptake**, engagement, and **study outcomes transparently**, **adhering to CONSORT guidelines**. Third, we recommend the **use of outcome measures** that have been **validated within the target population**, with a preference for measures commonly used in previous research to support a more coherent and robust evidence base. Fourth, we recommend investigating **web-based psychosocial intervention effects** in a **broader range of patient populations**, including understudied national and cultural cohorts and men. Finally, we recommend **interventions** that are **directly targeted at specific diagnostic groups** or support needs, including customizable feedback and features, to encourage greater intervention engagement.  Future research can seek to promote both **intervention uptake and engagement** by addressing **participant anxiety about technology** and perceived time burden.  Future studies including **head-to-head comparisons**, which are fully **powered to conduct subgroup analyses**, are needed to conclusively establish what works best for maximizing recruitment, engagement, and efficacy. |
| Licquirish (2019)  **Tools** to **facilitate communication** during **physician-patient consultations** in cancer care: an overview of systematic reviews | It is important that health services are adequately resourced to respond to the range of clinical and broader patient needs, which can be identified through the **routine use of patient-reported outcomes**. **Question prompt lists** can **increase the number of questions** asked by patients without increasing **consultation length** and may encourage them to reflect and **plan questions** before the **consultation**.  Although we identified a large body of research showing that certain interventions can improve patient-physician communication, there is still a need for more **well-designed RCTs** of **novel interventions**.  We recommend **high-quality trials** of the **effectiveness of survivorship care plans**, the distress thermometer, and decision aids, with physicians as participants, to determine whether these improve patient participation in consultations; promote shared decision making; and improve understanding, satisfaction, and treatment outcomes. **Economic evaluation of the tools** to justify investment from health services is also warranted. Implementation studies are needed to provide evidence about the reach, uptake, fidelity, and scalability of the tools discussed in this overview. |
| Li (2023)  The role of **complementary and alternative medicine** on **cancer-related fatigue** in **adults**: an overview of systematic reviews | **Yoga** (a kind of practice combining stretches, meditation, and breathing to relax the mind and body) is recommended by the **latest international guidelines** such as guidelines set by the National Comprehensive Cancer Network (NCCN), American Society of Clinical Oncology (ASCO),42 and European Society for Medical Oncology (ESMO), to treat **cancer related fatigue** in patients either undergoing **active cancer treatment** or **post-treatment.**  A more **robust conclusion** needs to be further assessed through **well-designed** and **well-conducted clinical trials**.  Larger RCTs are needed to identify **the effects of dietary supplements** on **cancer related fatigue**.  It should be **well-designed in future RCTs** in accordance with previous studies and be considered as **a stratification factor** when analyzing the results in future systematic reviews.  The effects of other **complementary and alternative medicine interventions** on **cancer-related symptoms** warrant further investigation.  Conducting further research with **rigorous methodological designs** and **sufficient sample sizes** is still necessary. |
| Loh (2015)  Methods to improve **rehabilitation** of patients following **breast cancer surgery**: a review of systematic reviews | Studies are also needed on **interventions** to **overcome** these **barriers to exercise** and to ensure adherence to **exercise regimes** to gain its **benefit on cancer recurrences** and for better quality of living during the survivorship phases.  Future occupational studies should investigate the **breadth and depth of rehabilitation** methods (eg, work stamina, tolerance, psychological factors for facilitating work re-entry, work accommodations such as flexibility towards work hours etc) for enabling post-operation survivors to **return to work** in a design that has a control or comparison group (ie, other types of interventions, wait-list, etc).  Future studies **with higher methodological rigor** should be conducted on **health promotion strategies** to enable healthy lifestyle. |
| Mazzocco (2023)  Evidence for choosing **qigong** as an **integrated intervention in cancer care**: an umbrella review | The difference between **objective and subjective measures** should be **further investigated** to understand whether **patients’ psychological interpretations** of their conditions might have affected their perceptions of any effects.  Given the evidence summarized above and bearing in mind the need for **high-quality studies** with **larger sample sizes**, a definitive, univocal decision is challenging to make on whether, when, and **how Qigong can be prescribed** to cancer patients.  It is paramount to **improve the quality of future clinical trials** on the matter by planning **longer follow-up times**, including **measures of variables** able to reflect cancer’s multifactorial nature and **Qigong’s core functioning**, and identifying **more objective measurements**, such as **the systematic investigation of** **biological markers**. |
| Mokhtari-Hessari (2020)  Healthrelated **quality of life** **in breast cancer patients**: review of reviews from 2008 to 2018 | Perhaps this is an area that could be addressed independently since there are **differences in quality of life** between **newly diagnosed breast cancer patients**, patients who are receiving different treatments, and **the long-term survivors** who successfully completed their treatments and now they have back to normal life.  It is important to notice that this review of reviews **did not separate the interventional studies** from other types of studies (usually descriptive or correlational). Perhaps a better organization might be to **reporting reviews** **based on separate objectives**. |
| Olsson Möller (2019)  A comprehensive approach to **rehabilitation interventions** following **breast cancer treatment** - a systematic review of systematic reviews | Taking the next step from evaluating the **effect of narrow rehabilitation** studies on specific outcomes, **helping health care professionals** by identifying a knowledge base that could be used to enable **individualization in clinical practice**, requires a more **comprehensive approach** to **individualized rehabilitation**.  To establish a systematic way **of providing individualized rehabilitation**, further research is warranted **to bridge the gap** between rehabilitation research and clinical practice.  There is still a gap between **rehabilitation research** and **practice** which emphasis further research focusing on **dissemination and implementation** of available research findings. |
| Pedro (2021)  Evidence of **psychological and biological effects** of structured **Mindfulness‐Based Interventions** for **cancer patients** and survivors: A meta‐review | A further step on research in this field would be to test **the effectiveness** of **Mindfulness‐Based Stress Reduction /Mindfulness‐Based Cognitive Therapy/Mindfulness‐Based Cancer Recovery** on **different populations** to identify those who would **benefit more and how**, exploring the moderators. Furthermore, it would be interesting to determine if **Mindfulness‐Based Stress Reduction/Mindfulness‐Based Cognitive Therapy/Mindfulness‐Based Cancer Recovery** effects are clinically relevant, whether an **improvement in depression or anxiety or telomerase activity** is clinically significant, affecting health status.  Future studies should focus on **developing high‐quality studies** with **longer follow‐ups**, exploring the moderating effects that might contribute to **biased results**, and on **who might benefit** more from **Mindfulness‐Based Stress Reduction/Mindfulness‐Based Cognitive Therapy/Mindfulness‐Based Cancer Recovery** interventions. |
| Petrigna (2023)  Methodological consideration for **a physical activity intervention** in **breast cancer population**: an umbrella review | Considering all these limitations it was only possible to propose exercise training recommendations for future studies that can be adopted only in a second stage, after a **personalized intervention** planned to reach the minimum level of **physical activity**. Our exercise training recommendations **for breast cancer** patients was based on the main results and the **training protocols** adopted of the included studies considering the benefits of each intervention proposal.  Future studies should focalize the attention, with original researches, on the level of physical activity adapted to the breast cancer patients. Information should be collected before, **during and after the treatment** to present **data** **that can be personalized** according to the **patient’s necessities.**  An intervention should be composed of **combined training** **(aerobic and resistance training)** with a component of a **mindfulness intervention**, with an intensity from moderate to high, and 3 times a week. The intervention should be supervised in the first period and then it could be home-based. **Exercise** **training** should be **personalized** to the patients treated. |
| Qiu (2023)  **Promoting** **physical activity** among **cancer survivors**: an umbrella review of systematic reviews | At the same time, the importance of **personal contact components** was also found in this umbrella review, but the **frequency of assessment** or feedback in **exercise intervention** needs **further study**.  The **details of the intervention** still need further research, including the **effect of duration and frequency** on **patients’ physical activity levels**.  Faced with **different groups of patients**, such as overweight, sedentary behavior, the elderly, and limited physical activity, **appropriate interventions** should be adopted according to the **characteristics of the population**.  Future research may benefit cancer survivors by more **comprehensively applying electronic**, **wearable health technology-based**, **behavior change techniques** (BCTs), and **theory-based interventions**. |
| Rapti (2023)  **Effects of exercise** and **physical activity levels** on **childhood cance**r: an umbrella review | We suggest considering the outcomes of the current umbrella review in everyday clinical practice; however, these **should be treated with caution** when reported by systematic reviews of critically low quality. Future research is needed to **focus on high-quality trials with long-term follow-up** and the o**ptimal type of exercise**, in addition to the duration of the exercise intervention. Equally important is the **determination of the optimum duration and intensity of exercise sessions**. The above characteristics are necessary to be determined in order for **exercise and physical activity guidelines for this population to be formed.** National or international **multicenter studies should be strongly encouraged** for this purpose. To achieve the above, **doctors and families should become familiar with the beneficial effects of exercise/physical activity programs** and their safe nature at all therapy phases. |
| Ricetti (2021)  **Migrants and ethnic minorities** with cancer: an umbrella review on their information and supportive care needs | **Leaflets and brochures should be provided in the language of the patients or survivors affected by cancer**, as only **providing languages spoken by the majority population was perceived as a barrier**, as reported by Alananzeh et al. with respect to Arabic migrant cancer patients [3].  Moreover, the **information and supportive care needs of migrants and ethnic minority cancer patients and survivors in Europe** should be **investigated in detail in future research**.  Providers of **supportive care in cancer should name concrete personnel** who are **responsible for the dissemination of specific information needs**.  **Information needs regarding sensitive topics of body/image and sexuality should be investigated with caution** but not be avoided**. Indirect ways to inform on these topics could help to fulfil the information needs in this domain.**  As **differences in information and supportive care needs** are present **between and within migrant and ethnic minority cancer patients and survivors**, **medical personnel should never overlook the individual characteristics of a patient**. |
| King (2023)  **Psychosocial experiences** of **breast cancer survivors**: a meta-review | **Lifestyle changes are recommended to reduce risk of cardiotoxicity and osteoporosis** including **addressing tobacco and alcohol use, managing weight, and increasing exercise.**  Future studies could **incorporate a wider range of research types**.  The quality of research within this meta-review was moderate, and **future studies should make use of methodological quality guidelines** when conducting systematic reviews and primary research.  To address gaps in the breast cancer research, future **studies should include breast cancer survivors with BRCA1/2 gene mutations**, **women receiving tailored treatments, women from low socioeconomic background**s, breast cancer survivors with **multimorbidity and complex health care needs, late effects, as well as interventions targeting gender and sexually diverse breast cancer survivors.** |
| Schroter (2023)  **Osteoradionecrosis treatment in head and neck cancer patients**: An overview of systematic reviews | More **robust clinical studies are needed** to establish the best treatment for ORN.  The **treatment choice** should be **based on stage and patient’s clinical osteoradionecrosis condition**. |
| Slev (2016)  **Effects of eHealth** for patients and **informal caregivers confronted with cancer**: A meta-review | In future research (both at the level of separate intervention studies and the level of systematic reviews), **more attention should be given to the effects of eHealth interventions in relation to the disease stage.**  The fact that the **Comprehensive Health Enhancement Support System for breast cancer patients is the most researched** eHealth intervention among the available eHealth interventions. This is all the more reason why **future research should concentrate on specific tumor types**.  **More tailored eHealth interventions may yield stronger effects**. However, **more research is needed to confirm this hypothesis**.  Recommendation to focus **future research on the identified lacunas and separately study different types of eHealth interventions, like single-component and multi-component eHealth interventions.** |
| Specchia (2020)  The **impact of tumor board on cancer care**: evidence from an umbrella review | To **make tumor boards effective, professionals should consider them a critical part of their working** **agenda** and save time to prepare and attend tumor boards. Moreover, **congruent time should be dedicated to the meetings** in order to avoid discussing many cases in a short amount of time. Additionally, the **time for acting on decisions made during the tumor boards should be taken into account** (Lamb et al. 2011).  To be addressed, **all these points require a cultural change concerning the way clinicians and other health professionals** understand their practice. Some changes have been made towards this and **several of the latest guidelines recommend the Multidisciplinary Team (MDT) approach in order to provide better cancer care** [15–17]. |
| Tuominen (2018)  **Effectiveness of nursing interventions** among patients with cancer: An overview of systematic reviews | Future research should focus on **interventions that save resources and are relatively easy to implement in daily practice**. **Large‐scale longitudinal research with follow‐up measures is needed to identify long‐term effects,** especially on the lives of patients who have **survived cancer or live with chronic illness**.  To improve the effectiveness, authors recommended considering the individual characteristics of the participants, such as educational level, disease stage and treatment trajectory, when administering EW interventions to patients with cancer.  Future investigation needs **to focus on the most beneficial intervention components to guide clinical practice development**.  **Implementation of these nursing interventions into clinical practice** is important to improve patients’ knowledge and quality of life as well as **reducing various symptoms and side effects related to cancer and its treatment.** |
| TriguerosMurillo (2023)  Effects of **musicbased interventions** on **cancerrelated pain, fatigue, and distress**: an overview of systematic reviews | Finally, most systematic reviews did not clarify whether ‘**standard’ or ‘usual’ care** included supportive cancer care, as **a paradigm for modern treatment** in oncology [72], to manage the **physical, psychological, social, and spiritual needs of patients** [73], or specific cancer treatments such as chemotherapy. This needs to be **clarified in future systematic reviews**. |
| Tune (2022)  How Is **Quality of mHealth Interventions** for **Cancer Survivors** Defined and Described? An Umbrella Review | Our recommendation is that **mHealth technologies for cancer survivors** implement industry **standard data encryption to ensure the security of private information.**  **More research** is recommended in how quality is defined in **mHealth among cancer survivors**. Further research may help propel the development of a **comprehensive quality framework** that allows for **consistent evaluation of mHealth technologies in cancer survivors**. |
| Wang (2022)  The **effectiveness of case management** for cancer patients: an umbrella review | Indicating **a more rigorous design** **and evaluation** is needed **to avoid this information bias. (not blinding)**  It is well-needed to report how those **case management intervention** were conducted **follow standard reporting guidelines**, in order to provide recommendation for future research.  Future research should **clearly describe details of case management intervention** and its implementation, **including theoretical underpinnings, dose and intensity, interventionist qualifications, protocol or manual used, fidelity**, etc. In that way these details can be included in future systematic reviews, and **effectiveness of individual elements of the intervention can be examined** [27]. We recommend **use standard guidelines to help organize** the case management intervention reporting. For example, the Template for Intervention Description and Replication (TIDeiR) is one of the most popular guidelines that could be used to report the full breadth of case management interventions: from intervention rationale to assessments of treatment adherence and fidelity [42]. 2) **More rigorous trials are needed to evaluate the effectiveness** of case management. 3) Studies should also **explore the barriers to and facilitators of case management implementation** across various types of cancer patients at different stages, **providing evidence for conducting successful case management implementation in the future.** |
| Wu a (2015)  **Effectiveness of acupuncture** and **related therapies for palliative care** of cancer: overview of systematic reviews | Future confirmatory **trials should adhere to CONSORT recommendations for reporting**. Researchers should also **adopt a comparative effectiveness approach and design trials** **that allow real-world evaluation of acupuncture and related therapies**.  Specifically, the **combined effects of acupuncture and related therapies** in addition to **guideline-recommended conventional care** (e.g., glucocorticoids, 5-HT3 antagonists and/or NK1R antagonists in CINV) should be compared with conventional care alone so that the **additional benefits of acupuncture can be elucidated.**  Future trials should **choose the most clinically relevant endpoint as the primary outcome** and **measure it using a validated method**11 so as to **ensure the utility of future clinical evidence.**  **Future comparative effectiveness research in this area should pay attention** **to improving the reporting and methodological quality of trials**.  **Describing the treatment protocol according to the STRICTA51 and TIDieR checklist52**, so that the **procedure can be replicated in other trials or be adopted into clinical practice** if it is found to be effective.  And **choosing guideline-recommended treatment** for the **control group and validating outcome measures**. |
| Xing (2023)  **Effectiveness of manual lymphatic drainage** for **breast cancer-related lymphoedema**: an overview of systematic reviews and meta-analyses | **More studies on measurement instruments should be explored**, with attention to **early detection of lymphedema flow, convenient clinical application, and accurate measuring.**  More **well designed and large RCTs are needed** to provide **a higher level of evidence to confirm the role of manual lymphatic drainage in complete decongestive therapy**, especially for **patients under 60 years of age or with 1 month intervention duration.** |
| Wu b (2016)  **Chinese herbal medicine** for **improving quality of life among nonsmall cell lung cancer patients** | Methodological limitations of RCTs have limited the trustworthiness of these conclusions and future RCTs should address the following: use a **Chinese herbal medicine placebo in the control group**; ensure **blinding of outcome assessment**; **measuring patient outcomes comprehensively using validated scales**; report **trial implementation and results according to the CONSORT statement**; and **reducing publication bias by releasing RCT protocols on trial registries**. |
| Bao (2014)  **Complementary and alternative medicine** for **cancer pain**: an overview of systematic reviews | In the future, in order to prescribe, complementary and alternative medicine the **health care professionals should be more careful**. **The methodological quality for primary studies was low and their sample size was small**, so in the future **large and well-designed RCTs should be conducted** to confirm the conclusions of available systematic reviews.  The key methodological aspects, such as **methods of randomization**, **concealed allocation, and blinding**, should **be well conducted and reported**. |
| Zanghi (2022)  The practice of **physical activity on psychological, mental, physical, and social wellbeing** for **breast-cancer survivors**: an umbrella review | Future studies should focalize attention on **the different effects of physical activity on breast cancer patients** **under therapy or under other conditions**. |
| Zhang a (2022)  **Acupuncture for cancer-related conditions**: An overview of systematic reviews | Clinical studies should be recommended on conditions commonly used acupuncture in clinic, but **lacking high-quality or well-reported evidence**, **like cancer-related depression and anxiety, breast cancer related arthralgia, radiation-induced xerostomia, and dysphagia**. Clinical trials are **strongly recommended to report by CONSORT Statement** and its extension to acupuncture trials (STRICTA) (Hughes et al., 2019) to keep a high methodological quality, and no more low-quality, insufficient reported studies (Anshasi and Ahmad, 2021).  For some conditions, there seems plenty of RCTs published **but the reviews did not included all eligible evidence or only old evidence included**, for instance, **cancer-related depression and anxiety, gastrointestinal dysfunction, which will be worth conducting a new SR**.  **According to the Preferred Reporting Items for Systematic reviews and Meta-Analyses (PRISMA)** (Page et al., 2021), it is recommended to **register protocol before conducting, provide exclusion list if possible, and report by acknowledged criteria.**  Future reviews are recommended to **report according to the acknowledged reporting standards** **to improve the quality of evidence**. |
| Zhang b (2023)  **Effectiveness of exercise interventions** in the **management of cancerrelated fatigue**: a systematic review of systematic reviews | Exercise should be **carefully recommended to avoid complications if the patient is in poor physical condition**, weak, or **has associated post-treatment effects (such as cardiopulmonary restriction, fever, anemia, neutropenia, or thrombocytopenia).**  Future research should **employ the incidence of adverse events as the main evaluation index of the study.** |
| Zhang c (2020)  How can **alternative exercise traditions** help against the **background of the covid-19 in cancer care**? an overview of systematic reviews | The **quality of methodology needs to be further improved**.  In conducting an SR/MA, the **PRISMA statement should be used as basis** in **preparing a normative report to improve the overall report quality**.  In the future, other **physical measurement indicators for evaluating obesity** are expected.  However, whether or not they (**alternative exercise traditions) are more advantageous than conventional rehabilitation training** still **needs to be supported by a large sample size and strict standardized trials.**  Future research should **incorporate a large number of samples (in the baseline and follow-up phases), long-term follow-up evaluations** (eg, 6 months or more), and **clearly defined targeted measurement indicators into the design**.  We suggest that focus should be directed to the **improvement of the preliminary design scheme, publication status, literature retrieval, conflict of interest, and other aspects.** |
| Zhao (2023)  The **effectiveness of exercise** on the **symptoms in breast cancer patients undergoing adjuvant treatment**: an umbrella review of systematic reviews and meta-analyses | Several symptoms brought on **by adjuvant treatment for breast cancer can be alleviated with exercise**, but **this benefit needs to be further demonstrated**. First, future studies must **increase the sample size**. Second, although a **growing number of recent studies** have explored changes in symptom clusters or symptom networks over time during breast cancer adjuvant treatment, their clinical practice is inadequate. The complexity of the symptoms is one potential cause. To solve this problem, future research **may use ecological transient assessment to dynamically assess symptoms**. Thirdly, we found that there is still a **lack of research on the effects of exercise on relieving symptom clusters** or **symptom networks during adjuvant therapy in breast cancer patients**. We expect that further research will be conducted to **examine the effects of exercise on improving bridge symptoms** identified within or between symptom clusters and, in addition, **advance the development of symptomics using symptom network analysis**. This will improve the efficiency of symptom management and better meet the specific needs of more patients with breast cancer. |
| Zhou a (2022)  **Effects of exercise interventions** on **cancerrelated fatigue in breast cancer** patients: an overview of systematic reviews | For exercise type, the **study findings suggested that aerobic exercise and yoga were commonly recommended** as promising approaches to improving cancer related fatigue.  For future studies, **a list of excluded studies should be provided** **as an independent appendix** to journals **to facilitate readers’ understanding of the data selection process** and further improve the reliability of the review findings.  **Funding sources should be clearly declared** in future publications to **help readers determine whether funding bias existed**. To achieve a comprehensive literature search, future systematic reviews are suggested to identify **potential studies by searching not only the commonly used databases but also gray literature retrieval websites to minimize publication bias.**  In order to further improve the level of evidence of the included SRs, **more original studies with rigorous study designs and detailed descriptions of the intervention protocols** (e.g., type, frequency, intensity, and duration of the exercise) are necessary.  **More rigorously designed clinical studies are needed** to specify the exact **exercise type, duration, frequency, and intensity to have an optimal effect on cancer related fatigue in breast cancer patients.** |
| Zhou b (2020)  **Effects of perioperative exercise interventions** on **lung cancer patients**: An overview of systematic reviews | **Rigorous RCTs and systematic reviews are needed** to provide **high-quality evidence for the specificity of exercise interventions**, to more clearly delineate the specific effects of each type of exercise and to **establish the appropriate volume for each type of exercise, with the goal of optimising outcomes for surgical lung cancer patients.**  More high-quality research is required, to **evaluate the effects of different types and amounts of exercises on health outcomes for surgical lung cancer patients.** |

| “Investment in terms of time and resources in the training of competent a palliative care workforce is a recommended facilitator in addressing the **workforce shortages**. Also, volunteers can play crucial roles in supporting the health of cancer patients and overcoming workforce shortages”  Therefore, it is recommended that policy makers [in **low- and middle-income** countries] collaborate with national and international organisations to secure funding for **improving health care provision** [in terms of palliative care].  Integrating palliative care into primary care services is a recommended strategy to improve access to palliative care for patients living in remote area.  Future research efforts [in palliative care] are needed to develop a body of evidence that is adequate to support effective learning and policy development. Furthermore, other potential challenges that may hinder the provision of palliative care that have not been covered in this review may form the basis for future studies. For instance, two significant aspects may be considered. The first aspect is the **health care professionals’ voice** [in palliative care]. As most reviews have focused on knowledge, attitudes, and beliefs, none has investigated **communication competencies between patient and healthcare providers and their relationship with their patients**, an important aspect for the successful provision of palliative care. Communicating professionally with patients improves their attitudes [113–115]. The second aspect is the **patients’/family voice** [in palliative care]. Most reviews have examined the attitudes of patients and their families towards palliative care services, but none studied the **priorities, needs, and wishes of patients about palliative care services in low- and middle-income countries**. For policy development, **assessing the country readiness for the provision and integration of palliative care i**s an essential step to an effective adoption. | Codes from Abu-Odah et al (2020)  Challenges on the provision of palliative care for patients with cancer in low- and middle-income countries: a systematic review of reviews Investment in terms of time and resources in the training of competent palliative care workforce is a facilitator in addressing the workforce shortages.  Volunteers can play crucial roles in supporting the health of cancer patients and overcoming workforce shortages.  Recommended that policy makers [in **low- and middle-income** countries] collaborate with national and international organisations to secure funding for **improving health care provision** [in terms of palliative care].  Integrating palliative care into primary care services is a recommended strategy to improve access to palliative care for patients living in remote area.  Future research efforts [in palliative care] are needed to develop a body of evidence that is adequate to support effective learning and policy development.  Re **health care professionals’ voice** [in palliative care]---need to investigate **communication competencies between patient and healthcare providers and their relationship with their patients in** palliative care.  Re **patients’/family voice** [in palliative care] need to address the **priorities, needs, and wishes of patients about palliative care services in low- and middle-income countries**.  For policy development, **assessing the country readiness for the provision and integration of palliative care i**s an essential step to an effective adoption [of palliative care]. |
| --- | --- |
| “This ICF **(International Classification of Functioning, Disability and Health) model** **can be used as a common framework** to help prioritize personalized goals for PwL (patients with lymphoma), to set rehabilitation criteria.”  “[Lymphoma] Patients with **limited treatment options or persistent frailty** despite rehabilitative attempts **should be offered palliative care** in combination with or, where appropriate, replacing restorative and curative approaches.”  “Future studies [lymphoma] should consider **patient characteristics, outcome measures, timing, mode and intensity of rehabilitation interventions.”** | Amatya et al (2021) codes  The **International Classification of Functioning, Disability and Health model** **can be used as a common framework** to help prioritize personalized goals for patients with lymphoma to set rehabilitation criteria.  [Lymphoma] Patients with **limited treatment options or persistent frailty** despite rehabilitative attempts **should be offered palliative care** in combination with or, where appropriate, replacing restorative and curative approaches.  Future studies [lymphoma] should consider **patient characteristics, outcome measures, timing, mode and intensity of rehabilitation interventions.** |
| There is a requirement for future research [cancer pain] to identify the **active components of complex interventions**, and to be able to **target interventions to groups** most likely to benefit. **Trials of educational interventions** [relating to cancer pain] should be based upon an **underlying theoretical model** and take account of factors which might influence and modify the effect. In **cancer pain educational interventions**, these **factors would include intervention setting; dose of intervention; patient prognosis; and baseline pain score**s. The **Medical Research Council framework for complex interventions [25] could be used** to guide future projects. Studies of HCP (patient and healthcare providers) education should include a patient outcome. None of the published reviews of cancer pain education to date have considered **cost effectiveness.** It might also be desirable **to assess alternative outcome measures**, for example use of out of hours and emergency services. Empowered, educated patients who are able to self-manage may require less emergency care. | Adam (2015) codes  **Educational interventions for cancer pain. A systematic review of systematic reviews with nested narrative review of randomized controlled trials**  Future research should [cancer pain] identify the **active components of complex interventions**, and to be able to **target interventions to groups** most likely to benefit.  **Trials of educational interventions** [relating to cancer pain] should be based on **underlying theoretical model** and consider factors which might influence and modify the effect.  The **Medical Research Council framework for complex interventions could be used** to guide future projects.  Studies of HCP (patient and healthcare providers) education should include a patient outcome.  It might also be desirable **to assess alternative outcome measures**, for example use of out of hours and emergency services. |
| The **mucosal tissue of children is different from that of adults**, and **more interventions are needed** to provide more conclusive evidence. The paediatric oncology group recommends oral cryotherapy as a preventive intervention for oral mucositis.  Further RCTs are required to generate results that are more clinically reliable. | Amiri- Khosroshahi c et al 2022  The **mucosal tissue of children is different from that of adults**, and **more interventions (regarding cryotherapy) are needed** to provide more conclusive evidence.  Further RCTs are required to generate results that are more clinically reliable. |
| Although psychosocial interventions have been extensively studied and represent a valuable option for treating specific fatigue dimensions, research is warranted to evaluate the **efficacy of particular interventions within population clusters and examine their long-term effectiveness.** | Belloni d (2023)  A systematic review of systematic reviews and pooled meta-analysis on **psychosocial interventions** for improving **cancer-related fatigue**  Research is warranted to evaluate the **efficacy of particular interventions [psychosocial interventions] within population clusters and examine their long-term effectiveness.** |
| In line with our results, the European Society for Medical Oncology **guidelines recommend nonpharmacologic interventions**, **including physical exercise, psychoeducational, and mind-body approaches** as a valid option **for reducing cancer related fatigue**, excepting acupuncture because of the related adverse events [112].  While the literature encompasses numerous studies testing the **efficacy of complementary and integrative medicine** **interventions and physical exercise** on cancer patients, additional studies are needed to clarify the **effect of self-management/e-health and educational interventions on** **cancer related fatigue** considering selected populations’ digital health literacy. However, rather than supporting an overall intervention approach, **targeting subgroups of cancer populations**— in terms of **demographic, clinical, and behavioural characteristics**— who best benefit from a specific intervention type might lead to maximizing the effect of the applied intervention. Future research should **focus on testing these interventions on specific cancer population clusters and trajectories**. | Belloni b (2023)  **Non-pharmacologic interventions** for improving **cancer-related fatigue** (CRF): A systematic review of systematic reviews and pooled meta-analysis  Additional studies are needed to clarify the **effect of self-management/e-health and educational interventions on** **cancer related fatigue** considering selected populations’ digital health literacy.  However, rather than supporting an overall intervention approach, **targeting subgroups of cancer populations**— in terms of **demographic, clinical, and behavioural characteristics**— who best benefit from a specific intervention type might lead to maximizing the effect of the applied intervention.  Future research should **focus on testing these interventions on specific cancer population clusters and trajectories**. |
| **Physical activity** recommendations should **be integrated into patients’ experiences** within the context of a patients’ life, recognizing the **impairing effects of cancer treatments,** **home and working life**, and **patient’s physical and psychological needs** [7].  However, additional valuable research needs to be conducted to enable an overall synthesis of the **effect of physical exercise on other cancer diagnoses.**  “For this reason, future research should clarify the quality of the evidence regarding the **validity and reliability of the several tools to measure** **cancer-related fatigue** for providing a **theory-grounded base for clinicians and researchers who require determining which domain of fatigue is more susceptible to physical exercise.** The future **critical appraisal of the characteristics of the available tools for measuring cancer-related fatigue** could help to clarify with more precision in which domains cancer-related fatigue could be defined, as currently, there is no consensus about this aspect [17].”  Further research should focus on **frameworks’** **implementation** to deliver tailored interventions. | Belloni a (2021)  Effects from **physical exercise** on reduced **cancer**-**related fatigue**: a systematic review of systematic reviews and meta-analysis  **Physical activity** recommendations should **be integrated into patients’ experiences** within the context of a patients’ life, recognizing the **impairing effects of cancer treatments,** **home and working life**, and **patient’s physical and psychological needs**.  Further research needs to be conducted to enable an overall synthesis of the **effect of physical exercise on other cancer diagnoses.**  Future research should clarify the quality of the evidence regarding the **validity and reliability of the several tools to measure** **cancer-related fatigue** for providing a **theory-grounded base for clinicians and researchers.**  The future **critical appraisal of the characteristics of the available tools for measuring cancer-related fatigue** could help to clarify domains cancer-related fatigue could be defined.  Further research should focus on **frameworks’** **implementation** to deliver tailored interventions. |
| Further accurate and targeted studies need to be conducted to **specifically address pharmacological interventions for the treatment of CRF (cancer related fatigue)**, considering **individual cancer population clusters** and **direct comparisons between therapeutic options**.  Although no severe adverse events were reported in the current research, and no significant statistical difference was found between patients treated with methylphenidate/dexamphetamine and patients with placebo, the safety of **psychostimulants** still needs to be investigated in future trials for assessing effects of the long-term therapies (Wood et al., 2014). | Belloni e (2021)  A systematic review of systematic reviews and pooled meta-analysis on **pharmacological interventions** to improve **cancer-related fatigue**  Further studies need to be conducted to **specifically address pharmacological interventions for the treatment of cancer related fatigue.**  Future research should consider **individual cancer population clusters** and **direct comparisons between therapeutic options**.  The safety of **psychostimulants** still needs to be investigated in future trials for assessing effects of the long-term therapies. |
| Our findings regarding **nutritional/herbal supplements** are consistent with clinical guidelines on this topic, discouraging the **consumption of acetyl-L-carnitine** during cancer treatments. However, these results require further **in-depth investigations** if we consider that **dietary supplements** are the most **commonly used CIM** (**complementary and integrative medicine)** among patients with cancer during and after cancer treatments.  This study also identified the main current knowledge gaps, given the **lack of focus on specific cancer diseases** and **direct comparisons between concurrent** **complementary and integrative medicine**, to determine the most effective CIM intervention in reducing CRF. For this reason, we recommend **more robust research** to fill these gaps. | Belloni c (2023)  A Systematic Review of Systematic Reviews and a Pooled Meta-Analysis on **Complementary and Integrative Medicine** for **Improving Cancer-Related Fatigue**  The most **commonly used complementary and integrative medicine intervention,** dietary supplements, requires further in-depth investigations.  We recommend more robust research to emphasis the lack of focus on specific cancer diseases and to facilitate direct comparisons between concurrent complementary and integrative medicine interventions. |
| Future primary studies should consider and **explicitly report** other **patient-important outcomes** (such as QoL (quality of life), symptom control and quality of end of life care), in order to **provide useful data for evidence syntheses** and clinical practice guidelines. Future SRs should also consider these outcomes in their **protocols**, **planning to meta-analyse** data from primary studies or **explicitly report evidence gaps in primary research**. | Bracchiglione 2023  **Systemic oncological treatments** versus **supportive care** for patients with **advanced hepatobiliary cancers**: an overview of systematic reviews  Future primary studies should consider and explicitly report patient-important outcomes [quality of life, symptom control]**,** in order to **provide useful data for evidence syntheses** and **clinical practice guidelines**.  Future systematic reviews should also consider these outcomes [quality of life, symptom control] in their **protocols**, **planning to meta-analyse** data from primary studies or **explicitly report evidence gaps in primary research**. |
| We need data on the **perspectives of all stakeholders** to inform **intervention development**, to ensure we target all influential factors. Thus, future reviews and individual studies should focus specifically on ensuring these perspectives are heard and understood.  Our findings supported Feuerstein’s framework of return to work [7], but complemented it by **suggesting a greater focus on cultural considerations**, underemphasised in this framework. Future **interventions should be multi-factorial** and informed by these findings, addressing the diverse range of issues impacting return to work reported by cancer survivors themselves, including **survivors’ personal goals and needs**, **workplace communication**, **culture, policy and resources, and the wider family, culture and societal context.** | Butow (2020)  **Return to work** after a **cancer diagnosis**: a meta-review of reviews and a meta-synthesis of recent qualitative studies  We need data on the **perspectives of all stakeholders** to inform **intervention development**, to ensure we target all influential factors. Thus, future reviews and individual studies should focus specifically on ensuring these perspectives are heard and understood.  Data is needed on the **perspectives of all stakeholders [regarding return to work after diagnosis]** to inform **intervention development.**  Future **interventions should be multi-factorial,** and addressing the diverse range of components, including **survivors’ personal goals and needs**, **workplace communication**, **culture, policy and resources, and the wider family, culture and societal context.** |
| NR | Casuso‑Holgado (2022)  **Mind–body practices** for cancerrelated **symptoms management**: an overview of systematic reviews including one hundred twentynine metaanalyses  **NR** |
| The findings demonstrate the need for the publication **of more detailed descriptions of complex interventions**, promoting **methodological strictness** and **transparency in the design** and throughout the trial process.  Given the current state of the evidence, we recommend that researchers **improve quality and reporting**.  Attention should also be given to the **timing of assessment**, the **duration of the intervention** to maximize benefits, **longer follow-up periods** and the **comparison of psychosocial interventions** versus **usual care or attentional controls**. Further evidence is needed from high-quality trials **with large samples** that fully report rigorous methodological characteristics in the design stage and estimate the optimal sample size based on the existing research results, so as to ensure that the conclusions of the research carried out are sufficiently credible.  Additional studies with **homogeneous samples of cancer patients** are needed.  The **standardized reporting of the parameters of the different programs** would be useful for investigators and would allow the aggregation of findings between the different trials, thus **enabling the design of specific intervention protocols**. | Cedenilla Ramón (2023)  **Psychosocial interventions** for the treatment of **cancer-related fatigue**: an umbrella review  There is a need for the publication **of more detailed descriptions of complex interventions**.  Future trials need to transparent in the design and promote methodological strictness throughout the trial process.  Recommended that researchers **improve quality and reporting**.  **Timing of assessment**, the **duration of the intervention**, and **longer follow-up periods** should address detailed in further research.    Further evidence is needed from high-quality trials with large samples that fully report rigorous methodological characteristics in the design stage.  Future research is needed that from high-quality trials estimate the optimal sample size based on the existing research results, aiming to ensure that the conclusions drawn from the research are valid.  More studies with **homogeneous samples of cancer patients** are needed.  The **standardized reporting of the parameters of the different programs are** needed to **enable the design of specific intervention protocols**. |
| Future research is needed to expand the understanding of **effective models of care in diverse cancer survivor populations** **including paediatric cancer survivors, adolescent and young adult (AYA) survivor groups** [55], **older adults** [56], and **a broader range of cancer types** as well as **advanced stages of the diseas**e. Future studies should also **prioritize robust primary studies to address gaps in the literature** for outcomes in the domains of health promotion, **chronic conditions**, **clinical structure, and decision-making**. Addressing these gaps will help determine effective models of care using accurate measures [57].  Future research must **expand healthcare outcomes beyond the quality of life** and execute additional **robust economic evaluations** for a wider range of models of care to provide evidence for health systems to fund and **promote the transition to alternative models**. Additionally, interventions tested in trials should be **further tested in real-world settings, especially at the population level**.  In addition, the **development of best practice guidelines** including decision trees for selecting the most appropriate model of care for the **local setting** and individual cancer survivor, **implementation guides, and standardized outcomes** for the evaluation would be helpful to advance this field of science and practice. Future research evaluating models of care should be conducted with **clear descriptions of the model of care elements** and characteristics using **validated tools to assess outcomes** and to identify **sustainable and viable alternatives to specialist-led care.** | Chan c (2023)  **Effectiveness** and implementation of models of **cancer survivorship care**: an overview of systematic reviews  Future research is needed to expand the understanding of **effective models of care in diverse cancer survivor populations** **including paediatric cancer survivors, adolescent and young adult survivor group**, **older adults**, and **a broader range of cancer types** as well as **advanced stages of the diseas**e.    Future studies should **prioritize robust primary studies to address gaps in the literature** for outcomes in the domains of health promotion, **chronic conditions**, **clinical structure, and decision-making**.  Interventions tested in trials should be **further tested in real-world settings, especially at the population level**.  Future research must **expand robust economic evaluations** for a wider range of models of care to provide evidence for health systems to fund and **promote the transition to alternative models.**  In addition, the **development of best practice guidelines** including decision trees for selecting the most appropriate model of care for the **local setting** and individual cancer survivor, **implementation guides, and standardized outcomes** for the evaluation would be helpful to advance this field of science and practice.  Future research evaluating models of care should be conducted with **clear descriptions of the model of care elements** and characteristics using **validated tools to assess outcomes** and to identify **sustainable and viable alternatives to specialist-led care.**  It is recommended that the development of best practice guidelines including decision trees for selecting the most appropriate model of care for the individual cancer survivors, **implementation guides, and standardized outcomes** for the evaluation.  Future research evaluating models of care should be conducted using **validated tools to assess outcomes.** |
| Future research should dedicate focus toward evaluating the **effectiveness of patient navigation** **in other common cancers**, such as **prostate cancer, lung cancer, and melanoma**; rare cancer types; and **hematologic malignancies**. Furthermore, **cancer stage was rarely reported in the literature**, and the effectiveness of patient navigation interventions **for patients with advanced or metastatic cancers and those in palliative care and end‐of‐life care settings** needs to be explored.  Future policy research is needed to inform consensus **best‐practice standards** (including **standardized definitions and criteria**) for cancer patient navigation that are specific to the context.  Research into **indigenous populations worldwide** is needed to understand the **unique cultural factors facing indigenous people**, including their pathways to health and well‐being and their access barriers to cancer care. | Chan a (2023)  **Patient navigation** across the **cancer care continuum**: An overview of systematic reviews and emerging literature  Future research should dedicate the **effectiveness of patient navigation** **in common cancers**, such as **prostate cancer, lung cancer, and melanoma**; rare cancer types; and **hematologic malignancies**.  **The** effectiveness of patient navigation interventions **for patients with advanced or metastatic cancers and those in palliative care and end‐of‐life care settings** needs to be explored.  Future policy research is needed to inform consensus **best‐practice standards,** including **standardized definitions and criteria** for cancer patient navigation.  Research into **indigenous populations worldwide** is needed to understand the **unique cultural factors facing indigenous people**, including their pathways to health and well‐being and their access barriers to cancer care. |
| For telemedicine delivery designed to replace **face-to-face consults**, research is needed to establish minimally noninferiority or superiority **compared with usual care.**  Undertake research to explore the **use of telemedicine** to **support chronic disease management**, **medication management**, **cancer screening, surveillance** for recurrence, **and disease prevention**, as well as addressing outcomes across multiple domains of care.  In the assessment of outcomes, **validated tools and objective measures should be prioritized**. Additional outcomes of interest can include knowledge, **self-efficacy, motivation, and adherence**.  Conduct **economic evaluations** to examine potential **cost-effectiveness** or **cost-minimization** following **implementation of telemedicine interventions/services**.  **Develop standards and guidelines** to guide implementors on **the optimal approach to deploy telemedicine**. This includes strategies to engage and identify survivors who will most likely benefit, as well as to **improve accessibility for survivors with different technology** literacy and potential cognitive challenges.  Determine the optimal delivery methods using standardized telemedicine screening/assessing tools, interventions, and outcomes.  Conduct studies to **evaluate the use of different technological platforms** (e.g. application-based versus videoconferencing, or a combination) to provide telemedicine services.  Conduct implementation research studies to **examine and maximize reach, effectiveness, adoption, implementation and maintenance outcomes** of the telemedicine strategies over time.  Expand studies to examine uptake of telemedicine from an equity and disparities perspective through **clearly defined populations covering different cancer** **types, ages, languages, demographic groups, educational levels**, and in **remote, rural, or low-resource settings** over extended periods. | Chan b (2021)  The efficacy, **challenges, and facilitators of telemedicine** in **post-treatment cancer survivorship** care: an overview of systematic reviews  For telemedicine delivery designed to replace **face-to-face consults**, research is needed to establish minimally noninferiority or superiority **compared with usual care.**  Undertake research to explore the **use of telemedicine** to **support chronic disease management**, **medication management**, **cancer screening, surveillance** for recurrence, **and disease prevention.**  In the assessment of outcomes, **validated tools and objective measures should be prioritized**.  **Economic evaluations** should be conducted to examine potential cost-effectiveness or cost-minimization following **implementation of telemedicine interventions.**  **S****tandards and guidelines** should be developed to guide implementors on **the optimal approach to deploy telemedicine**.  Future research should determine the optimal delivery methods using **standardized telemedicine screening/assessing tools**, **interventions, and outcomes.**  **Evaluating the use of different technological platforms** recommended to **provide telemedicine services.**  Implementation research studies is recommended to examine and **maximize effectiveness, adoption, implementation and maintenance outcomes** of the telemedicine strategies over time.  Future studies should examine the populations covering **different cancer types, ages, languages, demographic groups, educational levels, and in remote, rural, or low-resource settings** over extended periods. |
| **Cancer related fatigue should be assessed on a regular basis in clinical settings** to aid in the identification of appropriate and **effective therapies, treatments, and management [31].**  However, because this **symptom is becoming more common**, and because it can have a significant impact on a patient’s daily life, **healthcare practitioners should be encouraged** to **inquire about cancer related fatigue**, and to pay attention to its management.  Further research is needed to establish firm evidence and further recommendation. | Choi (2022)  **Acupuncture** and **moxibustion** for **cancer-related fatigue**: an overview of systematic reviews and meta-analysis  **Cancer related fatigue** should be assessed on a **regular basis in clinical settings** to aid in the identification of **effective therapies, treatments, and management.**    Further research is needed to **establish firm evidence and further recommendation**. |
| Future SR should **comply with the PRISMA statement** such that it is **more useable for policy makers and clinicians**.  In the future, **well reported observational studies and RCTs** are needed to clarify the presence of **short and long term toxicities of Chinese herbal medicine**.  To **prevent publication bias**, it is recommended that all **clinical trials protocols** on the topic should **register with a recognized platform** (e.g. the Chinese Clinical Trial Registry).  Future trials are suggested to adopt **more specific QoL (quality of life) measurement tool** such as the Short Form 36 questionnaire, and the European Organisation for Research and Treatment of Cancer Quality of Life Questionnaire-core 30.  Future trials are suggested to investigate the **effectiveness of Chinese herbal medicine in managing common symptoms** like pain, fatigue, anorexia, insomnia, limbs edema and constipation.  To provide **more rigorous evidence** on the effectiveness of Chinese herbal medicine, future SRs and trials must **adhere to high methodological and reporting standards.** | Chung (2015)  Effectiveness of **Chinese herbal medicine** for **cancer palliative care**: overview of systematic reviews with meta-analyses  Future systematic review should **comply with the PRISMA statement** such that it is **more useable for policy makers and clinicians**.  **Well reported observational studies and randomised controlled trials** are needed to clarify the presence of **short and long term toxicities of Chinese herbal medicine**.    To **prevent publication bias**, it is recommended that all **clinical trials protocols** on the topic should **register with a recognized platform**.    Future trials are suggested to adopt **more specific quality of life measurement tool** such as the Short Form 36 questionnaire, and the European Organisation for Research and Treatment of Cancer Quality of Life Questionnaire-core.  Future trials are suggested to investigate the **effectiveness of Chinese herbal medicine in managing common symptoms** like pain, fatigue, and anorexia.  To provide **more rigorous evidence** [Chinese herbal medicine], future systematic reviews and trials must **adhere to high methodological and reporting standards.** |
| It is recommended that future meta-reviews that **focus on the prevention, detection and management** of **cancer treatment-induced toxicities** should **not include only Cochrane reviews**, as high-quality systematic reviews that **potentially contain unbiased and important recommendations for practice** could be overlooked. | Conway (2015)  The **prevention, detection and management** of cancer **treatment-induced cardiotoxicity**: a meta-review  It is recommended that future research should **not include only Cochrane reviews**, to **avoid overlooking** high-quality systematic reviews that potentially contain **unbiased and important recommendations**. |
| Future research is needed to **examine the acceptability and effectiveness of exercise** **and psychosocial interventions** in **more diverse populations**, and strategies to support optimal translation into routine clinical practice are needed. In a controlled trial, the **benefits of specific exercise regimes** **compared** with **usual care can be clearly tested**; however, there is limited evidence of these interventions being scaled effectively to reach large sections of the population in real‐world settings.  Future trials also need to **assess how to sustain intervention effects** over a longer **follow‐up period**. | Crawford‐Williams (2018)  **Interventions** for **prostate cancer survivorship**: A systematic review of reviews  Future research is needed to **examine the acceptability and effectiveness of exercise** **and psychosocial interventions** in **more diverse populations**.  In a controlled trial, the **benefits of specific exercise regimes** **compared** with **usual care can be clearly tested in a** population in real‐world settings.  Future trials need to **assess how to sustain intervention effects** over a longer **follow‐up period**. |
| There is not much evidence to **support comparative effectiveness of intervention modalities such as** **group versus individual**, **monodimensional versus multidimensional or multidisciplinary**; further work is needed to **examine these different approaches**. Given the **accessibility of social media** and its p**opularit**y, the findings that email contact was related to poorer **quality of life need further investigation**; although interactive websites were beneficial, the overall findings about digital interventions were equivocal.  Future research should consider the **effectiveness of interventions** **targeting people living beyond all types of cancer** and **with poor overall quality of life**. | Duncan (2017)  Review of systematic reviews of **non-pharmacological interventions** to **improve quality of life** in cancer survivors  Further research is needed to **examine comparative effectiveness of intervention modalities** such as group versus individual, monodimensional versus multidimensional or multidisciplinary. [non-pharmacological interventions]  Future research should consider the **effectiveness of interventions** **targeting people living beyond all types of cancer** and **with poor overall quality of life**. |
| Additional higher-quality research is needed, particularly in **non-surgical populations**, including **subgroup analyses to determine optimal exercise types and settings.** | Edbrooke (2023)  **Exercise** across the **lung cancer care** continuum: an overview of systematic reviews  Higher-quality research is needed, including **subgroup analyses to determine optimal exercise types and settings.** |
| A comparison between studies could be possible by using **toxicity criteria** applied by each investigator. An **absence of a high level of evidence trials** is observed regarding the **role of honey in oral mucotisis management**. Indeed, although some trials are characterized by adequate randomization and methodology, they lack in blindness. Moreover**, other randomized trials lack in blindness allocation concealment while others do not have adequate data or their samples are too small to give statistical power**. It is necessary that further trials should be conducted, fulfilling all the aforementioned characteristics in order to achieve a high level of evidence. In addition to this, one further interesting issue concerns the **variety of mucositis toxicity scales** that have occasionally been used. A question arises, thus, about the fact that if trials using the **same Oral mucositis toxicity scale were conducted**, **more powerful data** with strong cohesion could be derived. It is noteworthy that if more trials were conducted by setting and subsequently **studying the same criteria**, such as weight loss, pain degree or oral mucositis duration, concerning the participants' quality of life during their treatment, highly valuable data, which could **improve the quality of life,** might arise. | Gkantaifi (2020)  **Honey** against **radiation-induced oral mucositis** in **head and neck cancer** patients. an umbrella review of systematic reviews and meta- analyses of the literature  Indeed, although some trials are characterized by adequate randomization and methodology, they lack in blindness. Moreover**, other randomized trials lack in blindness allocation concealment while others do not have adequate data or their samples are too small to give statistical power.**  It is necessary that further trials should be conducted with **blinding, allocation concealment, and a sufficient sample size** to provide statistical power.  More trials should be conducted by setting and subsequently **studying the same criteria**, such as weight loss, pain degree or oral mucositis duration, concerning the participants' quality of life during their treatment. |
| Researchers are encouraged to use the **template for the intervention** description and **replication (TIDieR) checklist** [65].  Consensus around a **core outcome set to measure** impact on **psychological morbidity** is also required for use in both unimodal **psychological prehabilitation interventions** as well as multimodal approaches. Agreement around **optimal timing of assessments** in relation **to surgery and neoadjuvant/ adjvant therapies** is also warranted.  The **Principles and Guidance for Prehabilitation Within the Management and Support of People with Cancer** [60••] recommends a **triage system** that includes universal, targeted, and **specialist levels of prehabilitation**. This strategy proposes that all healthcare professionals can be equipped to provide “**universal” psychological support**.  We encourage those designing and reporting new clinical trials to **provide sufficient information to replicate their intervention**. Attention should also be given to **ensure new programs** have the **potential to be implemented within clinical practice** with consideration for designing and testing care that is **accessible and has the potential for large-scale implementation**. | Grimmett (2022)  **Psychological interventions** prior to **cancer surgery**: a review of reviews    Researchers should use the T**emplate for the intervention** description and **replication (TIDieR) checklist.**  Consensus around a **core outcome set to measure** impact on **psychological morbidity** is also required for use in both unimodal **psychological prehabilitation interventions** as well as multimodal approaches. Agreement around **optimal timing of assessments** in relation **to surgery and neoadjuvant/ adjvant therapies** is also warranted.  Consensus on a **core outcome set to measure psychological morbidity impact** is required for both single-method psychological prehabilitation and multimodal approaches. [psychological interventions]  Agreement on the **best timing for assessments** relative to surgery and neoadjuvant/adjuvant therapies is also essential. [cancer surgery]  Designing and reporting new clinical trials well is essential to **provide sufficient information to replicate their intervention.**  New psychological **intervention programs** should ensure they have the potential to be implemented **within clinical practice**, designed and tested for **accessibility in large-scale implementation**. |
| Guideline developers should consider describing the **resource implications of applying recommendations** and **suggest audit criteria** to monitor and drive implementation. Furthermore, editorial independence was assessed as being particularly poor for the guidelines identified. **To improve transparency within guideline development**, the views of the **funding body** and **competing interests** of the contributors should be disclosed and easy to identify.  Healthcare professionals should be aware that although the **risk of harm** is unlikely with most of these strategies, the **likelihood of benefit is often unclear**.  There is a need for **high-quality longitudinal primary research** of promising **self-management interventions** for **women with breast cancer using adjuvant endocrine therapy**. Findings from such trials should be **incorporated within clinical guidelines** that have considered how to **implement these strategies into routine practice**. | Hall (2022)  Strategies to **self-manage side-effects** of **adjuvant endocrine therapy among breast cancer survivors**: an umbrella review of empirical evidence and clinical guidelines  Guideline developers should consider describing the **resource implications of applying recommendations** and **suggest audit criteria** to monitor and drive implementation.  **To improve transparency within guideline development**, the views of the **funding body** and **competing interests** of the contributors should be disclosed.    There is a need for **high-quality longitudinal primary research** of promising **self-management interventions** for **women with breast cancer using adjuvant endocrine therapy**. |
| In order to confirm the **effects of resistance exercise**, more **rigorously designed**, **high-quality, large-scale,** randomized controlled **trials will be required** in the future.  Further reviews should focus on **standardization in reporting** and also aim to select randomized controlled trials of **higher quality and lower risk of bias**.  Given the difficulties we encountered in extracting data on **the specific modalities of exercise** (frequency, intensity, duration, and type), future studies should **refine the relevant data to facilitate clinical application**.  In the future, future studies should perform a **long-term follow-up** to **increase exercise intervention utilization** and **application in the clinical setting**.  More **rigorous randomized controlled trials** are recommended for verification in the future.  **Large randomized controlled trials** in patients with **advanced lung cancer** are recommended for verification, so as **to find suitable exercise for** patients with **poor exercise acceptance**.  We offer the following suggestions: first, **strictly controlling bias** when conducting original research; second, retrieve as much **comprehensive information from relevant original research** as possible in the writing of the review; third, **update systematic review in time** when new original research is being published; last but not least, it is recommended to **carry out more diversified original research in different environments**. | Hou (2023)  Is **physical activity** effective against **cancerrelated fatigue** **in lung cancer** patients? An umbrella review of systematic reviews and metaanalyses  More **rigorously designed**, **high-quality, large-scale,** randomized controlled **trials will be required** in the future in order to confirm the **effects of resistance exercise**.    Further reviews should focus on **standardization in reporting** and aim to select randomized controlled trials of **higher quality and lower risk of bias**.    Future studies should refine the relevant data of exercise to facilitate clinical application specifically frequency, intensity, duration, and type of exercise.  Future studies should perform a **long-term follow-up** to **increase exercise intervention utilization** and **application in the clinical setting**.  More **rigorous randomized controlled trials** are recommended for verification in the future.    **Large randomized controlled trials** in patients with **advanced lung cancer** are recommended **for finding suitable exercise for** patients with **poor exercise acceptance**.  **Strictly controlling bias** in future original research is essential.  Retrieving as much **comprehensive information from relevant original research** as possible in the writing of the review is needed. |
| **Exercise therapy** has been suggested as an **effective and convenient support care** intervention for **patients with breast cancer.**  The **efficacy of Tai chi in the treatment** of breast cancer **requires high-quality studies** to provide more convincing evidence.  We recommend that authors, readers, reviewers, and editors to become more acquainted with and to **more strictly adhere to the AMSTAR-2, PRISMA**, and **GRADE criteria** in future research.  Further rigorous, **comprehensive systematic reviews of meta-analysis and randomized controlled trials** that adhere to the guidelines are **required to provide robust evidence** for definitive conclusions. | Huang (2021)  The **effectiveness of tai chi** in patients with **breast cancer**: an overview of systematic reviews and meta-analyses    The **efficacy of Tai chi in the treatment** of breast cancer **requires high-quality studies** to provide more convincing evidence.  It is recommended to **adhere more strictly to the AMSTAR-2, PRISMA**, and **GRADE criteria** in future research.  Further rigorous, **comprehensive systematic reviews of meta-analysis and randomized controlled trials** that adhere to the guidelines are **required to provide robust evidence** for definitive conclusions. |
| Our result indicated that **aerobic exercise is a supportive intervention** on **reducing cancer related fatigue** and the quality of evidence was graded as moderate. These activities are **cost-effectiveness** and **should** **be popularized in clinical practice**.  Hence, **whether Yoga could alleviate fatigue among breast cancer patients** needs **further rigorous randomized controlled trials**.  It has been possible to suggest key components of future exercise interventions for fatigue in breast cancer patients. Firstly, future research is required to **clarify the effectiveness of different intervention modalities** (frequency, intensity, time and type) for breast cancer patients with fatigue to allow **determination of exercise dose-response**. Secondly, it is essential for future **randomized controlled trials and Meta analyses** should **not only focus on the effects of exercise on fatigue** but also how to **improve breast cancer patients’ adherence to exercise protocols**. Thirdly, additional studies are needed to **evaluate the cost-analysis and adverse events of exercise**. Fourthly, there is an urgent need for **innovative methods to generate high-quality evidence.** | Jiang (2020)  **Exercise for fatigue in breast cancer** patients: An umbrella review of systematic reviews  **Aerobic exercise** as a supportive intervention for reducing cancer-related fatigue is **cost-effective and should be popularized in clinical practice.**  **Further rigorous randomized controlled trials are needed to implement yoga among breast cancer patients.**  Future research is required **to clarify the effectiveness of different intervention modalities**, including frequency, intensity, time, and type, and **to determine the exercise dose-response**. [breast cancer patients with fatigue]  It is essential for future randomized controlled trials and meta-analyses should **focus on the effects of exercise on fatigue**.  It is essential for future randomized controlled trials and meta-analyses should focus on **improving breast cancer patients’ adherence to exercise protocols.**  Future studies are needed to **evaluate the cost-analysis and adverse events of exercise**.  Future research is needed for **innovative methods to generate high-quality evidence.** |
| There is an urgent need for **high-quality randomized controlled trials with a large sample size**, **better study design** and **more complete outcome assessment** to **provide high-quality evidence for the effects of probiotics on chemotherapy and radiotherapy-related diarrhoea** as well as **explore potential subgroup effects.** | Amiri Khosroshahi a (2023)  Effect of **probiotic supplementation** on **chemotherapy- and radiotherapy related diarrhoea** in patients with cancer: an umbrella review of systematic reviews and meta-analyses  There is a need for **high-quality randomized controlled trials with a large sample size provide high-quality evidence.** [probiotics for chemotherapy and radiotherapy-related diarrhoea]  **Better study design,** more **complete outcome assessment, and explore potential subgroup effects are needed.** [probiotics for chemotherapy and radiotherapy-related diarrhoea] |
| It is important that **higher-quality** **randomized controlled trials** be conducted for **more conclusive and clinically applicable results** to be generated. | Amiri Khosroshahi b (2023)  **Nutritional interventions** for the **prevention and treatment** of **cancer therapy-induced oral mucositis**: an umbrella review of systematic reviews and meta-analysis  It is recommended that **higher-quality randomized controlled trials** be conducted for **more conclusive and clinically applicable results**. [nutritional interventions for prevention of oral mucositis] |
| Future studies of **interventions** **aromatase inhibitor-associated arthralgia** for should **include an analysis of adverse events**.  A strategy for **controlling** **aromatase inhibitor-associated arthralgia** should not be focused on estrogen replacement therapy but **on symptom management**.  Further evaluation of the **effects of pharmacologic interventions, aerobic exercise, Nordic walking, omega-3 fatty acids, and vitamin D is necessary**. | Kim (2018)  **Therapeutic options** for **aromatase inhibitor-associated arthralgia in breast cancer survivors**: A systematic review of systematic reviews, evidence mapping, and network meta-analysis  A strategy for **controlling** **aromatase inhibitor-associated arthralgia** should be focused **on symptom management**.    Further evaluation of the **effects of pharmacologic interventions, aerobic exercise, Nordic walking, omega-3 fatty acids, and vitamin D is necessary**. [aromatase inhibitor-associated arthralgia in breast cancer survivors] |
| The review also highlights some notable gaps in evidence including **lower representation of cancers** other than **prostate and patients with comorbidities and sarcopenia** that should be **prioritised in future research**.  Future research should focus on **mechanisms underlying physical activity** effectiveness and on **underrepresented populations**. | Knowles (2022)  **Physical activity interventions** in **older people with cancer**: A review of systematic reviews  Future research should **prioritize lower representation of cancers** other than prostate cancer and **patients with comorbidities and sarcopenia.**  Future research should focus on **mechanisms underlying physical activity** effectiveness and on **underrepresented populations**. |
| There are **evidence gaps in** other topics, such as **spirituality, social connections, body image and coping strategies**. This indicates that there may be an opportunity for more focused and thorough reviews on these issues.  There was also a paucity of reviews on a number of other aspects of survivorship, such as **experiences of ongoing symptoms** (including side/late effects), **financial toxicity**, **multi-morbidity, and psychological issues** such as **fear of cancer recurrence, mental health disorders, stigma** and characteristics associated with better adjustment such as resilience. This likely indicates that these topics are currently underresearched or **lack comprehensive synthesis**, and there may be **potential opportunities for novel research** contributions. | Laidsaar-Powell (2019)  A meta-review of qualitative research on **adult cancer survivors**: current **strengths and evidence gaps**  Future research should more focus on **spirituality, social connections, body image and coping strategies** in cancer patients.  **Various aspects of survivorship** such as experiences of ongoing symptoms, financial toxicity, multi-morbidity, and psychological issues, including fear of cancer recurrence, mental health disorders, and stigma**,** need more investigation. |
| This study has suggested that the key to enabling BCS to lose weight could be through multicomponent interventions comprising dietary advice, physical activity and behavioural change support. Further research is required to determine the most effective combination/balance of these components, along with the **optimum duration and intensity**. Furthermore, studies with **long-term follow-up** are essential to assess whether positive impacts from intervention can be maintained in the long term, and crucially, whether or not this translates to a **reduction in the likelihood** of breast cancer **recurrence**. **Further research is needed** to define the optimum **combination, intensity and duration** of **a multimodal** component **intervention** for breast cancer survivors. | Lake (2022)  **Effectiveness** of **weight loss interventions** in **breast cancer survivors**: a systematic review of reviews  Further research is required to combine multicomponent interventions comprising physical activity, dietary advice, and behavioral change support, along with the optimum duration and intensity. [breast cancer survivors]  Studies with **long-term follow-up are essential** to assess whether **positive impacts from intervention can be maintained** in the long term. [weight loss interventions in breast cancer survivors] |
| The absence of **large RCTs** highlights the **need to expand the research** in the field with larger studies and individual patient data meta-analyses, in order to provide the **young cancer patients and survivors** with the **best care** available. In the clinical practice guideline that was included in our review, (Loeffen et al., 2020) the **use of hypnosis** was **strongly recommended** for all **needle procedures**, including **lumbar puncture** and **bone marrow aspiration**.  **A yearly psychosocial assessment** for **childhood cancer survivors** is therefore **recommended for prevention**.  The available scientific literature needs to be augmented and expanded**, including larger RCTs and meta-analyses** of individual patient data, in order to allow better decision-making and provide paediatric cancer patients, survivors and their families with the **best possible quality of life**. | Lavasidis (2022)  **Supportive interventions** for **childhood cancer**: An umbrella review of randomized evidence    Future randomized controlled trials are needed to **expand supportive care interventions for childhood cancer,** to provide the young cancer patients with the best care available.  **A yearly psychosocial assessment** for **childhood cancer survivors** is highly recommended **for prevention.**  **Future larger randomized controlled trials and meta-analyses are needed**, to provide paediatric cancer patients, survivors and their families with the **best possible quality of life**. |
| **Cryotherapy** should be initiated **5 to 30 minutes before cancer therapy** to ensure that **blood vessels** in the oral cavity have **sufficient time to constrict**.  Care should be taken to prevent an **oral temperature of less than 18°C** during **cryotherapy.**  **Cryotherapy** should be started **5 minutes before chemotherapy**, with the patient placing **crushed ice** or small pieces of ice in the mouth. Cryotherapy should **not exceed 30 minutes** per session.  Research on the **effectiveness of cryotherapy** in **reducing oral mucositis severity** remains limited; thus, more **clinical trials are warranted**. | Lee a (2023)  **Effectiveness of Cryotherapy** on Cancer Therapy–**Induced Oral Mucositis**  More clinical trials on the **effectiveness of cryotherapy** in **reducing oral mucositis severity** are needed. |
| Future studies must **treat the risk of methodological bias** with caution. **High-quality systematic review** in which selection of high-quality studies is combined with adequate methodology, are needed to **clarify the true efficacy** of **complementary therapies** for cancer patients. | Lee b (2018)  An overview of systematic reviews: **complementary therapies** for **cancer patients**  Future studies should reduce **the risk of methodological bias**.  **High-quality systematic review** in which selection of high-quality studies is combined with adequate methodology, are needed to **clarify the true efficacy** of **complementary therapies** for cancer patients. |
| First, for a greater number **of fully powered randomized controlled trials**, to enable more **robust conclusions about the efficacy** of **web-based psychosocial oncology interventions**. Second, we recommend that authors of future studies **report study uptake**, engagement, and **study outcomes transparently**, **adhering to CONSORT guidelines**. Third, we recommend the **use of outcome measures** that have been **validated within the target population**, with a preference for measures commonly used in previous research to support a more coherent and robust evidence base. Fourth, we recommend investigating **web-based psychosocial intervention effects** in a **broader range of patient populations**, including understudied national and cultural cohorts and men. Finally, we recommend **interventions** that are **directly targeted at specific diagnostic groups** or support needs, including customizable feedback and features, to encourage greater intervention engagement.  Future research can seek to promote both **intervention uptake and engagement** by addressing **participant anxiety about technology** and perceived time burden.  Future studies including **head-to-head comparisons**, which are fully **powered to conduct subgroup analyses**, are needed to conclusively establish what works best for maximizing recruitment, engagement, and efficacy. | Leslie (2022)  **Web-based psychological interventions** for people living with and beyond cancer: meta-review of what works and what does not for **maximizing recruitment, engagement, and efficacy**  There is needed for **fully powered randomized controlled trials**, to enable more **robust conclusions about the efficacy** of **web-based psychosocial oncology interventions.**  **Adhering to CONSORT guidelines, to report study outcomes transparently** is recommended.  **Outcome measures that validated within the target population,** to support more coherent and robust evidence base should be used.  Investigating **web-based psychosocial intervention effects** in a **broader range of patient** populations is needed.  Future research can promote both **intervention uptake and engagement** by addressing **participant anxiety about technology** and perceived time burden. [web-based psychological interventions]  Future studies including **head-to-head comparisons**, which are fully **powered to conduct subgroup analyses**, are needed. |
| It is important that health services are adequately resourced to respond to the range of clinical and broader patient needs, which can be identified through the **routine use of patient-reported outcomes**. **Question prompt lists** can **increase the number of questions** asked by patients without increasing **consultation length** and may encourage them to reflect and **plan questions** before the **consultation**.  Although we identified a large body of research showing that certain interventions can improve patient-physician communication, there is still a need for more **well-designed RCTs** of **novel interventions**.  We recommend **high-quality trials** of the **effectiveness of survivorship care plans**, the distress thermometer, and decision aids, with physicians as participants, to determine whether these improve patient participation in consultations; promote shared decision making; and improve understanding, satisfaction, and treatment outcomes. **Economic evaluation of the tools** to justify investment from health services is also warranted. Implementation studies are needed to provide evidence about the reach, uptake, fidelity, and scalability of the tools discussed in this overview. | Licquirish (2019)  **Tools** to **facilitate communication** during **physician-patient consultations** in cancer care: an overview of systematic reviews  There is still a need for more **well-designed randomized controlled trials** of **novel interventions** to improve patient-physician communication.  **High-quality trials** of the **effectiveness of survivorship care plans**, to determine improved **patient participation in consultations**, and promote shared decision-making are recommended. [physician-patient consultations**]**  **Economic evaluation of the tools** to justify investment in health services is needed. [Tools to facilitate communication with physician]  **Future implementation studies are needed** to provide evidence about the reach, uptake, fidelity, and scalability of the tools discussed in this overview. [Tools to facilitate communication with physician] |
| **Yoga** (a kind of practice combining stretches, meditation, and breathing to relax the mind and body) is recommended by the **latest international guidelines** such as guidelines set by the National Comprehensive Cancer Network (NCCN), American Society of Clinical Oncology (ASCO),42 and European Society for Medical Oncology (ESMO), to treat **cancer related fatigue** in patients either undergoing **active cancer treatment** or **post-treatment.**  A more **robust conclusion** needs to be further assessed through **well-designed** and **well-conducted clinical trials**.  Larger RCTs are needed to identify **the effects of dietary supplements** on **cancer related fatigue**.  It should be **well-designed in future RCTs** in accordance with previous studies and be considered as **a stratification factor** when analyzing the results in future systematic reviews.  The effects of other **complementary and alternative medicine interventions** on **cancer-related symptoms** warrant further investigation.  Conducting further research with **rigorous methodological designs** and **sufficient sample sizes** is still necessary. | Li (2023)  The role of **complementary and alternative medicine** on **cancer-related fatigue** in **adults**: an overview of systematic reviews    A more **robust conclusion** needs to be further assessed through **well-designed** and **well-conducted clinical trials**.  Larger randomised controlled trials are needed to identify **the effects of dietary supplements** on **cancer related fatigue.**  Well-designed randomised controlled trials in future is needed and should be considered a **stratification factor** **when analysing the results** in future systematic reviews.    The effects of other **complementary and alternative medicine interventions** on **cancer-related symptoms** warrant further investigation.  Conducting further research with **rigorous methodological designs** and **sufficient sample sizes** is necessary. [CAM on cancer-related fatigue] |
| Studies are also needed on **interventions** to **overcome** these **barriers to exercise** and to ensure adherence to **exercise regimes** to gain its **benefit on cancer recurrences** and for better quality of living during the survivorship phases.  Future occupational studies should investigate the **breadth and depth of rehabilitation** methods (eg, work stamina, tolerance, psychological factors for facilitating work re-entry, work accommodations such as flexibility towards work hours etc) for enabling post-operation survivors to **return to work** in a design that has a control or comparison group (ie, other types of interventions, wait-list, etc).  Future studies **with higher methodological rigor** should be conducted on **health promotion strategies** to enable healthy lifestyle. | Loh (2015)  Methods to improve **rehabilitation** of patients following **breast cancer surgery**: a review of systematic reviews  Studies are needed on **exercise interventions** to ensure **adherence to exercise regimes**, and to gain its **benefits on cancer recurrences.**  Studies are needed on interventions **to overcome barriers to exercise among breast cancer patients.**  Future occupational studies should **investigate the breadth and depth of rehabilitation methods** for enabling **post-operation survivors to return to work.**  Future studies **with higher methodological rigor** should be conducted on **health promotion strategies** to enable healthy lifestyle. |
| The difference between **objective and subjective measures** should be **further investigated** to understand whether **patients’ psychological interpretations** of their conditions might have affected their perceptions of any effects.  Given the evidence summarized above and bearing in mind the need for **high-quality studies** with **larger sample sizes**, a definitive, univocal decision is challenging to make on whether, when, and **how Qigong can be prescribed** to cancer patients.  It is paramount to **improve the quality of future clinical trials** on the matter by planning **longer follow-up times**, including **measures of variables** able to reflect cancer’s multifactorial nature and **Qigong’s core functioning**, and identifying **more objective measurements**, such as **the systematic investigation of** **biological markers**. | Mazzocco (2023)  Evidence for choosing **qigong** as an **integrated intervention in cancer care**: an umbrella review  The difference between **objective and subjective measures** should be **further investigated** to understand **patients’ psychological interpretations** of their conditions.    High-quality studies with larger sample sizes and longer follow-up times are needed. [qigong intervention] |
| Perhaps this is an area that could be addressed independently since there are **differences in quality of life** between **newly diagnosed breast cancer patients**, patients who are receiving different treatments, and **the long-term survivors** who successfully completed their treatments and now they have back to normal life.  It is important to notice that this review of reviews **did not separate the interventional studies** from other types of studies (usually descriptive or correlational). Perhaps a better organization might be to **reporting reviews** **based on separate objectives**. | Mokhtari-Hessari (2020)  Healthrelated **quality of life** **in breast cancer patients**: review of reviews from 2008 to 2018  It is important to notice that this review of reviews **did not separate the interventional studies** from other types of studies (usually descriptive or correlational).  **Reporting reviews** **based on separate objectives, interventional studies, and type of studies** is recommended. [quality of life in breast cancer patients] |
| Taking the next step from evaluating the **effect of narrow rehabilitation** studies on specific outcomes, **helping health care professionals** by identifying a knowledge base that could be used to enable **individualization in clinical practice**, requires a more **comprehensive approach** to **individualized rehabilitation**.  To establish a systematic way **of providing individualized rehabilitation**, further research is warranted **to bridge the gap** between rehabilitation research and clinical practice.  There is still a gap between **rehabilitation research** and **practice** which emphasis further research focusing on **dissemination and implementation** of available research findings. | Olsson Möller (2019)  A comprehensive approach to **rehabilitation interventions** following **breast cancer treatment** - a systematic review of systematic reviews  **Helping healthcare professionals** by enabling individualization in clinical practice **requires a more comprehensive approach to individualized rehabilitation.**    To establish a systematic way **of providing individualized rehabilitation**, further research is needed **to bridge the gap** between rehabilitation research and clinical practice.  There is still a gap between **rehabilitation research** and **practice** which emphasis further research focusing on **dissemination and implementation** of available research findings. |
| A further step on research in this field would be to test **the effectiveness** of **Mindfulness‐Based Stress Reduction /Mindfulness‐Based Cognitive Therapy/Mindfulness‐Based Cancer Recovery** on **different populations** to identify those who would **benefit more and how**, exploring the moderators. Furthermore, it would be interesting to determine if **Mindfulness‐Based Stress Reduction/Mindfulness‐Based Cognitive Therapy/Mindfulness‐Based Cancer Recovery** effects are clinically relevant, whether an **improvement in depression or anxiety or telomerase activity** is clinically significant, affecting health status.  Future studies should focus on **developing high‐quality studies** with **longer follow‐ups**, exploring the moderating effects that might contribute to **biased results**, and on **who might benefit** more from **Mindfulness‐Based Stress Reduction/Mindfulness‐Based Cognitive Therapy/Mindfulness‐Based Cancer Recovery** interventions. | Pedro (2021)  Evidence of **psychological and biological effects** of structured **Mindfulness‐Based Interventions** for **cancer patients** and survivors: A meta‐review  **The effectiveness** of **multiple components of mindfulness based interventions** on **different populations** to identify those who would **benefit more and how**, are needed to investigate.  Future studies should focus on **developing high‐quality studies** with **longer follow‐ups** and reducing **biased results.** [Mindfulness‐Based Interventions] |
| Considering all these limitations it was only possible to propose exercise training recommendations for future studies that can be adopted only in a second stage, after a **personalized intervention** planned to reach the minimum level of **physical activity**. Our exercise training recommendations **for breast cancer** patients was based on the main results and the **training protocols** adopted of the included studies considering the benefits of each intervention proposal.  Future studies should focalize the attention, with original researches, on the level of physical activity adapted to the breast cancer patients. Information should be collected before, **during and after the treatment** to present **data** **that can be personalized** according to the **patient’s necessities.**  An intervention should be composed of **combined training** **(aerobic and resistance training)** with a component of a **mindfulness intervention**, with an intensity from moderate to high, and 3 times a week. The intervention should be supervised in the first period and then it could be home-based. **Exercise** **training** should be **personalized** to the patients treated. | Petrigna (2023)  Methodological consideration for **a physical activity intervention** in **breast cancer population**: an umbrella review  Implementation of physical activity intervention outcomes should be collected **before**, **during and after the treatment** to present **data** **that can be personalized** according to the **patient’s necessities.** |
| At the same time, the importance of **personal contact components** was also found in this umbrella review, but the **frequency of assessment** or feedback in **exercise intervention** needs **further study**.  The **details of the intervention** still need further research, including the **effect of duration and frequency** on **patients’ physical activity levels**.  Faced with **different groups of patients**, such as overweight, sedentary behavior, the elderly, and limited physical activity, **appropriate interventions** should be adopted according to the **characteristics of the population**.  Future research may benefit cancer survivors by more **comprehensively applying electronic**, **wearable health technology-based**, **behavior change techniques** (BCTs), and **theory-based interventions**. | Qiu (2023)  **Promoting** **physical activity** among **cancer survivors**: an umbrella review of systematic reviews  The **details of the intervention** need further research, including the **effect of duration and frequency** on **patients’ physical activity levels**.  **Appropriate interventions** should be adopted according to the **characteristics of the different population**. [physical activity**]**  Future research should consider more **comprehensively applying electronic**, **wearable health technology-based**, **behavior change techniques,** and **theory-based interventions**. |
| We suggest considering the outcomes of the current umbrella review in everyday clinical practice; however, these **should be treated with caution** when reported by systematic reviews of critically low quality. Future research is needed to **focus on high-quality trials with long-term follow-up** and the o**ptimal type of exercise**, in addition to the duration of the exercise intervention. Equally important is the **determination of the optimum duration and intensity of exercise sessions**. The above characteristics are necessary to be determined in order for **exercise and physical activity guidelines for this population to be formed.** National or international **multicenter studies should be strongly encouraged** for this purpose. To achieve the above, **doctors and families should become familiar with the beneficial effects of exercise/physical activity programs** and their safe nature at all therapy phases. | Rapti (2023)  **Effects of exercise** and **physical activity levels** on **childhood cance**r: an umbrella review  Future research is needed to **focus on high-quality trials with long-term follow-up,** the o**ptimal type of exercise**, and the duration of the exercise intervention.  **Doctors and families should become familiar with the beneficial effects of exercise/physical activity programs** and their safe nature at all therapy phases. |
| **Leaflets and brochures should be provided in the language of the patients or survivors affected by cancer**, as only **providing languages spoken by the majority population was perceived as a barrier**, as reported by Alananzeh et al. with respect to Arabic migrant cancer patients [3].  Moreover, the **information and supportive care needs of migrants and ethnic minority cancer patients and survivors in Europe** should be **investigated in detail in future research**.  Providers of **supportive care in cancer should name concrete personnel** who are **responsible for the dissemination of specific information needs**.  **Information needs regarding sensitive topics of body/image and sexuality should be investigated with caution** but not be avoided**. Indirect ways to inform on these topics could help to fulfil the information needs in this domain.**  As **differences in information and supportive care needs** are present **between and within migrant and ethnic minority cancer patients and survivors**, **medical personnel should never overlook the individual characteristics of a patient**. | Ricetti (2021)  **Migrants and ethnic minorities** with cancer: an umbrella review on their information and supportive care needs  **Leaflets and brochures should be provided in the language of the foreigner patients or** survivors affected by cancer**.**  The information and **supportive care needs of migrants and ethnic minority cancer patients and survivors in Europe** should be **investigated in detail in future research**.  Providers of **supportive care in cancer should name concrete personnel** who are **responsible for the dissemination of specific information needs**.    **Information needs regarding sensitive topics of body/image and sexuality should be investigated with caution** but not be avoided**.** [migrants and ethnic minority needs]  As **differences in information and supportive care needs** are present **between migrant and ethnic minority cancer patients and survivors**, **medical personnel should never overlook the individual characteristics of a patient**. |
| **Lifestyle changes are recommended to reduce risk of cardiotoxicity and osteoporosis** including **addressing tobacco and alcohol use, managing weight, and increasing exercise.**  Future studies could **incorporate a wider range of research types**.  The quality of research within this meta-review was moderate, and **future studies should make use of methodological quality guidelines** when conducting systematic reviews and primary research.  To address gaps in the breast cancer research, future **studies should include breast cancer survivors with BRCA1/2 gene mutations**, **women receiving tailored treatments, women from low socioeconomic background**s, breast cancer survivors with **multimorbidity and complex health care needs, late effects, as well as interventions targeting gender and sexually diverse breast cancer survivors.** | King (2023)  **Psychosocial experiences** of **breast cancer survivors**: a meta-review  **Lifestyle changes are recommended to reduce risk of cardiotoxicity and osteoporosis** including **addressing tobacco and alcohol use, managing weight, and increasing exercise.**    Future studies could **incorporate a wider range of research types.**  **Future studies should make use of methodological quality guidelines** when conducting systematic reviews and primary research.  Future studies should include breast cancer survivors with **BRCA1/2 gene mutations**, **women receiving tailored treatments, women from low socioeconomic background**s, breast cancer survivors with **multimorbidity and complex health care needs, late effects, as well as interventions targeting gender and sexually diverse breast cancer survivors.** |
| **Well-detailed prospective studies should be designed** to increase the quality of evidence on **osteoradionecrosis treatment** protocols.  More **robust clinical studies are needed** to establish the best treatment for ORN.  The **treatment choice** should be **based on stage and patient’s clinical osteoradionecrosis condition.** | Schroter (2023)  **Osteoradionecrosis treatment in head and neck cancer patients**: An overview of systematic reviews  **Well-detailed prospective studies should be designed** to increase the quality of evidence on **osteoradionecrosis treatment** protocols.    More **robust clinical studies are needed** to establish the best treatment for **osteoradionecrosis.**  The **treatment choice** should be **based on stage and patient’s clinical osteoradionecrosis condition.** |
| In future research (both at the level of separate intervention studies and the level of systematic reviews), **more attention should be given to the effects of eHealth interventions in relation to the disease stage.**  The fact that the **Comprehensive Health Enhancement Support System for breast cancer patients is the most researched** eHealth intervention among the available eHealth interventions. This is all the more reason why **future research should concentrate on specific tumor types**.  **More tailored eHealth interventions may yield stronger effects**. However, **more research is needed to confirm this hypothesis**.  Recommendation to focus **future research on the identified lacunas and separately study different types of eHealth interventions, like single-component and multi-component eHealth interventions.** | Slev (2016)  **Effects of eHealth** for patients and **informal caregivers confronted with cancer**: A meta-review  In future research, **more attention should be given to the effects of eHealth interventions in relation to the disease stage.**    **Future research should concentrate on specific tumor types**.  More research is needed **to tailor eHealth interventions to yield stronger effects.**    **Future research should focus on separately studying different types of eHealth interventions, like single-component and multi-component eHealth interventions.** |
| To **make tumor boards effective, professionals should consider them a critical part of their working** **agenda** and save time to prepare and attend tumor boards. Moreover, **congruent time should be dedicated to the meetings** in order to avoid discussing many cases in a short amount of time. Additionally, the **time for acting on decisions made during the tumor boards should be taken into account** (Lamb et al. 2011) | Specchia (2020)  The **impact of tumor board on cancer care**: evidence from an umbrella review  To **make tumor boards effective, professionals should consider them a critical part of their working** **agenda** and save time to prepare and attend tumor boards.  **Congruent time should be dedicated to the meetings** in order to avoid discussing many cases in a short amount of time. |
| Future research should focus on **interventions that save resources and are relatively easy to implement in daily practice**. **Large‐scale longitudinal research with follow‐up measures is needed to identify long‐term effects,** especially on the lives of patients who have **survived cancer or live with chronic illness**.  To improve the effectiveness, authors recommended considering the individual characteristics of the participants, such as educational level, disease stage and treatment trajectory, when administering EW interventions to patients with cancer.  Future investigation needs **to focus on the most beneficial intervention components to guide clinical practice development**. | Tuominen (2018)  **Effectiveness of nursing interventions** among patients with cancer: An overview of systematic reviews  Future research should focus on **nursing interventions that save resources and are relatively easy to implement in daily practice.**  **Large‐scale longitudinal research with follow‐up measures is needed to identify long‐term effects,** especially on the lives of patients who have **survived cancer or live with chronic illness**. [nursing interventions]  **Considering the individual characteristics of the participants**, such as educational level, disease stage and treatment trajectory, when administering interventions to patients with cancer is recommended.  Future investigation needs **to focus on the most beneficial intervention components to guide clinical practice development**. |
| Finally, most systematic reviews did not clarify whether ‘**standard’ or ‘usual’ care** included supportive cancer care, as **a paradigm for modern treatment** in oncology [72], to manage the **physical, psychological, social, and spiritual needs of patients** [73], or specific cancer treatments such as chemotherapy. This needs to be **clarified in future systematic reviews**. | TriguerosMurillo (2023)  Effects of **musicbased interventions** on **cancerrelated pain, fatigue, and distress**: an overview of systematic reviews  ‘S**tandard’ or ‘usual’ care** included supportive cancer care, to manage the **physical, psychological, social, and spiritual needs of patients**, needs to be **clarified in future systematic reviews**. [musicbased interventions] |
| Our recommendation is that **mHealth technologies for cancer survivors** implement industry **standard data encryption to ensure the security of private information.**  **More research** is recommended in how quality is defined in **mHealth among cancer survivors**. Further research may help propel the development of a **comprehensive quality framework** that allows for **consistent evaluation of mHealth technologies in cancer survivors**. | Tune (2022)  How Is **Quality of mHealth Interventions** for **Cancer Survivors** Defined and Described? An Umbrella Review  It is recommended that **mHealth technologies** implement industry-standard data encryption to ensure the security of private information.  **More research** is recommended in how quality is defined in **mHealth among cancer survivors**.  Developing a **comprehensive quality framework** for **standardized evaluation of mHealth technologies** in cancer survivors is recommended. |
| Indicating **a more rigorous design** **and evaluation** is needed **to avoid this information bias. (not blinding)**  It is well-needed to report how those **case management intervention** were conducted **follow standard reporting guidelines**, in order to provide recommendation for future research.  Future research should **clearly describe details of case management intervention** and its implementation, **including theoretical underpinnings, dose and intensity, interventionist qualifications, protocol or manual used, fidelity**, etc. In that way these details can be included in future systematic reviews, and **effectiveness of individual elements of the intervention can be examined** [27]. We recommend **use standard guidelines to help organize** the case management intervention reporting. For example, the Template for Intervention Description and Replication (TIDeiR) is one of the most popular guidelines that could be used to report the full breadth of case management interventions: from intervention rationale to assessments of treatment adherence and fidelity [42]. 2) **More rigorous trials are needed to evaluate the effectiveness** of case management. 3) Studies should also **explore the barriers to and facilitators of case management implementation** across various types of cancer patients at different stages, **providing evidence for conducting successful case management implementation in the future.** | Wang (2022)  The **effectiveness of case management** for cancer patients: an umbrella review  Indicating **a more rigorous design** **and evaluation** is needed **to avoid blinding bias.** [case management]  It is needed to report how **case management intervention** were conducted **follow standard reporting guidelines**, to provide recommendation for future research.  Future research should **clearly describe details of case management intervention** and its implementation, **including theoretical underpinnings, dose and intensity, and interventionist qualifications.**  **Using standard guidelines to help organize** the case management intervention reporting is recommended.  Studies should **explore the barriers to and facilitators of case management implementation** across various types of cancer patients at different stages. |
| Future confirmatory **trials should adhere to CONSORT recommendations for reporting**. Researchers should also **adopt a comparative effectiveness approach and design trials** **that allow real-world evaluation of acupuncture and related therapies**.  Specifically, the **combined effects of acupuncture and related therapies** in addition to **guideline-recommended conventional care** (e.g., glucocorticoids, 5-HT3 antagonists and/or NK1R antagonists in CINV) should be compared with conventional care alone so that the **additional benefits of acupuncture can be elucidated.**  Future trials should **choose the most clinically relevant endpoint as the primary outcome** and **measure it using a validated method**11 so as to **ensure the utility of future clinical evidence.**  **Future comparative effectiveness research** in this area should pay attention to **improving the reporting and methodological quality of trials**.  **Developing an acupuncture treatment protocol** using an expert consensus technique, taking into account **regulatory requirements and the constraints of the practice setting.**  **Describing the treatment protocol according to the STRICTA51 and TIDieR checklist52**, so that the **procedure can be replicated in other trials or be adopted into clinical practice** if it is found to be effective.  And **choosing guideline-recommended treatment** for the **control group and validating outcome measures**. | Wu a (2015)  **Effectiveness of acupuncture** and **related therapies for palliative care** of cancer: overview of systematic reviews  Future **trials should adhere to CONSORT recommendations for reporting**. [palliative care]  Researchers should **adopt a comparative effectiveness approach and design trials** that allow real-world evaluation of acupuncture and related therapies.  Future trials should measure the most clinically relevant endpoint as the primary outcome **using a validated method,** to **ensure the utility of future clinical evidence. [palliative care]**  **Future comparative effectiveness of interventions** research should pay attention to **improving the reporting and methodological quality of trials**.  **Developing an acupuncture treatment protocol** using an expert consensus technique, considering **regulatory requirements and the constraints of the practice setting.**    Describing the **treatment protocol according to the TIDieR checklist**, so that the **procedure can be replicated in other trials.** |
| **More studies on measurement instruments should be explored**, with attention to **early detection of lymphedema flow, convenient clinical application, and accurate measuring.**  More **well designed and large RCTs are needed** to provide **a higher level of evidence to confirm the role of manual lymphatic drainage in complete decongestive therapy**, especially for **patients under 60 years of age or with 1 month intervention duration.** | Xing (2023)  **Effectiveness of manual lymphatic drainage** for **breast cancer-related lymphoedema**: an overview of systematic reviews and meta-analyses  **More studies on measurement instruments should be explored**, with attention to **early detection of lymphedema flow, and accurate measuring.** [breast cancer-related lymphoedema]  More **well designed and large randomized controlled trials are needed** to provide a higher level of evidence **to confirm the role of manual lymphatic drainage in complete decongestive therapy.** [breast cancer related lymphoedema] |
| Methodological limitations of RCTs have limited the trustworthiness of these conclusions and future RCTs should address the following: use a **Chinese herbal medicine placebo in the control group**; ensure **blinding of outcome assessment**; **measuring patient outcomes comprehensively using validated scales**; report **trial implementation and results according to the CONSORT statement**; and **reducing publication bias by releasing RCT protocols on trial registries**. | Wu b (2016)  **Chinese herbal medicine** for **improving quality of life among nonsmall cell lung cancer patients**  Future randomized controlled trials should use **Chinese herbal medicine placebo in the control group,** and ensure **blinding of outcome assessment.**  Future randomized controlled trials should **measure patient outcomes comprehensively using validated scales.** [Chinese herbal medicine]  Future randomized controlled trials should report trial implementation and results **according to the CONSORT statement.** [Chinese herbal medicine]  Future randomized controlled trials should **reduce publication bias by releasing randomized** **controlled trials** **protocols on trial registries**. |
| In the future, in order to prescribe, complementary and alternative medicine the **health care professionals should be more careful**. **The methodological quality for primary studies was low and their sample size was small**, so in the future **large and well-designed RCTs should be conducted** to confirm the conclusions of available systematic reviews.  The key methodological aspects, such as **methods of randomization**, **concealed allocation, and blinding**, should **be well conducted and reported**. | Bao (2014)  **Complementary and alternative medicine** for **cancer pain**: an overview of systematic reviews  Future **large and well-designed randomized controlled trials** are needed to evaluate the effectiveness of Complementary and alternative medicine for cancer pain.  **Methods of randomization**, **concealed allocation, and blinding** should be well conducted and reported in future studies. [Complementary and alternative medicine] |
| Future studies should focalize attention on **the different effects of physical activity on breast cancer patients** **under therapy or under other conditions**. | Zanghi (2022)  The practice of **physical activity on psychological, mental, physical, and social wellbeing** for **breast-cancer survivors**: an umbrella review  Future studies should focalize attention on **the different effects of physical activity on breast cancer patients** **under therapy or under other conditions**. |
| Clinical trials are **strongly recommended to report by CONSORT Statement** and its extension to acupuncture trials (STRICTA) (Hughes et al., 2019) to keep a high methodological quality, and no more low-quality, insufficient reported studies (Anshasi and Ahmad, 2021).  **According to the Preferred Reporting Items for Systematic reviews and Meta-Analyses (PRISMA)** (Page et al., 2021), it is recommended to **register protocol before conducting, provide exclusion list if possible, and report by acknowledged criteria.**  Future reviews are recommended to **report according to the acknowledged reporting standards** **to improve the quality of evidence**. | Zhang a (2022)  **Acupuncture for cancer-related conditions**: An overview of systematic reviews    Clinical trials are **recommended to report by CONSORT Statement** and its extension to acupuncture trials (STRICTA) to keep a high methodological quality.  It is recommended to **register protocol before conducting, provide exclusion list if possible.**    Future reviews are recommended to **report according to the reporting standards** **to improve the quality of evidence**. |
| Future research should **employ the incidence of adverse events as the main evaluation index of the study.** | Zhang b (2023)  **Effectiveness of exercise interventions** in the **management of cancerrelated fatigue**: a systematic review of systematic reviews  Future research should **employ the incidence of adverse events as the main evaluation index of the study.** [exercise interventions] |
| The **quality of methodology needs to be further improved**.  In conducting an SR/MA, the **PRISMA statement should be used as basis** in **preparing a normative report to improve the overall report quality**.  In the future, other **physical measurement indicators for evaluating obesity** are expected.  However, whether or not they (**alternative exercise traditions) are more advantageous than conventional rehabilitation training** still **needs to be supported by a large sample size and strict standardized trials.**  Future research should **incorporate a large number of samples (in the baseline and follow-up phases), long-term follow-up evaluations** (eg, 6 months or more), and **clearly defined targeted measurement indicators into the design**. | Zhang c (2020)  How can **alternative exercise traditions** help against the **background of the covid-19 in cancer care**? an overview of systematic reviews  The **quality of methodology needs to be further improved**.    **PRISMA statement should be used as guidance** in **preparing a normative report to improve the overall report quality** in future systematic reviews and meta-analysis.  **In the future, more physical measurement indicators for evaluating obesity** are expected.    Future research should **incorporate a large number of samples, long-term follow-up evaluations,** and **clearly defined targeted measurement indicators into the design**. |
| First, future studies must **increase the sample size**. Second, although a **growing number of recent studies** have explored changes in symptom clusters or symptom networks over time during breast cancer adjuvant treatment, their clinical practice is inadequate. The complexity of the symptoms is one potential cause. To solve this problem, future research **may use ecological transient assessment to dynamically assess symptoms**. Thirdly, we found that there is still a **lack of research on the effects of exercise on relieving symptom clusters** or **symptom networks during adjuvant therapy in breast cancer patients**. We expect that further research will be conducted to **examine the effects of exercise on improving bridge symptoms** identified within or between symptom clusters and, in addition, **advance the development of symptomics using symptom network analysis**. This will improve the efficiency of symptom management and better meet the specific needs of more patients with breast cancer. | Zhao (2023)  The **effectiveness of exercise** on the **symptoms in breast cancer patients undergoing adjuvant treatment**: an umbrella review of systematic reviews and meta-analyses  Large sample size is needed in future studies. [effectiveness of exercise]  A growing number of recent studies have explored **changes in symptom clusters or symptom networks** over time during **breast cancer adjuvant treatment**, their clinical practice is needed.  Future research should use **ecological transient assessment to dynamically measure symptoms**.  **The effects of exercise on relieving symptom clusters** or **symptom networks during adjuvant therapy in breast cancer patients**.  Further research should be conducted to examine **the effects of exercise on improving bridge symptoms** identified within or **between symptom clusters**.  **Advancing the development of symptomics using symptom network analysis** is needed. |
| For future studies, **a list of excluded studies should be provided** **as an independent appendix** to journals **to facilitate readers’ understanding of the data selection process** and further improve the reliability of the review findings.  **Funding sources should be clearly declared** in future publications to **help readers determine whether funding bias existed**. To achieve a comprehensive literature search, future systematic reviews are suggested to identify **potential studies by searching not only the commonly used databases but also gray literature retrieval websites to minimize publication bias.**  In order to further improve the level of evidence of the included SRs, **more original studies with rigorous study designs and detailed descriptions of the intervention protocols** (e.g., type, frequency, intensity, and duration of the exercise) are necessary.  **More rigorously designed clinical studies are needed** to specify the exact **exercise type, duration, frequency, and intensity to have an optimal effect on cancer related fatigue in breast cancer patients.** | Zhou a (2022)  **Effects of exercise interventions** on **cancerrelated fatigue in breast cancer** patients: an overview of systematic reviews  **A list of excluded studies** should be provided as an **independent appendix** to journals **to facilitate readers’ understanding** of the data selection process and further **improve the reliability of the review** findings.  **Funding sources** should be clearly declared in future publications to **help readers determine whether funding bias existed**.  To achieve a comprehensive literature search, future systematic reviews are suggested to identify **potential studies by searching not only the commonly used databases but also gray literature retrieval websites to minimize publication bias.**  To achieve a comprehensive literature search, future systematic reviews should also search **gray literature, to retrieval websites to minimize publication bias.**  **Improving the level of evidence** of the included systematic reviews, more original studies with rigorous study designs and detailed descriptions of the intervention protocols such as, type, frequency, intensity, and duration of the exercise are necessary. |
| **Rigorous RCTs and systematic reviews are needed** to provide **high-quality evidence for the specificity of exercise interventions**, to more clearly delineate the specific effects of each type of exercise and to **establish the appropriate volume for each type of exercise, with the goal of optimising outcomes for surgical lung cancer patients.**  More high-quality research is required, to **evaluate the effects of different types and amounts of exercises on health outcomes for surgical lung cancer patients.** | Zhou b (2020)  **Effects of perioperative exercise interventions** on **lung cancer patients**: An overview of systematic reviews  Rigorous randomized controlled trials and systematic reviews are needed **to provide** **high-quality evidence for the specificity of exercise interventions.**  More high-quality research is required, to **evaluate the effects of different types and amounts of exercises on health outcomes for surgical lung cancer patients.** |
