# Supplementary material for: Umbrella Reviews Conducted in an Oncology Healthcare Context Focusing on Supportive Care, Systems, and Models of Care: A Review of Umbrella Reviews
Source: Cancer Med. 2026 Mar 25;15(4):e71708. doi: 10.1002/cam4.71708 (PMC13140849; doi:10.1002/cam4.71708)
Supplement: Supplementary file 6 — Data S2: Supporting Information. [file CAM4-15-e71708-s006.docx]

Contents

[Stage 1. Extraction of text and formation of codes, grouping of codes under preliminary headings 6](#_Toc186401580)

[Workforce 6](#_Toc186401581)

[Return to work 6](#_Toc186401582)

[Funding healthcare and policy 6](#_Toc186401583)

[Access to services 6](#_Toc186401584)

[Future research 6](#_Toc186401585)

[Healthcare professionals voice/ HCP competencies 7](#_Toc186401586)

[Patient voice, PPI 7](#_Toc186401587)

[Outcome measures 7](#_Toc186401588)

[Multiple components of interventions 8](#_Toc186401589)

[Further intervention necessity 9](#_Toc186401590)

[Specific population/diagnosis 10](#_Toc186401591)

[Diverse population/diagnosis 10](#_Toc186401592)

[Validity of the tools 11](#_Toc186401593)

[Transparency 11](#_Toc186401594)

[Standardized approach 11](#_Toc186401595)

[Reporting standards 12](#_Toc186401596)

[Guidance/framework/ theory underpinning interventions, studies and methodological approaches 12](#_Toc186401597)

[Methodological needs 13](#_Toc186401598)

[Cost effectiveness/Economic evaluation 14](#_Toc186401599)

[Symptom and adverse effect management 14](#_Toc186401600)

[Utilization of intervention/application of clinical setting 15](#_Toc186401601)

[Survivorship 15](#_Toc186401602)

[Patient necessities/ individualized needs 15](#_Toc186401603)

[Implementation/ clinical practice 15](#_Toc186401604)

[Minority populations needs 16](#_Toc186401605)

[Comparing effectiveness of interventions 16](#_Toc186401606)

[Higher level of evidence 16](#_Toc186401607)

[Bias 17](#_Toc186401608)

[Evidence gaps 17](#_Toc186401609)

[Quality of life 17](#_Toc186401610)

[Stage 2. Iteratively grouping codes 18](#_Toc186401611)

[Recommendations for workforce shortages 19](#_Toc186401612)

[Return to work recommendation 19](#_Toc186401613)

[Funding healthcare and policy recommendations 19](#_Toc186401614)

[Recommendations for accessing to services 19](#_Toc186401615)

[Recommendations for healthcare professionals voice/ HCP competencies 19](#_Toc186401616)

[Recommendations for patient voice, PPI, and patient experiences 20](#_Toc186401617)

[Recommendations for future intervention research 20](#_Toc186401618)

[Further intervention necessity recommendations 21](#_Toc186401619)

[Recommendation for specific population/patients with cancer and specific diagnosis 23](#_Toc186401620)

[Recommendations for diverse/ underrepresented population and diagnosis 24](#_Toc186401621)

[Recommendations for validity of the tools 24](#_Toc186401622)

[Recommendations for transparency 25](#_Toc186401623)

[Recommendations for standardized approach 25](#_Toc186401624)

[Recommendations for reporting standards 25](#_Toc186401625)

[Guidance/framework/ theory underpinning interventions, studies and methodological approaches 26](#_Toc186401626)

[Recommendations for methodological needs 27](#_Toc186401627)

[Recommendations for cost-analysis and effectiveness 28](#_Toc186401628)

[Recommendations for utilization of intervention/application of clinical setting 29](#_Toc186401629)

[Recommendations for survivorship 29](#_Toc186401630)

[Recommendations for patient necessities/ individualized needs 29](#_Toc186401631)

[Recommendations for implementation/ clinical practice 30](#_Toc186401632)

[Recommendations for migrant and minority population needs 31](#_Toc186401633)

[Recommendations for comparing effectiveness of interventions 31](#_Toc186401634)

[Recommendations for higher level of evidence 31](#_Toc186401635)

[Recommendations for preventing bias 31](#_Toc186401636)

[Recommendations for addressing evidence gaps 32](#_Toc186401637)

[Recommendations for enhancing quality of life 32](#_Toc186401638)

[Stage 3. Iteratively grouping codes and creation of final codes 33](#_Toc186401639)

[Recommendations for workforce shortages 34](#_Toc186401640)

[Funding healthcare and policy recommendations 34](#_Toc186401641)

[Recommendations for accessing to services 34](#_Toc186401642)

[Recommendations for healthcare professionals 34](#_Toc186401643)

[Recommendations for patient voice, PPI, and patient experiences 35](#_Toc186401644)

[Recommendations for future intervention research 35](#_Toc186401645)

[Recommendation for specific population/patients with cancer and specific diagnosis 37](#_Toc186401646)

[Recommendations for diverse/ underrepresented population and diagnosis 38](#_Toc186401647)

[Recommendations for validity of the tools 38](#_Toc186401648)

[Recommendations for transparency 39](#_Toc186401649)

[Recommendations for standardized approach 39](#_Toc186401650)

[Recommendations for reporting standards 39](#_Toc186401651)

[Recommendations for guidance/framework/ theory underpinning interventions, studies and methodological approaches 40](#_Toc186401652)

[Recommendations for cost-analysis and effectiveness 42](#_Toc186401653)

[Recommendations for utilization of intervention/application of clinical setting 43](#_Toc186401654)

[Recommendations for survivorship 43](#_Toc186401655)

[Recommendations for patient necessities/ individualized needs 43](#_Toc186401656)

[Recommendations for implementation/ clinical practice 44](#_Toc186401657)

[Recommendations for migrant and minority population needs 45](#_Toc186401658)

[Recommendations for comparing effectiveness of interventions 45](#_Toc186401659)

[Recommendations for preventing bias 45](#_Toc186401660)

[Recommendations for addressing evidence gaps 46](#_Toc186401661)

[Recommendations for enhancing quality of life 46](#_Toc186401662)

[Stage 4. Creating overarching and subthemes 47](#_Toc186401663)

[Recommendations for future review efforts 47](#_Toc186401664)

[Recommendations for future trials 47](#_Toc186401665)

[Recommendations for future interventions and experimental studies 48](#_Toc186401666)

[Recommendations for diverse diagnosis/patients 49](#_Toc186401667)

[Recommendations for future policymakers, Health care managers, leaders and team and researchers 50](#_Toc186401668)

## Stage 1. Extraction of text, pertaining to recomendations, from uncluded umbrella reviews and formation of codes, grouping of codes under preliminary headings

## Workforce

Investment in terms of time and resources in the training of competent palliative care workforce is a facilitator in addressing the **workforce shortages**.

Volunteers can play crucial roles in supporting the health of cancer patients and overcoming **workforce shortages**.

## Return to work

Future occupational studies should **investigate the breadth and depth of rehabilitation methods** for enabling **post-operation survivors to return to work.**

## Funding healthcare and policy

Recommended that policy makers [in low- and middle-income countries] **collaborate** with national and international organisations to secure **funding** for improving **health care provision** [in terms of palliative care].

Future research must expand **robust economic evaluations** for a wider range of models of care to **provide evidence for health systems to fund** and promote the transition to alternative models.

For policy development, **assessing the country readiness** for the provision and integration of palliative care is an essential step to an effective **adoption [of palliative care**].

**Future research** efforts [in palliative care] are needed to develop a **body of evidence** that is adequate to support **effective learning and policy development**.

## Access to services

Integrating palliative care into **primary care services** is a recommended strategy to improve **access to palliative care** for patients living in remote area.

Evaluating the use of different technological platforms is recommended to **provide telemedicine services.**

[Lymphoma] Patients with **limited treatment options** or persistent frailty despite rehabilitative attempts should be **offered palliative care services** in combination with or, where appropriate, replacing restorative and curative approaches.

## Future research

Future **research** efforts [in palliative care] are needed to develop a **body of evidence** that is adequate to support **effective learning and policy development**.

Future trials of educational interventions [relating to cancer pain] should be based on **underlying theoretical model** and consider factors which might influence and modify the effect.

Future randomized controlled trials are required to generate results that are **more clinically reliable**.

Future primary studies should consider and explicitly report patient-important outcomes [quality of life, symptom control]**,** to **provide useful data for evidence syntheses** and **clinical practice guidelines**.

Further research is needed to **establish firm evidence and further recommendation**. [acupuncture and moxibustion for cancer-related fatigue]

Well reported **observational studies and randomised controlled trials** are needed to clarify the presence of **short and long term toxicities of Chinese herbal medicine**.

Future trials need to **assess how to sustain intervention effects** over a longer **follow‐up period**. [Interventions for prostate cancer]

Future research is needed for agreement on the **best timing for assessments** relative to surgery and neoadjuvant/adjuvant therapies. [cancer surgery]

More **rigorous randomized controlled trials** are recommended for verification of evidence in the future. [physical activity intervention]

There is needed for **fully powered randomized controlled trials**, to enable more **robust conclusions about the efficacy** of **web-based psychosocial oncology interventions.**

Future studies **with higher methodological rigor** should be conducted on **health promotion strategies** to enable healthy lifestyle.

Future research should **employ the incidence of adverse events as the main evaluation index of the study.** [exercise interventions]

More studies with **homogeneous samples of cancer patients** are needed.

## Healthcare professionals voice/ HCP competencies

Re **health care professionals’ voice** [in palliative care] need to investigate **communication competencies** between patient and healthcare providers and their relationship with their patients in palliative care.

Studies of **patient and healthcare providers** education should include a **patient outcome.**

## Patient voice, PPI

Re **patients’/family voice** [in palliative care] need **to address the priorities**, needs, and wishes of patients about palliative care services in low- and middle-income countries.

Physical activity interventions should be integrated into **patients’ experiences**, recognizing the impairing effects of cancer treatments, home and working life, and patient’s physical and psychological needs.

Data is needed on the **perspectives of all stakeholders [regarding return to work after diagnosis]** to inform **intervention development.**

**High-quality trials** of the **effectiveness of survivorship care plans**, to determine improved **patient participation in consultations**, and promote shared decision-making are recommended. [physician-patient consultations]

## Outcome measures

It might also be desirable **to assess alternative outcome measures**, for example use of out of hours and emergency services.

Consensus around a **core outcome set to measure psychological morbidity impact** is required for both single-method psychological prehabilitation and multimodal approaches. [psychological interventions]

The difference between **objective and subjective outcome measures** should be further investigated to understand **patients’ psychological interpretations** of their conditions. [qigong intervention]

More studies on **outcome measurement instruments should be explored**, with attention to early detection of lymphedema flow, and accurate measuring. [breast cancer-related lymphoedema]

In the future, **more physical measurement indicators** for evaluating obesity are expected.

Future research should use **ecological transient assessment to dynamically measure symptoms**.

Future trials are suggested to adopt **more specific quality of life measurement tool.**

## Multiple components of interventions

Future research should [cancer pain] identify the active **components of complex interventions**, and to be able to target interventions to groups most likely to benefit.

Future **interventions should be multi-factorial,** and addressing the diverse range of components, including **survivors’ personal goals and needs**, **workplace communication**, **culture, policy and resources, and the wider family, culture and societal context.** [return to work after diagnosis]

Future research is required **to clarify the effectiveness of different intervention modalities**, including frequency, intensity, time, and type, and **to determine the exercise dose-response**. [breast cancer patients with fatigue]

Further research is required to **combine multicomponent interventions** comprising physical activity, dietary advice, and behavioral change support, along with the optimum duration and intensity. [breast cancer survivors]

Future research can promote both **intervention uptake and engagement** by addressing **participant anxiety about technology** and perceived time burden. [web-based psychological interventions]

The effectiveness of **multiple components of mindfulness‐based interventions** on **different populations** to identify those who would **benefit more and how**, are needed to investigate.

Future research should focus on separately studying **different types of eHealth interventions, like single-component and multi-component eHealth interventions.**

Future investigation needs **to focus on the most beneficial intervention components** to guide clinical practice development. [nursing interventions]

Future research should **clearly describe details of case management intervention** and its implementation, including theoretical underpinnings, dose and intensity, and interventionist qualifications.

More high-quality research is required, to **evaluate the effects of different types and amounts of exercises** on health outcomes for surgical lung cancer patients.

The **details of the intervention** need further research, including the **effect of duration and frequency** on **patients’ physical activity levels**. [physical activity intervention]

Future research is needed to **focus on high-quality trials with long-term follow-up,** the o**ptimal type of exercise**, and the duration of the exercise intervention.

## Further intervention necessity

The mucosal tissue of children is different from that of adults, and **more interventions** [regarding cryotherapy] are needed to provide more conclusive evidence.

Additional studies are needed to **clarify the effect of self-management/e-health and educational interventions** on cancer related fatigue considering selected populations’ digital health literacy.

The safety of **psychostimulants** needs to be investigated in future trials for assessing effects of the long-term therapies.

Future trials are suggested to investigate the **effectiveness of Chinese herbal medicine** in managing common symptoms like pain, fatigue, and anorexia.

The **efficacy of Tai chi in the treatment** of breast cancer **requires high-quality studies** to provide more convincing evidence.

Further rigorous randomized controlled trials are needed to **implement yoga among breast cancer patients.**

Further evaluation of the **effects of pharmacologic interventions, aerobic exercise, Nordic walking, omega-3 fatty acids, and vitamin D is necessary**. [aromatase inhibitor-associated arthralgia in breast cancer survivors]

Future research should more focus on **spirituality, social connections, body image and coping strategies** in cancer patients.

Future randomized controlled trials are needed to **expand supportive care interventions for childhood cancer,** to provide the young cancer patients with the best care available.

**A yearly psychosocial assessment** for **childhood cancer survivors** is highly recommended **for prevention.**

More clinical trials on the **effectiveness of cryotherapy** in **reducing oral mucositis severity** are needed.

Investigating **web-based psychosocial intervention effects** in a **broader range of patient** populations is needed.

Larger randomised controlled trials are needed to identify **the effects of dietary supplements** on **cancer related fatigue.**

There is still a need for more **well-designed randomized controlled trials** of **novel interventions** to improve patient-physician communication.

The effects of other **complementary and alternative medicine interventions** on **cancer-related symptoms** warrant further investigation.

Studies are needed on **exercise interventions** to ensure **adherence to exercise regimes**, and to gain its **benefits on cancer recurrences.**

Future research should consider more **comprehensively applying electronic**, **wearable health technology-based**, **behavior change techniques,** and **theory-based interventions**.

In future research, **more attention should be given to the effects of eHealth interventions in relation to the disease stage.**

Future **large and well-designed randomized controlled trials** are needed to evaluate the effectiveness of Complementary and alternative medicine for cancer pain.

Further research should be conducted to examine **the effects of exercise on improving bridge symptoms** identified within or **between symptom clusters**.

Future research is warranted **to evaluate the efficacy of particular interventions** [psychosocial interventions] within population clusters and examine their long-term effectiveness.

Undertake research to explore the **use of telemedicine** to **support chronic disease management**, **medication management**, **cancer screening, surveillance** for recurrence, **and disease prevention.**

More research is needed to **tailor eHealth interventions to yield stronger effects.**

## Specific population/diagnosis

Future research should focus on testing these interventions [non-pharmacological interventions] on **specific cancer population clusters and trajectories**.

Future research should consider **individual cancer population clusters** and **direct comparisons between therapeutic options [pharmacological interventions]**.

More robust research recommended to emphasis the **lack of focus on specific cancer diseases** and to **facilitate direct comparisons between concurrent complementary and integrative medicine interventions.**

The effectiveness of patient navigation interventions **for patients with advanced or metastatic cancers and those in palliative care and end‐of‐life care settings** needs to be explored.

Future research should dedicate the **effectiveness of patient navigation** **in common cancers**, such as **prostate cancer, lung cancer, and melanoma**; rare cancer types; and **hematologic malignancies**.

Research into **indigenous populations worldwide** is needed to understand the **unique cultural factors** facing indigenous people.

Large randomized controlled trials in **patients with** **advanced lung cancer** are recommended **for finding the suitable exercise for** patients with **poor exercise acceptance**.

Studies are needed on interventions **to overcome barriers to exercise among breast cancer patients.**

Future studies should include breast cancer survivors with **BRCA1/2 gene mutations**, **women receiving tailored treatments, women from low socioeconomic background**s, breast cancer survivors with **multimorbidity and complex health care needs, late effects, as well as interventions targeting gender and sexually diverse breast cancer survivors.**

Future research should **concentrate on specific tumor types.**

Future studies should focalize attention on the different effects of physical activity **on breast cancer patients under therapy or under other conditions.**

## Diverse population/diagnosis

Appropriate interventions should be adopted according to the **characteristics of the different population.** [physical activity]

Future research needs to be conducted to enable an overall synthesis of the **effect of physical exercise** **on other cancer diagnoses.**

Future research should focus on mechanisms underlying physical activity effectiveness and on **underrepresented populations**.

Future research should **prioritize lower representation of cancers** other than prostate cancer and **patients with comorbidities and sarcopenia.**

Future studies should **examine the populations** covering **different cancer types, ages, languages, demographic groups, educational levels, and in remote, rural, or low-resource settings** over extended periods. [telemedicine]

Future research should consider the **effectiveness of interventions** **targeting people living beyond all types of cancer** and **with poor overall quality of life**. [non-pharmacological interventions]

Future research is needed to **examine the acceptability and effectiveness of exercise** **and psychosocial interventions** in **more diverse populations**.

Future research is needed to expand the understanding of **effective models of care in diverse cancer survivor populations** **including paediatric cancer survivors, adolescent and young adult survivor group**, **older adults**, and **a broader range of cancer types** as well as **advanced stages of the diseas**e.

## Validity of the tools

The future **critical appraisal of the characteristics of the available tools for measuring cancer-related fatigue** could help to clarify domains cancer-related fatigue could be defined.

[physical exercise] Future research should clarify the quality of the evidence regarding the **validity and reliability of the several tools to measure cancer-related fatigue** for providing a **theory-grounded base for clinicians and researchers.**

Future research evaluating models of care should be conducted **using validated tools to assess outcomes.**

In the assessment of outcomes, **validated tools and objective measures should be prioritized**.

Future trials should measure the most clinically relevant endpoint as the primary outcome **using a validated method,** to **ensure the utility of future clinical evidence. [palliative care]**

**Outcome measures that validated within the target population,** to support more coherent and robust evidence base should be used.

Future randomized controlled trials should measure patient outcomes comprehensively **using validated scales.** [Chinese herbal medicine]

## Transparency

Further studies need to be conducted to **address pharmacological interventions specifically and transparently** for the treatment of cancer related fatigue.

There is a need for the publication **of more transparency descriptions of complex interventions**.

Future trials need to **transparent in the design** and **promote methodological strictness** throughout the trial process.

**Timing of assessment**, the **duration of the intervention**, and **longer follow-up periods** should address detailed transparently in further research. [Psychosocial interventions]

**To improve transparency within guideline development**, the views of the **funding body** and **competing interests** of the contributors should be disclosed.

‘S**tandard’ or ‘usual’ care** included supportive cancer care, to manage the physical, psychological, social, and spiritual needs of patients, **needs to be** **transparent in future systematic reviews**. [musicbased interventions]

**A list of excluded studies should be provided** **transparently** as an independent appendix to journals **to facilitate readers’ understanding of the data selection process** and further improve the reliability of the review findings.

## Standardized approach

Future policy research is needed to inform consensus **best‐practice standards,** including **standardized definitions and criteria** for cancer patient navigation.

**Standards and guidelines** should be developed to guide implementors on **the optimal approach to deploy telemedicine**.

Future research should determine the optimal delivery methods using **standardized telemedicine screening/assessing tools**, **interventions, and outcomes.**

## Reporting standards

Future reviews are recommended to **report according to the reporting standards** to improve the quality of evidence.

It is needed to report how **case management intervention** were conducted **follow standard reporting guidelines**, to provide recommendation for future research.

To provide more rigorous evidence [Chinese herbal medicine], future systematic reviews and trials must **adhere to high methodological and reporting standards.**

Further reviews should focus on **standardization in reporting** and aim to select randomized controlled trials of higher quality and lower risk of bias.

The **standardized reporting of the parameters of the different programs are** needed to enable the design of specific intervention protocols. [Psychosocial interventions]

## Guidance/framework/ theory underpinning interventions, studies and methodological approaches

Future **trials of educational interventions** [relating to cancer pain] should be based on **underlying theoretical model and consider factors** which might influence and modify the effect.

**The Medical Research Council framework** for complex interventions could be used to **guide future projects.**

Future systematic review should comply with the **PRISMA statement** that it is **useable for policy makers and clinicians.**

Researchers should use the Template for the intervention description and replication **(TIDieR) checklist.**

Further rigorous, comprehensive systematic reviews of meta-analysis and randomized controlled trials that **adhere to the guidelines are required to provide robust evidence for definitive conclusions.**

**Adhering to CONSORT guidelines, to report study outcomes transparently is recommended.**

Future studies should make use of **methodological quality guidelines when conducting systematic reviews and primary research.**

**Using standard guidelines, such as** Template for the intervention description and replication **(TIDieR) checklist** to help organize the case management intervention reporting is recommended.

Future trials should **adhere to CONSORT recommendations** for reporting. [palliative care]

Describing the **treatment protocol according to** Template for the intervention description and replication **TIDieR checklist**, so that the **procedure can be replicated in other trials. [palliative care]**

**Future randomized controlled trials should report trial implementation and results according to the CONSORT statement**. [Chinese herbal medicine]

Clinical trials are recommended to **report by CONSORT Statement and its extension to acupuncture trials (STRICTA)** to keep a high methodological quality.

**PRISMA statement should be used as guidance** in **preparing a normative report to improve the overall report quality** in future systematic reviews and meta-analysis.

Further research should focus on **frameworks’** **implementation** to deliver tailored interventions [physical exercise interventions].

It is recommended that **the development of best practice guidelines** including decision trees for selecting the most appropriate model of care for the individual cancer survivors, implementation guides, and standardized outcomes for the evaluation.

Developing a **comprehensive quality framework** for standardized evaluation of mHealth technologies in cancer survivors is recommended.

It is recommended to **adhere more strictly to the AMSTAR-2, PRISMA**, and **GRADE criteria** in future research.

## Methodological needs

It is recommended that future research should **not include only Cochrane reviews**, to **avoid overlooking** high-quality systematic reviews that potentially contain **unbiased and important recommendations**.

Higher-quality research is needed, including **subgroup analyses to determine optimal exercise types and settings.**

It is necessary that further trials should be conducted with **blinding, allocation concealment, and a sufficient sample size** to provide statistical power.

**Designing and reporting new clinical trials well** is essential to **provide sufficient information to replicate their intervention.**

Retrieving as much **comprehensive information from relevant original research** as possible in the writing of the review is needed.

A better study design, more complete outcome assessment, and exploration of potential subgroup effects are needed. [probiotics for chemotherapy and radiotherapy-related diarrhoea]

**High-quality systematic review** in which selection of high-quality studies is **combined with adequate methodology,** are needed to clarify the true efficacy of complementary therapies for cancer patients.

A more **robust conclusion** needs to be further assessed through **well-designed** and **well-conducted clinical trials**.

Conducting further research with **rigorous methodological designs** and **sufficient sample sizes** is necessary. [CAM on cancer-related fatigue]

High-quality studies **with larger sample sizes and longer follow-up times** are needed. [qigong intervention]

**Reporting reviews** **based on separate objectives, interventional studies, and type of studies** is recommended. [quality of life in breast cancer patients]

Future studies could **incorporate a wider range of research types.**

**Large‐scale longitudinal research with follow‐up measures is needed to identify long‐term effects,** especially on the lives of patients who have survived cancer or live with chronic illness. [nursing interventions]

Further evidence is needed from high-quality trials with large samples that **fully report rigorous methodological characteristics in the design stage.**

Future research is needed from high-quality trials that **estimate the optimal sample size** based on the existing research results, aiming to ensure that the **conclusions drawn from the research are valid.**

Future randomized controlled trials should use Chinese herbal medicine **placebo in the control group and ensure blinding of outcome assessment.**

**Methods of randomization, concealed allocation, and blinding** should be well conducted and reported in future studies. [Complementary and alternative medicine]

It is recommended to **register protocol before conducting, provide exclusion list if possible.**

Future research should **incorporate a large number of samples, long-term follow-up evaluations,** and **clearly defined targeted measurement indicators into the design**. [alternative exercise traditions]

**Large sample size** is needed in future studies. [effectiveness of exercise]

**Advancing the development of symptomics using symptom network analysis** is needed. [exercise on the symptoms in breast cancer]

Studies with **long-term follow-up are essential** to assess whether **positive impacts from intervention can be maintained** in the long term. [weight loss interventions in breast cancer survivors]

Future studies including **head-to-head comparisons**, which are fully **powered to conduct subgroup analyses**, are needed. [Web-based psychological interventions]

Well-designed randomised controlled trials in future is needed and should be considered a **stratification factor** **when analysing the results** in future systematic reviews. [complementary and alternative medicine on cancer-related fatigue]

Future systematic reviews should consider outcomes [quality of life, symptom control] **in their protocols**, planning to meta-analyse data from primary studies. [Systemic oncological treatments versus supportive care]

More **rigorously designed**, **high-quality, large-scale,** randomized controlled **trials will be required** in the future, to confirm the effects of resistance exercise.

**Improving quality and reporting** is recommended for future research.

It is recommended that **higher-quality randomized controlled trials** be conducted **for more conclusive and clinically applicable results**. [nutritional interventions for prevention of oral mucositis]

## Cost effectiveness/Economic evaluation

**Re economic evaluations** should be conducted to examine potential cost-effectiveness or cost-minimization following **implementation of telemedicine interventions.**

**Aerobic exercise** as a supportive intervention for reducing cancer-related fatigue is **cost-effective and should be popularized in clinical practice.**

Future studies are needed to **evaluate the cost-analysis and adverse events of exercise**.

**Economic evaluation** of the tools to justify investment in health services is needed.

## Symptom and adverse effect management

**Cancer related fatigue should be assessed** on a regular basis in clinical settings to aid in the identification of effective therapies, treatments, and management.

It is essential for future randomized controlled trials and meta-analyses should **focus on the effects of exercise on fatigue**.

A strategy for controlling aromatase inhibitor-associated arthralgia should be **focused on symptom management.**

**Lifestyle changes** are recommended to **reduce risk of cardiotoxicity and osteoporosis** including addressing tobacco and alcohol use, managing weight, and increasing exercise.

More robust clinical studies are needed to establish the **best treatment for** **osteoradionecrosis.**

## Utilization of intervention/application of clinical setting

Future studies should perform a **long-term follow-up** to **increase exercise intervention utilization** and **application in the clinical setting**.

Future studies should refine the relevant data of exercise to **facilitate clinical application** specifically frequency, intensity, duration, and type of exercise.

Future research should focus on **nursing interventions that save resources and are relatively easy to implement in daily practice.**

Studies should explore **the barriers to and facilitators of case management implementation** across various types of cancer patients at different stages.

Future randomized controlled trials and meta-analyses should focus on **improving breast cancer patients’ adherence to exercise protocols.**

## Survivorship

**Various aspects of survivorship** such as experiences of ongoing symptoms, financial toxicity, multi-morbidity, and psychological issues, including fear of cancer recurrence, mental health disorders, and stigma**,** need more investigation.

## Patient necessities/ individualized needs

Implementation of physical activity intervention outcomes should be collected before, during and after the treatment to present **data** **that can be personalized** according to the **patient’s necessities.**

**Considering the individual characteristics of the participants,** such as educational level, disease stage and treatment trajectory, when administering interventions to patients with cancer is recommended.

The **treatment choice** should be **based on stage and patient’s clinical osteoradionecrosis condition.**

Future studies [lymphoma] should consider **patient characteristics**, outcome measures, timing, mode and intensity of rehabilitation interventions.

The International Classification of Functioning, Disability and Health model can be used as a common framework **to help prioritize** **personalized goals** for patients with lymphoma to **set rehabilitation criteria**.

To establish a systematic way of providing **individualized rehabilitation,** further research is needed **to bridge the gap between rehabilitation research and clinical practice.**

## Implementation/ clinical practice

**Doctors and families should become familiar with the beneficial effects of exercise/physical activity programs** and their safe nature at all therapy phases.

Providers of **supportive care in cancer should name concrete personnel** who are **responsible for the dissemination of specific information needs**.

To **make tumor boards effective, professionals should consider them a critical part of their working** **agenda** and save time to prepare and attend tumor boards.

**Congruent time [tumor boards] should be dedicated** to the meetings in order to **avoid discussing many cases in a short amount of time.**

It is recommended that **mHealth technologies implement industry-standard data encryption** to ensure the security of private information.

A growing number of recent studies have explored **changes in symptom clusters or symptom networks** over time during **breast cancer adjuvant treatment**, their **clinical practice is needed**.

Implementation research studies is recommended to examine and **maximize effectiveness, adoption, implementation and maintenance outcomes** of the telemedicine strategies over time.

**Future implementation studies are needed** to provide evidence about the reach, uptake, fidelity, and scalability of the tools discussed in this overview. [Tools to facilitate communication with physician]

New psychological **intervention programs** should ensure they have the potential to be implemented **within clinical practice**, designed and tested for **accessibility in large-scale implementation**.

Interventions tested in trials should be **further tested in real-world settings, especially at the population level.** [models of cancer survivorship care]

**Leaflets and brochures should be provided in the language of the foreigner patients or** survivors affected by cancer**.**

## Minority populations needs

The information and **supportive care needs of migrants and ethnic minority cancer patients and survivors in Europe** should be **investigated in detail in future research**.

**Information needs regarding sensitive topics of body/image and sexuality should be investigated with caution** but not be avoided**.** [migrants and ethnic minority needs]

As **differences in information and supportive care needs** are present between migrant and ethnic minority cancer patients and survivors, **medical personnel should never overlook the individual characteristics of a patient**.

## Comparing effectiveness of interventions

Researchers should **adopt a comparative effectiveness approach and design trials** that allow real-world evaluation of acupuncture and related therapies.

Future controlled trials should compare the **benefits of specific exercise regimes** **compared** with **usual care in a** population in real‐world settings.

Further research is needed to **examine comparative effectiveness of intervention modalities** such as group versus individual, monodimensional versus multidimensional or multidisciplinary. [non-pharmacological interventions]

**Future comparative effectiveness of interventions** research should pay attention to improving the reporting and methodological quality of trials.

## Higher level of evidence

More well designed and large randomized controlled trials are needed **to provide a higher level of evidence** to confirm the role of manual lymphatic drainage in complete decongestive therapy. [breast cancer related lymphoedema]

**Improving the level of evidence** of the included systematic reviews, more original studies with rigorous study designs and detailed descriptions of the intervention protocols such as, type, frequency, intensity, and duration of the exercise are necessary.

Rigorous randomized controlled trials and systematic reviews are needed **to provide high-quality evidence for the specificity of exercise interventions.**

Future research is needed for **innovative methods to generate high-quality evidence.**

There is a need for high-quality randomized controlled trials with a large sample size **to provide high-quality evidence.** [probiotics for chemotherapy and radiotherapy-related diarrhoea]

## Bias

Funding sources should be clearly declared in future publications to **help readers determine whether funding bias existed.**

**To prevent publication bias,** it is recommended that all clinical trials protocols on the topic should register with a recognized platform.

**Strictly controlling bias** in future original research is essential.

Future studies should **reduce the risk of methodological bias**.

Indicating a more rigorous design and **evaluation** is needed **to avoid blinding bias.** [case management]

Future randomized controlled trials should **reduce publication bias by releasing randomized controlled trials protocols on trial registries. [**Chinese herbal medicine]

Future studies should focus on developing high‐quality studies with longer follow‐ups and **reducing** **biased results.** [Mindfulness‐Based Interventions]

To achieve a comprehensive literature search, future systematic reviews should also search **gray literature, to retrieval websites to minimize publication bias.**

## Evidence gaps

Future systematic reviews should **explicitly report evidence gaps** in primary research.

Future studies should **prioritize robust primary studies to address gaps in the literature** for outcomes in the domains of health promotion**, chronic conditions, clinical structure, and decision-making.**

## Quality of life

**Future larger randomized controlled trials and meta-analyses are needed**, to provide paediatric cancer patients, survivors and their families with the **best possible quality of life**.

More trials should be conducted by setting and subsequently **studying the same criteria**, such as weight loss, pain degree or oral mucositis duration, **concerning the participants' quality of life during their treatment.** [honey intervention for oral mucositis]

# Stage 2. Iteratively grouping codes

| Heading | Original codes | New draft codes |
| --- | --- | --- |
| **Recommendations for future research efforts** | Future **research** efforts [in palliative care] are needed to develop a **body of evidence** that is adequate to support **effective learning and policy development**.  Future trials of educational interventions [relating to cancer pain] should be based on **underlying theoretical model** and consider factors which might influence and modify the effect [of interventions].  Future randomized controlled trials are required to generate results that are **more clinically reliable**. (check meaning)  Future primary studies should consider and explicitly report patient-important outcomes [quality of life, symptom control]**,** to **provide useful data for evidence syntheses** and **clinical practice guidelines**.  Further research is needed to **establish firm evidence and further recommendation**. [acupuncture and moxibustion for cancer-related fatigue]  Well reported **observational studies and randomised controlled trials** are needed to clarify the presence of **short and long term toxicities of Chinese herbal medicine[s]**.  Future trials need to **assess how to sustain intervention effects** over a longer **follow‐up period**. [Interventions for prostate cancer]  Future research is needed for agreement on the **best timing for assessments** relative to surgery and neoadjuvant/adjuvant therapies. [cancer surgery]  More **rigorous randomized controlled trials** [of non-pharmacological interventions] are recommended for verification of evidence in the future. [physical activity intervention]  There is needed for **fully powered randomized controlled trials**, to enable more **robust conclusions about the efficacy** of **web-based psychosocial oncology interventions.**  Future studies **with higher methodological rigor** should be conducted on **health promotion strategies** to enable healthy lifestyle.  Future research should **employ the incidence of adverse events as the main evaluation index of the study.** [exercise interventions]  More studies with **homogeneous samples of cancer patients** are needed. | Future **research** efforts are needed to develop a **body of evidence** that is adequate to support **policy development**.  Further research is needed to **establish firm evidence and further recommendation**.  Future trials should be based on **underlying theoretical model** and consider factors which might influence and modify the effect of interventions.  Future randomized controlled trials are required to generate results that are **more clinically reliable**.  More **rigorous randomized controlled trials** of non-pharmacological interventions are recommended.  There is needed for **fully powered randomized controlled trials**, to enable more **robust conclusions about the efficacy** of **web-based oncology interventions.**  Future studies **with higher methodological rigor** should be conducted on **health promotion strategies.**  More studies with **homogeneous samples of cancer patients** are needed.  Future primary studies should explicitly report patient-important outcomes to **provide useful data for evidence syntheses** and **clinical practice guidelines**.  Well reported **observational studies and randomised controlled trials** are needed to clarify the presence of **short and long term toxicities of herbal medicine**.  Future research should **employ the incidence of adverse events as the main evaluation index of the study.**  Future trials need to **assess how to sustain intervention effects** over a longer **follow‐up period**.  Future research is needed for agreement on the **best timing for assessments** relative to surgery and neoadjuvant/adjuvant therapies. |
| **Recommendations for workforce shortages** | Investment in terms of time and resources in the training of competent palliative care workforce is a facilitator in addressing the **workforce shortages**.  Volunteers can play crucial roles in supporting the health of cancer patients and overcoming **workforce shortages**. | Investment in time and resources in the training of a competent care workforce is a facilitator in addressing the **workforce shortages**.  Volunteers can support the health of cancer patients and overcoming **workforce shortages**. |
| **Return to work recommendation** | Future occupational studies should investigate **the breadth and depth of rehabilitation methods** for enabling **post-operation survivors to return to work.** | Future studies should investigate **the breadth and depth of rehabilitation methods** for **post-operation survivors to return to work.** |
| **Funding healthcare and policy recommendations** | Recommended that policy makers [in low- and middle-income countries] **collaborate** with national and international organisations to secure **funding** for improving **health care provision** [in terms of palliative care].  Future research must expand **robust economic evaluations** for a wider range of models of care to **provide evidence for health systems to fund** and promote the transition to alternative models.  For policy development, **assessing the country readiness** for the provision and integration of palliative care is an essential step to an effective **adoption [of palliative care**].  **Future research** efforts [in palliative care] are needed to develop a **body of evidence** that is adequate to support **effective learning and policy development**. | Recommended that policy makers **collaborate** with national and international organisations to secure **funding** for improving **health care provision.**  Future research should expand **robust economic evaluations** to **provide evidence for health systems to fund** and promote the transition to alternative models.  For policy development, **assessing the country readiness** for the provision and integration of evidence-based care is recommended.  **Future research** efforts are needed to develop a **body of evidence** that is adequate to support **policy development**. |
| **Recommendations for accessing to services** | Integrating palliative care into **primary care services** is a recommended strategy to improve **access to palliative care** for patients living in remote area.  Evaluating the use of different technological platforms is recommended to **provide telemedicine services.**  [Lymphoma] Patients with **limited treatment options** or persistent frailty despite rehabilitative attempts should be **offered palliative care services** in combination with or, where appropriate, replacing restorative and curative approaches. | Integrating palliative care into **primary care services,** to improve **access to palliative care** for patients living in remote area is recommended.  Patients with **limited treatment options** or persistent frailty despite rehabilitative attempts should be **offered palliative care services.**  Evaluating the use of different technological platforms is recommended to **provide telemedicine services.** |
| **Recommendations for healthcare professionals voice/ HCP competencies** | Re **health care professionals’ voice** [in palliative care] need to investigate **communication competencies** between patient and healthcare providers and their relationship with their patients in palliative care.  **Helping healthcare professionals** by enabling individualization in clinical practice requires a more comprehensive approach to **individualized rehabilitation**. | **Health care professionals** need to investigate **communication competencies** between patient and healthcare providers.  **Helping healthcare professionals** by enabling individualization in clinical practice requires a more comprehensive approach to **individualized rehabilitation**. |
| **Recommendations for patient voice, PPI, and patient experiences** | Re **patients’/family voice** [in palliative care] need **to address the priorities**, needs, and wishes of patients about palliative care services in low- and middle-income countries.  Physical activity interventions should be integrated into **patients’ experiences**, recognizing the impairing effects of cancer treatments, home and working life, and patient’s physical and psychological needs.  Data is needed on the **perspectives of all stakeholders [regarding return to work after diagnosis]** to inform **intervention development.**  **High-quality trials** of the **effectiveness of survivorship care plans**, to determine improved **patient participation in consultations**, and promote shared decision-making are recommended. [physician-patient consultations] | **Patients’/family voice** need **to address the priorities** in low- and middle-income countries.  Non-pharmacologic interventions should be integrated into **patients’ experiences**, recognizing the impairing effects of cancer treatments, home and working life, and patient’s physical and psychological needs.  Data is needed on the **perspectives of all stakeholders,** to improve **intervention development.**  High-quality trials of the **effectiveness of survivorship care plans**, to determine improved **patient participation in phsycian consultations**, and promote shared decision-making are recommended. |
| **Recommendations for future intervention research** | Future research should [cancer pain] identify the active **components of complex interventions**, and to be able to target interventions to groups most likely to benefit.  Future **interventions should be multi-factorial,** and addressing the diverse range of components, including **survivors’ personal goals and needs**, **workplace communication**, **culture, policy and resources, and the wider family, culture and societal context.** [return to work after diagnosis]  Future research is required **to clarify the effectiveness of different intervention modalities**, including frequency, intensity, time, and type, and **to determine the exercise dose-response**. [breast cancer patients with fatigue]  Further research is required to **combine multicomponent interventions** comprising physical activity, dietary advice, and behavioral change support, along with the optimum duration and intensity. [breast cancer survivors]  Future research can promote both **intervention uptake and engagement** by addressing **participant anxiety about technology** and perceived time burden. [web-based psychological interventions]  The effectiveness of **multiple components of mindfulness‐based interventions** on **different populations** to identify those who would **benefit more and how**, are needed to investigate.  Future research should focus on separately studying **different types of eHealth interventions, like single-component and multi-component eHealth interventions.**  Future investigation needs **to focus on the most beneficial intervention components** to guide clinical practice development. [nursing interventions]  Future research should **clearly describe details of case management intervention** and its implementation, including theoretical underpinnings, dose and intensity, and interventionist qualifications.  More high-quality research is required, to **evaluate the effects of different types and amounts of exercises** on health outcomes for surgical lung cancer patients.  The **details of the intervention** need further research, including the **effect of duration and frequency** on **patients’ physical activity levels**. [physical activity intervention]  Future research is needed to **focus on high-quality trials with long-term follow-up,** the o**ptimal type of exercise**, and the duration of the exercise intervention. | Future research should identify the active **components of complex interventions.**  Future **interventions should be multi-factorial,** and addressing the diverse range of components, including **survivors’ personal goals and needs**, **workplace communication**, **culture, policy and resources, and the wider family, culture and societal context.**  Future research is required **to clarify the effectiveness of different intervention modalities**, including frequency, intensity, time, and type, and **to determine the intervention dose-response**.  Further research is required to **combine multicomponent interventions** comprising physical activity, dietary advice, and behavioral change support, along with the optimum duration and intensity.  Future research can promote both **intervention uptake and engagement** by addressing **participant anxiety about technology** and perceived time burden.  The effectiveness of **multiple components of interventions** on **different populations** to identify those who would **benefit more and how**, are needed to investigate.  Future research should focus on separately studying **different types of interventions.**  Future investigation needs **to focus on the most beneficial intervention components** to guide clinical practice development.  Future research should **clearly describe details of intervention** and its implementation, including theoretical underpinnings, dose and intensity, and interventionist qualifications.  More high-quality research is required, to **evaluate the effects of different types and amounts of** on health outcomes for patients.  The **details of the intervention** need further research, including the **effect of duration and frequency** on **patients’ physical activity levels**.  Future research is needed to **focus on high-quality trials with long-term follow-up,** the o**ptimal type of intervention**, and the duration of the intervention. |
| **Further intervention necessity recommendations** | The mucosal tissue of children is different from that of adults, and **more interventions** [regarding cryotherapy] are needed to provide more conclusive evidence.  Additional studies are needed to **clarify the effect of self-management e-health and educational interventions** on cancer related fatigue considering selected populations’ digital health literacy.  The safety of **psychostimulants** needs to be investigated in future trials for assessing effects of the long-term therapies.  Future trials are suggested to investigate the **effectiveness of Chinese herbal medicine** in managing common symptoms like pain, fatigue, and anorexia.  The **efficacy of Tai chi in the treatment** of breast cancer **requires high-quality studies** to provide more convincing evidence.  Further rigorous randomized controlled trials are needed to **implement yoga among breast cancer patients.**  Further evaluation of the **effects of pharmacologic interventions, aerobic exercise, Nordic walking, omega-3 fatty acids, and vitamin D is necessary**. [aromatase inhibitor-associated arthralgia in breast cancer survivors]  Future research should more focus on **spirituality, social connections, body image and coping strategies** in cancer patients.  Future randomized controlled trials are needed to **expand supportive care interventions for childhood cancer,** to provide the young cancer patients with the best care available.  **A yearly psychosocial assessment** for **childhood cancer survivors** is highly recommended **for prevention.**  More clinical trials on the **effectiveness of cryotherapy** in **reducing oral mucositis severity** are needed.  Investigating **web-based psychosocial intervention effects** in a **broader range of patient** populations is needed.  Larger randomised controlled trials are needed to identify **the effects of dietary supplements** on **cancer related fatigue.**  There is still a need for more **well-designed randomized controlled trials** of **novel interventions** to improve patient-physician communication.  The effects of other **complementary and alternative medicine interventions** on **cancer-related symptoms** warrant further investigation.  Studies are needed on **exercise interventions** to ensure **adherence to exercise regimes**, and to gain its **benefits on cancer recurrences.**  Future research should consider more **comprehensively applying electronic**, **wearable health technology-based**, **behavior change techniques,** and **theory-based interventions**.  In future research, **more attention should be given to the effects of eHealth interventions in relation to the disease stage.**  Future **large and well-designed randomized controlled trials** are needed to evaluate the effectiveness of Complementary and alternative medicine for cancer pain.  Further research should be conducted to examine **the effects of exercise on improving bridge symptoms** identified within or **between symptom clusters**.  Future research is warranted **to evaluate the efficacy of** psychosocial interventions within population clusters and examine their long-term effectiveness.  Undertake research to explore the **use of telemedicine** to **support chronic disease management**, **medication management**, **cancer screening, surveillance** for recurrence, **and disease prevention.**  More research is needed to **tailor eHealth interventions to yield stronger effects.**  More robust research recommended to emphasis the **lack of focus on specific cancer diseases** and to **facilitate direct comparisons between concurrent complementary and integrative medicine interventions.** | The mucosal tissue of children is different from that of adults, and **more cryotherapy interventions** are needed.  More clinical trials on the **effectiveness of cryotherapy** in **reducing oral mucositis severity** are needed.    Future trials are suggested to investigate the **effectiveness of Chinese herbal medicine** in managing common symptoms like pain, fatigue, and anorexia.  The **efficacy of Tai chi requires high-quality studies** to provide more convincing evidence.  Further rigorous randomized controlled trials are needed to **implement yoga.**  Studies are needed on **exercise interventions** to ensure **adherence to exercise regimes**, and to gain its **benefits on cancer recurrences.**  Further research should be conducted to examine **the effects of exercise on improving bridge symptoms** identified within or **between symptom clusters**.  Further evaluation of the **effects of pharmacologic interventions, aerobic exercise, Nordic walking, omega-3 fatty acids, and vitamin D is necessary**.  The safety of **psychostimulants** needs to be investigated in future trials for assessing effects of the long-term therapies.  Future randomized controlled trials are needed to **expand supportive care interventions for childhood cancer.**  P**sychosocial assessment** for **childhood cancer survivors** is highly recommended.  Larger randomised controlled trials are needed to identify **the effects of dietary supplements** on **cancer related fatigue.**  There is a need for more **well-designed randomized controlled trials** of **novel interventions** to improve patient-physician communication.  The effects of other **complementary and alternative medicine interventions** on **cancer-related symptoms** warrant further investigation.  Future large and well-designed randomized controlled trials are needed to evaluate the **effectiveness of complementary and alternative medicine for cancer pain.**  More robust research recommended to **facilitate direct comparisons between concurrent complementary and integrative medicine interventions.**  Future research should consider more **comprehensively applying electronic**, **wearable health technology-based**, **behavior change techniques,** and **theory-based interventions**.  Future research should more focus on **spirituality, social connections, body image and coping strategies** in cancer patients.  Investigating **web-based psychosocial intervention effects** in a **broader range of patient** populations is needed.  Future research is warranted **to evaluate the efficacy of psychosocial interventions** within population clusters and examine their long-term effectiveness.  In future research, **more attention should be given to the effects of eHealth interventions in relation to the disease stage.**  Studies are needed to **clarify the effect of e-health and educational interventions** on cancer related fatigue considering selected populations’ digital health literacy.  More research is needed to **tailor eHealth interventions to yield stronger effects.**  Undertake research to explore the **use of telemedicine** to **support chronic disease management**, **medication management**, **cancer screening, surveillance** for recurrence, **and disease prevention.** |
| **Recommendation for specific population/patients with cancer and specific diagnosis** | Future research should focus on testing these interventions [non-pharmacological interventions] on **specific cancer population clusters and trajectories**.  Future research should consider **individual cancer population clusters** and **direct comparisons between therapeutic options [pharmacological interventions]**.  More robust research recommended to emphasis the **lack of focus on specific cancer diseases** and to **facilitate direct comparisons between concurrent complementary and integrative medicine interventions.**  The effectiveness of patient navigation interventions **for patients with advanced or metastatic cancers and those in palliative care and end‐of‐life care settings** needs to be explored.  Future research should dedicate the **effectiveness of patient navigation** **in common cancers**, such as **prostate cancer, lung cancer, and melanoma**; rare cancer types; and **hematologic malignancies**.  Research into **indigenous populations worldwide** is needed to understand the **unique cultural factors** facing indigenous people.  Large randomized controlled trials in **patients with** **advanced lung cancer** are recommended **for finding the suitable exercise for** patients with **poor exercise acceptance**.  Studies are needed on interventions **to overcome barriers to exercise among breast cancer patients.**  Future studies should include breast cancer survivors with **BRCA1/2 gene mutations**, **women receiving tailored treatments, women from low socioeconomic background**s, breast cancer survivors with **multimorbidity and complex health care needs, late effects, as well as interventions targeting gender and sexually diverse breast cancer survivors.**  Future research should **concentrate on specific tumor types.**  Future studies should focalize attention on the different effects of physical activity **on breast cancer patients under therapy or under other conditions.** | Future research should focus on testing interventions on **specific cancer population clusters and trajectories**.  Future research should consider **individual cancer population clusters** and **direct comparisons between therapeutic options.**  Future research should **concentrate on specific tumor types.**  More robust research recommended to emphasis the **lack of focus on specific cancer diseases** and to **facilitate direct comparisons between concurrent complementary and integrative medicine interventions.**  The effectiveness of patient navigation interventions **for patients with advanced or metastatic cancers and those in palliative care and end‐of‐life care settings** needs to be explored.  Future research should dedicate the **effectiveness of patient navigation** **in common cancers**, such as **prostate cancer, lung cancer, and melanoma**; rare cancer types; and **hematologic malignancies**.  Research into **indigenous populations worldwide** is needed to understand the **unique cultural factors** facing indigenous people.  Large randomized controlled trials are recommended that **finding the suitable exercise for** patients with **poor exercise acceptance**.  Studies are needed on interventions **to overcome barriers to exercise in cancer patients.**  Future studies should include breast cancer survivors with **BRCA1/2 gene mutations**, **women receiving tailored treatments, women from low socioeconomic backgrounds**, breast cancer survivors with **multimorbidity and complex health care needs, late effects, as well as interventions targeting gender and sexually diverse breast cancer survivors.**  Future studies should focalize attention on the different effects of physical activity **on breast cancer patients under therapy or under other conditions.** |
| **Recommendations for diverse/ underrepresented population and diagnosis** | Appropriate interventions should be adopted according to the **characteristics of the different population.** [physical activity]  Future research needs to be conducted to enable an overall synthesis of the **effect of physical exercise** **on other cancer diagnoses.**  Future research should focus on mechanisms underlying physical activity effectiveness and on **underrepresented populations**.  Future research should **prioritize lower representation of cancers** other than prostate cancer and **patients with comorbidities and sarcopenia.**  Future studies should **examine the populations** covering **different cancer types, ages, languages, demographic groups, educational levels, and in remote, rural, or low-resource settings** over extended periods. [telemedicine]  Future research should consider the **effectiveness of interventions** **targeting people living beyond all types of cancer** and **with poor overall quality of life**. [non-pharmacological interventions]  Future research is needed to **examine the acceptability and effectiveness of exercise** **and psychosocial interventions** in **more diverse populations**.  Future research is needed to expand the understanding of **effective models of care in diverse cancer survivor populations** **including paediatric cancer survivors, adolescent and young adult survivor group**, **older adults**, and **a broader range of cancer types** as well as **advanced stages of the diseas**e. | Appropriate interventions should be adopted according to the **characteristics of the different population.**  Future studies should **examine the populations** covering **different cancer types, ages, languages, demographic groups, educational levels, and in remote, rural, or low-resource settings** over extended periods.  Future research is needed to expand the understanding of **effective models of care in diverse cancer survivor populations** **including paediatric cancer survivors, adolescent and young adult survivor group**, **older adults**, and **a broader range of cancer types** as well as **advanced stages of the diseas**e.  Future research should **prioritize lower representation of cancers.**  Future research should consider the **effectiveness of interventions** **targeting people living beyond all types of cancer** and **with poor overall quality of life**.  Future research is needed to **examine the acceptability and effectiveness of interventions** in **more diverse populations**.  Future research needs to synthesis of the **effect of interventions** **on other cancer diagnoses.** Future research should focus on mechanisms underlying interventions effectiveness and on **underrepresented populations.** |
| **Recommendations for validity of the tools** | The future **critical appraisal of the characteristics of the available tools for measuring cancer-related fatigue** could help to clarify domains cancer-related fatigue could be defined.  [physical exercise]  Future research should clarify the quality of the evidence regarding the **validity and reliability of the several tools to measure cancer-related fatigue** for providing a **theory-grounded base for clinicians and researchers.**  Future research evaluating models of care should be conducted **using validated tools to assess outcomes.**  In the assessment of outcomes, **validated tools and objective measures should be prioritized**.  Future trials should measure the most clinically relevant endpoint as the primary outcome **using a validated method,** to **ensure the utility of future clinical evidence. [palliative care]**  **Outcome measures that validated within the target population,** to support more coherent and robust evidence base should be used.  Future randomized controlled trials should measure patient outcomes comprehensively **using validated scales.** [Chinese herbal medicine] | The future **critical appraisal of the characteristics of the available tools for measuring cancer-related fatigue** could help to clarify domains cancer-related fatigue could be defined.  Future research should clarify the quality of the evidence regarding the **validity and reliability of the several tools to measure cancer-related fatigue** for providing a **theory-grounded base for clinicians and researchers.**  Future research evaluating models of care should be conducted **using validated tools to assess outcomes.**  In the assessment of outcomes, **validated tools and objective measures should be prioritized**.  **Outcome measures that validated within the target population,** to support more coherent and robust evidence base should be used.  Future randomized controlled trials should measure patient outcomes comprehensively **using validated scales.**  Future trials should measure outcomes **using a validated method,** to **ensure the utility of future clinical evidence.** |
| **Recommendations for transparency** | Further studies need to be conducted to **address pharmacological interventions specifically and transparently** for the treatment of cancer related fatigue.  There is a need for the publication **of more transparency descriptions of complex interventions**.  Future trials need to **transparent in the design** and **promote methodological strictness** throughout the trial process.  **Timing of assessment**, the **duration of the intervention**, and **longer follow-up periods** should address detailed transparently in further research. [Psychosocial interventions]  **To improve transparency within guideline development**, the views of the **funding body** and **competing interests** of the contributors should be disclosed.  ‘S**tandard’ or ‘usual’ care** included supportive cancer care, to manage the physical, psychological, social, and spiritual needs of patients, **needs to be** **transparent in future systematic reviews**. [musicbased interventions]  **A list of excluded studies should be provided** **transparently** as an independent appendix to journals **to facilitate readers’ understanding of the data selection process** and further improve the reliability of the review findings. | Further studies need to be conducted to **address interventions specifically and transparently**.  There is a need **of more transparency descriptions of complex interventions**.  Interventions and routine care to manage the physical, psychological, social, and spiritual needs of patients, **needs to be** **transparent in future systematic reviews**.  **Timing of assessment**, the **duration of the intervention**, and **longer follow-up periods** should address detailed transparently in further research.  Future trials need to **transparent in the design** and **promote methodological strictness** throughout the trial process.  **A list of excluded studies should be provided** **transparently** as an independent appendix to journals.  **To improve transparency within guideline development**, the views of the **funding body** and **competing interests** of the contributors should be disclosed. |
| **Recommendations for standardized approach** | Future policy research is needed to inform consensus **best‐practice standards,** including **standardized definitions and criteria** for cancer patient navigation.  **Standards and guidelines** should be developed to guide implementors on **the optimal approach to deploy telemedicine**.  Future research should determine the optimal delivery methods using **standardized telemedicine screening/assessing tools**, **interventions, and outcomes.** | Future policy research is needed to inform consensus **best‐practice standards,** including **standardized definitions and criteria** for cancer care management.  **Standards and guidelines** should be developed to guide implementors on **the optimal approach to deploy interventions**.  Future research should determine the optimal delivery methods using **standardized intervention assessing tools**, **and outcomes.** |
| **Recommendations for reporting standards** | Future reviews are recommended to **report according to the reporting standards** to improve the quality of evidence.  It is needed to report how **case management intervention** were conducted **follow standard reporting guidelines**, to provide recommendation for future research.  To provide more rigorous evidence [Chinese herbal medicine], future systematic reviews and trials must **adhere to high methodological and reporting standards.**  Further reviews should focus on **standardization in reporting** and aim to select randomized controlled trials of higher quality and lower risk of bias.  The **standardized reporting of the parameters of the different programs are** needed to enable the design of specific intervention protocols. [Psychosocial interventions] | Future reviews are recommended to **report according to the reporting standards** to improve the quality of evidence.  To provide more rigorous evidence, future systematic reviews and trials must **adhere to high methodological and reporting standards.**  Further reviews should focus on **standardization in reporting** and aim to select randomized controlled trials of higher quality and lower risk of bias.  It is needed to report how **intervention** were conducted **follow standard reporting guidelines**, to provide recommendation for future research.  The **standardized reporting of the parameters of the different interventions** are needed to enable the design of specific intervention protocols. |
| **Guidance/framework/ theory underpinning interventions, studies and methodological approaches** | Future **trials of educational interventions** [relating to cancer pain] should be based on **underlying theoretical model and consider factors** which might influence and modify the effect.  **The Medical Research Council framework** for complex interventions could be used to **guide future projects.**  Future systematic review should comply with the **PRISMA statement** that it is **useable for policy makers and clinicians.**  Researchers should use the Template for the intervention description and replication **(TIDieR) checklist.**  Further rigorous, comprehensive systematic reviews of meta-analysis and randomized controlled trials that **adhere to the guidelines are required to provide robust evidence for definitive conclusions.**  **Adhering to CONSORT guidelines, to report study outcomes transparently is recommended.**  Future studies should make use of **methodological quality guidelines when conducting systematic reviews and primary research.**  **Using standard guidelines, such as** Template for the intervention description and replication **(TIDieR) checklist** to help organize the case management intervention reporting is recommended.  Future trials should **adhere to CONSORT recommendations** for reporting. [palliative care]  Describing the **treatment protocol according to** Template for the intervention description and replication **TIDieR checklist**, so that the **procedure can be replicated in other trials. [palliative care]**  **Future randomized controlled trials should report trial implementation and results according to the CONSORT statement**. [Chinese herbal medicine]  Clinical trials are recommended to **report by CONSORT Statement and its extension to acupuncture trials (STRICTA)** to keep a high methodological quality.  **PRISMA statement should be used as guidance** in **preparing a normative report to improve the overall report quality** in future systematic reviews and meta-analysis.  Further research should focus on **frameworks’** **implementation** to deliver tailored interventions [physical exercise interventions].  It is recommended that **the development of best practice guidelines** including decision trees for selecting the most appropriate model of care for the individual cancer survivors, **implementation guides, and standardized outcomes for the evaluation.**  Developing a **comprehensive quality framework** for standardized evaluation of mHealth technologies in cancer survivors is recommended.  It is recommended to **adhere more strictly to the AMSTAR-2, PRISMA**, and **GRADE criteria** in future research. | Future **trials of interventions** should be based on **underlying theoretical model and consider factors** which might influence and modify the effect.  Further research should focus on **frameworks’** **implementation** to deliver tailored interventions.  Developing a **comprehensive quality framework** for standardized evaluation of interventions in cancer survivors is recommended.  Further rigorous, comprehensive systematic reviews of meta-analysis and randomized controlled trials that **adhere to the guidelines are required to provide robust evidence for definitive conclusions.**  Future studies should make use of **methodological quality guidelines when conducting systematic reviews and primary research.**  It is recommended that **the development of best practice guidelines** including decision trees for selecting the most appropriate model of care for the individual cancer survivors, **implementation guides, and standardized outcomes for the evaluation.**  **The Medical Research Council framework** for complex interventions could be used to **guide future projects.**  Future systematic review should comply with the **PRISMA statement** that it is **useable for policy makers and clinicians.**  **PRISMA statement should be used as guidance** in **preparing a normative report to improve the overall report quality** in future systematic reviews and meta-analysis.  Researchers should use the Template for the intervention description and replication **(TIDieR) checklist.**  **Using standard guidelines, such as** Template for the intervention description and replication **(TIDieR) checklist** to help organize the reporting of interventions is recommended.  Describing the **treatment protocol according to** Template for the intervention description and replication **TIDieR checklist**, so that the **procedure can be replicated in other trials.**  Future trials should **adhere to CONSORT recommendations** for reporting.  **Future randomized controlled trials should report trial implementation and results according to the CONSORT statement**.  Clinical trials are recommended to **report by CONSORT Statement** to keep a high methodological quality.  It is recommended to **adhere more strictly to the AMSTAR-2, PRISMA**, and **GRADE criteria** in future research. |
| **Recommendations for methodological needs** | It is recommended that future research should **not include only Cochrane reviews**, to **avoid overlooking** high-quality systematic reviews that potentially contain **unbiased and important recommendations**.  Higher-quality research is needed, including **subgroup analyses to determine optimal exercise types and settings.**  It is necessary that further trials should be conducted with **blinding, allocation concealment, and a sufficient sample size** to provide statistical power.  **Designing and reporting new clinical trials well** is essential to **provide sufficient information to replicate their intervention.**  Retrieving as much **comprehensive information from relevant original research** as possible in the writing of the review is needed.  A better study design, more complete outcome assessment, and exploration of potential subgroup effects are needed. [probiotics for chemotherapy and radiotherapy-related diarrhoea]  **High-quality systematic review** in which selection of high-quality studies is **combined with adequate methodology,** are needed to clarify the true efficacy of complementary therapies for cancer patients.  A more **robust conclusion** needs to be further assessed through **well-designed** and **well-conducted clinical trials**.  Conducting further research with **rigorous methodological designs** and **sufficient sample sizes** is necessary. [CAM on cancer-related fatigue]  High-quality studies **with larger sample sizes and longer follow-up times** are needed. [qigong intervention]  **Reporting reviews** **based on separate objectives, interventional studies, and type of studies** is recommended. [quality of life in breast cancer patients]  Future studies could **incorporate a wider range of research types.**  **Large‐scale longitudinal research with follow‐up measures is needed to identify long‐term effects,** especially on the lives of patients who have survived cancer or live with chronic illness. [nursing interventions]  Further evidence is needed from high-quality trials with large samples that **fully report rigorous methodological characteristics in the design stage.**  Future research is needed from high-quality trials that **estimate the optimal sample size** based on the existing research results, aiming to ensure that the **conclusions drawn from the research are valid.**  Future randomized controlled trials should use Chinese herbal medicine **placebo in the control group and ensure blinding of outcome assessment.**  **Methods of randomization, concealed allocation, and blinding** should be well conducted and reported in future studies. [Complementary and alternative medicine]  It is recommended to **register protocol before conducting, provide exclusion list if possible.**  Future research should **incorporate a large number of samples, long-term follow-up evaluations,** and **clearly defined targeted measurement indicators into the design**. [alternative exercise traditions]  **Large sample size** is needed in future studies. [effectiveness of exercise]  **Advancing the development of symptomics using symptom network analysis** is needed. [exercise on the symptoms in breast cancer]  Studies with **long-term follow-up are essential** to assess whether **positive impacts from intervention can be maintained** in the long term. [weight loss interventions in breast cancer survivors]  Future studies including **head-to-head comparisons**, which are fully **powered to conduct subgroup analyses**, are needed. [Web-based psychological interventions]  Well-designed randomised controlled trials in future is needed and should be considered a **stratification factor** **when analysing the results** in future systematic reviews. [complementary and alternative medicine on cancer-related fatigue]  Future systematic reviews should consider outcomes [quality of life, symptom control] **in their protocols**, planning to meta-analyse data from primary studies. [Systemic oncological treatments versus supportive care]  More **rigorously designed**, **high-quality, large-scale,** randomized controlled **trials will be required** in the future, to confirm the effects of resistance exercise.  **Improving quality and reporting** is recommended for future research.  It is recommended that **higher-quality randomized controlled trials** be conducted **for more conclusive and clinically applicable results**. [nutritional interventions for prevention of oral mucositis] | It is recommended that future research should **not include only Cochrane reviews**, to **avoid overlooking** high-quality systematic reviews that potentially contain **unbiased and important recommendations**.  Future studies could **incorporate a wider range of research types.**  Higher-quality research is needed, including **subgroup analyses.**  A better study design and exploration of potential subgroup effects are needed.  Future studies including **head-to-head comparisons**, which are fully **powered to conduct subgroup analyses**, are needed.  It is necessary that further trials should be conducted with **blinding, allocation concealment, and a sufficient sample size** to provide statistical power.  A more **robust conclusion** needs to be further assessed through **well-designed** and **well-conducted clinical trials**.  **Designing and reporting new clinical trials well** is essential to provide sufficient information to **replicate their intervention.**  Further evidence is needed from high-quality trials with large samples that **fully report rigorous methodological characteristics in the design stage.**  Future randomized controlled trials should use **placebo in the control group and ensure blinding of outcome assessment.**  **Methods of randomization, concealed allocation, and blinding** should be well conducted and reported in future studies.  Well-designed randomised controlled trials in future is needed and should be considered a **stratification factor** **when analysing the results** in future systematic reviews.  More **rigorously designed**, **high-quality, large-scale,** randomized controlled **trials will be required** in the future.  It is recommended that **higher-quality randomized controlled trials** be conducted **for more conclusive and clinically applicable results**.  Future research is needed from high-quality trials that **estimate the optimal sample size** based on the existing research results, aiming to ensure that the **conclusions drawn from the research are valid.**  Conducting further research with **rigorous methodological designs** and **sufficient sample sizes** is necessary.  High-quality studies **with larger sample sizes and longer follow-up times** are needed.  **Large‐scale longitudinal research with follow‐up measures is needed to identify long‐term effects,** especially on the lives of patients who have survived cancer or live with chronic illness.  Future research should **incorporate a large number of samples, long-term follow-up evaluations,** and **clearly defined targeted measurement indicators into the design.**  **Large sample size** is needed in future studies.  **Advancing the development of symptomics using symptom network analysis** is needed.  Studies with **long-term follow-up are essential** to assess whether **positive impacts from intervention can be maintained** in the long term.  Retrieving as much **comprehensive information from relevant original research** as possible in the writing of the review is needed.  **High-quality systematic review** in which selection of high-quality studies is **combined with adequate methodology,** are needed.  **Reporting reviews** **based on separate objectives, interventional studies, and type of studies** is recommended.  **Improving quality and reporting** is recommended for future research.  It is recommended to **register protocol before conducting, provide exclusion list if possible.**  Future systematic reviews should consider outcomes **in their protocols**, planning to meta-analyse data from primary studies. |
| **Recommendations for cost-analysis and effectiveness** | **Re economic evaluations** should be conducted to examine potential cost-effectiveness or cost-minimization following **implementation of telemedicine interventions.**  **Aerobic exercise** as a supportive intervention for reducing cancer-related fatigue is **cost-effective and should be popularized in clinical practice.**  Future studies are needed to **evaluate the cost-analysis and adverse events of exercise**.  **Economic evaluation** of the tools to justify investment in health services is needed. | **Economic evaluations** should be conducted to examine potential cost-effectiveness or cost-minimization following **implementation of interventions.**  **Cost-effective interventions should be popularized in clinical practice.**  Future studies are needed to **evaluate the cost-analysis and adverse events of supportive interventions.**  **Economic evaluation** of the tools to justify investment in health services is needed. |
| **Recommendations for symptom and adverse effect management** | **Cancer related fatigue should be assessed** on a regular basis in clinical settings to aid in the identification of effective therapies, treatments, and management.  It is essential for future randomized controlled trials and meta-analyses should **focus on the effects of exercise on fatigue**.  A strategy for controlling aromatase inhibitor-associated arthralgia should be **focused on symptom management.**  **Lifestyle changes** are recommended to **reduce risk of cardiotoxicity and osteoporosis** including addressing tobacco and alcohol use, managing weight, and increasing exercise.  More robust clinical studies are needed to establish the **best treatment for** **osteoradionecrosis.** | **Cancer related symptoms** should be assessed on a regular basis in clinical settings to aid in the identification of **effective therapies, treatments, and management.**  It is essential for future randomized controlled trials and meta-analyses should **focus on the effects of interventions on symptoms**.  A strategy for controlling should be **focused on symptom management.**  **Lifestyle changes** are recommended to **reduce risk of adverse effects.**  More robust clinical studies are needed to establish the **best treatment for** **adverse effects.** |
| **Recommendations for utilization of intervention/application of clinical setting** | Future studies should perform a **long-term follow-up** to **increase exercise intervention utilization** and **application in the clinical setting**.  Future studies should refine the relevant data of exercise to **facilitate clinical application** specifically frequency, intensity, duration, and type of exercise.  Future research should focus on **nursing interventions that save resources and are relatively easy to implement in daily practice.**  Studies should explore **the barriers to and facilitators of case management implementation** across various types of cancer patients at different stages.  Future randomized controlled trials and meta-analyses should focus on **improving breast cancer patients’ adherence to exercise protocols.** | Future research should focus on **interventions that save resources and are relatively easy to implement in daily practice.**  Studies should explore **the barriers to and facilitators of intervention implementation** across various types of cancer patients at different stages.  Future randomized controlled trials and meta-analyses should focus on **improving patients’ adherence to intervention protocols.**  Future studies should perform a **long-term follow-up** to **increase intervention utilization** and **application in the clinical setting**.  Future studies should refine the relevant data of intervention to **facilitate clinical application.** |
| **Recommendations for survivorship** | **Various aspects of survivorship** such as experiences of ongoing symptoms, financial toxicity, multi-morbidity, and psychological issues, including fear of cancer recurrence, mental health disorders, and stigma**,** need more investigation. | **Various aspects of survivorship** such as experiences of ongoing symptoms, financial toxicity, multi-morbidity, and psychological issues, including fear of cancer recurrence, mental health disorders, and stigma**,** need more investigation. |
| **Recommendations for patient necessities/ individualized needs** | Implementation of physical activity intervention outcomes should be collected before, during and after the treatment to present **data** **that can be personalized** according to the **patient’s necessities.**  **Considering the individual characteristics of the participants,** such as educational level, disease stage and treatment trajectory, when administering interventions to patients with cancer is recommended.  The **treatment choice** should be **based on stage and patient’s clinical osteoradionecrosis condition.**  Future studies [lymphoma] should consider **patient characteristics**, outcome measures, timing, mode and intensity of rehabilitation interventions.  The International Classification of Functioning, Disability and Health model can be used as a common framework **to help prioritize** **personalized goals** for patients with lymphoma to **set rehabilitation criteria**.  To establish a systematic way of providing **individualized rehabilitation,** further research is needed **to bridge the gap between rehabilitation research and clinical practice.** | Implementation of intervention outcomes should be collected before, during and after the treatment to present **data** **that can be personalized** according to the **patient’s necessities.**  **Considering the individual characteristics of the participants,** such as educational level, disease stage and treatment trajectory, when administering interventions to patients with cancer is recommended.  The **treatment choice** should be **based on stage and patient’s clinical condition.**  Future studies should consider **patient characteristics.**  The International Classification of Functioning, Disability and Health model can be used as a common framework **to help prioritize** **personalized goals** for patients.  To establish a systematic way of providing **individualized intervention,** further research is needed **to bridge the gap between various types of intervention research and clinical practice.** |
| **Recommendations for implementation/ clinical practice** | **Doctors and families should become familiar with the beneficial effects of exercise/physical activity programs** and their safe nature at all therapy phases.  Providers of **supportive care in cancer should name concrete personnel** who are **responsible for the dissemination of specific information needs**.  To **make tumor boards effective, professionals should consider them a critical part of their working** **agenda** and save time to prepare and attend tumor boards.  **Congruent time [tumor boards] should be dedicated** to the meetings in order to **avoid discussing many cases in a short amount of time.**  It is recommended that **mHealth technologies implement industry-standard data encryption** to ensure the security of private information.  A growing number of recent studies have explored **changes in symptom clusters or symptom networks** over time during **breast cancer adjuvant treatment**, their **clinical practice is needed**.  Implementation research studies is recommended to examine and **maximize effectiveness, adoption, implementation and maintenance outcomes** of the telemedicine strategies over time.  **Future implementation studies are needed** to provide evidence about the reach, uptake, fidelity, and scalability of the tools discussed in this overview. [Tools to facilitate communication with physician]  New psychological **intervention programs** should ensure they have the potential to be implemented **within clinical practice**, designed and tested for **accessibility in large-scale implementation**.  Interventions tested in trials should be **further tested in real-world settings, especially at the population level.** [models of cancer survivorship care]  **Leaflets and brochures should be provided in the language of the foreigner patients or** survivors affected by cancer**.** | **Leaflets and brochures should be provided in the language of the patients** or survivors affected by cancer**.**  **Doctors and families should become familiar with the beneficial effects of supportive interventions.**  Providers of supportive care in cancer should name **specific personnel** responsible for **disseminating particular information** to patients’ needs.  To **make cancer conference effective, professionals should consider them a critical part of their working** **agenda** and save time to prepare and attend conference.  **Conference time should be dedicated** to **avoiding discussing many cases in a short amount of time.**  It is recommended that **web-based interventions implement industry-standard data encryption** to ensure the security of private information.  Exploring **changes in symptom clusters or symptom networks** over time during **adjuvant treatment**, their **clinical practice is needed**.  Implementation research studies is recommended to examine and **maximize effectiveness, adoption, implementation and maintenance outcomes** of the intervention strategies over time.  **Future implementation studies are needed** to provide evidence about the reach, uptake, fidelity, and scalability of the tools discussed in this overview.  New **intervention programs** should ensure they have the potential to be implemented **within clinical practice**, designed and tested for **accessibility in large-scale implementation**.  Interventions tested in trials should be **further tested in real-world settings, especially at the population level.** |
| **Recommendations for migrant and minority population needs** | The information and **supportive care needs of migrants and ethnic minority cancer patients and survivors in Europe** should be **investigated in detail in future research**.  **Information needs regarding sensitive topics of body/image and sexuality should be investigated with caution** but not be avoided**.** [migrants and ethnic minority needs]  As **differences in information and supportive care needs** are present between migrant and ethnic minority cancer patients and survivors, **medical personnel should never overlook the individual characteristics of a patient**. | **Supportive care needs of migrants and ethnic minority cancer patients and survivors** should be **investigated in detail in future research**.  **Information needs regarding sensitive topics of body image and sexuality should be investigated.**  As **differences in information and supportive care needs** are present between migrant and ethnic minority cancer patients and survivors, **medical personnel should never overlook the individual characteristics of a patient**. |
| **Recommendations for comparing effectiveness of interventions** | Researchers should **adopt a comparative effectiveness approach and design trials** that allow real-world evaluation of acupuncture and related therapies.  Future controlled trials should compare the **benefits of specific exercise regimes** **compared** with **usual care in a** population in real‐world settings.  Further research is needed to **examine comparative effectiveness of intervention modalities** such as group versus individual, monodimensional versus multidimensional or multidisciplinary. [non-pharmacological interventions]  **Future comparative effectiveness of interventions** research should pay attention to improving the reporting and methodological quality of trials. | Researchers should **adopt a comparative effectiveness approach and design trials** that allow real-world evaluation of interventions.  Future controlled trials should compare the **benefits of specific intervention implementations** **compared** with **usual care in a** population in real‐world settings.  Further research is needed to **examine comparative effectiveness of intervention modalities.**  **Future comparative effectiveness of interventions** research should pay attention to improving the reporting and methodological quality of trials. |
| **Recommendations for higher level of evidence** | More well designed and large randomized controlled trials are needed **to provide a higher level of evidence** to confirm the role of manual lymphatic drainage in complete decongestive therapy. [breast cancer related lymphoedema]  **Improving the level of evidence** of the included systematic reviews, more original studies with rigorous study designs and detailed descriptions of the intervention protocols such as, type, frequency, intensity, and duration of the exercise are necessary.  Rigorous randomized controlled trials and systematic reviews are needed **to provide high-quality evidence for the specificity of exercise interventions.**  Future research is needed for **innovative methods to generate high-quality evidence.**  There is a need for high-quality randomized controlled trials with a large sample size **to provide high-quality evidence.** [probiotics for chemotherapy and radiotherapy-related diarrhoea] | More well designed and large randomized controlled trials are needed **to provide a higher level of evidence** to confirm the supportive interventions.  **Improving the level of evidence** of the included systematic reviews, more original studies with rigorous study designs and **detailed descriptions of the intervention protocol**s are necessary.  Rigorous randomized controlled trials and systematic reviews are needed **to provide high-quality evidence for the specificity of interventions.**  There is a need for high-quality randomized controlled trials with a large sample size **to provide high-quality evidence.** |
| **Recommendations for preventing bias** | Funding sources should be clearly declared in future publications to **help readers determine whether funding bias existed.**  **To prevent publication bias,** it is recommended that all clinical trials protocols on the topic should register with a recognized platform.  **Strictly controlling bias** in future original research is essential.  Future studies should **reduce the risk of methodological bias**.  Indicating a more rigorous design and **evaluation** is needed **to avoid blinding bias.** [case management]  Future randomized controlled trials should **reduce publication bias by releasing randomized controlled trials protocols on trial registries. [**Chinese herbal medicine]  Future studies should focus on developing high‐quality studies with longer follow‐ups and **reducing** **biased results.** [Mindfulness‐Based Interventions]  To achieve a comprehensive literature search, future systematic reviews should also search **gray literature, to retrieval websites to minimize publication bias.** | Funding sources should be clearly declared in future publications to reduce **funding bias.**  **To prevent publication bias,** it is recommended that all clinical trials protocols on the topic should register with a recognized platform.  Future randomized controlled trials should **reduce publication bias by releasing randomized controlled trials protocols on trial registries.**  **Strictly controlling bias** in future original research is essential.  Future studies should **reduce the risk of methodological bias**.  Indicating a more rigorous design and **evaluation** is needed **to avoid blinding bias.**  Future studies should focus on developing high‐quality studies with longer follow‐ups and **reducing** **biased results.**  To achieve a comprehensive literature search, future systematic reviews should also search **gray literature, to retrieval websites to minimize publication bias.** |
| **Recommendations for addressing evidence gaps** | Future systematic reviews should **explicitly report evidence gaps** in primary research.  Future studies should **prioritize robust primary studies to address gaps in the literature** for outcomes in the domains of health promotion**, chronic conditions, clinical structure, and decision-making.** | Future systematic reviews should **explicitly report evidence gaps** in primary research.  Future studies should **prioritize robust primary studies to address gaps in the literature** for outcomes in the domains of health promotion**, chronic conditions, clinical structure, and decision-making.** |
| **Recommendations for enhancing quality of life** | **Future larger randomized controlled trials and meta-analyses are needed**, to provide paediatric cancer patients, survivors and their families with the **best possible quality of life**.  More trials should be conducted by setting and subsequently **studying the same criteria**, such as weight loss, pain degree or oral mucositis duration, **concerning the participants' quality of life during their treatment.** [honey intervention for oral mucositis] | **Future larger randomized controlled trials and meta-analyses are needed**, to provide cancer patients, survivors and their families with the **best possible quality of life**.  More trials should be conducted by setting and subsequently **studying the same criteria**, such as weight loss, pain degree or symptom duration, **concerning the participants' quality of life during their treatment.** |

# Stage 3. Iteratively grouping codes and creation of final codes

| Heading | New codes | Final codes |
| --- | --- | --- |
| **Recommendations for future research efforts** | Future **research** efforts are needed to develop a **body of evidence** that is adequate to support **policy development**.  Further research is needed to **establish firm evidence and further recommendation**.  Future trials should be based on **underlying theoretical model** and consider factors which might influence and modify the effect of interventions.  Due to very low certainty of the current evidence in some areas randomized controlled trials are required toto address this uncertainty.  More **rigorous randomized controlled trials** of non-pharmacological interventions are recommended.  There is needed for **fully powered randomized controlled trials**, to enable more **robust conclusions about the efficacy** of **web-based oncology interventions.**  Future studies **with higher methodological rigor** should be conducted on **health promotion strategies.**  More studies with **homogeneous samples of cancer patients** are needed.  Future primary studies should explicitly report patient-important outcomes to **provide useful data for evidence syntheses** and **clinical practice guidelines**.  Well reported **observational studies and randomised controlled trials** are needed to clarify the presence of **short and long term toxicities of herbal medicine**.  Future research should **employ the incidence of adverse events as the main evaluation index of the study.**  Future trials need to **assess how to sustain intervention effects** over a longer **follow‐up period**.  Future research is needed for agreement on the **best timing for assessments** relative to surgery and neoadjuvant/adjuvant therapies. (clarify)  Consensus around a **core outcome set to measure** impact on **psychological morbidity** is also required for use in both unimodal **psychological prehabilitation interventions** as well as multimodal approaches. Agreement around **optimal timing of assessments** in relation **to surgery and neoadjuvant/ adjvant therapies** is also warranted. | Future **research** efforts are needed to develop a **body of evidence and further recommendations** that is adequate to support **policy development**.  Future trials should be based on **underlying theoretical model(s)** and consider factors which might influence and modify the effect of interventions.  More **rigorous randomized controlled trials** of non-pharmacological interventions; **web-based oncology interventions; health promotion strategies** to enable more **robust conclusions about the efficacy** of interventions are recommended.  Due to very low certainty of the current evidence in some areas (e.g. efficacy of cryotherapy) more randomized controlled trials are required to generate results to address this uncertainty.  To provide useful data for evidence syntheses and clinical practice guidelines, future well reported **observational studies and randomised controlled trials should:**   - be **fully powered.** - include **homogeneous samples of cancer** patients to allow for more definitive conclusions - explicitly report patient-important outcomes. - clarify the presence of **short- and long-term toxicities of interventions (e.g. herbal medicine).** - **employ the incidence of adverse events as the main evaluation index**. - **assess how to sustain intervention effects** over a longer **follow‐up period**. |
| **Recommendations for workforce shortages** | Investment in time and resources in the training of a competent care workforce is a facilitator in addressing the **workforce shortages**.  Volunteers can support the health of cancer patients and overcoming **workforce shortages**. | **Investment in time and resources** in training a competent care workforce and **supporting volunteers** for the health of cancer patients is a facilitator in **addressing the workforce shortages.** |
| **Funding healthcare and policy recommendations** | Recommended that policy makers **collaborate** with national and international organisations to secure **funding** for improving **health care provision.** For policy development, **assessing the country readiness** for the provision and integration of evidence-based care is recommended.  **Future research** efforts are needed to develop a **body of evidence** that is adequate to support **policy development**. | Policymakers **should collaborate** with national and international organizations **to secure funding** for **improving healthcare provision**.  **Assessing the country's readiness** for the provision and integration of **evidence-based care** and developing **a body of evidence** that is adequate to support **policy development** are recommended. |
| **Recommendations for accessing to services** | Integrating palliative care into **primary care services,** to improve **access to palliative care** for patients living in remote areas is recommended.  Patients with **limited treatment options** or persistent frailty despite rehabilitative attempts should be **offered palliative care services.**  Evaluating the use of different technological platforms is recommended to **provide telemedicine services.** | Integrate palliative care into **primary care services,** to improve **access to palliative care** for patients living in remote areas.  Access to palliative care is needed for patients with **limited treatment options** or **persistent frailty** despite rehabilitative attempts.  Evaluating the use of **different technological platforms** is recommended to **provide telemedicine services.** |
| **Recommendations for healthcare professionals** | **Health care professionals** need to investigate **communication competencies** between patient and healthcare providers.  **Helping healthcare professionals** by enabling individualization in clinical practice requires a more comprehensive approach to **individualized rehabilitation**. | Need to investigate **communication competencies** between patient and healthcare providers.  **Helping healthcare professionals** by enabling individualization in clinical practice requires a more comprehensive approach to **individualized rehabilitation**. (Exercise and yoga likewise have shown effects on anxiety, depression and QoL [42, 52]. Anxiety was also reduced by CBT, mindfulness-based stress reduction and massage [28, 34, 36]. This variety of interventions with positive outcomes indicates that it should be possible to optimize rehabilitation through evidence-based interventions. However, to enable this HCP need tools to identify patients’ needs and knowledge, both in how to do this and about available and effective interventions. Therefore, taking the next step from evaluating the effect of narrow rehabilitation studies on specific outcomes, helping HCP by identifying a knowledge base that could be used to enable individualization in clinical practice, requires a more comprehensive approach to individualized rehabilitation.) |
| **Recommendations for patient voice, PPI, and patient experiences** | **Patients’/family voice** need **to address the priorities** in low- and middle-income countries.  Non-pharmacologic interventions should be integrated into **patients’ experiences**, recognizing the impairing effects of cancer treatments, home and working life, and patient’s physical and psychological needs.  Data is needed on the **perspectives of all stakeholders,** to improve **intervention development.** | **Patients’/family voice is** needed **to address the priorities** in low- and middle-income countries.  Non-pharmacologic interventions should be integrated into **patients’ experiences and perspectives of all stakeholders,** recognizing the impairing effects of cancer treatments, home and working life, and patient’s physical and psychological needs.    Data is needed on the **perspectives of all stakeholders,** to improve **intervention development.** |
| **Recommendations for outcome measures** | It might also be desirable **to assess alternative outcome measures**, for example use of out of hours and emergency services.  The difference between **objective and subjective outcome measures** should be further investigated to understand **patients’ interpretations** of their conditions.  More studies on **outcome measurement instruments should be explored**, with attention to early detection of side-effects.  **More physical measurement indicators** for evaluating are expected.  Consensus around a **core outcome set to measure psychological morbidity impact** is required.  Future research should use **ecological transient assessment to dynamically measure symptoms**.  Future trials are suggested to adopt **more specific quality of life measurement tool** such as the Short Form 36 questionnaire, and the European Organisation for Research and Treatment of Cancer Quality of Life Questionnaire-core. | **Assessing alternative outcome measures**, and the difference between **objective and subjective outcome measures** should be further investigated to understand **patients’ interpretations** of their conditions, and to enable early detection of side-effects.  Consensus around a **core outcome set to measure psychological morbidity impact** is required.  Future research should use **ecological transient assessment to dynamically measure symptoms**.  Future trials are suggested to adopt **more specific quality of life measurement tool** such as the Short Form 36 questionnaire, and the European Organisation for Research and Treatment of Cancer Quality of Life Questionnaire-core. |
| **Recommendations for future intervention research** | The mucosal tissue of children is different from that of adults, and **more cryotherapy interventions** are needed.  More clinical trials on the **effectiveness of cryotherapy** in **reducing oral mucositis severity** are needed.    Future trials are suggested to investigate the **effectiveness of Chinese herbal medicine** in managing common symptoms like pain, fatigue, and anorexia.  The **efficacy of Tai chi requires high-quality studies** to provide more convincing evidence.  Further rigorous randomized controlled trials are needed to **implement yoga.**  Studies are needed on **exercise interventions** to ensure **adherence to exercise regimes**, and to gain its **benefits on cancer recurrences.**  Further research should be conducted to examine **the effects of exercise on improving bridge symptoms** identified within or **between symptom clusters**.  Further evaluation of the **effects of pharmacologic interventions, aerobic exercise, Nordic walking, omega-3 fatty acids, and vitamin D is necessary**.  The safety of **psychostimulants** needs to be investigated in future trials for assessing effects of the long-term therapies.  Future randomized controlled trials are needed to **expand supportive care interventions for childhood cancer.**  **A yearly psychosocial assessment** for **childhood cancer survivors** is highly recommended.  There is a need for more **well-designed randomized controlled trials** of **novel interventions** to improve patient-physician communication.  The effects of other **complementary and alternative medicine interventions** on **cancer-related symptoms** warrant further investigation.  Future large and well-designed randomized controlled trials are needed to evaluate the **effectiveness of complementary and alternative medicine for cancer pain.**  More robust research recommended to emphasis to **facilitate direct comparisons between concurrent complementary and integrative medicine interventions.**  Future research should consider more **comprehensively applying electronic**, **wearable health technology-based**, **behavior change techniques,** and **theory-based interventions**.  Future research should more focus on **spirituality, social connections, body image and coping strategies** in cancer patients.  Investigating **web-based psychosocial intervention effects** in a **broader range of patient** populations is needed.  Future research is warranted **to evaluate the efficacy of psychosocial interventions** within population clusters and examine their long-term effectiveness.  In future research, **more attention should be given to the effects of eHealth interventions in relation to the disease stage.**  Additional studies are needed to **clarify the effect of e-health and educational interventions** on cancer related fatigue considering selected populations’ digital health literacy.  More research is needed to **tailor eHealth interventions to yield stronger effects.**  Undertake research to explore the **use of telemedicine** to **support chronic disease management**, **medication management**, **cancer screening, surveillance** for recurrence, **and disease prevention.** | The mucosal tissue of children is different from that of adults, and **more cryotherapy interventions** are needed in **reducing oral mucositis.**  Future trials are suggested to investigate the **effectiveness of Chinese herbal medicine** in managing common symptoms like pain, fatigue, and anorexia.  Further rigorous randomized controlled trials on the **efficacy of Tai Chi, yoga, and exercise interventions** in improving symptoms identified within or between symptom clusters, and their impact **on cancer recurrences**, are needed.  Further evaluation of the long-term **effects of pharmacologic interventions, dietary supplements, psychostimulants, aerobic exercise, Nordic walking, omega-3 fatty acids, and vitamin D** is necessary.  Future randomized controlled trials are needed to **expand supportive care interventions, and psychosocial assessment for childhood cancer.**  There is a need for more well-designed randomized controlled trials of novel interventions to **improve patient-physician communication**.  More robust research is recommended to facilitate direct comparisons between concurrent **complementary and integrative medicine interventions** and their effects on cancer-related symptoms.    Future research should consider more comprehensively **applying electronic**, **wearable health technology-based**, **behavior change techniques,** and **theory-based interventions**.  Future research should focus on **spirituality, social connections, body image and coping strategies** in cancer patients.  Investigating long-term effectiveness of **psychosocial interventions and web-based psychosocial interventions** in a **broader range of patient** populations is needed.  The use of **telemedicine, tailored eHealth interventions, and educational interventions** for cancer-related fatigue, considering selected populations' digital health literacy, is necessary to **support chronic disease management, medication management, cancer screening, surveillance for recurrence, and disease prevention**. |
| **Recommendation for specific population/patients with cancer and specific diagnosis** | Future research should focus on testing interventions on **specific cancer population clusters and trajectories**.  Future research should consider **individual cancer population clusters** and **direct comparisons between therapeutic options.**  Future research should **concentrate on specific tumor types.**  The effectiveness of patient navigation interventions **for patients with advanced or metastatic cancers and those in palliative care and end‐of‐life care settings** needs to be explored.  Future research should dedicate the **effectiveness of patient navigation** **in common cancers**, such as **prostate cancer, lung cancer, and melanoma**; rare cancer types; and **hematologic malignancies**.  Research into **indigenous populations worldwide** is needed to understand the **unique cultural factors** facing indigenous people.  Large randomized controlled trials are recommended that **finding the suitable exercise for** patients with **poor exercise acceptance**.  Studies are needed on interventions **to overcome barriers to exercise in cancer patients.**  Future studies should include breast cancer survivors with **BRCA1/2 gene mutations**, **women receiving tailored treatments, women from low socioeconomic backgrounds**, breast cancer survivors with **multimorbidity and complex health care needs, late effects, as well as interventions targeting gender and sexually diverse breast cancer survivors.**  Future studies should focalize attention on the different effects of physical activity **on breast cancer patients under therapy or under other conditions.** | Future research should focus on testing interventions on **specific tumor types** and include direct comparisons between therapeutic options **amongst specific cancer population clusters and trajectories.**  Research into **indigenous populations worldwide** is needed to understand the **unique cultural factors** facing indigenous people.  Large randomized controlled trials are recommended **to overcome barriers to exercise and** to **find suitable exercises for** patients with **poor exercise acceptance**.  Future studies should include breast cancer survivors with **BRCA1/2 gene mutations**, **women receiving tailored treatments, and women from low socioeconomic backgrounds, their complex health care needs, and those experiencing late effects.** |
| **Recommendations for diverse/ underrepresented population and diagnosis** | Appropriate interventions should be adopted according to the **characteristics of the different population.**  Future studies should **examine the populations** covering **different cancer types, ages, languages, demographic groups, educational levels, and in remote, rural, or low-resource settings** over extended periods.  Future research is needed to expand the understanding of **effective models of care in diverse cancer survivor populations** **including paediatric cancer survivors, adolescent and young adult survivor group**, **older adults**, and **a broader range of cancer types** as well as **advanced stages of the diseas**e.  Future research should **prioritize lower representation of cancers.**  Future research should consider the **effectiveness of interventions** **targeting people living beyond all types of cancer** and **with poor overall quality of life**.  Future research is needed to **examine the acceptability and effectiveness of interventions** in **more diverse populations**.  Future research needs to synthesis of the **effect of interventions** **on other cancer diagnoses.**  Future research should focus on mechanisms underlying interventions effectiveness and on **underrepresented populations.** | Future studies should:   - **examine the populations** covering **different cancer types, ages, languages, demographic groups, educational levels, and in remote, rural, or low-resource settings.** - **prioritize inclusion of patients traditionally underrepresented in studies.** - consider the a**cceptability and effectiveness of interventions** **targeting people living beyond all types of cancer** and **with poor overall quality of life**. - expand the understanding of **effective models of care in diverse cancer survivor populations** **including paediatric cancer survivors, adolescent and young adult survivor group**, **older adults**, and **a broader range of cancer types** as well as **advanced stages of the diseas**e. |
| **Recommendations for validity of the tools** | The future **critical appraisal of the characteristics of the available tools for measuring cancer-related fatigue** could help to clarify domains cancer-related fatigue could be defined.  Future research should clarify the quality of the evidence regarding the **validity and reliability of the several tools to measure cancer-related fatigue** for providing a **theory-grounded base for clinicians and researchers.**  Future research evaluating models of care should be conducted **using validated tools to assess outcomes.**  In the assessment of outcomes, **validated tools and objective measures should be prioritized**.  **Outcome measures that validated within the target population,** to support more coherent and robust evidence base should be used.  Future randomized controlled trials should measure patient outcomes comprehensively **using validated scales.**  Future trials should measure outcomes **using a validated method,** to **ensure the utility of future clinical evidence.** | The future **critical appraisal of the characteristics of the available tools for measuring cancer-related fatigue** could help to clarify domains cancer-related fatigue could be defined, to provide a **theory-grounded base for clinicians and researchers.**  Future research is recommended to clarify the quality of the evidence regarding the **validity and reliability of tools and e measures** in the assessment of outcomes such as fatigue **within the target population,** to support a more coherent and robust evidence base. |
| **Recommendations for transparency** | Further studies need to be conducted to **address interventions specifically and transparently**.  There is a need **of more transparency descriptions of complex interventions**.  Interventions and routine care to manage the physical, psychological, social, and spiritual needs of patients, **needs to be** **transparent in future systematic reviews**.  **Timing of assessment**, the **duration of the intervention**, and **longer follow-up periods** should address detailed transparently in further research.  Future trials need to **transparent in the design** and **promote methodological strictness** throughout the trial process.  **To improve transparency within guideline development**, the views of the **funding body** and **competing interests** of the contributors should be disclosed. | There is a need **of more transparency in the description of complex interventions** and routine care**, trial design, adherence to trail protocol and intervention,** including timing of assessment, the duration of the intervention.  Future reviews should provide a **list of excluded studies** as an independent appendix to journals.  **To improve transparency within guideline development**, the views of the **funding body** and **competing interests** of the contributors should be disclosed. |
| **Recommendations for standardized approach** | Future policy research is needed to inform consensus **best‐practice standards,** including **standardized definitions and criteria** for cancer care management.  **Standards and guidelines** should be developed to guide implementors on **the optimal approach to deploy interventions**.  Future research should determine the optimal delivery methods using **standardized intervention assessing tools**, **and outcomes.** | Future policy research is needed to inform consensus **best‐practice standards,** including **standardized definitions and criteria** for cancer care management.  **Standards and guidelines** should be developed to guide implementors on **the optimal approach to deploy interventions, and delivery methods**.  Need for **standardized tools and outcomes** in future research. |
| **Recommendations for reporting standards** | Future reviews are recommended to **report according to the reporting standards** to improve the quality of evidence.  To provide more rigorous evidence, future systematic reviews and trials must **adhere to high methodological and reporting standards.**  Further reviews should focus on **standardization in reporting** and aim to select randomized controlled trials of higher quality and lower risk of bias.  It is needed to report how **intervention** were conducted **follow standard reporting guidelines**, to provide recommendation for future research.  The **standardized reporting of the parameters of the different interventions** are needed to enable the design of specific intervention protocols. | Future reviews should:   - **adhere to high methodological and reporting standards, use standard reporting guidelines** - comply with the **PRISMA statement so** that it is **useable for policy makers and clinicians.** - **use standardized reporting of the parameters of the different interventions.** - select randomized controlled trials of higher quality and lower risk of bias to provide more rigorous evidence. |
| **Recommendations for guidance/framework/ theory underpinning interventions, studies and methodological approaches** | Future **trials of interventions** should be based on **underlying theoretical model and consider factors** which might influence and modify the effect.  Further research should focus on **frameworks’** **implementation** to deliver tailored interventions.  Developing a **comprehensive quality framework** for standardized evaluation of interventions in cancer survivors is recommended.  Further rigorous, comprehensive systematic reviews of meta-analysis and randomized controlled trials that **adhere to the guidelines are required to provide robust evidence for definitive conclusions.**  Future studies should make use of **methodological quality guidelines when conducting systematic reviews and primary research.**  It is recommended that **the development of best practice guidelines** including decision trees for selecting the most appropriate model of care for the individual cancer survivors, **implementation guides, and standardized outcomes for the evaluation.**    **The Medical Research Council framework** for complex interventions could be used to **guide future projects.**  Future systematic review should comply with the **PRISMA statement** that it is **useable for policy makers and clinicians.**  **PRISMA statement should be used as guidance** in **preparing a normative report to improve the overall report quality** in future systematic reviews and meta-analysis.  Researchers should use the Template for the intervention description and replication **(TIDieR) checklist.**  **Using standard guidelines, such as** Template for the intervention description and replication **(TIDieR) checklist** to help organize the reporting of interventions is recommended.  Describing the **treatment protocol according to** Template for the intervention description and replication **TIDieR checklist**, so that the **procedure can be replicated in other trials.**  Future trials should **adhere to CONSORT recommendations** for reporting.  **Future randomized controlled trials should report trial implementation and results according to the CONSORT statement**.  Clinical trials are recommended to **report by CONSORT Statement** to keep a high methodological quality.  It is recommended to **adhere more strictly to the AMSTAR-2, PRISMA**, and **GRADE criteria** in future research. | Future **trials of interventions** should:   - be based on **underlying theoretical model and consider factors** which might influence and modify the effect of the intervention. - focus on **frameworks’ implementation** to deliver tailored interventions. (Literature encompasses multiple systematic reviews and meta-analyses on the efficacy of physical exercise for improving cancer-related fatigue. However, understanding whether physical exercise in a specific cancer population could be more effective than other cancer populations is still unclear, and knowledge remains fragmented. For this reason, providing a clear understanding of the efficacy of physical exercise on specific cancer populations in terms of tumor type could be strategic for addressing tailored and targeted interventions and avoid healthcare wastes.) - use a **comprehensive quality framework** for standardized evaluation of interventions in cancer survivors   Future reviews that **adhere to methodological quality guidelines, and the development of best practice guidelines are needed.**  Decision trees for selecting the most appropriate **model of care for individual cancer survivors**, along with implementation guides and standardized outcomes for evaluation, are required.  **The Medical Research Council framework** for complex interventions is recommended to **guide future projects.**  Future systematic review should use **PRISMA statement as guidance** in **preparing a normative report to improve the overall report quality.**  It is recommended to describe the **treatment protocol according to the TIDieR checklist (**Template for the intervention description and replication), to help organize the reporting of interventions and **facilitate replication in other trials.**  Future trials should **report implementation and results according to the CONSORT statement,** to provide a high methodological quality.  It is recommended to **adhere more strictly to the AMSTAR-2, PRISMA**, and **GRADE criteria** for quality appraisal in future reviews. |
| **Recommendations for methodological needs** | It is necessary that further trials should be conducted with **blinding, allocation concealment, and a sufficient sample size** to provide statistical power.  A more **robust conclusion** needs to be further assessed through **well-designed** and **well-conducted clinical trials**.  **Designing and reporting new clinical trials well** is essential to provide sufficient information to **replicate their intervention.**  Further evidence is needed from high-quality trials with large samples that **fully report rigorous methodological characteristics in the design stage.**  Future randomized controlled trials should use **placebo in the control group and ensure blinding of outcome assessment.**  **Methods of randomization, concealed allocation, and blinding** should be well conducted and reported in future studies.  Well-designed randomised controlled trials in future is needed and should be considered a **stratification factor** **when analysing the results** in future systematic reviews.  More **rigorously designed**, **high-quality, large-scale,** randomized controlled **trials will be required** in the future.  It is recommended that **higher-quality randomized controlled trials** be conducted **for more conclusive and clinically applicable results**.  Future research is needed from high-quality trials that **estimate the optimal sample size** based on the existing research results, aiming to ensure that the **conclusions drawn from the research are valid.**  Conducting further research with **rigorous methodological designs** and **sufficient sample sizes** is necessary.  High-quality studies **with larger sample sizes and longer follow-up times** are needed.  **Large‐scale longitudinal research with follow‐up measures is needed to identify long‐term effects,** especially on the lives of patients who have survived cancer or live with chronic illness.  Future research should **incorporate a large number of samples, long-term follow-up evaluations,** and **clearly defined targeted measurement indicators into the design.**  **Large sample size** is needed in future studies.  **Advancing the development of symptomics using symptom network analysis** is needed.  Studies with **long-term follow-up are essential** to assess whether **positive impacts from intervention can be maintained** in the long term.  Retrieving as much **comprehensive information from relevant original research** as possible in the writing of the review is needed.  It is recommended that future research should **not include only Cochrane reviews**, to **avoid overlooking** high-quality systematic reviews that potentially contain **unbiased and important recommendations**.  Future studies/reviews could **incorporate a wider range of research types.**  Higher-quality research is needed, including **subgroup analyses.**  A better study design and **exploration of potential subgroup effects** are needed.  Future studies including **head-to-head comparisons**, which are fully **powered to conduct subgroup analyses**, are needed.  **High-quality systematic review** in which selection of high-quality studies is **combined with adequate methodology,** are needed.  **Reporting reviews** **based on separate objectives, interventional studies, and type of studies** is recommended.  **A list of excluded studies should be provided** **transparently** as an independent appendix to journal.  **Improving quality and reporting** is recommended for future research.  It is recommended to **register protocol before conducting, provide exclusion list if possible.**  Future systematic reviews should consider outcomes **in their protocols**, planning to meta-analyse data from primary studies. | Future trials, to provide higher quality, should:   - conduct **blinding, allocation concealment, and a sufficient sample size.** - be **well-designed and well-reported** to provide **sufficient information to replicate** their interventions. - use **placebo in the control group.** - **estimate the optimal sample size** based on the existing research results, aiming to ensure that the **conclusions drawn from the research are valid.**   Future reviews should:   - conduct **large‐scale longitudinal research with follow‐up measures,** especially on the lives of patients who have survived cancer or live with chronic illness. - incorporate **a large number of samples, long-term follow-up evaluations,** and **clearly defined targeted measurement** indicators into the design. - assess whether **positive impacts from intervention can be maintained** in the long term. - retrieve **comprehensive information from relevant original research.** - incorporate **a wider range of research types** and they should not include **only Cochrane reviews**, to **avoid overlooking** high-quality systematic reviews that potentially contain **unbiased and important recommendations**. - develop **symptomics using symptom network analysis.** - **explore subgroup analyses and potential subgroup effects through head-to-head comparisons.** - report reviews **based on separate objectives, interventional studies, and type of studies.** - provide **exclusion list, and** outcomes **in their protocols.** |
| **Recommendations for cost-analysis and effectiveness** | **Economic evaluations** should be conducted to examine potential cost-effectiveness or cost-minimization following **implementation of interventions.**  **Cost-effective interventions should be popularized in clinical practice.**  Future studies are needed to **evaluate the cost-analysis and adverse events of supportive interventions.**  **Economic evaluation** of the tools to justify investment in health services is needed.  Future research should expand **robust economic evaluations** to **provide evidence for health systems to fund** and promote the transition to alternative models. | **Economic evaluation and cost-analysis and adverse events** should be evaluated to examine potential cost-effectiveness or cost-minimization **following implementation** of interventions**,** and **cost-effective interventions should be popularized** in clinical practice.  **Robust economic evaluation** of intervention/tools are needed to justify investment in health services.  Evidence for health systems to fund and **promote the transition to alternative models of interventions is needed.** |
| **Recommendations for symptom and adverse effect management** | **Cancer related symptoms** should be assessed on a regular basis in clinical settings to aid in the identification of **effective therapies, treatments, and management.**  It is essential for future randomized controlled trials and meta-analyses should **focus on the effects of interventions on symptoms**.  A strategy for controlling should be **focused on symptom management.**  **Lifestyle changes** are recommended to **reduce risk of adverse effects.**  More robust clinical studies are needed to establish the **best treatment for** **adverse effects.** | It is recommended that randomized controlled trials, reviews and meta-analyses should focus on the **effects of interventions on symptoms and assess symptoms** regularly **in clinical settings** to aid in **identifying effective therapies, treatments, and management strategies.**  More robust clinical studies are recommended to establish the **best treatment for** **adverse effects and lifestyle changes** are recommended to **reduce risk of adverse effects.** |
| **Recommendations for utilization of intervention/application of clinical setting** | Future research should focus on **interventions that save resources and are relatively easy to implement in daily practice.**  Studies should explore **the barriers to and facilitators of intervention implementation** across various types of cancer patients at different stages.  Future randomized controlled trials and meta-analyses should focus on **improving patients’ adherence to intervention protocols.**  Future studies should perform a **long-term follow-up** to **increase intervention utilization** and **application in the clinical setting**.  Future studies should refine the relevant data of intervention to **facilitate clinical application.**  (Given the difficulties we encountered in extracting data on **the specific modalities of exercise** (frequency, intensity, duration, and type), future studies should **refine the relevant data to facilitate clinical application**.) | Future research should:   - **Focus on interventions that save resources and are relatively easy to implement in daily practice.** - explore **the barriers to and facilitators of intervention implementation** across various types of cancer patients at different stages. - improve **patients’ adherence to intervention protocols.** - **ensure long-term follow-up** to increase intervention **utilization** and **application in the clinical settings.** |
| **Recommendations for survivorship** | **Various aspects of survivorship** such as experiences of ongoing symptoms, financial toxicity, multi-morbidity, and psychological issues, including fear of cancer recurrence, mental health disorders, and stigma**,** need more investigation.  High-quality trials of **the effectiveness of survivorship care plans**, the distress thermometer, and decision aids, with physicians as participants, to determine whether these improve patient participation in consultations; promote shared decision making; and improve understanding, satisfaction, and treatment outcomes are needed. | Future research should focus on **various aspects of survivorship** such as experiences of ongoing symptoms, financial toxicity, multi-morbidity, and psychological issues, including fear of cancer recurrence, mental health disorders, and stigma.  High-quality trials of the **effectiveness of survivorship care plans**, the distress thermometer, and decision aids, with physicians as participants, to determine whether these improve patient participation in consultations; promote shared decision making; and improve understanding, satisfaction, and treatment outcomes are needed. |
| **Recommendations for patient necessities/ individualized needs** | Implementation of intervention outcomes should be collected before, during and after the treatment to present **data** **that can be personalized** according to the **patient’s necessities.**  **Considering the individual characteristics of the participants,** such as educational level, disease stage and treatment trajectory, when administering interventions to patients with cancer is recommended.  The **treatment choice** should be **based on stage and patient’s clinical condition.**  Future studies should consider **patient characteristics.**  The International Classification of Functioning, Disability and Health model can be used as a common framework **to help prioritize** **personalized goals** for patients.  To establish a systematic way of providing **individualized intervention,** further research is needed **to bridge the gap between various types of intervention research and clinical practice.** | Future studies should consider **patient characteristics** such as educational level, disease stage and treatment trajectory, when administering interventions **to patients with cancer.** Data should be collected before, during and after the treatment.  The data needs to be presented in a way that can be **personalized according to each patient’s needs**.  The International Classification of Functioning, Disability and Health model can be used as a common framework **to help prioritize** **personalized goals** for patients is recommended.  To establish a systematic way of **providing individualized intervention**, further research is needed to bridge the gap between various types of intervention research and clinical practice. |
| **Recommendations for implementation/ clinical practice** | **Leaflets and brochures should be provided in the language of the patients** or survivors affected by cancer**.**  **Doctors and families should become familiar with the beneficial effects of supportive interventions.**  Providers of supportive care in cancer should name **specific personnel** responsible for **disseminating particular information** to patients’ needs.  To **make cancer conference effective, professionals should consider them a critical part of their working** **agenda** and save time to prepare and attend conference.  **Conference time should be dedicated** to **avoiding discussing many cases in a short amount of time.**  It is recommended that **web-based interventions implement industry-standard data encryption** to ensure the security of private information.  Exploring **changes in symptom clusters or symptom networks** over time during **adjuvant treatment**, their **clinical practice is needed**.  Implementation research studies is recommended to examine and **maximize effectiveness, adoption, implementation and maintenance outcomes** of the intervention strategies over time.  **Future implementation studies are needed** to provide evidence about the reach, uptake, fidelity, and scalability of the tools discussed in this overview.  New **intervention programs** should ensure they have the potential to be implemented **within clinical practice**, designed and tested for **accessibility in large-scale implementation**.  Interventions tested in trials should be **further tested in real-world settings, especially at the population level.** | **Leaflets and brochures should be provided in the language of the patients** or survivors affected by cancer**.**  **Doctors and families should become familiar with the beneficial effects of supportive interventions.**  Providers of supportive care in cancer should name **specific personnel** responsible for **disseminating particular information** to patients’ needs.  To make **cancer conferences effective,** professionals should consider them a **critical part of their working** **agenda** and should be dedicated to **avoiding discussing too many cases in a short amount of time.**  It is recommended that **web-based interventions implement industry-standard data encryption** to ensure the security of private information.  Exploring **changes in symptom clusters or symptom networks** over time during **adjuvant treatment** is needed.  Future trials should **examine and maximize effectiveness**, ensure potential for **implementation** **within clinical practice**, and test accessibility, reach, uptake, fidelity, and scalability of the tools/interventions in **large-scale and real-world settings**. |
| **Recommendations for migrant and minority population needs** | **Supportive care needs of migrants and ethnic minority cancer patients and survivors** should be **investigated in detail in future research**.  **Information needs regarding sensitive topics of body image and sexuality should be investigated.**  As **differences in information and supportive care needs** are present between migrant and ethnic minority cancer patients and survivors, **medical personnel should never overlook the individual characteristics of a patient**. | Future research should focus on the **information and supportive needs**, **sensitive topics** such as body image, and sexuality with a particular focus on **migrant and ethnic minority cancer patients** and survivors. Medical personnel should **never overlook the individual characteristics** of a patient. |
| **Recommendations for comparing effectiveness of interventions** | Researchers should **adopt a comparative effectiveness approach and design trials** that allow real-world evaluation of interventions.  Future controlled trials should compare the **benefits of specific intervention implementations** **compared** with **usual care in a** population in real‐world settings.  Further research is needed to **examine comparative effectiveness of intervention modalities.**  **Future comparative effectiveness of interventions** research should pay attention to improving the reporting and methodological quality of trials. | **Future comparative effectiveness of interventions research should** compare the **benefits of specific intervention implementations** **compared** with **usual care in a** population in real‐world settings and pay attention to improving the reporting and methodological quality of trials. |
| **Recommendations for higher-quality study** | More well designed and large randomized controlled trials are needed **to provide a higher level of evidence** to confirm the supportive interventions.  **Improving the level of evidence** of the included systematic reviews, more original studies with rigorous study designs and **detailed descriptions of the intervention protocol**s are necessary.  Rigorous randomized controlled trials and systematic reviews are needed **to provide high-quality evidence for the specificity of interventions.**  There is a need for high-quality randomized controlled trials with a large sample size **to provide high-quality evidence.** | More well-designed, large randomized controlled trials with a substantial sample size are needed to conduct detailed **descriptions of the intervention protocols** and **to provide a higher level of evidence**. |
| **Recommendations for preventing bias** | Funding sources should be clearly declared in future publications to reduce **funding bias.**  **To prevent publication bias,** it is recommended that all clinical trials protocols on the topic **should register with a recognized platform.**  Future randomized controlled trials should **reduce publication bias by releasing randomized controlled trials protocols on trial registries.**  **Strictly controlling bias** in future original research is essential.  Future studies should **reduce the risk of methodological bias**.  Indicating a more rigorous design and **evaluation** is needed **to avoid blinding bias.**  Future studies should focus on developing high‐quality studies with longer follow‐ups and **reducing** **biased results.**  To achieve a comprehensive literature search, future systematic reviews should also search **gray literature, to minimize publication bias.** | **To prevent bias,** it is recommended that:   - all clinical trials protocols on the topic **should register with a recognized platform.** - funding sources should be clearly declared in future publications to reduce **funding bias.** - developing high‐quality studies with longer follow‐ups, and **strictly controlling bias** is essential. - future systematic reviews should search **gray literature, to reduce publication bias.** |
| **Recommendations for addressing evidence gaps** | Future systematic reviews should **explicitly report evidence gaps** in primary research.  Future studies should **prioritize robust primary studies to address gaps in the literature** for outcomes in the domains of health promotion**, chronic conditions, clinical structure, and decision-making.** | Future systematic reviews should **explicitly report evidence gaps** in primary research for outcomes in the domains of health promotion**, chronic conditions, clinical structure, and decision-making**. |
| **Recommendations for enhancing quality of life** | **Future larger randomized controlled trials and meta-analyses are needed**, to provide cancer patients, survivors and their families with the **best possible quality of life**.  More trials should be conducted by setting and subsequently **studying the same criteria**, such as weight loss, pain degree or symptom duration, **concerning the participants' quality of life during their treatment.** | Future larger randomized controlled trials and meta-analyses are needed to provide cancer patients, survivors and their families with the **best possible quality of life**. |

# Stage 4. Creating overarching themes and subthemes

| **Overarching theme** | **Subthemes** |
| --- | --- |
| **Recommendations for future review efforts** | To aid the development of evidence informed policy and best practice guidelines, **future reviews of literature** should:   - **adhere to high methodological and reporting standards and guidelines;** comply with the **PRISMA statement.** - **use standardized reporting of the parameters of the different interventions.** - **adhere more strictly to the AMSTAR-2, PRISMA**, and **GRADE criteria** for quality appraisal. - where appropriate consider selecting **randomized controlled trials of higher quality** and lower risk of bias to provide more rigorous evidence. - incorporate **a wider range of research types** and reviews (not **only Cochrane reviews)**, to **avoid overlooking** high-quality research that potentially contain **unbiased and important recommendations regarding real world implementation contexts**. - provide **exclusion list, and definitions of** outcomes **in their protocols.** - report evidence **based on separate objectives, interventional studies, and type of studies and explore subgroup analyses and potential subgroup effects through head-to-head comparisons.** - **explicitly report evidence gaps** in primary research for outcomes in the domains of health promotion, chronic conditions, clinical structure, and decision-making. - include a search of the **gray literature, to reduce publication bias.**   It is recommended that future research and high-quality trials should:   - include **large‐scale longitudinal research with long-term follow-up evaluations, and clearly defined targeted measurement indicators, strictly controlling bias** involving patients who have survived cancer or live with chronic illness. - use ecological transient assessment to dynamically measure symptoms. - focus on **various aspects of survivorship** such as experiences of ongoing symptoms, financial toxicity, multi-morbidity, and psychological issues, including fear of cancer recurrence, mental health disorders, and stigma. - focus on **various aspects of care delivery and organization for example ensuring** the effectiveness of survivorship care plans, the distress thermometer, and decision aids in improving patient participation in consultations; promoting shared decision making; and improving understanding, satisfaction, and treatment outcomes. - test accessibility, reach, uptake, fidelity, and scalability of the tools/interventions in **large-scale and real-world settings.** - assess whether **positive impacts from interventions can be maintained** in the long term in the real-world setting. - develop **symptomics using symptom network analysis.** - clarify the quality of the evidence regarding the **validity and reliability of tools and e measures** in the assessment of outcomes such as fatigue **within the target population,** to support a more coherent and robust evidence base. - include larger randomized controlled trials and meta-analyses to provide cancer patients, survivors and their families with the optimum evidence to inform the **best possible quality of life**. - **assess alternative outcome measures**, and the difference between **objective and subjective outcome measures** to understand **patients’ interpretations** of their conditions, and to enable early detection of side-effects. - provide consensus around a **core outcome set to measure psychological morbidity impact.** |
| **Recommendations for future trials** | To provide useful data for evidence syntheses and clinical practice guidelines, future well reported **randomised controlled trials should:**   - **register with a recognized platform.** - declare funding sources in future publications to reduce **funding bias** - **estimate the optimal sample size based on the existing research results, be fully powered to ensure sufficient sample size, aiming to ensure that the conclusions drawn from the research are valid.** - include **homogeneous samples of cancer** patients to allow for more definitive conclusions. - conduct **blinding, allocation concealment** and use **placebo in the control group** as appropriate. - explicitly **report patient-important outcomes**. - adopt **more specific quality of life measurement tools** such as the Short Form 36 questionnaire, and the European Organisation for Research and Treatment of Cancer Quality of Life Questionnaire-core. - employ **the incidence of adverse events as one of the main evaluation indices**. - **report implementation and results according to the CONSORT statement,** to provide a high methodological quality. |
| **Recommendations for future interventions and experimental studies** | Future intervention-based studies should:   - attain the **perspectives of patients and all stakeholders in the co-design of interventions and interventional studies.** - **seek to address the** impairing effects of cancer treatments, support home and working life, and address patient’s physical and psychological needs and optimise **patients’ experiences**. - evaluate the short and long-term effects of **pharmacological interventions, dietary supplements, herbal medicine, complementary and integrative medicine interventions, nutritional therapies, aerobic/non-aerobic exercise interventions**, **cryotherapy interventions, psychosocial interventions and web-based interventions** to enable more **robust conclusions about the efficacy** of interventions**.** This includes their impact on supporting the prevention and management of cancer and treatment related symptoms (e.g. pain, fatigue, and anorexia), symptom clusters, cancer recurrence and promoting health and wellbeing. - explore **changes in symptom clusters or symptom networks** over time during **adjuvant treatment.** - consider how individual can **overcome barriers to engagement in physical activity** and seek to evaluate suitable exercises for **patients with poor exercise acceptance.** - focus on **spirituality, social connections, body image and coping strategies as outcomes** in cancer patients. - investigate the impact of interventions in a **broader range of patient** populations. - use **telemedicine, tailored eHealth interventions, wearable health technology-based interventions and educational interventions** for symptoms such as cancer-related fatigue, **supporting chronic disease management, medication adherence and management, cancer screening, surveillance for recurrence, and disease prevention**, whilst considering selected populations' digital health literacy. - focus on the development of novel interventions to **improve patient-physician communication** and investigate communication competencies between **patients and healthcare providers**.   Future well-designed randomized controlled trials of interventions in order to provide high certainty of the evidence should:   - use a **comprehensive quality framework** for standardized evaluation of interventions in cancer survivors. - focus on the **effects of interventions on symptoms and assess symptoms** regularly **in clinical settings** to aid in **identifying effective therapies, treatments, and management strategies.** - use **The Medical Research Council framework** for complex interventions. - be based on **underlying theoretical model(s), behavior change techniques** and consider factors which might influence and modify the effect of interventions. - use **standardized reporting of the parameters of the different interventions and**  **establish a systematic way of reporting and providing individualized interventions.** - optimise **patients’ adherence to intervention protocols.** - clarify the presence of **short- and long-term toxicities of interventions.** - **assess how to sustain intervention effects** over a longer **follow‐up period**. - be **more transparent in the description of complex interventions** and routine care**, trial design, adherence to trial protocol and intervention,** including timing of assessment, the duration of the intervention. - describe the **treatment protocol/intervention according to the TIDieR checklist (**Template for the intervention description and replication), to help organize the reporting of interventions and **facilitate replication in other trials.**   To enable translation of evidence into practice, future intervention research should:   - explore **the barriers to and facilitators of intervention implementation** across various types of cancer patients at different stages. - focus on **interventions that save resources** and are **relatively easy to implement in daily practice.** - **ensure long-term follow-up** to increase intervention **utilization** and **application in the clinical settings.** - bridge the gap between various types of interventional research and application in clinical practice. - evaluate **economic factors**, including **cost analysis and adverse events**, to examine the potential **cost-effectiveness or cost-minimization of interventions**. Cost-effective interventions should be popularized in clinical practice. - develop **standard operating procedures, standards and guidelines** to guide implementors on **the optimal approach to deploy interventions, and delivery methods**.   compare the **benefits of specific intervention implementation strategies** **compared** with **usual care in a** population in real‐world settings. |
| **Recommendations for diverse diagnosis/patients** | Future research should:   - **expand the populations** included in research to include **different cancer types, age ranges reflecting the lifespan, diverse languages, demographic groups, educational levels, and include population in remote, rural, or low-resource settings.** - **prioritize inclusion of patients traditionally underrepresented in studies.** - **address the priorities of the patients and their families** in low- and middle-income countries. - consider the a**cceptability and effectiveness of interventions** **targeting people living beyond all types of cancer** especially those with baseline **poor overall quality of life**. - expand the understanding of **effective models of care in diverse cancer survivor populations** **including paediatric cancer survivors, adolescent and young adult survivor group**, **older adults**, and **a broader range of cancer types** as well as **advanced stages of the diseas**e. - personalize the data **according to each patient’s needs** and use The International Classification of Functioning, Disability and Health model as a common framework **to help prioritize** **personalized goals** for patients. - focus on the **information and supportive needs**, **sensitive topics** such as body image, and sexuality including **migrant and ethnic minority cancer patients** and survivors. - develop decision trees for selecting the most appropriate **model of care for individual cancer survivors**, along with implementation guides and standardized outcomes for evaluation. - address understanding the **unique cultural factors of indigenous populations worldwide** who are facing cancer. - consider breast cancer survivors with **BRCA1/2 gene mutations**, **women receiving tailored treatments, and women from low socioeconomic backgrounds, their complex health care needs, and those experiencing late effects.** - focus on **specific tumor types** and include direct comparisons between therapeutic options **amongst specific cancer population clusters and trajectories.** |
| **Recommendations for future policymakers, Health care managers, leaders and team and researchers** | It is recommended that clinicians/policymakers/researchers should:   - **provide leaflets and brochures in the language of the patients** or survivors affected by cancer**.** - **become familiar with the beneficial effects of supportive interventions.** - name **specific personnel** responsible for **disseminating particular information** addressing patients’ needs. - consider **cancer conferences as critical part of their working agenda**. - **implement industry-standard data encryption for web-based interventions,** to ensure the security of private information. - evaluate the use of **different technological platforms** to **provide telemedicine services.** - integrate palliative care into **primary care services,** to improve **access to palliative care** for patients living in remote areas. - prioritize access to palliative care for patients with **limited treatment options** or **persistent frailty** despite rehabilitative attempts. - assess **the country's readiness** for the **provision and integration of evidence-based care** and develop a **sufficient body of evidence** to support policy development. - **collaborate** with national and international organizations **to secure funding** for **improving healthcare provision**. - consider consensus **best‐practice standards,** including **standardized definitions and criteria** for cancer care management. - invest time and resources in training a competent care workforce and **supporting volunteers for the health of cancer patients**, as this is a **facilitator in addressing workforce shortages**. |
